# Ring Transformation of Cyclopropenes to Benzo‐Fused Five‐Membered Oxa‐ and Aza‐Heterocycles via a Formal [4+1] Cyclization

**DOI:** 10.1002/advs.202407931

**Published:** 2024-08-29

**Authors:** Fengyan Gu, Binyan Lin, Zhi‐Huan Peng, Shijie Liu, Yuanqing Wu, Mei Luo, Ning Ding, Qichen Zhan, Peng Cao, Zhi Zhou, Tao Cao

**Affiliations:** ^1^ School of Pharmacy Nanjing University of Chinese Medicine Nanjing Jiangsu 210023 China; ^2^ State Key Laboratory on Technologies for Chinese Medicine Pharmaceutical Process Control and Intelligent Manufacture Nanjing University of Chinese Medicine Nanjing Jiangsu 210023 China; ^3^ Key Laboratory of Molecular Target and Clinical Pharmacology & State Key Laboratory of Respiratory Disease School of Pharmaceutical Sciences & the Fifth Affiliated Hospital Guangzhou Medical University Guangzhou Guangdong 511436 China; ^4^ Jiangsu Provincial Medicinal Innovation Center Affiliated Hospital of Integrated Traditional Chinese and Western Medicine Nanjing University of Chinese Medicine Nanjing Jiangsu 210028 China; ^5^ The Quzhou Affiliated Hospital of Wenzhou Medical University Quzhou People's Hospital Quzhou Zhejiang 324000 China; ^6^ Gaoyou Hospital of Traditional Chinese Medicine Yangzhou Jiangsu 225600 China

**Keywords:** cyclopropenes, DFT calculations, heterocycles, late‐stage modifications, rhodium, ring transformation

## Abstract

In the context of the growing importance of heterocyclic compounds across various disciplines, numerous strategies for their construction have emerged. Exploiting the distinctive properties of cyclopropenes, this study introduces an innovative approach for the synthesis of benzo‐fused five‐membered oxa‐ and aza‐heterocycles through a formal [4+1] cyclization and subsequent acid‐catalyzed intramolecular *O*‐ to *N*‐ rearrangement. These transformations exhibit mild reaction conditions and a wide substrate scope. The applications in the late‐stage modification of complex molecules and in the synthesis of a potential PD‐L1 gene down‐regulator, make this method highly appealing in related fields. Combined experimental mechanistic studies and DFT calculations demonstrate Rh(III)‐mediated sequential C─H coupling/π‐allylation/dynamically favorable *O*‐attack route.

## Graphical abstract

Taking advantage of the unique ring‐opening properties of cyclopropenes, a versatile and efficient approach toward benzo‐fused five‐membered oxa‐ and aza‐heterocycles from the same raw materials is reported. With this practical method, a potential PD‐L1 down‐regulator with a novel isobenzofuranone *O*‐alkyl oxime moiety is synthesized.

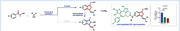

## 1 Introduction

**Figure 1:**
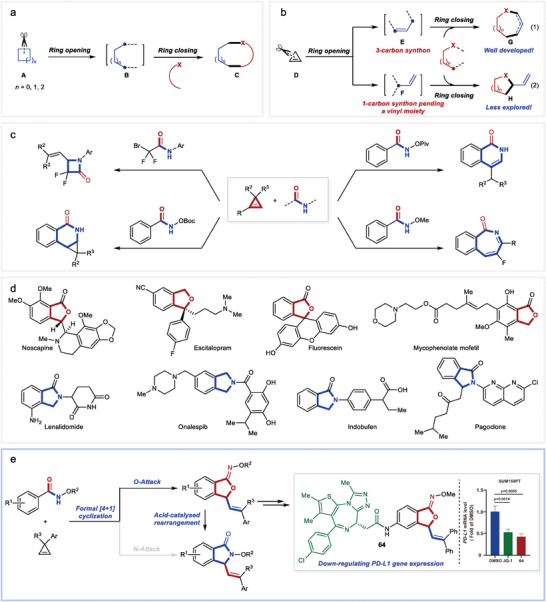
Background of this study. a) Intermolecular ring expansion by C─C cleavage. b) Two reaction paradigms for intermolecular stepwise ring‐opening/closing of cyclopropenes. c) Examples on the reactions of cyclopropenes with amides. d) Examples of natural products and drugs with benzo‐fused five‐membered oxa‐ and aza‐heterocyclic moieties. e) This study: formal [4+1] cyclization of cyclopropenes with *N*‐alkoxy benzamides for heterocycles synthesis.

The development of strategies for constructing heterocyclic compounds has always been a central focus for synthetic chemists. This research area has attracted significant attention^[^ ^1^, ^2^, ^3^, ^4^ ^]^ due to its pivotal role in drug discovery^[^ ^5^, ^6^ ^]^ and materials science.^[^ ^7^, ^8^ ^]^ Among the various strategies explored, the transformation of one cyclic compound into another has emerged as an efficient and intriguing approach.^[^ ^9^, ^10^ ^]^ One notable concept in recent studies is the “cut and sew” strategy for such ring transformations (**Figure** **1**a).^[^ ^11^, ^12^ ^]^ In essence, this approach involves breaking a C─C bond within a ring (referred to as ring **A**) to produce a linear synthon (referred to as **B**). By subsequently reacting both carbon termini of synthon **B**, an expanded ring (referred to as **C**) is formed, where all atoms from the original ring **A** are incorporated. This ring expansion strategy has proven successful in the construction of various heterocyclic compounds, employing functionalized three‐,^[^ ^13^, ^14^, ^15^, ^16^ ^]^ four‐,^[^ ^13^, ^17^, ^18^, ^19^ ^]^ and five‐membered rings,^[^ ^20^ ^]^ as well as other cyclic systems,^[^ ^21^, ^22^, ^23^, ^24^ ^]^ as starting materials.

In a related specific case, the cleavage of a C─C bond of cyclopropenes **D** ^[^ ^25^, ^26^ ^]^ typically leads to the transformation into synthon **E**, serving as three‐carbon components for the subsequent formation of heterocycles **G** (Figure 1b, Equation (1)).^[^ ^14^, ^15^, ^25^, ^26^, ^27^, ^28^, ^29^, ^30^, ^31^, ^32^, ^33^ ^]^ In contrast, the attractive but more challenged synthon **F** (Figure 1b, Equation (2)) can also be derived from cyclopropenes **D**, which represents a distinct one‐carbon component capable of forming two bonds on the same carbon atom, and with an attached vinyl group allowing for further modifications. While numerous transformations into acyclic compounds and all‐carbon cycles have been developed using synthon **F**,^[^ ^34^, ^35^, ^36^, ^37^ ^]^ it is worth noting that intermolecular cycloaddition reactions to form corresponding heterocyclic compounds (referred to as **H**) are relatively rare.^[^ ^38^ ^]^

*N*‐Alkoxy benzamides have been extensively utilized as starting materials in C─H functionalization reactions. In most cases, the resulting heterocycles incorporate a nitrogen atom from the amide bond, while the specifically selective incorporation of oxygen atom to form oxa‐heterocycles remains un‐addressed (only limited cases reported the formation of oxa‐heterocycles as side‐products^[^ ^39^ ^]^ or with unsatisfactory selectivity^[^ ^40^ ^]^). The diverse reactivity of cyclopropenes allows for producing various heterocycles through coupling with different amides. Known transformations include the synthesis of four‐, six‐, and seven‐membered rings using α‐bromodifluoroacetamides, *N*‐acyloxybenzamides, or *N*‐alkoxybenzamides (Figure 1c).^[^ ^28^, ^30^, ^38^, ^41^ ^]^ Notably, all these reactions exclusively incorporate the nitrogen atom into the products, forming aza compounds [Supplementary-material advs9389-supitem-0001].

Among the vast array of heterocyclic compounds, benzo‐fused five‐membered heterocycles containing either an oxygen or nitrogen atom represent two crucial core structures frequently encountered in natural products and pharmaceuticals (Figure 1d).^[^ ^2^, ^5^, ^6^, ^42^, ^43^, ^44^, ^45^, ^46^, ^47^, ^48^, ^49^ ^]^

Recognizing the significance of these cyclic compounds, we envisioned that under appropriate conditions, the reactions of cyclopropenes that undergo a ring‐opening/cyclization sequence with *N‐*alkoxy benzamides partner would lead to the formation of five‐membered heterocycles in a formal [4+1] cyclization process. With this concept in mind, we successfully achieved the highly selective consecutive C─H/C─C cleavage‐coupling,^[^ ^28^, ^29^, ^30^, ^31^, ^32^, ^33^, ^50^ ^]^ and an uncommon *O‐*attack cyclization (Figure 1e). Furthermore, during our investigations, we realized an acid‐catalyzed intramolecular *O*‐ to *N*‐rearrangement, resulting in the production of *N‐*attack cyclization products. A series of control experiments and density functional theory (DFT) calculations have been conducted and reveal that the steric interactions between the Cp^*^ ligand on rhodium center and the alkoxyl group in the substrates play a crucial role in determining the selectivity between *O*‐attack and *N*‐attack. Remarkably, when the well‐studied bromodomain and extra‐terminal (BET) protein BRD4 inhibitor JQ‐1 was linked with the benzo‐fused oxa‐five‐membered ring, compound **64** exhibited an enhanced ability to down‐regulate programmed cell death ligand 1 (PD‐L1) gene expression. Given the critical role of PD‐L1 inhibition in cancer immunotherapy, the structures obtained in this study hold significant promise for further applications.

## 2 Results and Discussion

### 2.1 Method Development

At the outset, the reaction between *N‐*methoxy benzamide **S1** and 3,3‐diphenylcyclopropene **S2**, catalyzed by [Cp^*^RhCl_2_]_2_ (2.5 mol%) and with the presence of Ag_2_CO_3_ (2 equiv) in dichloromethane at room temperature, yielded no desired products (**Table** **1**, Entry 1). Surprisingly, the addition of HOAc (2 equiv) resulted in the formation of products **1** and **2** through *O*‐ and *N*‐attack, respectively, with a 3.9:1 ratio (Table 1, Entry 2). Solvent screening identified methanol as the most suitable choice (Table 1, Entry 3–7). Substituting HOAc with NaOAc led to a significant decrease in yields (Table 1, Entry 8). Further optimization efforts involved experimenting with various oxidants. While AgSbF_6_, AgF, AgOMs, and AgOTf did not enhance yields or selectivity (Table 1, Entry 9–12), the use of AgOAc as the oxidant significantly improved the NMR yield of **1** to 81%, with a product ratio of 11.6 (Table 1, Entry 13). Intriguingly, in the absence of HOAc, the yield of **1** and the selectivity remained unaffected (Table 1, Entry 14). Increasing the reaction temperature resulted in lower yields of **1** (Table 1, Entry 15,16). Furthermore, employing 5 mol% of [Cp^*^RhCl_2_]_2_ further improved the yield of **1** to 92% (Table 1, Entry 17). As a result, the reaction of *N*‐alkoxy benzamides with cyclopropenes in the presence of [Cp^*^RhCl_2_]_2_ (5 mol%) and AgOAc (2 equiv) in methanol at room temperature have been established as the standard conditions for further investigation.

**Table 1:** Optimization of the reaction conditions.

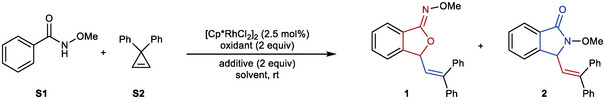						
Entry^a)^	Oxidant	Solvent	Additive	Yield of 1 [%]^b)^	Yield of 2 [%]^b)^	Ratio of 1/2
1	Ag_2_CO_3_	DCM	–	trace	Trace	–
2	Ag_2_CO_3_	DCM	HOAc	55	14	3.9
3	Ag_2_CO_3_	DCE	HOAc	66	17	3.9
4	Ag_2_CO_3_	TFE	HOAc	5	4	1.2
5	Ag_2_CO_3_	H_2_O	HOAc	15	4	3.8
6	Ag_2_CO_3_	EtOH	HOAc	47	20	2.4
7	Ag_2_CO_3_	MeOH	HOAc	65	12	5.4
8	Ag_2_CO_3_	MeOH	NaOAc	38	6	6.3
9	AgSbF_6_	MeOH	HOAc	trace	Trace	–
10	AgF	MeOH	HOAc	35	3	11.7
11	AgOMs	MeOH	HOAc	trace	Trace	–
12	AgOTf	MeOH	HOAc	trace	Trace	–
13	AgOAc	MeOH	HOAc	81	7	11.6
14	AgOAc	MeOH	–	81 (72)^c)^	7	11.6
15^d)^	AgOAc	MeOH	–	75	7	10.7
16^e)^	AgOAc	MeOH	–	66	7	9.4
17^f)^	AgOAc	MeOH	–	92 (86)^c)^	7	13.1

^a)^ Reaction conditions: **S1** (1 equiv), **S2** (1.5 equiv), [Cp^*^RhCl_2_]_2_ (2.5 mol%), oxidant (2 equiv), w/ or w/o additive (2 equiv), solvent (0.1 M), room temperature;

^b)^ Yield determined by ^1^H NMR analysis of the crude mixtures with dibromomethane as internal standard;

^c)^ Yield of isolated product;

^d)^ Reaction at 40 °C;

^e)^ Reaction at 80 °C;

^f)^ Reaction with [Cp^*^RhCl_2_]_2_ (5 mol%).

### 2.2 Reaction Compatibility and Late‐Stage Modification of Complex Molecules

**Figure 2:**
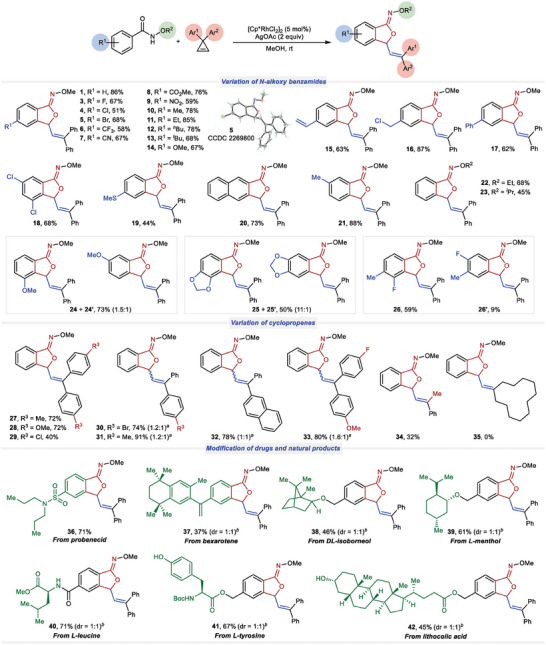
Scope of *N*‐alkoxy benzamides and cyclopropenes in the formal [4+1] cyclization to form isobenzofuranone *O*‐alkyl oximes. *^a^* Ratio of the stereoisomers on C═C bond as determined by ^1^H NMR analysis of the product. *^b^* The dr value in parentheses was based on original chiral centers of the substrate.

After establishing the optimized reaction conditions, our exploration of substrate scope commenced (**Figure** **2**). In reactions involving 3,3‐diphenylcyclopropene, *p*‐halo‐substituted *N*‐methoxy benzamides yielded *O*‐attack products **3**–**5** in the range of 51–68% yields. The structure of **5** has been unambiguously confirmed by the single crystal X‐ray diffraction.^[^ ^51^ ^]^ Notably, both electron‐withdrawing and electron‐donating groups were well‐tolerated in this reaction. For electron‐withdrawing substituents like trifluoromethyl, cyano, ester, and nitro groups, products **6**–**9** were obtained with yields ranging from 58% to 76%. Similarly, electron‐donating alkyl groups and a methoxy group yielded products **10**–**14** with yields of 67–85%. Encouragingly, the presence of reactive vinyl and chloromethyl groups did not interfere with the reaction, leading to the formation of products **15** and **16** with yields of 63% and 87%, respectively. Products **17** with a phenyl group and **18** with two chloro atoms were obtained with yields of 62% and 68%, respectively. It's worth noting that in the presence of a coordinating methyl sulfur group, the corresponding product **19** was obtained with a slightly reduced yield of 44%, highlighting the strong directing ability of the *N*‐methoxy amide group. This effect outweighed the influence of the sulfur atom, which could potentially affect the transition metal catalysts.^[^ ^52^ ^]^ Interestingly, reactions involving the β‐naphthylamide and the *m*‐methylbenzamide predominantly occurred at the less hindered positions, resulting in products **20** and **21** with excellent regioselectivity, yielding 73% and 88%, respectively. Not surprisingly that *N*‐ethoxy and *N*‐isopropyoxy benzamides reacted smoothly in this reaction (**22** and **23**). However, when a *m*‐methoxy substituent was present, a pair of regioisomers **24** and **24′** formed in a ratio of 1.5:1. A similar outcome was observed with a piperonylic acid derivative, producing products **25** and **25′** with a high regioselectivity of 11:1. Finally, 3‐fluoro‐*N*‐methoxy‐4‐methyl benzamide yielded separable **26** and **26′** in yields of 59% and 9%, respectively. The observed regioselectivity for **24**–**26** may be attributed to the directing ability of oxygen atom towards rhodium center, or the strong electron‐withdrawing inductive effect of oxygen and fluorine atoms for a more acidic yet more hindered *ortho* C─H bond.

Various cyclopropenes were then investigated in this reaction. Cyclopropenes bearing dimethyl, dimethoxy, and dichloro substituents yielded **27**–**29** with yields ranging from 40% to 72%. Cyclopropenes substituted with two different aryl groups typically produced a mixture of two stereoisomers with high yields (**30**–**33**, 74–91% yields). The use of 3‐methyl‐3‐phenylcyclopropene was also successful in this reaction, resulting in **34** as a single isomer with a moderate yield of 32%. However, the attempt to obtain the cycloalkyl product **35** was not successful.

Considering the simple operation and mild conditions of the developed protocol, the application of this method has been demonstrated in late‐stage modification of several complex molecules. As a result, derivatives of pharmaceutical compounds (**36** and **37**), terpenoids (**38** and **39**), amino acids (**40** and **41**), and a steroid (**42**) have been efficiently synthesized using this approach.

### 2.3 Synthesis of *N*‐Alkoxy Isoindolinones and Assessment of the Potential for Post‐Modifications

**Figure 3:**
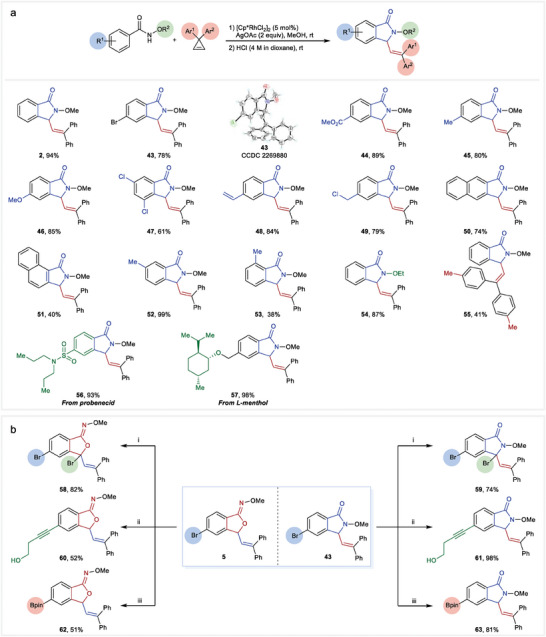
a) Synthesis of *N*‐alkoxy isoindolinones and b) assessment of the potential for post‐modifications. Reaction conditions: i) NBS (1.5 equiv), MeCN, 40 °C. ii) Pd(PPh_3_)_2_Cl_2_ (1 mol%), CuI (1 mol%), 3‐butyn‐1‐ol (1.2 equiv), Et_3_N, 80 °C. iii) Pd(dppf)Cl_2_ (5 mol%), B_2_pin_2_ (1.5 equiv), KOAc (3 equiv), dioxane, 90 °C.

Having established the efficient protocol for accessing a series of oxa‐heterocycles, we assumed that the intriguing aza‐heterocycles might be also realized by appropriately adjusting the reaction conditions thus leading to divergent products from the same raw materials. To our delight, we discovered that treating the crude products obtained in the rhodium‐catalyzed [4 + 1] reactions with HCl in 1,4‐dioxane resulted in a complete transformation into *N*‐alkoxy isoindolinones. Several substrates have been explored with this two‐step transformation (**Figure** **3**a). *N*‐Methoxy benzamide provided compound **2** in an impressive yield of 94%. Substrates featuring both electron‐withdrawing groups (such as halo and methoxycarbonyl groups) and electron‐donating groups (such as alkyl and methoxy groups) yielded the corresponding isoindolinones in good to excellent yields (**43**–**47**). The structure of **43** has been unambiguously confirmed by the single crystal X‐ray diffraction.^[^ ^51^ ^]^ Notably, reactive vinyl and chloromethyl groups remained unaffected during the reaction, leading to the formation of **48** and **49**. The reaction involving β or α‐naphthalene starting materials resulted in the synthesis of **50** and **51** in yields of 74% and 40%, respectively. Methyl groups situated at *meta*‐ or *ortho*‐ positions produced **52** and **53** with outstanding regioselectivity. *N*‐Ethoxy benzamide also smoothly underwent the reaction to form **54**. Furthermore, the reaction with 3,3‐di(4‐methylphenyl)cyclopropane yielded **55** in a moderate yield. Substrates derived from probenecid and *L*‐menthol proceeded smoothly, affording **56** and **57** in excellent yields.

With the isobenzofuranone *O*‐alkyl oximes and *N*‐alkoxy isoindolinones in hand, we aimed to assess the potential for post modification reactions of these two types of rings. Compounds **5** and **43**, each containing a functionalizable bromine atom, were selected as representatives (Figure 3b). To our delight, neither rearrangement nor degradation of the heterocyclic moieties was observed under radical bromination conditions (**58** and **59**), palladium‐catalyzed Sonogashira coupling (**60** and **61**), and borylation conditions (**62** and **63**). These results demonstrated the potential of these two types of heterocyclic moieties as building blocks for construction of complex molecules.

### 2.4 Synthesis of a Potential PD‐L1 Down‐Regulator

**Figure 4:**
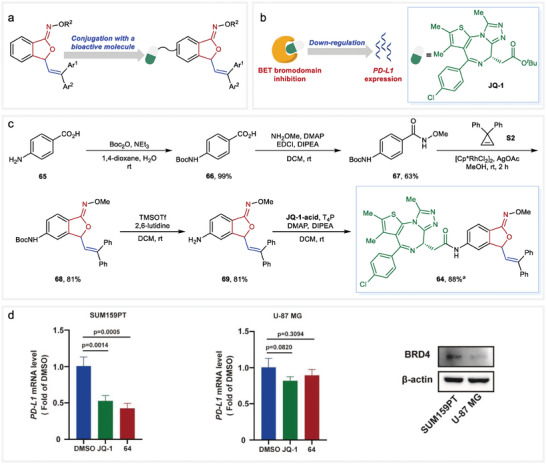
Synthesis and biological evaluation of compound **64**. a) The concept of conjugation of 3‐vinyl isobenzofuranone *N*‐alkoxy oximes with a bioactive molecule. b) Small‐molecule BET bromodomain inhibitors (such as JQ‐1) down‐regulate the expression of PD‐L1. c) Synthetic route of compound **64**. d) Evaluation of PD‐L1 gene down‐regulating effect. Data shown represent mean ± SD, and *p*‐values were calculated using one‐way ANOVA. *^a^* dr = 1:1.

Owing to the structural similarity to the promising 3‐substituted phthalide motif, we rationally supposed that the obtained 3‐vinyl isobenzofuranone *O*‐alkyl oximes should have significant bioactivities and act as distinctive pharmacophores for drug design and disease therapy. With this in mind and encouraged by the profound potential of such skeletons for convenient derivatizations, we aimed to construct bioactive molecules with this rarely studied structure. As a proof‐of‐concept study, we chose to conjugate the isobenzofuranone moiety with a well‐studied bioactive molecule to preliminarily evaluate the pharmacological effect of this kind of structure (**Figure** **4**a).

Within the area of cancer therapy, restoration of antitumor immunity has proven highly effective through the use of antibodies to block PD‐L1 signaling.^[^ ^53^, ^54^ ^]^ However, antibody‐based approaches can sometimes lead to severe immune‐related adverse events due to dysregulation of the balance of immune system.^[^ ^55^, ^56^ ^]^ Recent research highlights a novel approach that small‐molecule BET bromodomain inhibitors can effectively suppress PD‐L1 gene expression of cancer cells.^[^ ^57^, ^58^ ^]^ Be aware of the significance of developing small‐molecule PD‐L1 down‐regulators in cancer immunotherapy,^[^ ^59^ ^]^ we decided to conjugate the novel 3‐vinyl isobenzofuranone *N*‐alkoxy oxime moiety obtained in this study to the BET bromodomain inhibitor, JQ‐1 (Figure 4b),^[^ ^60^ ^]^ to assess the impact of this structure on the ability of down‐regulating PD‐L1 expression.

Compound **64** has been synthesized as a representative example (Figure 4c). Protection of *p*‐aminobenzoic acid (**65**) with a Boc group resulted in the production of compound **66**, which was subjected to condensation with methoxyamine, yielding compound **67**. The crucial formal [4+1] reaction with cyclopropene **S2** proceeded smoothly, resulting in the formation of the key intermediate **68** in a yield of 81%. The combination of TMSOTf with 2,6‐lutidine effectively removed the Boc group to form **69**. Subsequently, the coupling reaction with JQ‐1‐acid completed the synthesis of compound **64**.

When treated with either JQ‐1 or compound **64**, a significant suppression of PD‐L1 expression was observed in the SUM159PT cell line, as indicated by the results of quantitative PCRs (Figure 4d). Remarkably, compound **64** demonstrated even higher efficacy than JQ‐1. In contrast, neither JQ‐1 nor **64** exhibited a significant down‐regulation effect for the U‐87 MG cell line. Considering that JQ‐1 suppresses PD‐L1 expression typically through BRD4 inhibition,^[^ ^57^ ^]^ immunoblotting has been conducted in both cell lines. The result showed a much higher BRD4 protein expression in the SUM159PT cell line compared to the U‐87 MG cell line, thus accounting for the poor down‐regulation effect of PD‐L1 in the U‐87 MG cell line (Figure 4d). Taken together, these results demonstrated that the 3‐vinyl isobenzofuranone *O*‐alkyl oxime tethered JQ‐1 derivative **64** featured similar acting mechanisms with an improved PD‐L1 down‐regulated index, which further strengthen the utility potential of the developed protocol.

### 2.5 Combined Experimental and Computational Mechanistic Study

**Figure 5:**
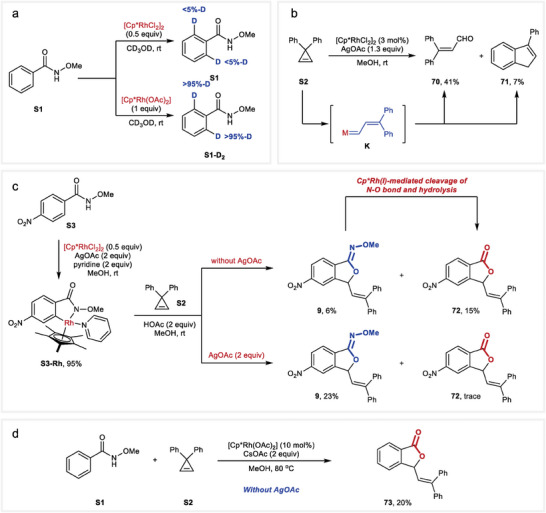
Experimental mechanistic studies. a) H/D exchange with rhodium catalysts. b) Detection of a metal carbene species. c) Synthesis of a rhodacyle **S3‐Rh** and stoichiometric reactions. d) Preliminary attempt for a redox‐neutral transformation.

A series of control experiments was next conducted to probe the reaction mechanism (**Figure** **5**). The reaction of *N*‐methoxybenzamide (**S1**) with [Cp^*^RhCl_2_]_2_ (0.5 equiv) in the absence of **S2** in CD_3_OD resulted in negligible deuteration at *ortho* positions of **S1**, while the reaction with Cp^*^Rh(OAc)_2_ (1 equiv) afforded an average of more than 95%‐D at both *ortho* positions (Figure 5a). These results indicated that Cp^*^Rh(OAc)_2_ should be the active catalyst for C─H cleavage. On the other hand, when cyclopropene **2** was subjected to the reaction conditions without the presence of compound **S1**, the formation of 3,3‐diphenylacrylaldehyde **70** and 3‐phenyl‐1*H*‐indene **71** was observed, indicating the formation of a metal carbene species **K** (Figure 5b).^[^ ^31^, ^61^, ^62^ ^]^

To further investigate the potential intermediate of this reaction, several stoichiometric reactions were conducted. The rhodacycle complex **S3‐Rh**,^[^ ^63^ ^]^ stabilized with a pyridine ligand, was successfully synthesized in 95% yield by the reaction of *N*‐methoxy p‐nitrobenzamide (**S3**) with [Cp^*^RhCl_2_]_2_ (0.5 equiv) in the presence of AgOAc (2 equiv) and pyridine (2 equiv) in MeOH. Surprisingly, the reaction of **S3‐Rh** with cyclopropene **S2** without the addition of AgOAc yielded product **9** alongside the formation of lactone **72**. We hypothesized that **72** was formed through the cleavage of the N─O bond in **9** mediated by the in situ generated Cp^*^Rh(I) followed by hydrolysis. This observation indicated that silver salt probably did not play a role in the formation of the product **9**. Furthermore, the absence of an appropriate oxidant ruled out a possible Rh(III)‐Rh(IV)‐Rh(II) mechanism (Figure 5c).^[^ ^64^, ^65^ ^]^

Moreover, when the reaction of **S3‐Rh** with **S2** was conducted in the presence of AgOAc, lactone **72** was generated in only trace amounts. We postulated that AgOAc oxidized the in situ generated Cp^*^Rh(I) to Cp^*^Rh(III), thereby preventing the cleavage of the N─O bond in **9**. This assumption was further supported by a preliminary attempt at a catalytic redox‐neutral transformation. Consequently, the reaction of **S1** and **S2** in the absence of AgOAc yielded lactone **73** in 20% yield (Figure 5d).

**Figure 6:**
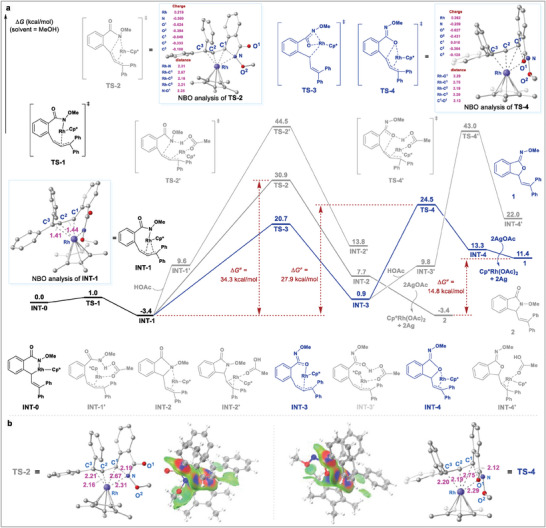
Computational mechanistic studies. a) Computed Gibbs free energy changes of the C─N/C─O cyclization processes. b) Weak interaction (IGMH) analysis using Multiwfn (version 3.7), isovalue = 0.005.

In order to further clarify the detailed mechanism, in particular, to unveil the origin of the selectivity observed in this transformation, the density functional theory (DFT) calculations were next performed using Gaussian 09 (**Figure** **6**a).^[^ ^66^, ^67^ ^]^ The six‐membered rhodacycle **INT‐0** was rationally selected as the starting point with MeOH being the solvent, which facilely converted into a more stable η^3^ π‐allyl rhodium species **INT‐1** featuring typical homogenization of the bond length with a free energy of −3.4 kcal mol^−1^. From INT‐1, two cyclization modes involving the formation of C─N or C─O bond via intramolecular nucleophilic substitution of different atoms to the π‐allylrhodium moiety were calculated. The result revealed that the C─N annulation occurred via **TS‐2** (*∆*G^≠^ = 30.9 kcal mol^−1^) with a relatively high energy barrier of 34.3 kcal mol^−1^ (from **INT‐1** to **TS‐2**) to deliver the isoindolinone skeleton **INT‐2**. As a comparison, the amide‐oxime tautomerism via **TS‐3** (*∆*G^≠^ = 20.7 kcal mol^−1^) afforded the *O*‐ligated intermediate **INT‐3**, which underwent the attack of the oxygen atom to the π‐allylrhodium moiety via **TS‐4** (*∆*G^≠^ = 24.5 kcal mol^−1^) to give the isobenzofuranone skeleton **INT‐4** with an energy barrier of 27.9 kcal mol^−1^ (from **INT‐1** to **TS‐4**).^[^ ^68^ ^]^ An alternative protonolysis/S_N_2‐type cyclization process with the assistance of HOAc from either **INT‐1** or **INT‐3** could be ruled out owing to the relatively high energy barriers of 47.9 kcal mol^−1^ (from **INT‐1** to **TS‐2′**) and 46.4 kcal mol^−1^ (from **INT‐1** to **TS‐4′**), suggesting that a synergetic rather than a stepwise cyclization process was more favored for the formation of the isobenzofuranone scaffold.

Further natural bond orbital (NBO) analysis of the key transition states demonstrated that the O^1^ featured more negative charge in comparison with N (**TS‐4**: −0.627 vs **TS‐2**: −0.300), thus furnishing a shorter C^1^─O^1^ length in **TS‐4** (2.12 Å vs 2.19 Å in **TS‐2**). These results provided adequate evidence to support that the C─O cyclization mode was more dynamically favorable and in good line with the experimental observation. In contrast, the isoindolinone product **2** was thermodynamically more stable compared with isobenzofuranone **1** (*∆G* ^≠^ = 14.8 kcal mol^−1^), which accounted for the acid‐enabled facile conversion of isobenzofuranones into isoindolinone derivatives. The weak interaction (IGMH) analysis of **TS‐2** and **TS‐4** was then carried out using Multiwfn (version 3.7). As shown in Figure 6b, both transition states exhibited distinct Rh‐*π* coordination interaction, and a strong N─C^1^ or O─C^1^ attractive interaction was detected in **TS‐2** and **TS‐4** respectively. Additionally, an obvious van der Waals interaction between the ‐OMe moiety and the Cp^*^ ligand was observed in **TS‐2**, implying a crowded configuration for such transition state. In contrast, no obvious van der Waals interaction was detected between the directing group and Cp^*^ ligand in **TS‐4** owing to the modified configuration, which probably accounted for the relatively good stability of **TS‐4** and low energy barrier for *O*‐attack cyclization.^[^ ^69^ ^]^

**Figure 7:**
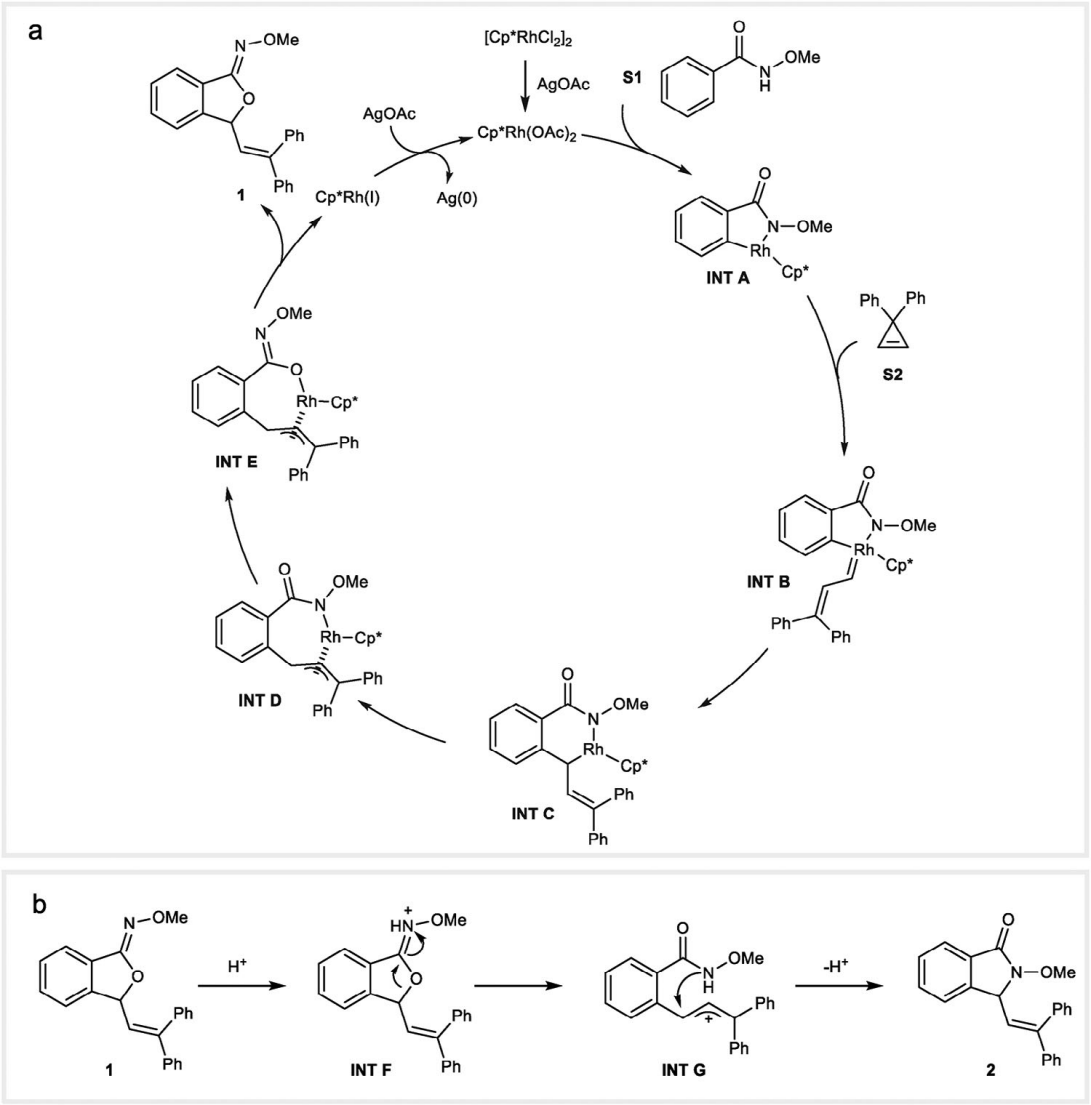
A plausible mechanism. a) Catalytic cycle for the generation of compound **1**. b) Proposed mechanism for the *O*‐ to *N*‐rearrangement.

Based on the experimental and computational findings and in consideration of previous research,^[^ ^40^ ^]^ we propose a plausible mechanistic pathway (**Figure** **7**). The process commences with the formation of the cationic Cp^*^Rh(III) species, arising from the interaction between [Cp^*^RhCl_2_]_2_ and AgOAc. This catalytic species subsequently engages in a C─H activation event with compound **S1**, yielding **INT A**. Subsequently, the interation of **INT A** with cyclopropene **S2** results in the formation of **INT B**. An intramolecular 1,1‐insertion process leads to the generation of an η^1^ complex **INT C**, which, through a η^1^ to η^3^ rearrangement, forms the allyl complex **INT D**. The steric hindrance between the methoxy group and the methyl groups on the Cp^*^ ring leads to a switch from *N*‐ligation to *O*‐ligation, resulting in the formation of **INT E**. Reductive elimination of **INT E** produces the ultimate product **1** and releases a Cp^*^Rh(I) species, which is oxidized by AgOAc to regenerate the cationic Rh(III) catalyst (Figure 7a).

In the *O*‐ to *N*‐ rearrangement process triggered by HCl treatment, compound **1** initially undergoes protonation, forming **INT F**. This intermediate then proceeds through a C─O bond cleavage, leading to the creation of **INT G**. Subsequent intramolecular *N*‐attack and a final proton elimination event result in the formation of the thermodynamically more stable product **2**, thus completing the reaction pathway (Figure 7b).

## 3 Conclusion

In conclusion, our study has unveiled a versatile and efficient approach for the synthesis of isobenzofuranone *O*‐alkyl oximes and *N*‐alkoxy isoindolinones through a strategic formal [4+1] cyclization of cyclopropenes. This methodology not only offers a diverse array of heterocyclic compounds but also demonstrates its applicability to the late‐stage modification of complex molecules and to the synthesis of molecules with potential biological activity. Mechanistic investigations shed light on the involvement of a key metal carbene species, enhancing our understanding of the reaction pathway. With its broad substrate scope, mild reaction conditions, and perfect atom‐economy, this approach presents an attractive platform for the synthesis of valuable compounds in the fields of organic chemistry and drug discovery, promising innovative avenues for future research and applications.

## Conflict of Interest

The authors declare no conflict of interest.

## Supporting information

Supporting Information

Supporting Information

## Data Availability

The data that support the findings of this study are available in the supplementary material of this article.
