# Supplementary material for: Ring Transformation of Cyclopropenes to Benzo‐Fused Five‐Membered Oxa‐ and Aza‐Heterocycles via a Formal [4+1] Cyclization
Source: Adv Sci (Weinh). 2024 Aug 29;11(40):2407931. doi: 10.1002/advs.202407931 (PMC11516165; doi:10.1002/advs.202407931)
Supplement: Supplementary file 1 — Supporting Information [file ADVS-11-2407931-s001.pdf]

ADVANCED SCIENCE
Open Access

Supporting Information

for *Adv. Sci.*, DOI 10.1002/advs.202407931

Ring Transformation of Cyclopropenes to Benzo-Fused Five-Membered Oxa- and Aza-Heterocycles via a Formal [4+1] Cyclization

Fengyan Gu, Binyan Lin, Zhi-Huan Peng, Shijie Liu, Yuanqing Wu, Mei Luo, Ning Ding, Qichen Zhan, Peng Cao, Zhi Zhou* and Tao Cao**

Supporting Information

Ring Transformation of Cyclopropenes to Benzo-Fused Five-Membered Oxa- and Aza-Heterocycles via a Formal [4+1] Cyclization

Fengyan Gu, Binyan Lin, Zhi-Huan Peng, Shijie Liu, Yuanqing Wu, Mei Luo, Ning Ding, Qichen Zhan, Peng Cao, Zhi Zhou,* and Tao Cao**

Table of Contents

1. Supplementary Notes........S3

2. Supplementary Methods........S3

2.1 Synthesis of isobenzofuranone *O*-alkyl oximes........S3

2.2 Synthesis of *N*-alkoxy isoindolinones........S27

2.3 Transformation of products to assess the potential for post-modifications. S37

2.4 Synthesis of a potential PD-L1 down-regulator........S41

2.5 Biological activity evaluation. S44

2.6 Experimental mechanistic studies........S45

2.7 Computational Mechanistic Studies S49

3. Supplementary References........S119

4. ^{1}H NMR, ^{13}C NMR, and ^{19}F NMR Spectra S123

1. Supplementary Notes

Commercially available reagents and solvents for synthesis of compounds were purchased from Adamas, Sinopharm, Sigma-Aldrich and used without further purification. All the temperatures are referred to the preheated oil baths used. ^{1}H NMR spectra were recorded on a Bruker AVANCE III 500 spectrometer and are internally referenced to residual protic $CDCl_3$ (7.26 ppm) or DMSO-d_6 (2.50 ppm). The data are reported as follows: chemical shift (δ ppm), multiplicity (s = singlet, d = doublet, t = triplet, q = quartet, m = multiplet, dd = doublet of doublets, dt = doublet of triplets, td = triplet of doublets, tt = triplet of triplets), coupling constant (Hz), and integration. ^{13}C NMR spectra were recorded on a Bruker AVANCE III 500 spectrometer and data are reported in terms of chemical shift relative to $CDCl_3$ (77.10 ppm) or or DMSO-d_6 (39.50). ^{19}F NMR spectra were recorded on a Bruker AVANCE III 500 spectrometer. The *N*-alkoxy benzamides[S1-S5] and cyclopropenes[S6-S10] were prepared according to published procedures.

2. Supplementary Methods

2.1 Synthesis of isobenzofuranone *O*-alkyl oximes.

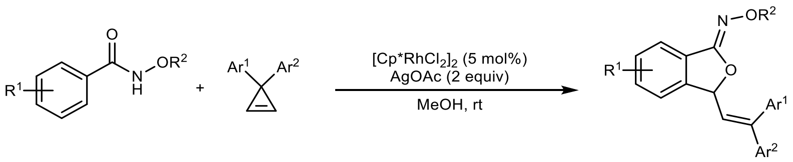

General procedure I: To a 25 mL reaction tube equipped with a magnetic stir bar was added *N*-alkoxy benzamide (1.0 equiv), cyclopropene (1.5 equiv), $[Cp^{*}RhCl_2]_2$ (5 mol%), AgOAc (2 equiv), and MeOH (0.1 M) successively. After being stirred at room temperature for indicated time, the reaction was complete as monitored by TLC. The mixture was filtered through a short pad of silica gel, washed with ethyl acetate, and

concentrated in vacuo to afford corresponding isobenzofuranone *O*-alkyl oxime via flash chromatography on silica gel.

(1) (*Z*)-3-(2,2-Diphenylvinyl)isobenzofuran-1(3*H*)-one *O*-methyl oxime (1) (GFY-3-19, GFY-3-37).

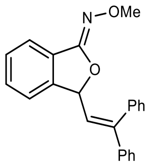

1

Following General Procedure I, after being stirred overnight, **1** was afforded as a white solid (0.2 mmol scale, 58.9 mg, 86%), eluent (0-7% of ethyl acetate in petroleum ether): m.p.= 139.3-140.9 ^{o}C (ethyl acetate/petroleum ether); ^{1}H NMR (500 MHz, CDCl3) δ 7.68 (d, *J* = 7.6 Hz, 1H), 7.49 - 7.45 (m, 1H), 7.44 - 7.42 (m, 2H), 7.41 - 7.36 (m, 4H), 7.33 - 7.30 (m, 1H), 7.27 (d, *J* = 1.3 Hz, 5H), 6.12 (d, *J* = 9.9 Hz, 1H), 5.99 (d, *J* = 9.9 Hz, 1H), 3.96 (s, 3H); ^{13}C NMR (126 MHz, $CDCl_3$) δ 156.07, 147.82, 144.01, 140.72, 138.16, 130.90, 129.91, 128.91, 128.54, 128.41, 128.29, 128.23, 128.06, 127.73, 123.58, 122.18, 121.70, 84.18, 62.72; HRMS calcd for $C_{23}H_{19}NNaO_2^+$ ($[M+Na]^+$): 364.1308; found: 364.1315.

(2) (*Z*)-3-(2,2-Diphenylvinyl)isobenzofuran-1(3*H*)-one *O*-methyl oxime (3) (GFY-3-49).

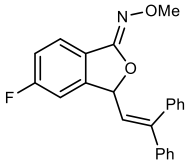

3

Following General Procedure I, after being stirred overnight, **3** was afforded as a white solid (0.2 mmol scale, 47.9 mg, 67%), eluent (0-6% of ethyl acetate in petroleum ether): m.p.= 140.0-149.9 ^{o}C (ethyl acetate/petroleum ether); ^{1}H NMR (500 MHz, $CDCl_3$) δ 7.65 (dd, *J* = 8.5, 4.8 Hz, 1H), 7.49 - 7.41 (m, 2H), 7.43 - 7.37 (m, 1H), 7.40 - 7.34 (m, 2H), 7.32 - 7.26 (m, 5H), 7.11 (td, *J* = 8.7, 2.3 Hz, 1H), 6.99 (dd, *J* = 7.8, 2.3 Hz, 1H), 6.08 (d, *J* = 9.9 Hz, 1H), 5.97 (d, *J* = 9.9 Hz, 1H), 3.95 (s, 3H); ^{13}C NMR (126 MHz, $CDCl_3$) δ 164.59 (d, *J* = 251.9 Hz), 155.09, 148.49, 146.35 (d, *J* = 8.9 Hz), 140.50,

137.97, 129.83, 128.64, 128.46, 128.29, 128.20, 127.74, 124.37, 123.57 (d, *J* = 9.5 Hz), 122.83, 116.90 (d, *J* = 23.9 Hz), 109.51 (d, *J* = 24.4 Hz) 83.64, 62.77. ^{19}F NMR (471 MHz, $CDCl_3$) δ -108.21; HRMS calcd for $C_{23}H_{19}FNO_2^+$ ($[M+H]^+$): 360.1394; found: 360.1400.

(3) (*Z*)-5-Chloro-3-(2,2-diphenylvinyl)isobenzofuran-1(3*H*)-one *O*-methyl oxime (4) (GFY-3-50).

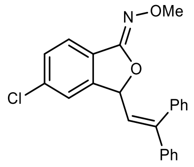

4

Following General Procedure I, after being stirred overnight, **4** was afforded as a white solid (0.2 mmol scale, 38.2 mg, 51%), eluent (0-7% of ethyl acetate in petroleum ether): m.p.= 43.8-45.5 °C (ethyl acetate/petroleum ether); ^{1}H NMR (500 MHz, $CDCl_3$) δ 7.60 (d, *J* = 8.3 Hz, 1H), 7.47 - 7.43 (m, 2H), 7.41 - 7.35 (m, 4H), 7.31 - 7.27 (m, 6H), 6.08 (d, *J* = 9.9 Hz, 1H), 5.96 (d, *J* = 9.9 Hz, 1H), 3.95 (s, 3H); ^{13}C NMR (126 MHz, $CDCl_3$) δ 155.05, 148.63, 145.66, 140.48, 137.93, 137.06, 129.84, 129.54, 128.64, 128.49, 128.29, 128.22, 127.76, 127.06, 122.83, 122.69, 122.57, 83.67, 62.83; HRMS calcd for $C_{23}H_{19}{}^{35}ClNO_2^+$ ($[M+H]^+$): 376.1099; found: 376.1106.

(4) (*Z*)-5-Bromo-3-(2,2-diphenylvinyl)isobenzofuran-1(3*H*)-one *O*-methyl oxime (5) (GFY-4-4, GFY-10-39).

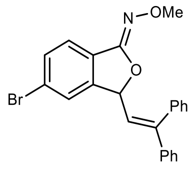

5

Following General Procedure I, after being stirred overnight, **5** was afforded as a white solid (0.2 mmol scale, 58.3 mg, 68%), eluent (0-15% of ethyl acetate in petroleum ether): m.p.= 59.3-61.7 °C (ethyl acetate/petroleum ether); ^{1}H NMR (500 MHz, $CDCl_3$) δ 7.53 (s, 2H), 7.46 - 7.43(m, 3H), 7.41 - 7.35 (m, 3H), 7.30 - 7.22 (m, 5H), 6.09 (d, *J* = 10.0 Hz, 1H), 5.96 (d, *J* = 9.9 Hz, 1H), 3.95 (s, 3H); ^{13}C NMR (126 MHz, $CDCl_3$) δ 155.08, 148.67, 145.87, 140.47, 137.92, 132.33, 129.83, 128.62, 128.47, 128.27,

128.20, 127.75, 127.58, 125.52, 125.16, 123.02, 122.67, 83.59, 62.80; HRMS calcd for $C_{23}H_{18}{}^{79}BrNNaO_2^+$ ([M+Na]$^+$): 442.0413; found: 442.0409.

Crystal data and structure refinement of 5:

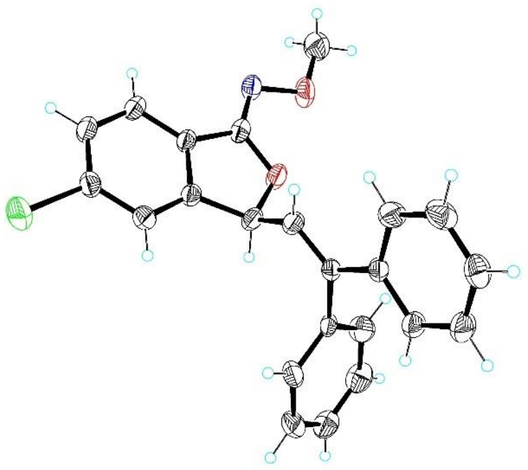

Bond precision:	C-C = 0.0055 A	Wavelength=1.54178	
Cell:	a=11.6213(7)	b=16.4338(11)	c=11.4359(8)
	alpha=90	beta=115.688(3)	gamma=90
Temperature:	300 K		
	Calculated	Reported	
Volume	1968.2(2)	1968.2(2)	
Space group	P 21/c	P 21/c	
Hall group	-P 2ybc	-P 2ybc	
Moiety formula	C23 H18 Br N O2	C23 H18 Br N O2	
Sum formula	C23 H18 Br N O2	C23 H18 Br N O2	
Mr	420.28	420.29	
Dx,g cm-3	1.418	1.418	
Z	4	4	
Mu (mm-1)	2.979	2.979	
F000	856.0	856.0	
F000’	855.37		
h,k,lmax	14,20,14	14,20,14	
Nref	3880	3829	

Tmin,Tmax	0.545,0.585	0.864,0.864
Tmin’	0.495	

Correction method= # Reported T Limits: Tmin=0.864 Tmax=0.864

AbsCorr = MULTI-SCAN

Data completeness= 0.987	Theta(max)= 72.271
R(reflections)= 0.0897(3292)	wR2(reflections)= 0.2464(3829)
S = 1.054	Npar= 244

(5) (*Z*)-3-(2,2-Diphenylvinyl)-5-(trifluoromethyl)isobenzofuran-1(3*H*)-one *O*-methyl oxime (6) (GFY-3-31, GFY-3-44).

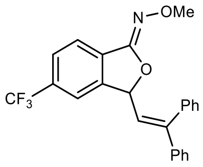

6

Following General Procedure I, after being stirred overnight, **6** was afforded as a white solid (0.2 mmol scale, 47.2 mg, 58%), eluent (0-7% of ethyl acetate in petroleum ether): m.p.= 49.7-50.5 °C (ethyl acetate/petroleum ether); ^{1}H NMR (500 MHz, $CDCl_3$) δ 7.78 (d, J = 8.1 Hz, 1H), 7.68 (d, *J* = 8.6 Hz, 1H), 7.52 (s, 1H), 7.46 (t, *J* = 7.1 Hz, 2H), 7.43 - 7.37 (m, 3H), 7.32 - 7.27 (m, 5H), 6.17 (d, *J* = 9.9 Hz, 1H), 5.98 (d, *J* = 9.9 Hz, 1H), 3.98 (s, 3H); ^{13}C NMR (126 MHz, $CDCl_3$) δ 154.65, 149.11, 144.44, 140.39, 137.85, 132.95 (q, *J* = 32.6 Hz), 132.06, 129.83, 128.66, 128.57, 128.30, 128.28, 127.78, 126.26, 123.60 (q, *J* = 273.3 Hz), 122.31, 122.65, 119.45 (q, *J* = 3.9 Hz). 84.10, 62.96; ^{19}F NMR (471 MHz, $CDCl_3$) δ -62.33; HRMS calcd for $C_{24}H_{19}F_3NO_2^+$ ($[M+H]^+$): 410.1362; found: 410.1372.

(6) (*Z*)-3-(2,2-Diphenylvinyl)-1-(methoxyimino)-1,3-dihydroisobenzofuran-5-carbonitrile (7) (GFY-7-44).

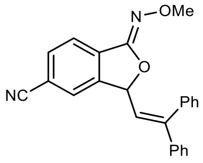

7

Following General Procedure I, after being stirred overnight, **7** was afforded as a white solid (0.2 mmol scale, 48.6 mg, 67%), eluent (0-17% of ethyl acetate in petroleum ether): m.p.= 175.3-176.1 °C (ethyl acetate/petroleum ether); ^{1}H NMR (500 MHz, $CDCl_3$) δ 7.78 (d, *J* = 8.3 Hz, 1H), 7.70 (d, *J* = 8.0 Hz, 1H), 7.60 (s, 1H), 7.51 - 7.45 (m, 2H), 7.44 - 7.42 (m, 1H), 7.40 - 7.36 (m, 2H), 7.32 - 7.29 (m, 3H), 7.28 - 7.25 (m, 2H), 6.16 (d, *J* = 9.9 Hz, 1H), 5.96 (d, *J* = 9.9 Hz, 1H), 3.99 (s, 3H); ^{13}C NMR (126 MHz, $CDCl_3$) δ 154.23, 149.36, 144.52, 140.22, 137.73, 132.85, 132.75, 129.72, 128.70, 128.64, 128.34, 128.31, 127.73, 126.30, 122.49, 121.92, 117.98, 114.32, 83.97, 63.04; HRMS calcd for $C_{24}H_{19}N_2O_2^+$ ($[M+H]^+$): 367.1441; found: 367.1447.

(7) Methyl (*Z*)-3-(2,2-diphenylvinyl)-1-(methoxyimino)-1,3-dihydroisobenzofuran -5-carboxylate (8) (GFY-7-34).

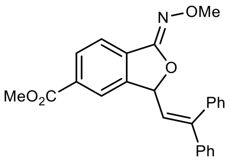

8

Following General Procedure I, after being stirred overnight, **8** was afforded as a white solid (0.2 mmol scale, 61.5 mg, 76%), eluent (0-15% of ethyl acetate in petroleum ether): m.p.= 62.4-62.7 °C (ethyl acetate/petroleum ether); ^{1}H NMR (500 MHz, $CDCl_3$) δ 8.10 (dd, *J* = 8.1, 1.4 Hz, 1H), 7.97 (s, 1H), 7.73 (d, *J* = 8.1 Hz, 1H), 7.49 - 7.43 (m, 2H), 7.43 - 7.37 (m, 3H), 7.28 (s, 5H), 6.16 (d, *J* = 10.0 Hz, 1H), 5.97 (d, *J* = 10.0 Hz, 1H), 3.98 (s, 3H), 3.95 (s, 3H); ^{13}C NMR (126 MHz, $CDCl_3$) δ 166.09, 155.08, 148.76, 144.14, 140.56, 137.96, 132.74, 132.56, 130.40, 129.91, 128.58, 128.44, 128.25, 128.18, 127.78, 123.59, 122.71, 121.62, 84.11, 62.92, 52.49; HRMS calcd for $C_{25}H_{22}NO_4^+$ ($[M+H]^+$): 400.1543; found: 400.1550.

(8) *(Z)*-3-(2,2-Diphenylvinyl)-5-nitroisobenzofuran-1(3*H*)-one *O*-methyl oxime (9) (GFY-12-25).

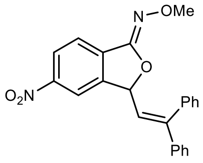

9

Following General Procedure I, after being stirred 2 h, **9** was afforded as an oil (0.2 mmol scale, 45.9 mg, 59%), eluent (0-7% of ethyl acetate in petroleum ether); ^{1}H NMR (500 MHz, $CDCl_3$)δ 8.31 (dd, *J* = 8.5, 2.0 Hz, 1H), 8.18 - 8.14 (m, 1H), 7.83 (d, *J* = 8.5 Hz, 1H), 7.53 – 7.47 (m, 2H), 7.46 – 7.42 (m, 3H), 7.34 - 7.27 (m, 5H), 6.22 (d, *J* = 9.9 Hz, 1H), 6.00 (d, *J* = 9.9 Hz, 1H), 4.01 (s, 3H); ^{13}C NMR (126 MHz, $CDCl_3$) δ 153.88, 149.71, 149.66, 145.09, 140.24, 137.74, 134.48, 129.79, 128.73, 128.69, 128.39, 128.33, 127.82, 127.78, 124.70, 122.54, 121.75, 118.02, 83.95, 63.14; HRMS calcd for $C_{25}H_{22}NO_4^+$ ($[M+H]^+$): 400.1543; found: 400.1550.

(9) (*Z*)-3-(2,2-Diphenylvinyl)-5-methylisobenzofuran-1(3*H*)-one *O*-methyl oxime (10) (GFY-4-5).

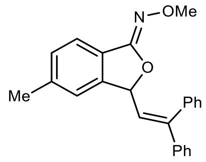

10

Following General Procedure I, after being stirred overnight, **10** was afforded as a white solid (0.2 mmol scale, 55.9 mg, 78%), eluent (0-6% of ethyl acetate in petroleum ether): m.p.= 44.5-46.5 °C (ethyl acetate/petroleum ether); ^{1}H NMR (500 MHz, $CDCl_3$) δ 7.57 (d, *J* = 7.9 Hz, 1H), 7.48 - 7.41 (m, 2H), 7.40 (dt, *J* = 7.8, 2.3 Hz, 3H), 7.28 (s, 5H), 7.23 - 7.20 (m, 1H), 7.10 (s, 1H), 6.08 (d, *J* = 9.9 Hz, 1H), 5.98 (d, *J* = 9.9 Hz, 1H), 3.96 (s, 3H), 2.43 (s, 3H); ^{13}C NMR (126 MHz, $CDCl_3$) δ 156.18, 147.69, 144.41, 141.51, 140.75, 138.16, 129.98, 129.91, 128.49, 128.23, 128.19, 128.01, 127.71, 125.74, 123.70, 122.47, 121.43, 83.88, 62.60, 21.71; HRMS calcd for $C_{24}H_{22}NO_2^+$ ($[M+H]^+$): 356.1645; found: 356.1654.

(10) (*Z*)-3-(2,2-Diphenylvinyl)-5-ethylisobenzofuran-1(3*H*)-one *O*-methyl oxime (11) (GFY-4-7).

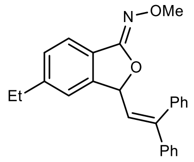

11

Following General Procedure I, after being stirred overnight, **11** was afforded as an oil (0.2 mmol scale, 65.5 mg, 85%), eluent (0-3% of ethyl acetate in petroleum ether):^{1}H NMR (500 MHz, $CDCl_3$) δ 7.61 (d, *J* = 7.9 Hz, 1H), 7.50 - 7.43 (m, 2H), 7.44 - 7.37 (m, 3H), 7.29 (d, *J* = 10.2 Hz, 6H), 7.12 (d, *J* = 1.4 Hz, 1H), 6.11 (d, *J* = 9.9 Hz, 1H), 6.01 (d, *J* = 9.9 Hz, 1H), 3.97 (s, 3H), 2.74 (q, *J* = 7.6 Hz, 2H), 1.28 (t, *J* = 7.6 Hz, 3H); ^{13}C NMR (126 MHz, $CDCl_3$) δ 156.24, 147.94, 147.71, 144.46, 140.79, 138.18, 129.95, 128.91, 128.52, 128.26, 128.22, 128.04, 127.75, 125.94, 123.74, 121.56, 121.29, 83.97, 62.65, 29.08, 15.49; HRMS calcd for $C_{25}H_{24}NO_2^+$ ([M+H]$^+$): 370.1802; found: 370.1810.

(11) (*Z*)-5-Butyl-3-(2,2-diphenylvinyl)isobenzofuran-1(3*H*)-one *O*-methyl oxime (12) (GFY-4-12).

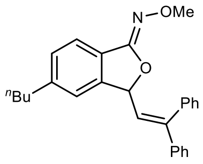

12

Following General Procedure I, after being stirred overnight, **12** was afforded as an oil (0.2 mmol scale, 62.3 mg, 78%), eluent (0-7% of ethyl acetate in petroleum ether): ^{1}H NMR (500 MHz, $CDCl_3$) δ 7.59 (d, *J* = 7.9 Hz, 1H), 7.49 - 7.43 (m, 2H), 7.42 - 7.37 (m, 3H), 7.28 (s, 5H), 7.23 (dd, *J* = 7.9, 1.5 Hz, 1H), 7.09 (s, 1H), 6.09 (d, *J* = 9.9 Hz, 1H), 6.00 (d, *J* = 10.0 Hz, 1H), 3.96 (s, 3H), 2.72 - 2.65 (m, 2H), 1.67 - 1.57 (m, 2H), 1.43 - 1.34 (m, 2H), 0.95 (t, *J* = 7.3 Hz, 3H); ^{13}C NMR (126 MHz, $CDCl_3$) δ 156.21, 147.70, 146.64, 144.38, 140.79, 138.17, 129.94, 129.37, 128.49, 128.23, 128.19, 128.01, 127.74, 125.91, 123.73, 121.76, 121.44, 83.93, 62.61, 35.84, 33.52, 22.34, 13.86; HRMS calcd for $C_{27}H_{28}NO_2^+$ ([M+H]$^+$): 398.2115; found: 398.2120.

(12) (*Z*)-5-(*tert*-Butyl)-3-(2,2-diphenylvinyl)isobenzofuran-1(3*H*)-one *O*-methyl oxime (13) (GFY-4-14).

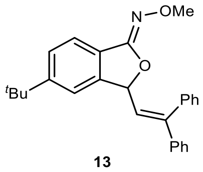

13

Following General Procedure I, after being stirred overnight, **13** was afforded as a white solid (0.2 mmol scale, 54.5 mg, 68%), eluent (0-15% of ethyl acetate in petroleum ether): m.p.= 46.2-48.2 °C; ^{1}H NMR (500 MHz, $CDCl_3$) δ 7.61 (d, *J* = 8.2 Hz, 1H), 7.49 - 7.42 (m, 3H), 7.44 - 7.36 (m, 3H), 7.40 - 7.25 (m, 6H), 6.10 (d, *J* = 9.9 Hz, 1H), 6.02 (d, *J* = 9.9 Hz, 1H), 3.96 (s, 3H), 1.35 (s, 9H); ^{13}C NMR (126 MHz, $CDCl_3$) δ 156.22, 154.93, 147.59, 144.20, 140.76, 138.24, 129.94, 128.54, 128.25, 128.22, 128.03, 126.58, 125.63, 123.88, 121.25, 118.50, 84.16, 62.64, 35.21, 31.30; HRMS calcd for $C_{27}H_{28}NO_2^+$ ([M+H]$^+$): 398.2115; found: 398.2120.

(13) (*Z*)-3-(2,2-Diphenylvinyl)-5-methoxyisobenzofuran-1(3*H*)-one *O*-methyl oxime (14) (GFY-4-6, GFY-8-15).

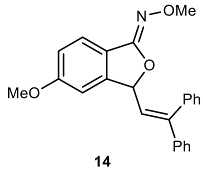

14

Following General Procedure I, after being stirred for 2 h, **14** was afforded as a white solid (0.2 mmol scale, 50.3 mg, 67%), eluent (0-14% of ethyl acetate in petroleum ether): m.p.= 51.8-62.9 °C (ethyl acetate/petroleum ether); ^{1}H NMR (500 MHz, $CDCl_3$) δ 7.59 (d, *J* = 8.5 Hz, 1H), 7.47 - 7.41 (m, 2H), 7.41 - 7.36 (m, 3H), 7.29 - 7.26 (m, 5H), 6.94 (dd, *J* = 8.5, 2.3 Hz, 1H), 6.75 (d, *J* = 2.2 Hz, 1H), 6.06 (d, *J* = 9.9 Hz, 1H), 5.99 (d, *J* = 9.9 Hz, 1H), 3.94 (s, 3H), 3.85 (s, 3H); ^{13}C NMR (126 MHz, $CDCl_3$) δ 162.29, 156.04, 147.85, 146.21, 140.71, 138.15, 129.91, 128.56, 128.31, 128.24, 128.08, 127.74, 123.60, 122.96, 120.64, 115.71, 106.80, 83.67, 62.61, 55.68; HRMS calcd for $C_{24}H_{22}NO_3^+$ ([M+H]$^+$): 372.1594; found: 372.1604.

(14) (*Z*)-3-(2,2-Diphenylvinyl)-5-vinylisobenzofuran-1(3*H*)-one *O*-methyl oxime (15) (GFY-3-43, GFY-3-30, GFY-10-42).

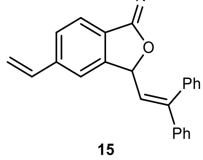

15

Following General Procedure I, after being stirred overnight, **15** was afforded as a white solid (0.2 mmol scale, 46.2 mg, 63%), eluent (0-6% of ethyl acetate in petroleum ether): m.p.= 59.2-61.9 °C (ethyl acetate/petroleum ether); ^{1}H NMR (500 MHz, $CDCl_3$) δ 7.63 (d, *J* = 8.0 Hz, 1H), 7.49 - 7.43 (m, 3H), 7.40 (d, *J* = 7.1 Hz, 3H), 7.29 - 7.25 (m, 6H), 6.75 (dd, *J* = 17.6, 10.9 Hz, 1H), 6.11 (d, *J* = 9.9 Hz, 1H), 5.99 (d, *J* = 9.9 Hz, 1H), 5.83 (d, *J* = 17.5 Hz, 1H), 5.37 (d, *J* = 10.9 Hz, 1H), 3.96 (s, 3H); ^{13}C NMR (126 MHz, $CDCl_3$) δ 155.90, 148.02, 144.63, 140.70, 140.55, 138.12, 135.92, 129.94, 128.56, 128.34, 128.25, 128.21, 128.11, 127.77, 127.12, 123.45, 121.80, 119.71, 116.09, 83.97, 62.75; HRMS calcd for $C_{25}H_{21}NNaO_2^+$ ([M+Na]$^+$): 390.1465; found: 390.1457.

(15) (*Z*)-5-(Chloromethyl)-3-(2,2-diphenylvinyl)isobenzofuran-1(3*H*)-one *O*-methyl oxime (16) (GFY-7-35).

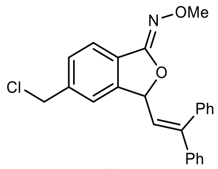

16

Following General Procedure I, after being stirred overnight, **16** was afforded as a white solid (0.2 mmol scale, 67.8 mg, 87%), eluent (0-10% of ethyl acetate in petroleum ether): m.p.= 55.8-56.3 °C (ethyl acetate/petroleum ether); ^{1}H NMR (500 MHz, $CDCl_3$) δ 7.67 (d, *J* = 8.0 Hz, 1H), 7.50 - 7.42 (m, 3H), 7.41 - 7.38 (m, 3H), 7.33 - 7.25 (m, 6H), 6.12 (d, *J* = 9.9 Hz, 1H), 5.98 (d, *J* = 10.0 Hz, 1H), 4.63 (s, 2H), 3.97 (s, 3H); ^{13}C NMR (126 MHz, $CDCl_3$) δ 155.47, 148.32, 144.63, 140.58, 140.55, 138.01, 129.87, 129.51, 128.64, 128.57, 128.38, 128.24, 128.13, 127.74, 123.08, 122.21, 122.04, 83.96, 62.76, 45.50; HRMS calcd for $C_{24}H_{20}{}^{35}ClNNaO_2^+$ ([M+Na]$^+$): 412.1075; found: 412.1082.

(16) (*Z*)-3-(2,2-Diphenylvinyl)-5-phenylisobenzofuran-1(3*H*)-one *O*-methyl oxime (17) (GFY-4-13, GFY-9-37).

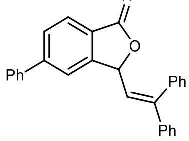

17

Following General Procedure I, after being stirred for 2 h, **17** was afforded as a white solid (0.2 mmol scale, 51.7 mg, 62%), eluent (0-9% of ethyl acetate in petroleum ether): m.p.= 56.4-59.2 °C (ethyl acetate/petroleum ether); ^{1}H NMR (500 MHz, $CDCl_3$) δ 7.74 (d, *J* = 8.0 Hz, 1H), 7.64 (dd, *J* = 8.1, 1.5 Hz, 1H), 7.61 - 7.57 (m, 2H), 7.50 - 7.45 (m, 4H), 7.44 (s, 1H), 7.43 - 7.38 (m, 4H), 7.28 (s, 5H), 6.18 (d, *J* = 10.0 Hz, 1H), 6.05 (d, *J* = 10.0 Hz, 1H), 3.99 (s, 3H); ^{13}C NMR (126 MHz, $CDCl_3$) δ 155.93, 148.11, 144.86, 144.40, 140.71, 140.15, 138.15, 129.96, 128.97, 128.57, 128.34, 128.30, 128.24, 128.11, 128.08, 127.79, 127.37, 127.33, 123.49, 122.02, 120.70, 84.13, 62.77; HRMS calcd for $C_{29}H_{23}NNaO_2^+$ ([M+Na]$^+$): 440.1621; found: 440.1630.

(17) (*Z*)-4,6-Dichloro-3-(2,2-diphenylvinyl)isobenzofuran-1(3*H*)-one *O*-methyl oxime (18) (GFY-4-27, GFY-11-46).

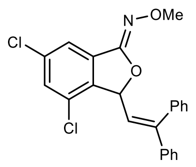

18

Following General Procedure I, after being stirred overnight, **18** was afforded as a white solid (0.2 mmol scale, 56.0 mg, 68%), eluent (0-4% of ethyl acetate in petroleum ether): m.p.= 143.7-146.3 °C (ethyl acetate/petroleum ether); ^{1}H NMR (500 MHz, $CDCl_3$) δ 7.56 (d, *J* = 1.7 Hz, 1H), 7.45 - 7.42 (m, 4H), 7.41 - 7.34 (m, 2H), 7.29 - 7.27 (m, 2H), 7.25 - 7.23 (m, 2H), 6.09 (d, *J* = 10.2 Hz, 1H), 5.84 (d, *J* = 10.4 Hz, 1H), 3.95 (s, 3H); ^{13}C NMR (126 MHz, $CDCl_3$) δ 153.98, 150.30, 141.15, 139.72, 138.20, 136.07, 132.18, 131.10, 129.94, 129.67, 128.48, 128.46, 128.22, 128.14, 127.92, 120.87, 120.27, 84.06, 63.01; HRMS calcd for $C_{23}H_{18}{}^{35}Cl_2NO_2^+$ ([M+H]$^+$): 410.0709; found: 410.0711.

(18) (*Z*)-3-(2,2-Diphenylvinyl)-5-(methylthio)isobenzofuran-1(3*H*)-one *O*-methyl oxime (19) (GFY-4-34, GFY-9-1).

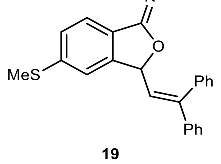

19

Following General Procedure I, after being stirred for 2 h, **19** was afforded as an oil (0.2 mmol scale, 34.8 mg, 44%), eluent (0-18% of ethyl acetate in petroleum ether):^{1}H NMR (500 MHz, $CDCl_3$) δ 7.59 (d, *J* = 8.1 Hz, 1H), 7.50 - 7.43 (m, 2H), 7.43 - 7.38 (m, 3H), 7.31 - 7.24 (m, 6H), 7.12 (s, 1H), 6.10 (d, *J* = 9.9 Hz, 1H), 6.01 (d, *J* = 9.9 Hz, 1H), 3.97 (s, 3H), 2.54 (s, 3H); ^{13}C NMR (126 MHz, $CDCl_3$) δ 155.78, 148.03, 144.85, 143.03, 140.61, 138.09, 129.88, 128.57, 128.34, 128.23, 128.10, 127.72, 126.54, 125.12, 123.34, 121.84, 119.02, 83.76, 62.68, 15.46; HRMS calcd for $C_{24}H_{21}NNaO_2S^+$ ([M+Na]$^+$): 410.1185; found: 410.1194.

(19) (*Z*)-3-(2,2-Diphenylvinyl)naphtho[2,3-*c*]furan-1(3*H*)-one *O*-methyl oxime (20) (GFY-4-37).

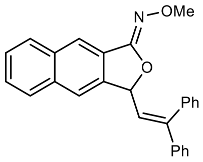

20

Following General Procedure I, after being stirred overnight, **20** was afforded as a white solid (0.2 mmol scale, 57.2 mg, 73%), eluent (0-18% of ethyl acetate in petroleum ether): m.p.= 142.4-144.1 °C (ethyl acetate/petroleum ether); ^1H NMR (500 MHz, $CDCl_3$) δ 8.21 (s, 1H), 7.91 (d, *J* = 7.9 Hz, 1H), 7.86 (d, *J* = 7.0 Hz, 1H), 7.72 (s, 1H), 7.57 – 7.49 (m, 2H), 7.47 (d, *J* = 4.4 Hz, 4H), 7.43 - 7.38 (m, 1H), 7.31 - 7.26 (m, 5H), 6.26 (d, *J* = 10.0 Hz, 1H), 6.07 (d, *J* = 9.9 Hz, 1H), 4.01 (s, 3H); ^{13}C NMR (126 MHz, $CDCl_3$) δ 155.58, 147.82, 140.78, 140.36, 138.18, 134.69, 133.40, 129.99, 128.92, 128.55, 128.29, 128.23, 128.11, 128.07, 127.78, 127.44, 126.72, 126.50, 123.85, 121.26, 121.03, 83.73, 62.81; HRMS calcd for $C_{27}H_{22}NO_2^+$ ([M+H]$^+$): 392.1645; found: 392.1651.

(20) (*Z*)-3-(2,2-Diphenylvinyl)-6-methylisobenzofuran-1(3*H*)-one *O*-methyl oxime (21) (GFY-4-29).

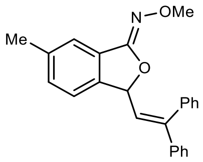

21

Following General Procedure I, after being stirred overnight, **21** was afforded as a white solid (0.2 mmol scale, 63.2 mg, 88%), eluent (0-15% of ethyl acetate in petroleum ether): m.p.= 123.2-125.6 °C (ethyl acetate/petroleum ether); ^{1}H NMR (500 MHz, $CDCl_3$) δ 7.51 (s, 1H), 7.46 - 7.40 (m, 2H), 7.39 (d, *J* = 7.1 Hz, 3H), 7.29 (s, 5H), 7.20 (d, *J* = 7.8 Hz, 1H), 6.09 (d, *J* = 9.9 Hz, 1H), 5.97 (d, *J* = 9.9 Hz, 1H), 3.97 (s, 3H), 2.41 (s, 3H); ^{13}C NMR (126 MHz, $CDCl_3$) δ 156.10, 147.55, 141.35, 140.76, 139.04, 138.18, 131.98, 129.89, 128.53, 128.47, 128.19, 128.18, 127.99, 127.69, 123.83, 121.83, 121.80, 84.07, 62.65, 21.16; HRMS calcd for $C_{24}H_{22}NO_2^+$ ($[M+H]^+$): 356.1645; found: 356.1655.

(21) (*Z*)-3-(2,2-Diphenylvinyl)isobenzofuran-1(3*H*)-one *O*-ethyl oxime (22) (GFY-7-40).

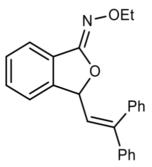

22

Following General Procedure I, after being stirred overnight, **22** was afforded as an oil (0.2 mmol scale, 48.3 mg, 68%), eluent (0-5% of ethyl acetate in petroleum ether); ^{1}H NMR (500 MHz, $CDCl_3$) δ 7.69 (d, *J* = 7.6 Hz, 1H), 7.48 - 7.42 (m, 3H), 7.42 - 7.37 (m, 4H), 7.34 - 7.30 (m, 1H), 7.28 (s, 5H), 6.14 (d, *J* = 9.9 Hz, 1H), 6.00 (d, *J* = 9.9 Hz, 1H), 4.25 - 4.16 (m, 2H), 1.39 (t, *J* = 7.1 Hz, 6H); ^{13}C NMR (126 MHz, $CDCl_3$) δ 155.72, 147.38, 143.89, 140.75, 138.17, 130.72, 129.88, 128.81, 128.66, 128.49, 128.21, 128.19, 128.01, 127.69, 123.85, 122.13, 121.66, 84.02, 70.34, 14.58; HRMS calcd for $C_{24}H_{22}NO_2^+$ ($[M+H]^+$): 356.1645; found: 356.1655.

(22) (*Z*)-3-(2,2-Diphenylvinyl)isobenzofuran-1(3*H*)-one *O*-isopropyl oxime (23) (GFY-11-26).

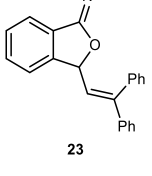

23

Following General Procedure I, after being stirred for 2h, **23** was afforded as an oil (0.2 mmol scale, 34.3 mg, 45%), eluent (0-7% of ethyl acetate in petroleum ether); ^{1}H NMR (500 MHz, $CDCl_3$) δ 7.68 (dd, *J* = 7.6, 1.1 Hz, 1H), 7.47 - 7.41 (m, 3H), 7.40 - 7.35 (m, 4H), 7.31 (dd, *J* = 7.6, 1.0 Hz, 1H), 7.27 (s, 5H), 6.13 (d, *J* = 9.9 Hz, 1H), 6.00 (d, *J* = 9.8 Hz, 1H), 4.39 - 4.30 (m, *J* = 6.2 Hz, 1H), 1.37 - 1.34 (m, *J* = 8.7, 6H); ^{13}C NMR (126 MHz, $CDCl_3$) δ 155.22, 146.95, 143.82, 140.86, 138.25, 130.57, 129.92, 129.02, 128.76, 128.50, 128.21, 128.18, 128.00, 127.72, 124.23, 122.13, 121.65, 83.92, 76.17, 21.57, 21.54; HRMS calcd for $C_{25}H_{23}NNaO_2^+$ ($[M+Na]^+$): 392.1621; found: 392.1621.

(23) (*Z*)-3-(2,2-Diphenylvinyl)-4-methoxyisobenzofuran-1(3*H*)-one *O*-methyl oxime (24) and (*Z*)-3-(2,2-diphenylvinyl)-6-methoxyisobenzofuran-1(3*H*)-one *O*-methyl oxime (24') (GFY-4-50).

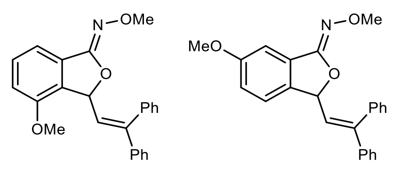

24 + **24'** (1.5:1)

Following General Procedure I, after being stirred overnight, a mixture of **24** and **24'** was afforded as a white solid (0.2 mmol scale, 55.7 mg, 73%; **24**:**24'** = 1.5: 1), eluent (0-20% of ethyl acetate in petroleum ether): ^{1}H NMR (500 MHz, $CDCl_3$) δ 7.46 - 7.44 (m, 2H), 7.43 - 7.40 (m, 2H), 7.43 - 7.36 (m, 4.34H), 7.39 - 7.33 (m, 3H), 7.29 - 7.23 (m, 7H), 7.19 (d, *J* = 8.4 Hz, 1H), 7.13 - 7.11 (m, 0.67H), 7.03 (dd, *J* = 8.4, 2.4 Hz, 0.67H), 6.90 (d, *J* = 8.0 Hz, 1H), 6.17 (d, *J* = 10.1 Hz, 1H), 6.06 (d, *J* = 9.8 Hz, 0.67H), 5.95 (d, *J* = 9.7 Hz, 0.67H), 5.89 (d, *J* = 10.0 Hz, 1H), 3.96 (s, 2H), 3.95 (s, 3H), 3.84 (s, 5H);; ^{13}C NMR (126 MHz, $CDCl_3$) δ 160.55, 156.30, 154.60, 147.55, 147.36, 141.43, 140.79, 138.63, 138.21, 136.41, 131.70, 130.83, 130.36, 130.20, 129.91, 129.72, 128.52, 128.24, 128.22, 128.15, 128.11, 128.02, 128.00, 127.86, 127.72, 123.94, 123.04, 122.95, 119.76, 113.61, 112.19, 104.14, 83.98, 83.24, 62.70, 55.72, 55.41; HRMS calcd for $C_{24}H_{22}NO_3^+$ ($[M+H]^+$): 372.1594; found: 372.1591.

(24) (*Z*)-8-(2,2-Diphenylvinyl)-[1,3]dioxolo[4,5-*e*]isobenzofuran-6(8*H*)-one *O*-methyl oxime (25) and (*Z*)-7-(2,2-diphenylvinyl)-[1,3]dioxolo[4,5-*f*]isobenzofuran-5(7*H*)-one *O*-methyl oxime (25’) (GFY-4-21).

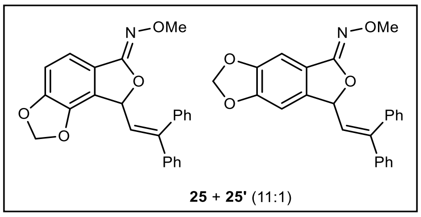

Following General Procedure I, after being stirred overnight, a mixture of **25** and **25’** was afforded as a white solid (0.2 mmol scale, 38.8 mg, 50%; **25**:**25’** = 11: 1), eluent (0-9% of ethyl acetate in petroleum ether): ^{1}H NMR (500 MHz, $CDCl_3$) δ 7.44 - 7.40 (m, 4.36H), 7.39 - 7.36 (m, 1.08H), 7.28 (s, 5.45H), 7.22 (d, *J* = 8.1 Hz, 1.08H), 7.03 (s, 0.08H), 6.88 (d, *J* = 8.1 Hz, 1H), 6.67 (s, 0.08H), 6.12 (d, *J* = 10.2 Hz, 1H), 6.10 (d, *J* = 1.4 Hz, 1H), 6.07 (d, *J* = 1.4 Hz, 1H), 6.05 (d, *J* = 1.2 Hz, 0.09H), 6.04 (d, *J* = 1.3 Hz, 0.09H), 5.96 (d, *J* = 10.1 Hz, 1.18H), 3.93 (s, 3.27H); ^{13}C NMR (126 MHz, $CDCl_3$) δ 155.51, 150.18, 148.11, 141.56, 140.94, 138.21, 130.22, 128.29, 128.26, 128.20, 127.98, 127.94, 124.33, 123.59, 122.12, 115.54, 109.74, 102.25, 81.26, 62.66; HRMS calcd for $C_{24}H_{20}NO_4^+$ ($[M+H]^+$): 386.1387; found: 386.1394.

(25) (*Z*)-3-(2,2-Diphenylvinyl)isobenzofuran-1(3*H*)-one *O*-ethyl oxime (26) and (*Z*)-3-(2,2-diphenyl vinyl)-4-fluoro-5-methylisobenzofuran-1(3*H*)-one *O*-methyl oxime (26’) (GFY-4-35, GFY-7-15).

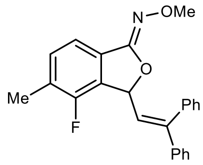

26

Following General Procedure I on 0.2 mmol scale, after being stirred overnight, **26** (44.1 mg, 59%) and **26’** (6.4 mg, 9%,) were afforded via flash chromatography on silica gel (0-6% of ethyl acetate in petroleum ether):

26: more polar product; white solid; m.p.= 149.6-150.1 ^{o}C (ethyl acetate/petroleum ether); ^{1}H NMR (500 MHz, $CDCl_3$) δ 7.43 (d, *J* = 4.4 Hz, 4H), 7.40 - 7.34 (m, 2H), 7.28 (s, 5H), 7.24 (dd, *J* = 12.3, 5.0 Hz, 1H), 6.19 (s, 1H), 5.96 (d, *J* =

10.1 Hz, 1H), 3.95 (s, 3H), 2.33 (s, 3H); ^{13}C NMR (126 MHz, $CDCl_3$) δ 155.53 (d, *J* = 249.2 Hz), 155.29, 148.31, 140.92, 138.23, 133.05 (d, *J* = 4.0 Hz), 130.11 (d, *J* = 18.3z Hz), 129.99 (d, *J* = 2.0 Hz), 129.23 (d, *J* = 4.5 Hz), 128.36, 128.25, 128.18, 127.94, 127.833 (d, *J* = 15.9 Hz), 127.827, 122.07, 117.01 (d, *J* = 4.4 Hz), 82.09 (d, *J* = 2.1 Hz), 62.75, 14.44 (d, *J* = 3.4 Hz); ^{19}F NMR (471 MHz, $CDCl_3$) δ -122.71; HRMS calcd for $C_{24}H_{21}FNO_2^+$ ([M+H]$^+$): 374.1551; found: 374.1558.

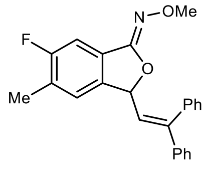

26'

26':less polar product; oil; ^{1}H NMR (500 MHz, $CDCl_3$) δ 7.44 (dd, *J* = 7.8, 6.5 Hz, 2H), 7.39 - 7.35 (m, 3H), 7.31 - 7.25 (m, 6H), 7.08 (d, *J* = 6.4 Hz, 1H), 6.05 (d, *J* = 9.9 Hz, 1H), 5.95 (d, *J* = 10.0 Hz, 1H), 3.94 (s, 3H), 2.33 (d, *J* = 2.2 Hz, 3H); ^{13}C NMR (126 MHz, $CDCl_3$) δ 161.78 (d, *J* = 247.0 Hz), 155.37, 148.01, 140.69, 139.43 (d, *J* = 2.6z Hz), 138.11, 129.90, 129.09 (d, *J* = 19.5 Hz), 128.57, 128.37, 128.27, 128.13, 127.75, 124.69 (d, *J* = 5.8 Hz), 123.49, 107.91 (d, *J* = 26.2f Hz), 83.82, 62.76, 29.69, 15.25 (d, *J* = 4.0 Hz); ^{19}F NMR (471 MHz, $CDCl_3$) δ -115.92; HRMS calcd for $C_{24}H_{21}FNO_2^+$ ([M+H]$^+$): 374.1551; found: 374.1557.

(26) (*Z*)-3-(2,2-Di-*p*-tolylvinyl)isobenzofuran-1(3*H*)-one *O*-methyl oxime (27) (GFY-5-18).

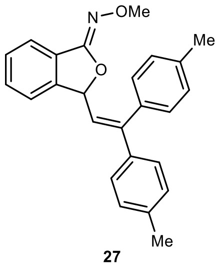

27

Following General Procedure I, after being stirred overnight, **27** was afforded as a white solid (0.2 mmol scale, 54.0 mg, 72%), eluent (0-9% of ethyl acetate in petroleum ether): m.p.= 130.1-132.2 °C (ethyl acetate/petroleum ether); ^{1}H NMR (500 MHz, $CDCl_3$) δ 7.69 (d, *J* = 7.7 Hz, 1H), 7.50 - 7.40 (m, 1H), 7.39 (t, *J* = 7.5 Hz, 1H), 7.32 -

7.26 (m, 3H), 7.24 (d, J = 7.9 Hz, 2H), 7.17 (d, J = 8.2 Hz, 2H), 7.08 (d, J = 8.0 Hz, 2H), 6.14 (d, J = 9.9 Hz, 1H), 5.93 (d, J = 9.9 Hz, 1H), 3.97 (s, 3H), 2.40 (s, 3H), 2.33 (s, 3H); ^{13}C NMR (126 MHz, $CDCl_3$) δ 156.13, 147.77, 144.20, 138.15, 138.12, 137.74, 135.34, 130.83, 129.80, 129.80, 129.66, 129.35, 129.13, 128.99, 128.87, 128.78, 127.65, 127.34, 122.43, 122.18, 121.62, 84.42, 62.65, 21.20, 21.08; HRMS calcd for $C_{25}H_{24}NO_2^+$ ([M+H]$^+$): 370.1802; found: 370.1810.

(27) (*Z*)-3-(2,2-Bis(4-methoxyphenyl)vinyl)isobenzofuran-1(3*H*)-one *O*-methyl oxime (28) (GFY-10-3, GFY-10-20).

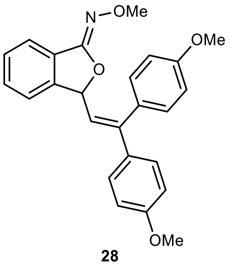

28

Following General Procedure I, after being stirred for 2 h, **28** was afforded as an oil (0.2 mmol scale, 58.2 mg, 72%), eluent (0-15% of ethyl acetate in petroleum ether): ^{1}H NMR (500 MHz, $CDCl_3$) δ 7.68 (d, J = 7.6 Hz, 1H), 7.49 - 7.43 (m, 1H), 7.40 (t, J = 7.5 Hz, 1H), 7.34 - 7.28 (m, 3H), 7.28 - 7.17 (m, 2H), 6.96 (dd, J = 8.6, 1.7 Hz, 2H), 6.83 - 6.77 (m, 2H), 6.13 (d, J = 9.9 Hz, 1H), 5.84 (d, J = 9.9 Hz, 1H), 3.96 (s, 3H), 3.84 (s, 3H), 3.79 (s, 3H); ^{13}C NMR (126 MHz, $CDCl_3$) δ 155.76, 145.66, 143.50, 138.81, 136.08, 134.55, 134.49, 131.22, 130.99, 129.10, 128.98, 128.95, 128.55, 128.42, 124.54, 121.98, 121.84, 83.68, 77.25, 62.75; HRMS calcd for $C_{25}H_{23}NNaO_4^+$ ([M+Na]$^+$): 424.1519; found: 424.1519.

(28) (*Z*)-3-(2,2-Bis(4-chlorophenyl)vinyl)isobenzofuran-1(3*H*)-one *O*-methyl oxime (29) (GFY-10-11).

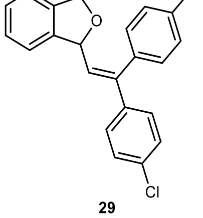

29

Following General Procedure I, after being stirred for 2 h, **29** was afforded as an oil (0.2 mmol scale, 32.2 mg, 40%), eluent (0-7% of ethyl acetate in petroleum ether): ^{1}H NMR (500 MHz, $CDCl_3$) δ 7.71 (d, *J* = 7.8 Hz, 1H), 7.50 (td, *J* = 7.5, 1.2 Hz, 1H), 7.45 (d, *J* = 8.3 Hz, 3H), 7.40 - 7.31 (m, 2H), 7.31 - 7.25 (m, 3H), 7.22 - 7.15 (m, 2H), 6.07 (d, *J* = 9.9 Hz, 1H), 5.99 (d, *J* = 9.9 Hz, 1H), 3.98 (s, 3H); ^{13}C NMR (126 MHz, $CDCl_3$) δ 155.76, 145.66, 143.50, 138.81, 136.08, 134.55, 134.49, 131.22, 130.99, 129.10, 128.98, 128.95, 128.55, 128.42, 124.54, 121.98, 121.84, 83.68, 77.25, 62.75; HRMS calcd for $C_{23}H_{17}{}^{35}Cl_2NNaO_2^+$ ($[M+Na]^+$): 432.0529; found: 432.0526.

(29) 3-(2-(4-Bromophenyl)-2-phenylvinyl)isobenzofuran-1(3*H*)-one *O*-methyl oxime (30) (GFY-5-38).

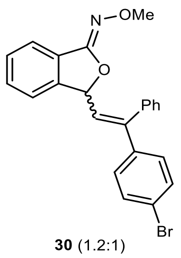

30 (1.2:1)

Following General Procedure I, after being stirred overnight, **30** was afforded as an oil (0.2 mmol scale, 62.5 mg, 74%, stereoisomers ratio = 1.2:1), eluent (0-9% of ethyl acetate in petroleum ether): δ 7.70 - 7.66 (m, 1.11H), 7.58 (d, *J* = 8.1 Hz, 1.11H), 7.49 - 7.46 (m, 1.11H), 7.44 - 7.41 (m, 2.22H), 7.40 - 7.35 (m, 2.22H), 7.31 - 7.25 (m, 4.44H), 7.24 - 7.22 (m, 1.11H), 7.13 (d, *J* = 8.3 Hz, 1.11H), 6.11 - 6.02 (m, 1.11H), 6.00 - 5.96 (m, 1.11H), 3.96 (s, 3.33H); ^{13}C NMR (126 MHz, $CDCl_3$) δ 155.93, 155.89, 146.81, 146.75, 143.75, 143.72, 140.26, 139.69, 137.61, 137.08, 131.81, 131.61, 131.41, 130.96, 129.84, 129.30, 129.03, 129.01, 128.70, 128.55, 128.43, 128.35, 128.33, 127.71, 124.09, 122.55, 122.39, 122.12, 122.04, 121.80, 121.78, 83.89, 62.76, 29.04, 11.41; HRMS calcd for $C_{23}H_{18}{}^{79}BrNNaO_2^+$ ($[M+Na]^+$): 442.0413; found: 442.0422.

(30) 3-(2-Phenyl-2-(*p*-tolyl)vinyl)isobenzofuran-1(3*H*)-one *O*-methyl oxime (31) (GFY-9-40).

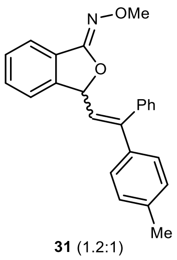

31 (1.2:1)

Following General Procedure I, after being stirred for 2 h, **31** was afforded as an oil (0.2 mmol scale, 64.2 mg, 91%, stereoisomers ratio = 1.2:1), eluent (0-9% of ethyl acetate in petroleum ether): ^{1}H NMR (500 MHz, $CDCl_3$) δ 7.71 (d, *J* = 7.6 Hz, 1.84H), 7.52 - 7.43 (m, 5.52H), 7.48 - 7.37 (m, 3.68H), 7.34 (d, *J* = 7.7 Hz, 2H), 7.30 (d, *J* = 4.6 Hz, 5.52H), 7.27 (d, *J* = 8.0 Hz, 2.68H), 7.19 (d, *J* = 8.3 Hz, 2H), 7.11 (d, *J* = 8.1 Hz, 2.52H), 6.18 (d, *J* = 9.9 Hz, 0.84H), 6.13 (d, *J* = 10.0 Hz, 1H), 5.99 (d, *J* = 2.7 Hz, 1H), 5.97 (d, *J* = 2.6 Hz, 0.84H), 4.00 (s, 5.52H), 2.42 (s, 2.52H), 2.35 (s, 3H); ^{13}C NMR (126 MHz, $CDCl_3$) δ 156.08, 147.87, 147.72, 144.10, 140.96, 138.32, 138.24, 137.87, 137.85, 135.18, 130.85, 129.88, 129.80, 129.18, 128.92, 128.83, 128.47, 128.40, 128.19, 128.16, 127.96, 127.75, 127.60, 123.36, 122.64, 122.16, 121.65, 84.29, 84.27, 62.67, 21.20, 21.08; HRMS calcd for $C_{24}H_{21}NNaO_2^+$ ($[M+Na]^+$): 378.1465; found: 378.1469.

(31) 3-(2-(Naphthalen-2-yl)-2-phenylvinyl)isobenzofuran-1(3*H*)-one *O*-methyl oxime (32) (GFY-10-21).

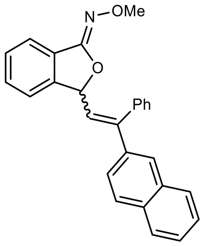

32 (1:1)

Following General Procedure I, after being stirred for 2 h, **32** was afforded as a white solid (0.2 mmol scale, 59.7 mg, 78%, stereoisomers ratio = 1:1), eluent (0-7% of ethyl acetate in petroleum ether): ^{1}H NMR (500 MHz, $CDCl_3$) δ 7.93 (s, 1H), 7.90 - 7.86 (m, 3H), 7.80 - 7.76 (m, 1H), 7.74 (d, *J* = 8.7 Hz, 1H), 7.72 - 7.67 (m, 3H), 7.62 (s, 1H), 7.55 - 7.50 (m, 2H), 7.49 - 7.44 (m, 5H), 7.44 - 7.38 (m, 8H), 7.35 (d, *J* = 7.7 Hz, 2H), 7.31 - 7.24 (m, 5H), 6.19 (d, *J* = 5.5 Hz, 1H), 6.17 (d, *J* = 5.8 Hz, 1H), 6.13

(d, *J* = 9.9 Hz, 1H), 6.07 (d, *J* = 9.9 Hz, 1H), 3.98 (s, 3H), 3.97 (s, 3H); ^{13}C NMR (126 MHz, $CDCl_3$) δ 156.04, 147.74, 147.63, 143.98, 143.93, 140.61, 138.13, 138.00, 135.59, 133.07, 132.83, 130.90, 129.97, 128.90, 128.81, 128.59, 128.42, 128.33, 128.30, 128.28, 128.25, 128.13, 128.11, 127.83, 127.77, 127.70, 127.57, 127.46, 126.49, 126.44, 126.35, 126.24, 125.04, 123.99, 123.97, 122.18, 122.09, 121.71, 84.20, 62.69; HRMS calcd for $C_{27}H_{21}NNaO_2^+$ ([M+Na]$^+$): 414.1465; found: 414.1465.

(32) 3-(2-(4-Fluorophenyl)-2-(4-methoxyphenyl)vinyl)isobenzofuran-1(3*H*)-one *O*-methyl oxime (33) (GFY-10-19, GFY-10-27).

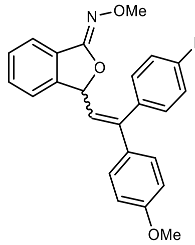

33 (1.6:1)

Following General Procedure I, after being stirred for 2 h, **33** was afforded as an oil (0.2 mmol scale, 60.8 mg, 80%, stereoisomers ratio = 1.6:1), eluent (0-6% of ethyl acetate in petroleum ether): ^{1}H NMR (500 MHz, $CDCl_3$) δ 7.68 (d, *J* = 7.6 Hz, 1.63H), 7.48 - 7.45 (m, 1.63H), 7.42 - 7.39 (m, 1.63H), 7.38 - 7.35 (m, 1.89H), 7.31 - 7.28 (m, 2.63H), 7.26 (d, *J* = 5.1 Hz, 0.63H), 7.24 - 7.22 (m, 1H), 7.17 (d, *J* = 8.5 Hz, 2H), 7.14 - 7.11 (m, 1.89H), 6.97 - 6.94 (m, 2.63H), 6.82 - 6.79 (m, 2H), 6.15 (d, *J* = 9.9 Hz, 0.63H), 6.05 (d, *J* = 9.9 Hz, 1H), 5.90 (d, *J* = 10.0 Hz, 1H), 5.86 (d, *J* = 9.9 Hz, 0.63H), 3.96 (s, 4.89H), 3.84 (s, 1.89H), 3.79 (s, 3H); ^{13}C NMR (126 MHz, $CDCl_3$) δ 163.71, 163.45, 161.74, 161.48, 159.84, 159.53, 155.99, 155.95, 146.76, 146.52, 144.02, 143.98, 137.35, 137.32, 134.28, 134.25, 133.09, 131.65, 131.58, 131.10, 130.87, 130.15, 129.56, 129.49, 128.93, 128.88, 128.40, 123.05, 122.07, 122.02, 121.68, 115.58, 115.41, 115.14, 114.97, 113.97, 113.62, 84.20, 76.75, 62.65, 55.21; ^{19}F NMR (471 MHz, $CDCl_3$) δ -113.61, -113.66; HRMS calcd for $C_{24}H_{20}FNNaO_3^+$ ([M+Na]$^+$): 412.1319; found: 412.1314.

(33) (*Z*)-3-((*E*)-2-Phenylprop-1-en-1-yl)isobenzofuran-1(3*H*)-one *O*-methyl oxime (34) (GFY-6-12/GFY-9-9).

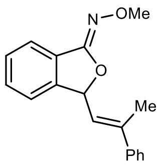

34

Following General Procedure I, after being stirred for 2 h, **34** was afforded as a white solid (0.2 mmol scale, 22.6 mg, 32%), eluent (0-8% of ethyl acetate in petroleum ether): m.p.= 120.4-120.9 °C (ethyl acetate/petroleum ether); ^{1}H NMR (500 MHz, $CDCl_3$) δ 7.70 (d, *J* = 7.6 Hz, 1H), 7.47 (td, *J* = 7.5, 1.2 Hz, 1H), 7.44 - 7.39 (m, 3H), 7.34 - 7.30 (m, 2H), 7.30 - 7.26 (m, 2H), 6.48 (d, *J* = 9.3 Hz, 1H), 5.74 (dd, *J* = 9.3, 1.4 Hz, 1H), 3.97 (s, 3H), 2.35 (d, *J* = 1.5 Hz, 3H); ^{13}C NMR (126 MHz, $CDCl_3$) δ 156.19, 144.26, 141.95, 141.76, 130.95, 128.86, 128.37, 128.29, 127.90, 125.98, 123.40, 121.89, 121.67, 83.11, 62.68, 16.66; HRMS calcd for $C_{18}H_{18}NO_2^+$ ([M+H]$^+$): 280.1332; found: 280.1338.

(34) (*Z*)-3-(2,2-Diphenylvinyl)-1-(methoxyimino)-*N*,*N*-dipropyl-1,3-dihydroisobenzofuran-5-sulfonamide (36) (GFY-6-18).

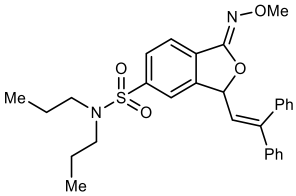

36

Following General Procedure I, after being stirred overnight, **36** was afforded as a white solid (0.2 mmol scale, 72.2 mg, 71%), eluent (0-21% of ethyl acetate in petroleum ether): m.p.= 130.1-132.2 °C (ethyl acetate/petroleum ether); ^{1}H NMR (500 MHz, $CDCl_3$) δ 7.84 (dd, *J* = 8.0, 1.5 Hz, 1H), 7.78 (d, *J* = 8.1 Hz, 1H), 7.73 (s, 1H), 7.49 - 7.42 (m, 2H), 7.43 - 7.38 (m, 3H), 7.31 - 7.24 (m, 3H), 7.28 - 7.21 (m, 2H), 6.17 (d, *J* = 9.9 Hz, 1H), 5.96 (d, *J* = 9.9 Hz, 1H), 3.97 (s, 3H), 3.11 (q, *J* = 7.3 Hz, 4H), 1.55 (q, *J* = 7.5 Hz, 4H), 0.87 (t, *J* = 7.3 Hz, 6H); ^{13}C NMR (126 MHz, $CDCl_3$) δ 154.47, 149.25, 144.66, 142.97, 140.42, 137.86, 132.20, 129.86, 128.61, 128.54, 128.25, 127.76, 127.67, 122.29, 122.25, 121.15, 84.10, 62.94, 49.78, 21.81, 11.09; HRMS calcd for $C_{29}H_{33}N_2O_4S^+$ ([M+H]$^+$): 505.2156; found: 505.2165.

(35) (*Z*)-3-(2,2-Diphenylvinyl)-5-(1-(3,5,5,8,8-pentamethyl-5,6,7,8-tetrahydronaphthalen-2-yl)vinyl)isobenzofuran-1(3*H*)-one *O*-methyl oxime (37) (GFY-6-21).

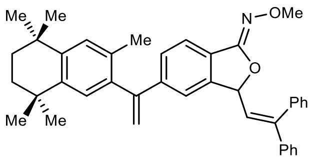

37

Following General Procedure I, after being stirred overnight, **37** was afforded as an oil (0.1 mmol scale, 20.8 mg, 36%, dr = 1:1), eluent (0-11% of ethyl acetate in petroleum ether): ^{1}H NMR (500 MHz, $CDCl_3$) δ 7.58 (d, *J* = 8.1 Hz, 1H), 7.41 - 7.32 (m, 4H), 7.27 - 7.25 (m, 3H), 7.23 - 7.20 (m, *J* = 7.3, 4.5, 2.9 Hz, 4H), 7.10 (s, 3H), 6.02 (d, *J* = 9.7 Hz, 1H), 5.96 (d, *J* = 9.7 Hz, 1H), 5.81 (d, *J* = 1.3 Hz, 1H), 5.31 (d, *J* = 1.3 Hz, 1H), 3.95 (s, 3H), 1.97 (s, 3H), 1.68 (s, 4H), 1.30 (s, 3H), 1.27 (s, 6H), 1.21 (s, 3H); ^{13}C NMR (126 MHz, $CDCl_3$) δ 156.01, 148.75, 147.35, 144.41, 144.40, 144.06, 142.39, 140.66, 138.22, 138.01, 132.70, 129.79, 128.51, 128.21, 128.06, 128.03, 127.94, 127.61, 127.39, 127.32, 123.90, 121.46, 120.19, 116.18, 84.07, 62.71, 35.19, 35.15, 33.98, 33.86, 31.92, 31.87, 31.85, 19.90; HRMS calcd for $C_{40}H_{42}NO_2^+$ ([M+H]$^+$): 568.3210; found: 568.3220.

(36) (*Z*)-3-(2,2-diphenylvinyl)-5-((((1*S*,2*S*,4*S*)-1,7,7-trimethylbicyclo[2.2.1]heptan-2-yl)oxy)methyl)isobenzofuran-1(3*H*)-one *O*-methyl oxime (38) (GFY-12-50, WYQ-6-1).

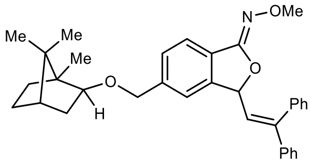

38

Following General Procedure I, after being stirred for 2 h, **38** was afforded as an oil (0.1 mmol scale, 32.0 mg, 46%, dr = 1:1), eluent (0-7% of ethyl acetate in petroleum ether): ^{1}H NMR (500 MHz, $CDCl_3$) 7.61 (d, *J* = 8.2 Hz, 1H),δ 7.45 - 7.41 (m, 2H), 7.40 - 7.35 (m, 4H), 7.31 (d, *J* = 7.0 Hz, 2H), 7.27 (s, 5H), 6.08 (d, *J* = 9.9 Hz, 1H), 5.97 (d, *J* = 9.9 Hz, 1H), 4.60 (dd, *J* = 13.2, 5.8 Hz, 1H), 4.43 (dd, *J* = 13.2, 6.3 Hz, 1H), 3.95

(s, 3H), 3.33 (dd, J = 7.6, 3.6 Hz, 1H), 1.87 - 1.82 (m, 1H), 1.74 - 1.67 (m, 2H), 1.67 - 1.58 (m, 2H), 1.56 (s, 3H), 1.53 - 1.48 (m, 1H), 1.06 (d, J = 2.4 Hz, 3H), 0.97 (d, J = 2.3 Hz, 3H), 0.83 (d, J = 2.1 Hz, 3H); ^{13}C NMR (126 MHz, $CDCl_3$) δ 156.14, 147.60, 144.30, 143.45, 140.82, 138.27, 129.89, 128.54, 128.23, 128.02, 127.73, 127.47, 127.17, 123.84, 121.42, 120.31, 87.08, 84.17, 84.14, 70.16, 62.67, 49.43, 46.53, 45.11, 38.45, 34.40, 27.26, 20.29, 20.23, 11.98; HRMS calcd for $C_{34}H_{38}NO_3^+$ ($[M+H]^+$): 508.2846; found: 508.2856.

(37) (*Z*)-3-(2,2-Diphenylvinyl)-5-((((1*R*,2*S*,5*R*)-2-isopropyl-5-methylcyclohexyl) oxy)methyl)isobenzofuran-1(3*H*)-one *O*-methyl oxime (39) (GFY-7-50).

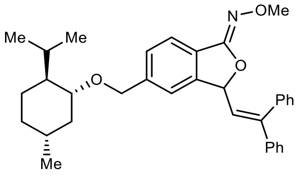

39

Following General Procedure I, after being stirred overnight, **39** was afforded as an oil (0.1 mmol scale, 31.8 mg, 61%, dr = 1:1), eluent (0-6% of ethyl acetate in petroleum ether): ^{1}H NMR (500 MHz, $CDCl_3$) δ 7.64 (d, J = 7.9 Hz, 1H), 7.43 (d, J = 7.0 Hz, 2H), 7.41 - 7.35 (m, 3H), 7.34 (d, J = 9.6 Hz, 2H), 7.27 (s, 5H), 6.10 (dd, J = 9.9, 2.7 Hz, 1H), 5.97 (dd, J = 10.0, 3.1 Hz, 1H), 4.73 (dd, J = 12.1, 6.9 Hz, 1H), 4.45 (dd, J = 12.1, 5.3 Hz, 1H), 3.96 (s, 3H), 3.22 - 3.17 (m, 1H), 2.33 - 2.25 (m, 1H), 2.21 - 2.17 (m, 1H), 1.68 - 1.62 (m, 3H), 1.44 - 1.24 (m, 3H), 0.98 - 0.92 (m, 3H), 0.91 (d, J = 2.6 Hz, 2H), 0.89 (d, J = 2.7 Hz, 1H), 0.86 (t, J = 2.5 Hz, 1H), 0.72 (t, J = 6.8 Hz, 3H); ^{13}C NMR (126 MHz, $CDCl_3$)δ 156.03, 147.77, 144.35, 142.80, 140.74, 138.16, 129.87, 128.51, 128.29, 128.24, 128.20, 128.03, 127.71, 123.59, 121.54, 121.16, 121.07, 84.06, 79.39, 69.79, 62.67, 48.25, 40.26, 34.45, 31.50, 25.69, 25.66, 23.24, 22.31, 20.94; HRMS calcd for $C_{34}H_{40}NO_3^+$ ($[M+H]^+$): 510.3003; found: 510.3013.

(38) Methyl ((*Z*)-3-(2,2-diphenylvinyl)-1-(methoxyimino)-1,3-dihydroisobenzofuran-5-carbonyl)-L-leucinate (40) (GFY-8-18).

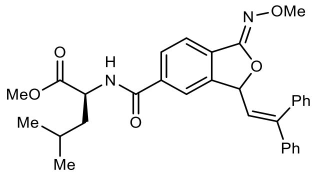

40

Following General Procedure I, after being stirred for 2 h, **40** was afforded as a white solid (0.1 mmol scale, 36.7 mg, 71%, dr = 1:1), eluent (0-25% of ethyl acetate in petroleum ether): ^{1}H NMR (500 MHz, $CDCl_3$) ^{1}H NMR (500 MHz, Chloroform-*d*) δ 7.82 - 7.75 (m, 1H), 7.74 - 7.69 (m, 1H), 7.45 - 7.42 (m, 2H), 7.40 - 7.38 (m, 3H), 7.28 - 7.72 (s, 4H), 6.67 (s, 1H), 6.15 - 6.12 (m, 1H), 5.96 - 5.92 (m, 1H), 4.88 - 4.83 (m, 1H),3.97 (s, 3H), 3.77 (s, 3H), 1.81 - 1.66 (m, 1H), 1.00 - 0.97 (m, 5H); ^{13}C NMR (126 MHz, $CDCl_3$) δ 173.48, 166.13, 155.10, 148.79, 148.73, 144.54, 140.51, 137.92, 136.44, 131.65, 129.87, 128.57, 128.41, 128.21, 128.15, 127.75, 127.36, 122.62, 121.81, 121.75, 84.14, 62.85, 52.45, 51.29, 41.72, 24.95, 22.75, 21.98; HRMS calcd for $C_{31}H_{33}N_2O_5^+$ ([M+H]$^+$): 513.2384; found: 513.2392.

(39) ((*Z*)-3-(2,2-Diphenylvinyl)-1-(methoxyimino)-1,3-dihydroisobenzofuran-5-yl)methyl (tert-butoxycarbonyl)-*L*-tyrosinate (41) (GFY-8-37).

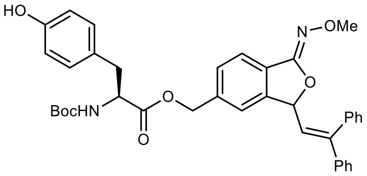

41

Following General Procedure I, after being stirred for 2 h, **41** was afforded as a white solid (0.1 mmol scale, 41.2 mg, 67%, dr = 1:1), eluent (0-40% of ethyl acetate in petroleum ether): ^{1}H NMR (500 MHz, $CDCl_3$) δ 7.66 (d, *J* = 7.9 Hz, 1H), 7.46 - 7.43 (m, 2H), 7.42 - 7.39 (m, 3H), 7.30 - 7.27 (m, 6H), 7.22 (d, *J* = 5.9 Hz, 1H), 6.87 (d, *J* = 8.0 Hz, 2H), 6.62 (dd, *J* = 8.7, 6.9 Hz, 2H), 6.13 (dd, *J* = 9.9, 2.1 Hz, 1H), 5.97 (dd, *J* = 9.9, 3.9 Hz, 1H), 5.21 (d, *J* = 12.6 Hz, 1H), 5.11 (dd, *J* = 12.6, 4.3 Hz, 1H), 5.00 (dd, *J* = 8.3, 4.3 Hz, 1H), 4.57 (q, *J* = 6.9 Hz, 1H), 3.98 (s, 3H), 3.01 - 2.96 (m, 1H), 1.80 (s, 1H), 1.41 (s, 9H); ^{13}C NMR (126 MHz, $CDCl_3$) δ 171.86, 155.86, 155.13, 148.42, 144.51, 140.63, 138.04, 130.32, 129.93, 129.31, 128.62, 128.42, 128.28, 128.18, 127.81, 127.79, 127.31, 123.08, 122.07, 121.97, 115.42, 84.13, 80.15, 77.20, 66.31,

66.27, 62.74, 54.65, 37.49, 28.26; HRMS calcd for $C_{38}H_{38}N_2NaO_7^+$ ([M+Na]$^+$):657.2571; found: 657.2577.

(40) ((*Z*)-3-(2,2-Diphenylvinyl)-1-(methoxyimino)-1,3-dihydroisobenzofuran-5-yl)methyl (4*R*)-4-((3*R*,5*R*,8*R*,9*S*,10*S*,13*R*,14*S*,17*R*)-3-hydroxy-10,13-dimethylhexadecahydro-1*H*-cyclopenta[*a*]phenanthren-17-yl)pentanoate (42) (GFY-8-5, GFY-8-17, GFY-10-43).

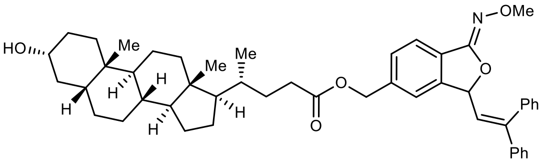

42

Following General Procedure I, after being stirred for 2 h, **42** was afforded as an oil (0.1 mmol scale, 32.3 mg, 45%, dr = 1:1), eluent (0-37% of ethyl acetate in petroleum ether): ^{1}H NMR (500 MHz, $CDCl_3$) (d, *J* = 7.9 Hz, 1H), 7.47 - 7.41 (m, 2H), 7.42 - 7.34 (m, 5H), 7.30 - 7.25 (m, 5H), 6.11 (d, *J* = 9.9 Hz, 1H), 5.97 (d, *J* = 9.9 Hz, 1H), 5.15 (t, *J* = 4.0 Hz, 2H), 3.96 (s, 3H), 3.63 (td, *J* = 10.8, 5.3 Hz, 1H), 2.41 (td, *J* = 10.0, 4.7 Hz, 1H), 2.34 - 2.24 (m, 1H), 1.95 - 1.89 (m, 2H), 1.88 - 1.77 (m, 6H), 1.76 - 1.66 (m, 2H), 1.56 - 1.46 (m, 3H), 1.44 - 1.30 (m, 10H), 1.28 - 1.18 (m, 6H), 1.12 - 1.04 (m, 4H), 1.03 - 0.95 (m, 4H), 0.93 - 0.87 (m, 6H), 0.59 (s, 3H); ^{13}C NMR (126 MHz, $CDCl_3$) δ 173.91, 155.70, 148.18, 144.48, 140.65, 139.46, 138.07, 129.91, 128.90, 128.66, 128.57, 128.37, 128.25, 128.13, 127.75, 123.25, 121.90, 121.70, 84.05, 71.83, 65.40, 62.75, 56.43, 55.84, 42.69, 42.04, 40.38, 40.10, 36.41, 35.79, 35.30, 35.27, 34.53, 31.17, 30.93, 30.50, 28.15, 27.15, 26.37, 24.14, 23.34, 20.77, 18.23, 11.98; HRMS calcd for $C_{48}H_{59}NNaO_5^+$ ([M+Na]$^+$): 752.4285; found: 752.4290.

2.2 Synthesis of *N*-alkoxy isoindolinones.

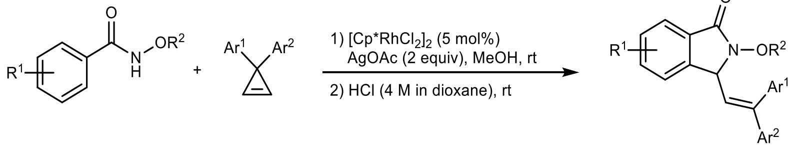

General Procedure II: To a 25 mL reaction tube equipped with a magnetic stir bar was added *N*-alkoxy benzamide (1.0 equiv), cyclopropene (1.5 equiv), [Cp*RhCl$_2$]$_2$

(5 mol%), AgOAc (2 equiv), and MeOH (0.1 M) successively. After being stirred at room temperature for 2 h, the reaction was complete as monitored by TLC. The mixture was filtered through a short pad of silica gel, washed with ethyl acetate, and concentrated in vacuo to afford a crude mixture.

To the above mixture was added HCl (4 M in dioxane) with a syringe to make a 0.05 M solution (concentration corresponding to *N*-alkoxy benzamide). After being stirred overnight at room temperature, the isomerization was complete as monitored by TLC. The resulting mixture was quenched with saturated $NaHCO_3$ solution (5 mL), extracted with ethyl acetate (5 mL × 3), washed with brine (5 mL), dried over anhydrous $MgSO_4$, filtered, and concentrated. The residue was purified by column chromatography on silica gel to afford corresponding *N*-alkoxy isoindolinone.

(1) 3-(2,2-Diphenylvinyl)-2-methoxyisoindolin-1-one (2) (GFY-8-7).

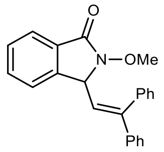

2

Following General Procedure II, **2** was afforded as an oil (0.2 mmol scale, 64.5 mg, 94%), eluent (0-15% of ethyl acetate in petroleum ether): ^1H NMR (500 MHz, $CDCl_3$) δ 7.85 (d, *J* = 7.6 Hz, 1H), 7.57 (td, *J* = 7.5, 1.2 Hz, 1H), 7.51 - 7.46 (m, 3H), 7.44 - 7.39 (m, 3H), 7.36 (d, *J* = 7.6 Hz, 1H), 7.29 - 7.26 (m, 5H), 5.89 (d, *J* = 10.1 Hz, 1H), 5.33 (d, *J* = 10.0 Hz, 1H), 3.95 (s, 3H); ^{13}C NMR (126 MHz, $CDCl_3$) δ 164.83, 148.60, 141.84, 140.63, 138.32, 132.27, 129.73, 129.59, 128.70, 128.67, 128.30, 128.21, 127.95, 127.38, 123.77, 123.08, 64.33, 60.01; HRMS calcd for $C_{23}H_{20}NO_2^+$ ($[M+H]^+$): 342.1489; found: 342.1498.

(2) 5-Bromo-3-(2,2-diphenylvinyl)-2-methoxyisoindolin-1-one (43) (GFY-7-49, GFY-6-39-3).

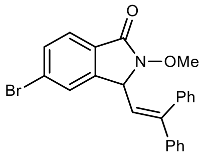

43

Following General Procedure II, **43** was afforded as a white solid (0.2 mmol scale, 65.9 mg, 78%), eluent (0-16% of ethyl acetate in petroleum ether): m.p.= 72.8-73.0 ^oC (ethyl acetate/petroleum ether); ^1H NMR (500 MHz, $CDCl_3$) δ 7.70 (d, *J* = 8.1 Hz, 1H), 7.62 (dd, *J* = 8.2, 1.7 Hz, 1H), 7.50 - 7.47 (m, 3H), 7.43 - 7.39 (m, 3H), 7.33 - 7.30 (m, 4H), 7.29 (d, *J* = 3.8 Hz, 1H), 5.86 (d, *J* = 10.0 Hz, 1H), 5.30 (d, *J* = 10.0 Hz, 1H), 3.94 (s, 3H); ^{13}C NMR (126 MHz, $CDCl_3$) δ 163.97, 149.44, 143.59, 140.36, 138.07, 132.19, 129.67, 128.80, 128.54, 128.42, 128.36, 128.11, 127.42, 126.96, 126.43, 125.31, 122.00, 64.45, 59.70; HRMS calcd for $C_{23}H_{18}{}^{79}BrNNaO_2^+$ ([M+Na]$^+$): 442.0414; found: 442.0418.

Crystal data and structure refinement of 43:

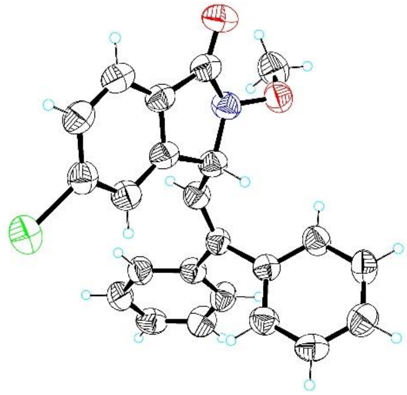

Bond precision:	C-C = 0.0084 A		Wavelength=1.54178
Cell:	a=11.00000	b=13.97200	c=15.42300
	alpha=73.4700	beta=72.0400	gamma=84.3400
Temperature:	300 K		
	Calculated		Reported
Volume	2161.541		2162
Space group	P -1		P -1
Hall group	-P 1		-P 1
Moiety formula	C23 H18 Br N O2 [+ solvent]		C23 H18 Br N O2
Sum formula	C23 H18 Br N O2 [+ solvent]		C23 H18 Br N O2
Mr	420.28		420.29
Dx,g cm-3	1.291		1.291

Z	4	4
Mu (mm-1)	2.713	2.713
F000	856.0	856.0
F000'	855.37	
h,k,lmax	13,16,18	0,0,0
Nref	7609	7504
Tmin,Tmax	0.758,0.805	0.864,0.864
Tmin'	0.688	

Correction method= # Reported T Limits: Tmin=0.864 Tmax=0.864

AbsCorr = MULTI-SCAN

Data completeness= 0.986	Theta(max)= 66.453
R(reflections)= 0.0898(4846)	wR2(reflections)= 0.2935(7504)
S = 1.065	Npar= 488

(3) Methyl 3-(2,2-diphenylvinyl)-2-methoxy-1-oxoisoindoline-5-carboxylate (44) (GFY-8-20).

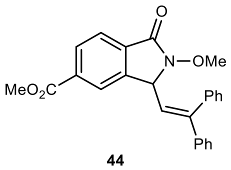

44

Following General Procedure II, **44** was afforded as a white solid (0.2 mmol scale, 70.4 mg, 89%), eluent (0-20% of ethyl acetate in petroleum ether): m.p.= 64.2-64.5 ^{o}C (ethyl acetate/petroleum ether); ^{1}H NMR (500 MHz, $CDCl_3$) δ 8.16 (dd, *J* = 8.0, 1.4 Hz, 1H), 8.01 (s, 1H), 7.90 (d, *J* = 7.9 Hz, 1H), 7.53 - 7.46 (m, 2H), 7.48 - 7.40 (m, 3H), 7.33 - 7.25 (m, 5H), 5.86 (d, *J* = 10.0 Hz, 1H), 5.38 (d, *J* = 10.0 Hz, 1H), 3.96 (s, 3H), 3.95 (s, 3H); ^{13}C NMR (126 MHz, $CDCl_3$) δ 166.12, 163.44, 149.56, 141.85, 140.43, 138.13, 133.73, 133.72, 130.09, 129.76, 128.79, 128.41, 128.37, 128.10, 127.44, 124.41, 123.83, 121.95, 77.26, 64.44, 60.00, 52.56; HRMS calcd for $C_{25}H_{22}NO_4^+$ ($[M+H]^+$): 400.1543; found: 400.1552.

(4) 3-(2,2-Diphenylvinyl)-2-methoxy-5-methylisoindolin-1-one (45) (GFY-8-3).

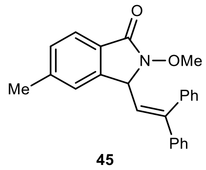

45

Following General Procedure II, **45** was afforded as a white solid (0.2 mmol scale, 56.7 mg, 80%), eluent (0-7% of ethyl acetate in petroleum ether): m.p.= 136.3-136.7 °C (ethyl acetate/petroleum ether); ^{1}H NMR (500 MHz, $CDCl_3$) δ 7.73 (d, *J* = 7.7 Hz, 1H), 7.48 (t, *J* = 7.5 Hz, 2H), 7.45 - 7.39 (m, 3H), 7.32 - 7.24 (m, 6H), 7.14 (s, 1H), 5.89 (d, *J* = 10.0 Hz, 1H), 5.28 (d, *J* = 10.0 Hz, 1H), 3.93 (s, 3H), 2.45 (s, 3H); ^{13}C NMR (126 MHz, $CDCl_3$) δ 165.27, 148.40, 143.14, 142.23, 140.72, 138.36, 129.79, 129.68, 128.70, 128.32, 128.20, 127.94, 127.42, 126.90, 123.69, 123.52, 123.35, 64.36, 59.98, 22.00; HRMS calcd for $C_{24}H_{22}NO_2^+$ ($[M+H]^+$): 356.1645; found: 356.1654.

(5) 3-(2,2-Diphenylvinyl)-2,5-dimethoxyisoindolin-1-one (46) (GFY-8-8).

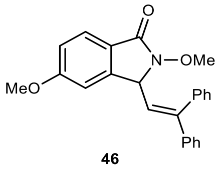

46

Following General Procedure II, **46** was afforded as a white solid (0.2 mmol scale, 62.3 mg, 85%), eluent (0-30% of ethyl acetate in petroleum ether): m.p.= 148.0-148.4 °C (ethyl acetate/petroleum ether); ^{1}H NMR (500 MHz, $CDCl_3$) δ 7.76 (d, *J* = 8.4 Hz, 1H), 7.50 - 7.44 (m, 2H), 7.44 - 7.37 (m, 3H), 7.32 -7.27 (m, 5H), 6.98 (dd, *J* = 8.5, 2.3 Hz, 1H), 6.81 (d, *J* = 2.2 Hz, 1H), 5.92 (d, *J* = 10.0 Hz, 1H), 3.93 (s, 3H), 3.86 (s, 3H); ^{13}C NMR (126 MHz, $CDCl_3$) δ 165.56, 163.38, 148.44, 144.24, 140.70, 138.34, 129.75, 128.72, 128.33, 128.23, 127.97, 127.42, 125.47, 123.37, 121.83, 114.76, 108.41, 64.42, 60.14, 55.64; HRMS calcd for $C_{24}H_{22}NO_3$ ($[M+H]^+$): 372.1594; found: 372.1603.

(6) 4,6-Dichloro-3-(2,2-diphenylvinyl)-2-methoxyisoindolin-1-one (47) (GFY-8-12).

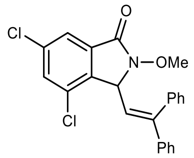

47

Following General Procedure II, **47** was afforded as an oil (0.2 mmol scale, 50.8 mg, 61%), eluent (0-11% of ethyl acetate in petroleum ether): ^{1}H NMR (500 MHz, $CDCl_3$) δ 7.74 (d, *J* = 1.9 Hz, 1H), 7.52 (d, *J* = 1.9 Hz, 1H), 7.49 - 7.40 (m, 5H), 7.34 - 7.28 (m, 3H), 7.28 - 7.23 (m, 2H), 5.68 (d, *J* = 10.1 Hz, 1H), 5.36 (d, *J* = 10.1 Hz, 1H), 3.94 (s, 3H); ^{13}C NMR (126 MHz, $CDCl_3$) δ 161.64, 150.56, 140.87, 138.46, 137.25, 135.81, 133.27, 132.57, 130.63, 129.72, 128.54, 128.39, 128.33, 128.03, 127.55, 122.56, 119.62, 64.52, 59.17; HRMS calcd for $C_{23}H_{17}{}^{35}Cl_2NNaO_2^+$ ($[M+Na]^+$): 432.0529; found: 432.0536.

(7) 3-(2,2-Diphenylvinyl)-2-methoxy-5-vinylisoindolin-1-one (48) (GFY-8-11).

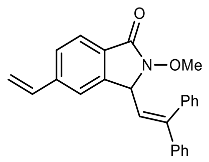

48

Following General Procedure II, **48** was afforded as a white solid (0.2 mmol scale, 59.6 mg, 84%), eluent (0-20% of ethyl acetate in petroleum ether): m.p.= 90.2-91.1 ^{o}C (ethyl acetate/petroleum ether); ^{1}H NMR (500 MHz, $CDCl_3$) δ 7.80 (d, *J* = 7.8 Hz, 1H), 7.54 - 7.46 (m, 3H), 7.45 - 7.40 (m, 3H), 7.34 - 7.32 (m, 1H), 7.30 (s, 4H), 6.77 (dd, *J* = 17.6, 10.9 Hz, 1H), 5.91 (d, *J* = 10.0 Hz, 1H), 5.85 (d, *J* = 17.5 Hz, 1H), 5.39 (d, *J* = 10.9 Hz, 1H), 5.33 (d, *J* = 10.0 Hz, 1H), 3.94 (s, 3H); ^{13}C NMR (126 MHz, $CDCl_3$) δ 164.76, 148.71, 142.40, 141.82, 140.61, 138.30, 136.02, 129.76, 128.86, 128.73, 128.33, 128.27, 128.00, 127.41, 126.78, 124.02, 122.95, 120.64, 116.56, 64.37, 60.03; HRMS calcd for $C_{25}H_{22}NO_2^+$ ($[M+H]^+$): 368.1645; found: 368.1644.

(8) 5-(Chloromethyl)-3-(2,2-diphenylvinyl)-2-methoxyisoindolin-1-one (49) (GFY-8-23).

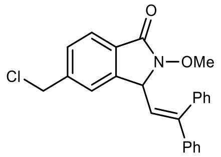

49

Following General Procedure II, **49** was afforded as a white solid (0.2 mmol scale, 61.8 mg, 79%), eluent (0-18% of ethyl acetate in petroleum ether): m.p.= 120.3-120.7

°C (ethyl acetate/petroleum ether); ^{1}H NMR (500 MHz, $CDCl_3$) δ 7.83 (d, *J* = 7.8 Hz, 1H), 7.51 - 7.48 (m, 3H), 7.45 - 7.40 (m, 3H), 7.37 (s, 1H), 7.32 - 7.27 (m, 5H), 5.89 (d, *J* = 10.0 Hz, 1H), 5.34 (d, *J* = 10.0 Hz, 1H),4.64 (s, 2H), 3.94 (s, 3H); ^{13}C NMR (126 MHz, $CDCl_3$)δ 164.16, 149.06, 142.42, 141.95, 140.49, 138.18, 129.71, 129.16, 128.75, 128.32, 128.03, 127.40, 124.19, 123.14, 122.49, 64.37, 59.92, 45.51; HRMS calcd for $C_{24}H_{20}{}^{35}ClNNaO_2^+$ ([M+Na]$^+$): 412.1075; found: 412.1083.

(9) 3-(2,2-Diphenylvinyl)-2-methoxy-2,3-dihydro-1H-benzo[f]isoindol-1-one (50) (GFY-8-26, W-5-46).

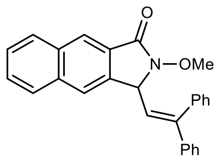

50

Following General Procedure II, **50** was afforded as an oil (0.2 mmol scale, 57.6 mg, 74%), eluent (0-31% of ethyl acetate in petroleum ether): ^{1}H NMR (500 MHz, $CDCl_3$) δ 8.38 (s, 1H), 7.99 (d, *J* = 8.0 Hz, 1H), 7.90 (d, *J* = 8.0 Hz, 1H), 7.76 (s, 1H), 7.62 - 7.53 (m, 2H), 7.51- 7.49 (m, 4H), 7.47 - 7.39 (m, 1H), 7.30 (s, 5H), 5.98 (d, *J* = 10.0 Hz, 1H), 5.48 (d, *J* = 9.9 Hz, 1H), 3.99 (s, 3H); ^{13}C NMR (126 MHz, $CDCl_3$)δ 164.45, 148.55, 140.72, 138.39, 137.01, 135.37, 133.06, 129.86, 129.50, 128.75, 128.35, 128.26, 128.04, 128.02, 127.85, 127.46, 127.29, 126.66, 124.33, 123.59, 122.28, 64.34, 59.88; HRMS calcd for $C_{27}H_{21}NNaO_2^+$ ([M+Na]$^+$): 414.1465; found: 414.1474.

(10) 3-(2,2-Diphenylvinyl)-2-methoxy-2,3-dihydro-1H-benzo[e]isoindol-1-one (51) (GFY-4-36, GFY-10-5, GFY-11-39).

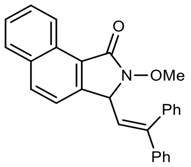

51

Following General Procedure II, **51** was afforded as a white solid (0.2 mmol scale, 31.2 mg, 40%), eluent (0-18% of ethyl acetate in petroleum ether): m.p.= 141.1-143.5 °C (ethyl acetate/petroleum ether); ^{1}H NMR (500 MHz, $CDCl_3$) δ 9.13 (d, *J* = 8.4 Hz,

1H), 8.03 (d, *J* = 8.4 Hz, 1H), 7.90 (d, *J* = 8.2 Hz, 1H), 7.67 (t, *J* = 7.6 Hz, 1H), 7.57 (t, *J* = 7.6 Hz, 1H), 7.53 - 7.45 (m, 4H), 7.43 - 7.39 (m, 2H), 7.29 (s, 5H), 5.91 (d, *J* = 10.1 Hz, 1H), 5.39 (d, *J* = 10.1 Hz, 1H), 4.01 (s, 3H); ^{13}C NMR (126 MHz, $CDCl_3$) (126 MHz, $CDCl_3$) δ 166.84, 148.79, 142.24, 140.81, 138.49, 133.24, 129.84, 129.23, 128.80, 128.37, 128.26, 128.21, 128.02, 127.47, 126.87, 124.00, 123.66, 123.19, 120.00, 64.58, 60.22; HRMS calcd for $C_{27}H_{22}NO_2^+$ ($[M+H]^+$): 392.1645; found: 392.1653.

(11) 3-(2,2-Diphenylvinyl)-2-methoxy-6-methylisoindolin-1-one (52) (GFY-8-27).

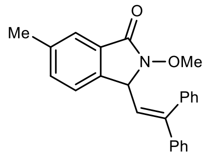

52

Following General Procedure II, **52** was afforded as an oil (0.2 mmol scale, 75.3 mg, 99%), eluent (0-25% of ethyl acetate in petroleum ether): ^{1}H NMR (500 MHz, $CDCl_3$) δ 7.68 (s, 1H), 7.50 (t, *J* = 7.5 Hz, 2H), 7.48 - 7.41 (m, 3H), 7.40 (d, *J* = 7.5 Hz, 1H), 7.36 - 7.24 (m, 5H), 5.90 (d, *J* = 10.0 Hz, 1H), 5.31 (d, *J* = 10.1 Hz, 1H), 3.97 (s, 3H), 2.45 (s, 3H); ^{13}C NMR (126 MHz, $CDCl_3$) δ 165.09, 148.31, 140.73, 139.06, 138.84, 138.38, 133.22, 129.76, 129.59, 128.68, 128.29, 128.16, 127.91, 127.39, 124.02, 123.43, 122.85, 64.31, 59.91, 21.35; HRMS calcd for $C_{24}H_{22}NO_2^+$ ($[M+H]^+$): 356.1645; found: 356.1654.

(12) 3-(2,2-Diphenylvinyl)-2-methoxy-7-methylisoindolin-1-one (53) (GFY-4-22, GFY-10-4).

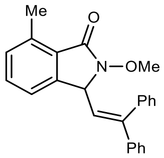

53

Following General Procedure II, **53** was afforded as an oil (0.2 mmol scale, 26.9 mg, 38%), eluent (0-15% of ethyl acetate in petroleum ether): ^{1}H NMR (500 MHz, $CDCl_3$) δ 7.50 - 7.45 (m, 2H), 7.44 - 7.39 (m, 4H), 7.30 - 7.27 (m, 5H), 7.20 (d, *J* = 7.4 Hz, 1H), 7.15 (d, *J* = 7.2 Hz, 1H), 5.89 (d, *J* = 10.0 Hz, 1H), 5.24 (d, *J* = 10.0 Hz, 1H), 3.94 (s, 3H), 2.70 (s, 3H); ^{13}C NMR (126 MHz, $CDCl_3$) δ 150.29, 149.63, 144.42,

140.15, 137.67, 132.57, 129.73, 128.78, 128.75, 128.46, 128.39, 127.76, 125.88, 124.67, 121.81, 121.58, 83.71, 77.20, 63.43; HRMS calcd for $C_{24}H_{22}NO_2^+$ ([M+H]$^+$): 356.1645; found: 356.1651.

(13) 3-(2,2-Diphenylvinyl)-2-ethoxyisoindolin-1-one (54) (GFY-8-24).

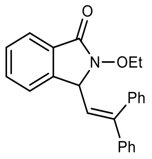

54

Following General Procedure II, **54** was afforded as a white solid (0.2 mmol scale, 62.0 mg, 87%), eluent (0-15% of ethyl acetate in petroleum ether): m.p.= 166.0-166.5 °C (ethyl acetate/petroleum ether); ^{1}H NMR (500 MHz, $CDCl_3$) δ 7.84 (d, *J* = 7.6 Hz, 1H), 7.56 (td, *J* = 7.5, 1.2 Hz, 1H), 7.51 - 7.44 (m, 1H), 7.43 - 7.38 (m, 3H), 7.36 (d, *J* = 7.6 Hz, 1H), 7.32 - 7.26 (m, 5H), 5.88 (d, *J* = 10.1 Hz, 1H), 5.32 (d, *J* = 10.1 Hz, 1H), 4.19 - 4.13 (m, 2H), 1.36 (t, *J* = 7.1 Hz, 3H); ^{13}C NMR (126 MHz, $CDCl_3$) δ 165.23, 148.40, 141.98, 140.71, 138.39, 132.19, 129.75, 129.73, 128.68, 128.64, 128.31, 128.19, 127.95, 127.40, 123.79, 123.38, 123.07, 72.29, 60.49, 13.78; HRMS calcd for $C_{24}H_{21}NNaO_2^+$ ([M+Na]$^+$): 378.1465; found: 378.1474.

(14) 3-(2,2-Di-*p*-tolylvinyl)-2-methoxyisoindolin-1-one (55) (GFY-8-13).

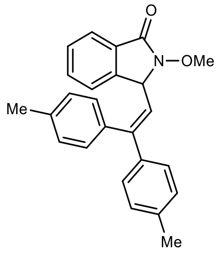

55

Following General Procedure II, **55** was afforded as an oil (0.2 mmol scale, 30.4 mg, 41%), eluent (0-20% of ethyl acetate in petroleum ether): ^{1}H NMR (500 MHz, $CDCl_3$) δ 7.84 (dt, *J* = 7.5, 1.0 Hz, 1H), 7.56 (td, *J* = 7.6, 1.2 Hz, 1H), 7.50 - 7.43 (m, 1H), 7.35 (dd, *J* = 7.4, 1.0 Hz, 1H), 7.29 (t, *J* = 8.8 Hz, 4H), 7.17 (d, *J* = 8.2 Hz, 2H), 7.10 (d, *J* = 8.1 Hz, 2H), 5.83 (d, *J* = 10.0 Hz, 1H), 5.34 (d, *J* = 10.0 Hz, 1H), 3.94 (s, 3H), 2.41 (s, 3H), 2.34 (s, 3H); ^{13}C NMR (126 MHz, $CDCl_3$) δ 164.89, 148.51, 142.10, 138.18, 138.13, 137.68, 135.53, 132.25, 129.67, 129.64, 129.37, 129.00, 128.62,

127.36, 123.78, 123.13, 122.01, 64.39, 60.23, 21.22, 21.09; HRMS calcd for $C_{25}H_{24}NO_2^+$ ([M+H]$^+$): 370.1802; found: 370.1812.

(15) 3-(2,2-Diphenylvinyl)-2-methoxy-1-oxo-*N*,*N*-dipropylisoindoline-5-sulfonamide (56) (GFY-8-28).

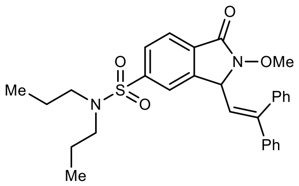

56

Following General Procedure II, **56** was afforded an oil (0.2 mmol scale, 95.8 mg, 93%), eluent (0-24% of ethyl acetate in petroleum ether): ^{1}H NMR (500 MHz, $CDCl_3$) δ 7.97 (d, *J* = 8.0 Hz, 1H), 7.91 (dd, *J* = 7.9, 1.5 Hz, 1H), 7.80 (s, 1H), 7.53 - 7.47 (m, 2H), 7.44 (dt, *J* = 8.0, 1.9 Hz, 3H), 7.34 - 7.29 (m, 3H), 7.29 - 7.26 (m, 2H), 5.86 (d, J = 10.0 Hz, 1H), 5.41 (d, *J* = 10.0 Hz, 1H), 3.97 (s, 3H), 3.13 - 3.10 (m, 4H), 1.56 (q, *J* = 7.5 Hz, 4H), 0.87 (t, *J* = 7.4 Hz, 6H); ^{13}C NMR (126 MHz, $CDCl_3$) δ 162.89, 150.06, 144.24, 142.49, 140.29, 138.00, 133.12, 129.68, 128.82, 128.53, 128.37, 128.19, 127.43, 127.32, 124.53, 121.96, 121.35, 64.51, 60.09, 49.79, 21.81, 11.08; HRMS calcd for $C_{29}H_{33}N_2O_4S^+$ ([M+H]$^+$): 505.2156; found: 505.2164.

(16) 3-(2,2-Diphenylvinyl)-5-((((1*R*,2*S*,5*R*)-2-isopropyl-5-methylcyclohexyl)oxy) methyl)-2-methoxyisoindolin-1-one (57) (GFY-8-30).

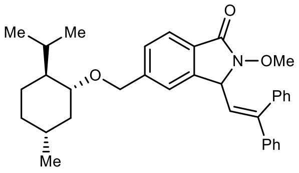

57

Following General Procedure II, **57** was afforded as an oil (0.1 mmol scale, 47.1 mg, 98%, dr = 1:1), eluent (0-21% of ethyl acetate in petroleum ether): ^{1}H NMR (500 MHz, $CDCl_3$) δ 7.80 (d, *J* = 7.8 Hz, 1H), 7.50 - 7.45 (m, 2H), 7.42 (d, *J* = 7.5 Hz, 4H), 7.38 (s, 1H), 7.33 - 7.24 (m, 5H), 5.88 (dd, *J* = 10.1, 3.7 Hz, 1H), 5.31 (dd, *J* = 10.1, 3.2 Hz, 1H), 4.75 (dd, *J* = 12.2, 5.5 Hz, 1H), 4.46 (dd, *J* = 12.3, 4.7 Hz, 1H), 3.94 (s, 3H), 3.23 - 3.17 (m, 1H), 2.35 - 2.25(m, 1H), 2.19 (dd, *J* = 12.1, 1.9 Hz, 1H), 1.69 - 1.61 (m, 2H), 1.45 - 1.24 (m, 3H), 0.94 (dd, *J* = 6.6, 3.6 Hz, 4H), 0.89 (d, *J* = 7.2 Hz,

4H), 0.72 (dd, J = 7.0, 3.4 Hz, 3H); ^{13}C NMR (126 MHz, $CDCl_3$) δ 164.97, 144.30, 142.16, 140.74, 138.37, 129.76, 128.71, 128.32, 128.21, 127.97, 127.89, 127.86, 127.43, 123.74, 123.25, 122.08, 122.02, 79.48, 69.91, 64.41, 60.14, 48.25, 40.25, 34.44, 31.49, 23.19, 22.30, 20.92, 16.13; HRMS calcd for $C_{34}H_{40}NO_3^+$ ($[M+H]^+$): 510.3003; found: 510.3010.

2.3 Transformation of products to assess the potential for post-modifications.

(1) Synthesis of (*Z*)-3,5-dibromo-3-(2,2-diphenylvinyl)isobenzofuran-1(3*H*)-one *O*-methyl oxime (58) (GFY-7-28, GFY-10-25).

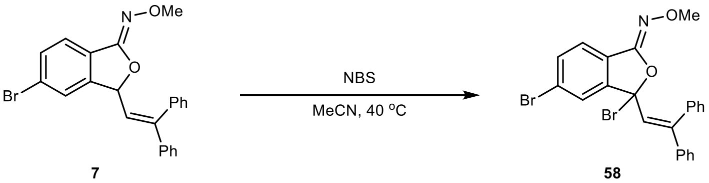

7 58

To a 10 mL of Schlenk tube was added *N*-bromosuccinimide (53.2 mg, 0.3 mmol), **7** (86.0 mg, 0.2 mmol), and acetonitrile (1 mL). The mixture was stirred at 40 °C overnight as monitored by TLC. The solution was cooled to room temperature and concentrated under vacuum to afford **58** (84.0 mg, 82%) via flash chromatography on silica gel (eluent: 8% of ethyl acetate in petroleum ether) as a white solid: m.p.= 150.0-150.4 °C (ethyl acetate/petroleum ether); ^{1}H NMR (500 MHz, $CDCl_3$) δ 7.67 - 7.54 (m, 2H), 7.51 - 7.43 (m, 1H), 7.41 - 7.37 (m, 4H), 7.36 - 7.34 (m, 2H), 7.33 (dd, J = 4.0, 2.9 Hz, 3H), 7.31 - 7.29 (m, 1H), 6.40 (s, 1H), 3.97 (s, 3H); ^{13}C NMR (126 MHz, $CDCl_3$) δ 154.61, 149.45, 144.38, 141.19, 139.30, 132.77, 128.98, 128.94, 128.87, 128.61, 128.51, 128.17, 125.21, 124.91, 122.94, 121.21, 115.13, 84.79, 62.94; HRMS calcd for $C_{23}H_{18}{}^{79}Br_2NO_2^+$ ($[M+H]^+$): 497.9699; found: 497.9707.

(2) Synthesis of 3,5-dibromo-3-(2,2-diphenylvinyl)-2-methoxyisoindolin-1-one (59) (GFY-9-19, GFY-10-34, W-2-25).

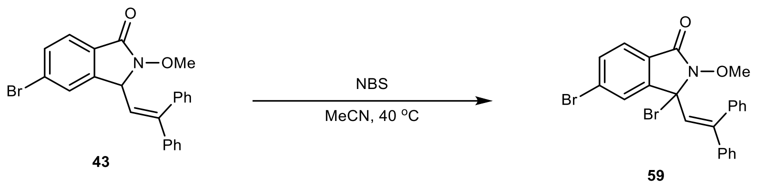

To a 10 mL of Schlenk tube was added *N*-bromosuccinimide (47.5 mg, 0.27 mmol), **43** (74.2 mg, 0.18 mmol), and acetonitrile (1 mL). The mixture was stirred at 40 °C overnight as monitored by TLC. The solution was cooled to room temperature and concentrated under vacuum to afford **59** (65.1 mg, 74%) via flash chromatography on silica gel (eluent: 20% of ethyl acetate in petroleum ether) as a white solid: m.p.= 60.5-60.7 °C (ethyl acetate/petroleum ether); ^{1}H NMR (500 MHz, $CDCl_3$) δ 7.70 (d, *J* = 8.2 Hz, 1H), 7.66 - 7.61 (m, 1H), 7.55 - 7.51 (m, 1H), 7.44 - 7.40 (m, 4H), 7.37 - 7.34 (m, 5H), 7.33 - 7.30 (m, 1H), 5.65 (s, 1H), 4.08 (s, 3H); ^{13}C NMR (126 MHz, $CDCl_3$) δ 164.51, 150.66, 142.26, 141.33, 139.48, 132.64, 129.15, 129.06, 128.79, 128.76, 128.45, 128.25, 128.17, 127.22, 125.65, 125.35, 120.62, 64.40, 63.66; HRMS calcd for $C_{23}H_{17}{}^{79}Br^{81}BrNNaO_2^+$ ([M+Na]$^+$): 521.9498; found: 521.9494.

(3) Synthesis of (*Z*)-3-(2,2-diphenylvinyl)-5-(4-hydroxybut-1-yn-1-yl)isobenzofuran-1(3*H*)-one *O*-methyl oxime (60) (GFY-7-37).

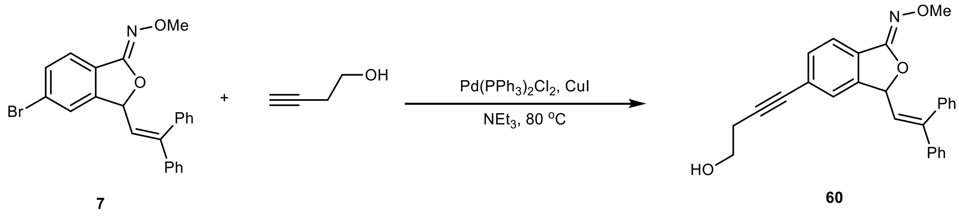

Under a nitrogen atmosphere, to a solution of **7** (42.3 mg, 0.1 mmol) in triethylamine (0.2 mL) was added $Pd(PPh_3)_2Cl_2$ (0.7 mg, 0.001 mmol), 3-butyn-1-ol (9.1 µL, 0.12 mmol), and CuI (0.3 mg, 1 mol%). The mixture was stirred overnight at 80 °C as monitored by TLC. After cooling to room temperature, the mixture was filtered through a short pad of silica gel and concentrated in vacuo. Flash chromatography on silica gel (eluent: 0-29% of ethyl acetate in petroleum ether) afforded **60** (21.1 mg, 52%) as a white solid: m.p.= 69.9-70.3 °C (ethyl acetate/petroleum ether); ^{1}H NMR (500 MHz, $CDCl_3$) δ 7.60 (d, *J* = 8.0 Hz, 1H), 7.47 - 7.41 (m, 3H), 7.40 - 7.36 (m, 3H), 7.33 (d, *J*

= 1.2 Hz, 1H), 7.29 - 7.26 (m,5H), 6.07 (d, *J* = 10.0 Hz, 1H), 5.95 (d, *J* = 10.0 Hz, 1H), 3.95 (s, 3H), 3.83 (t, *J* = 6.2 Hz, 2H), 2.71 (t, *J* = 6.3 Hz, 2H); ^{13}C NMR (126 MHz, $CDCl_3$) δ 155.57, 148.34, 144.13, 140.62, 138.03, 132.52, 129.90, 128.57, 128.38, 128.25, 128.14, 127.83, 127.77, 126.17, 125.28, 123.06, 121.57, 89.19, 83.89, 81.70, 62.79, 60.99, 23.83; HRMS calcd for $C_{27}H_{24}NO_3^+$ ($[M+H]^+$): 410.1751; found: 410.1744.

(4) Synthesis of 3-(2,2-diphenylvinyl)-5-(4-hydroxybut-1-yn-1-yl)-2-methoxyisoindolin-1-one (61) (GFY-9-32, W-2-27).

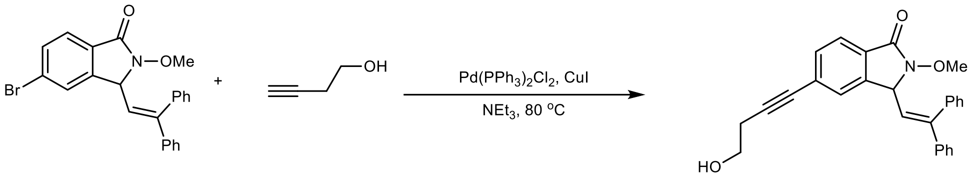

43 **61**

Under a nitrogen atmosphere, to a solution of **43** (42.4 mg, 0.1 mmol) in triethylamine (0.2 mL) was added $Pd(PPh_3)_2Cl_2$ (0.8 mg, 0.001 mmol), 3-butyn-1-ol (9.1 µL, 0.12 mmol), and CuI (0.3 mg, 0.001). The mixture was stirred overnight at 80 °C as monitored by TLC. After cooling to room temperature, the mixture was filtered through a short pad of silica gel and concentrated in vacuo. Flash chromatography on silica gel (eluent: 0-40% of ethyl acetate in petroleum ether) afforded **61** (40.3 mg, 98%) as a white solid: m.p.= 159.2-160.0 °C (ethyl acetate/petroleum ether); ^{1}H NMR (500 MHz, $CDCl_3$) δ 7.75 (d, *J* = 7.9 Hz, 1H), 7.50 - 7.47 (m, 3H), 7.44 - 7.39 (m, 3H), 7.36 (s, 1H), 7.31 - 7.26 (m, *J* = 10.3, 4.4 Hz, 5H), 5.85 (d, *J* = 10.1 Hz, 1H), 5.28 (d, *J* = 10.2 Hz, 1H), 3.93 (s, 3H), 3.84 (t, *J* = 6.3 Hz, 2H), 2.72 (t, *J* = 6.3 Hz, 2H), 2.10 (s, 1H); ^{13}C NMR (126 MHz, $CDCl_3$) δ 164.33, 149.08, 141.86, 140.49, 138.18, 132.25, 129.73, 128.75, 128.33, 128.03, 127.70, 127.41, 126.15, 123.68, 122.41, 90.00, 81.53, 64.43, 60.88, 59.88, 23.80; HRMS calcd for $C_{27}H_{24}NO_3^+$ ($[M+H]^+$): 410.1751; found: 410.1751.

(5) Synthesis of (*Z*)-3-(2,2-diphenylvinyl)-5-(4,4,5,5-tetramethyl-1,3,2-dioxaborolan-2-yl)isobenzofuran-1(3*H*)-one *O*-methyl oxime (62) (GFY-8-4).

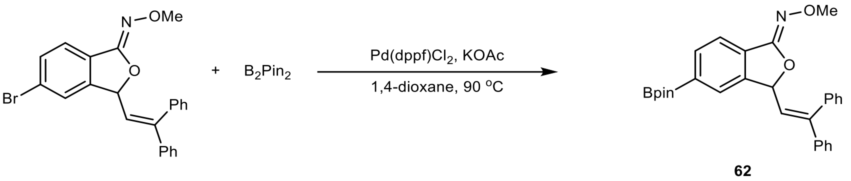

Under a nitrogen atmosphere, to a 10 mL of Schlenk tube was added **7** (42.4 mg, 0.1 mmol), B_2pin_2 (38.8 mg, 0.15 mmol), Pd(dppf)Cl_2 (3.7 mg, 0.005 mmol), KOAc (29.9 mg, 0.3 mmol), and 1,4-dioxane (1 mL). The mixture was stirred overnight at 90 ^oC as monitored by TLC. After cooling to room temperature, the mixture was filtered through a short pad of silica gel and concentrated in vacuo. Flash chromatography on silica gel (eluent: 0-17% of ethyl acetate in petroleum ether) afforded **62** (29.0 mg, 51%) as a white solid: m.p.= 198.4-199.1 ^oC (ethyl acetate/petroleum ether); ^1H NMR (500 MHz, $CDCl_3$) δ 7.86 (d, *J* = 7.7 Hz, 1H), 7.72 (s, 1H), 7.68 (d, *J* = 7.7 Hz, 1H), 7.46 - 7.41 (m, 4H), 7.40 - 7.36 (m, 1H), 7.28 (s, 5H), 6.13 (d, *J* = 10.0 Hz, 1H), 5.97 (d, *J* = 10.1 Hz, 1H), 3.96 (s, 3H), 1.36 (s, 12H); ^{13}C NMR (126 MHz, $CDCl_3$) δ 164.55, 148.90, 141.06, 140.68, 138.27, 135.15, 132.11, 129.93, 129.00, 128.67, 128.33, 128.25, 127.96, 127.45, 122.93, 122.70, 84.31, 75.01, 64.33, 59.91, 24.84, 24.82; HRMS calcd for $C_{29}H_{31}BNO_4^+$ ($[M+H]^+$): 468.2341; found: 468.2340.

(6) Synthesis of 3-(2,2-diphenylvinyl)-2-methoxy-5-(4,4,5,5-tetramethyl-1,3,2-dioxaborolan-2-yl)isoindolin-1-one (63) (GFY-9-36).

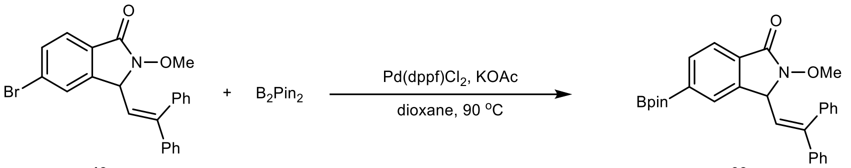

Under a nitrogen atmosphere, to a 10 mL of Schlenk tube was added **43** (42.0 mg, 0.1 mmol), B_2pin_2 (38.1 mg, 0.15 mmol), Pd(dppf)Cl_2 (3.7 mg, 0.005 mmol), KOAc (29.4 mg, 0.3 mmol), and 1,4-dioxane (1 mL). The mixture was stirred overnight at 90 ^oC as monitored by TLC. After cooling to room temperature, the mixture was filtered through a short pad of silica gel and concentrated in vacuo. Flash chromatography on silica gel (eluent: 0-30% of ethyl acetate in petroleum ether) afforded **63** (46.4 mg, 81%) as an oil: ^1H NMR (500 MHz, $CDCl_3$) δ 7.93 (d, *J* = 7.5 Hz, 1H), 7.84 (d, *J* = 7.5 Hz,

1H), 7.76 (s, 1H), 7.51 - 7.43 (m, 4H), 7.44 - 7.37 (m, 1H), 7.30 (s, 5H), 5.87 (d, J = 10.1 Hz, 1H), 5.33 (d, J = 10.1 Hz, 1H), 3.91 (s, 3H), 1.36 (s, 12H), 1.24 (s, 8.5H) ; ^{13}C NMR (126 MHz, $CDCl_3$) δ 164.55, 148.90, 141.06, 140.68, 138.27, 135.15, 132.11, 129.93, 129.00, 128.67, 128.33, 128.25, 127.96, 127.45, 122.93, 122.70, 84.31, 75.01, 64.33, 59.91, 24.84, 24.82; HRMS calcd for $C_{29}H_{31}BNO_4^+$ ($[M+H]^+$): 468.2341; found: 468.2345.

2.4 Synthesis of a potential PD-L1 down-regulator.

(1) 4-((*tert*-Butoxycarbonyl)amino)benzoic acid (66) (GFY-8-40).

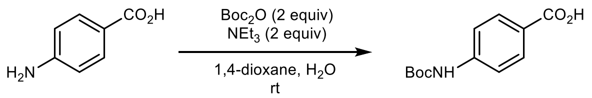

To a solution of **65** (1.3716 g, 10 mmol) in the mixture of dioxane (20 mL) and water (10 mL) was added triethylamine (2.8 mL, 20 mmol) and Boc_2O (4.6 mL, 20 mmol). The reaction mixture was stirred at room temperature for 24 h as monitored by TLC. After being concentrated in vacuo, to the residue was dropwisely added HCl (3 M, 30 mL). A precipitate formed, which was filtered, washed with water, and dried to provide **66** (2.8725 g, 99%). The characterization data was in agreement with literature.[S11]

(2) Methyl 4-((*tert*-butoxycarbonyl)amino)benzoic acid (67) (GFY-8-43, GFY-9-29).

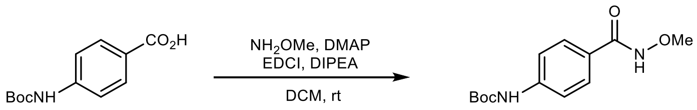

To a 250 mL of round bottom flask was added **66** (0.7322 g, 3.1 mmol), $NH_2OMe{\cdot}HCl$ (0.3324 g, 4 mmol), DMAP (0.3784 g, 3.1 mmol), EDC-HCl (1.1885 g, 6.2 mmol), DIPEA (2.0 mL, 11.2 mmol), and dichloromethane (40 mL). The mixture was stirred at room temperature overnight as monitored by TLC. After being

concentrated in vacuo, **67** (0.5177 g, 63%) was obtained via flash chromatography on silica gel (0-44% of ethyl acetate in petroleum ether). The characterization data was in agreement with literature.

(3) *tert*-Butyl (*Z*)-(3-(2,2-Diphenylvinyl)-1-(methoxyimino)-1,3-dihydroisobenzofuran-5-yl)carbamate (68) (GFY-9-3, GFY-8-48).

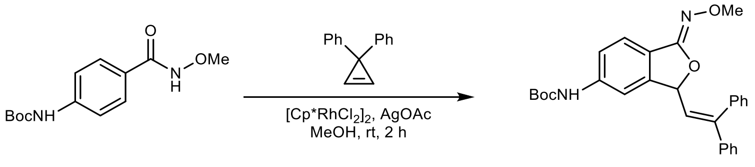

67 **68**, 81%

Following General Procedure I, after being reacted for 2 h, **68** (74.2 mg, 81%) was obtained via flash chromatography on silica gel (eluent: 11% of ethyl acetate in petroleum ether) as an oil: ^{1}H NMR (500 MHz, $CDCl_3$) δ 7.58 (d, *J* = 8.4 Hz, 1H), 7.54 (s, 1H), 7.45 - 7.41 (m, 2H), 7.41 - 7.37 (m, 3H), 7.32 - 7.29 (m, 1H), 7.28 - 7.24 (m, 5H), 6.99 (s, 1H), 6.08 - 6.02 (m, 1H), 5.98 - 5.92 (m, 1H), 3.96 (s, 3H), 1.53 (s, 9H); ^{13}C NMR (126 MHz, $CDCl_3$) δ 155.98, 148.10, 145.53, 141.34, 140.64, 138.01, 129.93, 128.46, 128.21, 128.13, 127.99, 127.72, 123.27, 122.47, 122.32, 119.12, 111.14, 83.85, 81.09, 62.53, 28.21; HRMS calcd for $C_{28}H_{29}N_2O_4^+$ ($[M+H]^+$): 457.2122; found: 457.2122.

(4) (*Z*)-5-Amino-3-(2,2-diphenylvinyl)isobenzofuran-1(3*H*)-one *O*-methyl oxime (69) (GFY-9-4/GFY-9-12).

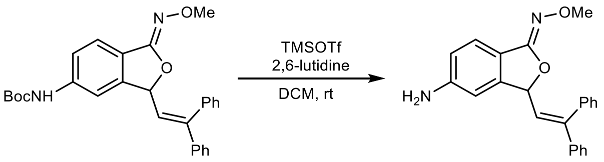

68 **69**, 81%

At 0 °C, to a solution of **68** (70.9 mg, 0.15 mmol) in dichloromethane (5 mL) was added 2,6-lutidine (109 µL, 0.93 mmol) and TMSOTf (112 µL, 0.62 mmol) dropwisely. The reaction mixture was stirred at 0 °C for 30 min, and then at room temperature for 2 hours as monitored by TLC. After being concentrated in vacuo, **69** (44.7 mg, 81%) was obtained via flash chromatography on silica gel (eluent: 0-18% of ethyl acetate in

petroleum ether) as a white solid: m.p.= 86.0-86.2 °C (ethyl acetate/petroleum ether); ^{1}H NMR (500 MHz, $CDCl_3$) δ 7.51 - 7.46 (m, 1H), 7.44 (d, *J* = 7.6 Hz, 2H), 7.41 - 7.36 (m, 3H), 7.29 (d, *J* = 1.4 Hz, 5H), 6.69 (dd, *J* = 8.4, 2.1 Hz, 1H), 6.53 (d, *J* = 2.1 Hz, 1H), 6.00 (d, *J* = 2.3 Hz, 2H), 4.04 (s, 2H), 3.94 (s, 3H); ^{13}C NMR (126 MHz, $CDCl_3$) δ 156.63, 149.41, 147.42, 146.35, 140.80, 138.20, 129.92, 128.49, 128.20, 128.00, 127.71, 123.94, 122.95, 118.02, 116.02, 106.90, 83.52, 62.48; HRMS calcd for $C_{23}H_{21}N_2O_2^+$ ($[M+H]^+$): 357.1598; found: 357.1595.

(5) 2-((*S*)-4-(4-Chlorophenyl)-2,3,9-trimethyl-6*H*-thieno[3,2-*f*][1,2,4]triazolo[4,3-*a*][1,4]diazepin-6-yl)-*N*-((*Z*)-3-(2,2-diphenylvinyl)-1-(methoxyimino)-1,3-dihydroisobenzofuran-5-yl)acetamide (64) (GFY-10-2).

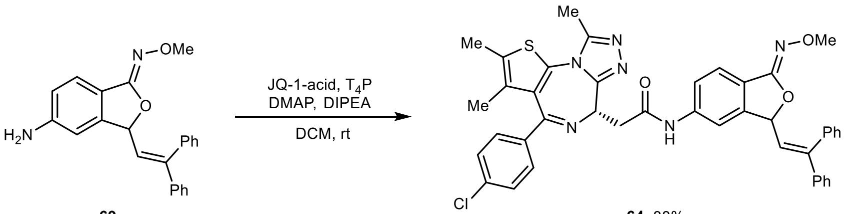

69 **64**, 88%

Under a nitrogen atmosphere, to a 50 mL of round bottom flask were added **69** (44.2 mg, 0.125 mmol), JQ-1-acid (39.7 mg, 0.1 mmol), DMAP (25.0 mg, 0.2 mmol), T_4P (50 % in ethyl acetate, 209.4 mg, 0.262 mmol), DIPEA (45 µL, 0.26 mmol), and dichloromethane (10 mL). The mixture was stirred at room temperature overnight as monitored by TLC. After being concentrated in vacuo, **64** (64.4 mg, 88%, dr = 1:1) was obtained via flash chromatography on silica gel (100% of ethyl acetate) as a white solid: m.p.= 215.8-216.1 °C; ^{1}H NMR (500 MHz, $CDCl_3$) δ 10.09 (d, *J* = 93.1 Hz, 1H), 7.88 (dd, *J* = 14.9, 1.7 Hz, 1H), 7.54 - 7.36 (m, 7H), 7.34 - 7.30 (m, 3H), 7.29 - 7.24 (m, 6H), 6.54 - 5.62 (m, 2H), 4.79 - 4.72 (m, 1H), 3.96 - 3.84 (m, 4H), 3.65 (ddd, *J* = 16.2, 14.8, 5.5 Hz, 1H), 2.63 (s, 3H), 2.40 (d, *J* = 2.1 Hz, 3H), 1.67 (d, *J* = 2.5 Hz, 3H); ^{13}C NMR (126 MHz, $CDCl_3$) δ 169.34, 169.31, 164.13, 155.90, 155.74, 150.05, 147.98, 147.45, 145.17, 145.01, 141.38, 141.23, 140.78, 140.58, 138.09, 138.07, 136.89, 136.88, 136.30, 131.89, 131.10, 130.94, 130.89, 130.49, 129.95, 129.92, 129.83, 128.65, 128.45, 128.16, 128.12, 128.10, 127.93, 127.88, 127.77, 127.71, 123.52, 123.48, 123.36, 123.19, 122.03, 121.93, 120.61, 120.41, 112.99, 112.77, 83.90, 83.87, 62.56,

62.53, 54.41, 54.39, 40.27, 40.19, 14.35, 14.32, 13.03, 11.71; HRMS calcd for $C_{42}H_{36}{}^{35}ClN_6O_3S^+$ ($[M+H]^+$): 739.2253; found: 739.2250.

2.5 Biological activity evaluation.

(1) Cell culture.

SUM159PT cells were maintained in RPMI-1640 medium (KeyGen BioTECH) supplemented with 10% (v/v) fetal bovine serum (FBS; VivaCell Bioscience). U-87 MG cells were cultured with Dulbecco's modified Eagle's medium media (DMEM; KeyGen BioTECH) with 10% FBS.

(2) Cell preparation.

Cells were cultured in 6-well plates. After confluence reached 70%, cells were treated with DMSO, JQ-1 (5 μM) or compound **64** (5 μM) for 12 h. After that, cells were washed by PBS and harvested for the following analysis.

(3) RNA extraction and qPCR analysis.

Total RNA was isolated with RNA isolater total RNA extraction reagent (Vazyme) and 1 μg of total RNA was used for cDNA synthesis using the HiScript II Q RT SuperMix (Vazyme). Quantitative PCRs were carried out using AceQ qPCR SYBR green master mix (Vazyme). Samples were obtained and analyzed on the LightCycler 480 System (Roche). The gene expression levels were normalized to β-Actin. The primer sequences were as follows:

β-Actin forward: 5'-GATCATTGCTCCTCCTGAGC-3'

β-Actin reverse: 5'-ACTCCTGCTTGCTGATCCAC-3'

PD-L1 forward: 5'-TGGCATTTGCTGAACGCATTT-3'

PD-L1 reverse: 5'-TGCAGCCAGGTCTAATTGTTTT-3'

(4) Statistical analysis.

The experiments were carried out in three independent experiments and the results are expressed as mean ± SD. One-way analysis of variance (ANOVA) was used for assessment of the statistical significance, and data were considered significant at $p \leq 0.05$. GraphPad Prism 8.0 software was used to perform all the statistical analyses and generating the graphics corresponding to these statistics.

(5) Immunoblotting.

Cells were washed twice with ice-cold PBS, lysed and denaturated with SDS-PAGE loading buffer (NCM Biotech). The proteins were separated with SDS-PAGE, transferred to PVDF membranes, and blocked with 5% skim milk and incubated with indicated antibodies (β-Actin (Abclonal, AC026), BRD4 (Proteintech, 28486-1-AP)). Imunoreactive bands were visualized with HRP-conjugated secondary antibodies (HRP Goat Anti-Rabbit IgG (H+L) (Abclonal AS014)).

2.6 Experimental mechanistic studies.

(1) H/D Exchange with $[RhCp^*Cl_2]_2$ (GFY-12-20-1)

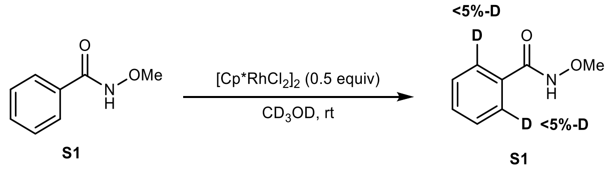

To a 10 mL of Schlenk tube was charged with **S1** (7.8 mg, 0.05 mmol), $[Cp^*RhCl_2]_2$ (15.4 mg, 0.025 mmol) and CD_3OD (0.5 mL). After being stirred at room temperature for 2 hours, the reaction mixture was directly analyzed by ^{1}H NMR, and less than 5%-D incorporation was observed at ortho positions.

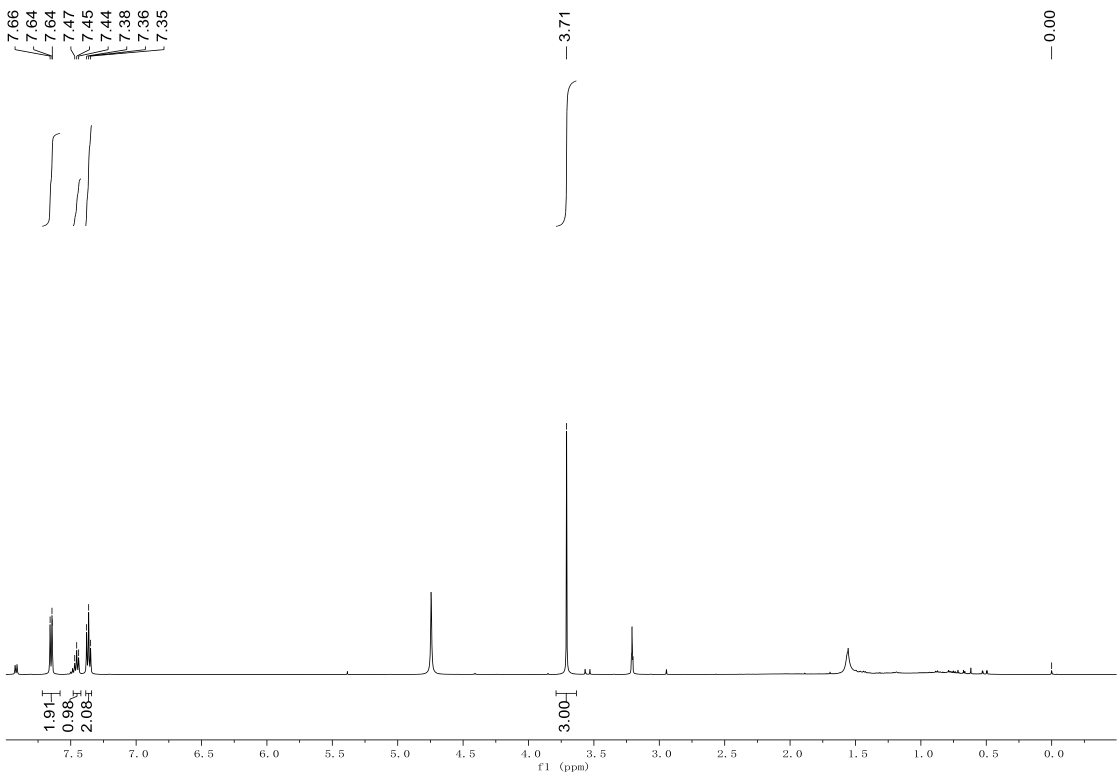

(2) H/D Exchange with RhCp*(OAc)2 (GFY-12-19-1)

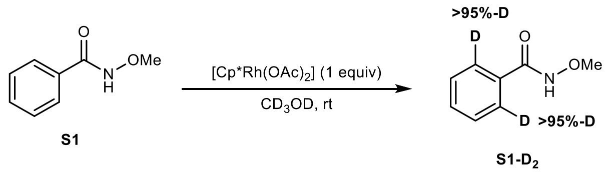

To a 10 mL of Schlenk tube was charged with **S1** (7.6 mg, 0.05 mmol), Cp*Rh(OAc)2 (18.5 mg, 0.05 mmol) and CD_3OD (0.5 mL). After being stirred at room temperature for 2 hours, the reaction mixture was directly analyzed by ^{1}H NMR, and an average of more than 95%-D incorporation was observed at ortho positions.

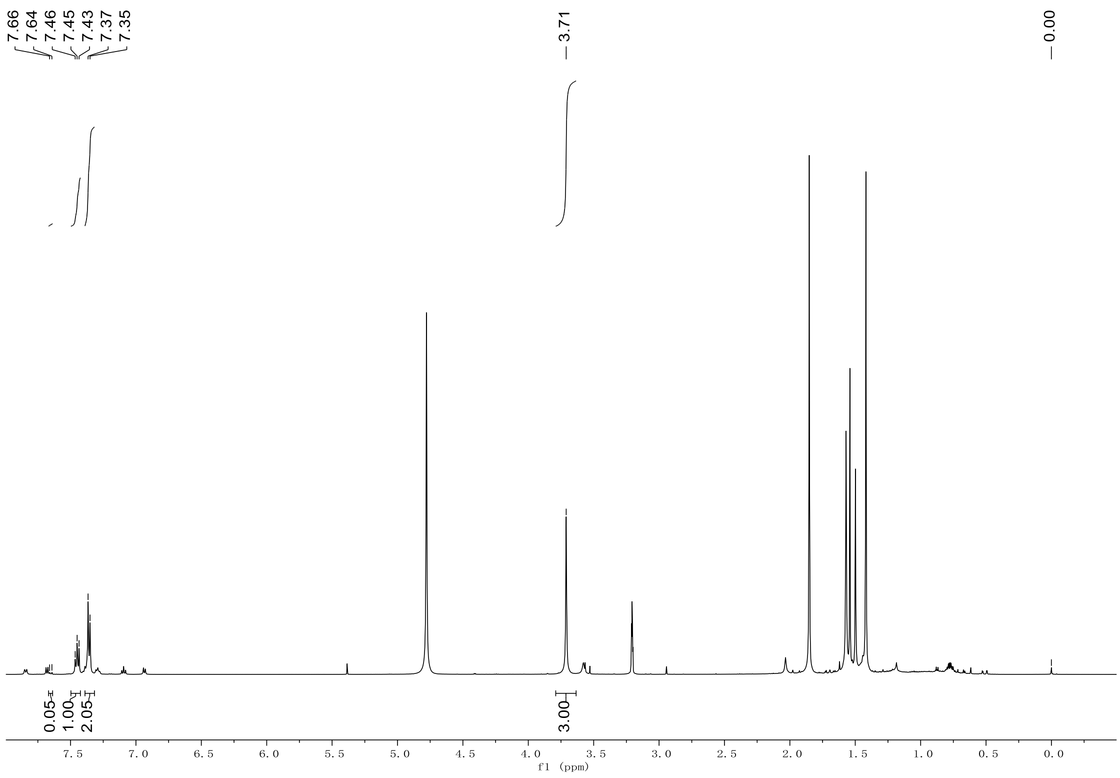

(3) Reaction of 2 in the absence of S1. (GFY-10-40)

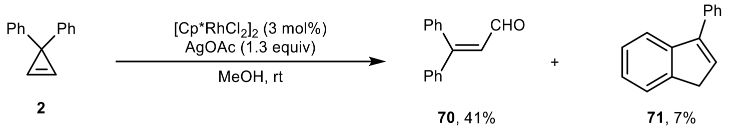

To a 10 mL of Schlenk tube equipped with a magnetic stir bar was added **2** (57.7 mg, 0.3 mmol), [Cp*$RhCl_2$]$_2$ (6.2 mg, 0.01 mmol), AgOAc (66.8 mg, 0.4 mmol), and MeOH (2 ml) successively. After being stirred at room temperature for 2 h, the crude product was filtered through a short pad of silica gel. After being concentrated in vacuo, the residue was purified by silica gel chromatography on silica gel (eluent: 0-8% of ethyl acetate in petroleum ether) to afford **70** (24.9 mg, 41%) and **71** (4.0 mg, 7%).

70:[S12] oil; ^{1}H NMR (500 MHz, $CDCl_3$) δ 9.53 (d, *J* = 8.0 Hz, 1H), 7.51 - 7.41 (m, 3H), 7.39 - 7.34 (m, 4H), 7.32 - 7.27 (m, 3H), 6.60 (d, *J* = 8.0 Hz, 1H). ^{13}C NMR (126 MHz, $CDCl_3$) δ 193.51, 162.25, 139.70, 136.67, 130.71, 130.47, 129.43, 128.65, 128.60, 128.33, 127.27.

71:[S13] oil; ^{1}H NMR (500 MHz, $CDCl_3$) δ 7.63 - 7.56 (m, 3H), 7.57 - 7.51 (m, 1H), 7.47 - 7.43 (m, 2H), 7.41 - 7.34 (m, 1H), 7.34 - 7.31 (m, 1H), 7.28 - 7.25 (m, 2H), 3.51 (d, *J* = 2.1 Hz, 2H). ^{13}C NMR (126 MHz, $CDCl_3$) δ 145.16, 144.75, 143.88, 136.12,

130.95, 128.55, 127.69, 127.55, 126.13, 124.83, 124.10, 120.29, 77.25, 77.00, 76.74, 38.17.

(4) Preparation of S3-Rh. (GFY-12-33)

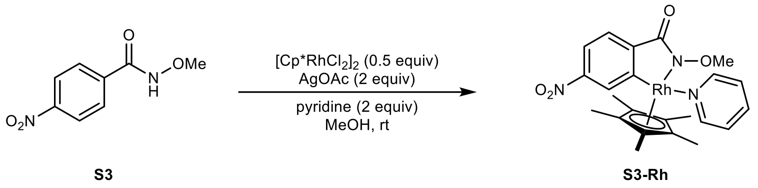

To a 25 mL of Schlenk tube equipped with a magnetic stir bar was added $[Cp^*RhCl_2]_2$ (61.8 mg, 0.1 mmol), pyridine (16 μL, 0.2 mmol) and MeOH (8 mL), after being stirred at room temperature for 2 h, AgOAc (81.0 mg, 0.48 mmol) and **S3** (57.7 mg, 0.3 mmol) was added successively. After being stirred at room temperature overnight, the crude product was purified by silica gel chromatography on silica gel (eluent: 0-5% of methanol in dichloromethane) to afford **S3-Rh**[S14] (80.8 mg, 95%) as a red solid; ^{1}H NMR (500 MHz, $CDCl_3$) δ 8.49 (d, J = 2.2 Hz, 1H), 8.44 (d, J = 4.5 Hz, 2H), 7.88 (dd, J = 8.2, 2.2 Hz, 1H), 7.68 - 7.60 (m, 2H), 7.23 - 7.16 (m, 2H), 4.04 (s, 3H), 1.64 (s, 15H); ^{13}C NMR (126 MHz, $CDCl_3$) δ 173.96, 173.92, 168.85, 168.60, 153.43, 147.95, 147.53, 137.52, 127.59, 125.90, 125.55, 119.00, 96.09, 96.04, 62.98, 9.20.

(5) Stoichiometric reaction of S3-Rh with S2 in the absence of AgOAc (GFY-12-30)

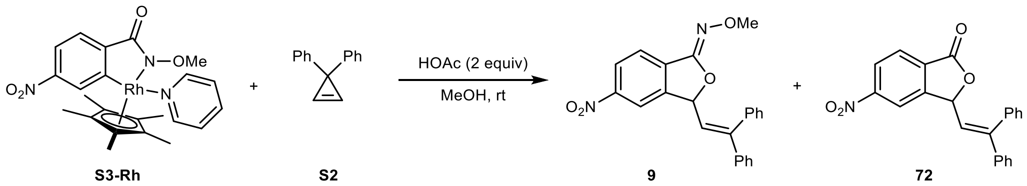

To a 10 mL of Schlenk tube equipped with a magnetic stir bar was added **S3-Rh** (51.7 mg, 0.1 mmol), **S2** (39.8 mg, 0.2 mmol), HOAc (12 μL, 0.2 mmol), and MeOH (1 ml). After being stirred at room temperature overnight, the crude product was purified by silica gel chromatography on silica gel (eluent: 0-7% of methanol in dichloromethane) to afford a mixture of **9** and **70** (29.4 mg, **9**:**70** = 1:2.5, yield of **9**: 6%, yield of **70**: 15%).

(6) Stoichiometric reaction of S3-Rh with S2 in the presence of AgOAc (GFY-12-37)

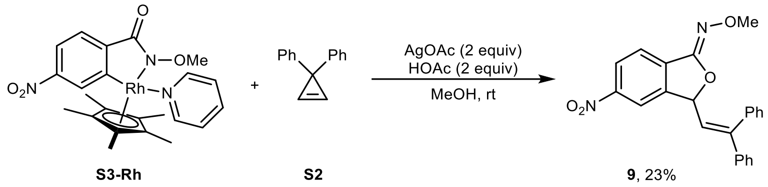

To a 10 mL of Schlenk tube equipped with a magnetic stir bar was added **S3-Rh** (51.7 mg, 0.1 mmol), **S2** (38.8 mg, 0.2 mmol), HOAc (12 μL, 0.2 mmol), AgOAc (33.8 mg, 0.2 mmol), and MeOH (1 ml). After being stirred at room temperature overnight, the crude product was purified by silica gel chromatography on silica gel (eluent: 0-7% of methanol in dichloromethane) to afford **9** (31.3 mg, 23%).

(7) Reaction of S1 with S2 leading to the formation of 73. (LSJ-3-43-2)

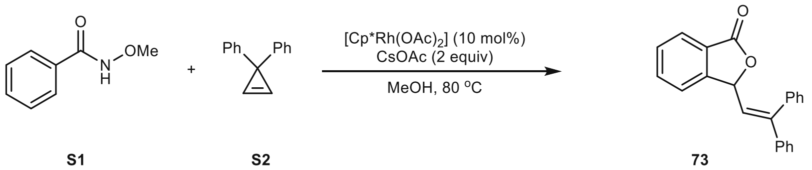

To a 25 mL of Schlenk tube equipped with a magnetic stir bar was added Cp*Rh(OAc)$_2$ (7.6 mg, 0.02 mmol), CsOAc (76.8 mg, 0.4 mmol), **S1** (31.4 mg, 0.2 mmol), **S2** (58.6 mg, 0.3 mmol) and MeOH (2 ml). After being stirred at 80 °C overnight, the crude product was purified by silica gel chromatography on silica gel (eluent: 0-7% of methanol in dichloromethane) to afford **73**[15] (13.3 mg, 20%, containing about 10% of **1**) as a white solid: ^{1}H NMR (500 MHz, CDCl$_3$) δ 7.91 (dd, J = 7.6, 2.2 Hz, 1H), 7.70 - 7.66 (m, 1H), 7.56 - 7.52 (m, 1H), 7.51 - 7.35 (m, 5H), 7.34 - 7.23 (m, 6H), 5.97 (dd, J = 9.8, 2.2 Hz, 1H), 5.89 (dd, J = 9.3, 1.9 Hz, 1H); ^{13}C NMR (126 MHz, CDCl$_3$) δ 170.50, 149.51, 149.46, 140.63, 138.11, 134.13, 129.94, 129.28, 128.65, 128.51, 128.32, 128.28, 127.78, 126.00, 125.67, 122.62, 122.45, 79.41.

2.7 Computational Mechanistic Studies

(1) Computational details:

Density functional theory (DFT) calculations were performed by using Gaussian 09 quantum chemical package.[S16] Unless otherwise specified, geometry optimizations were performed using B3LYP functional[S17] with the Stuttgart/Dresden effective core potential (SDD)[S18] was used for Rh in conjunction with a standard 6-31G(d,p) basis set for all other atoms. This level of theory is denoted as B3LYP/6-31G(d,p) SDD or B3LYP/BS1. Frequency analysis was conducted at the same level of theory to verify the stationary points to be real minima or saddle points and to obtain the thermodynamic energy corrections. Intrinsic reaction coordinate (IRC) calculation[S19] were carried out to confirm that all transition state structures connect the corresponding reactants and products. Solvent effects in Methanol were estimated by using the SMD[S20] solvation method at the M06 level of theory.[S21] Herein, SDD was used for Rh and 6-31G(d,p) basis set was used for hydrogen atoms meanwhile the 6-311++G(d,p) basis set was used for all other atoms. This level of theory is denoted as M06 SMD/6-311++G(d,p) SDD or M06 SMD/BS2. If not noted, the energies presented in this paper are the (M06 SMD/BS2)-calculated single point energies with (B3LYP/BS1)-calculated thermodynamic corrections which were calculated under standard conditions (1 atm and 298.15 K) (denoted as Δ*G*sol(M06 SMD/6-311++G(d,p) SDD//B3LYP/6-31G(d,p) SDD) or Δ*G* for clarity). The 3D diagrams of optimized structures are illustrated using CYLView.[S22]

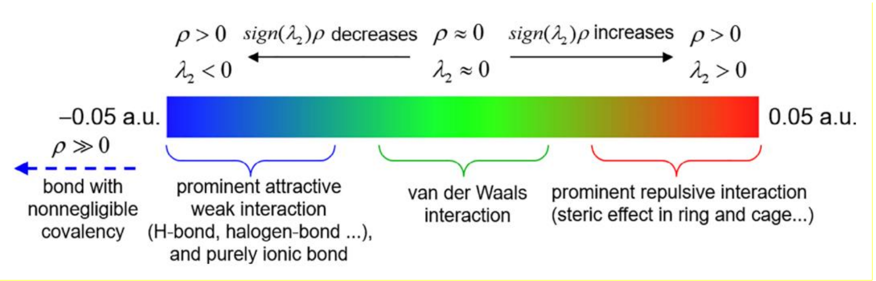

Figure S1.Common interpretation of coloring method of mapped function sign(λ2)ρ in IGMH maps

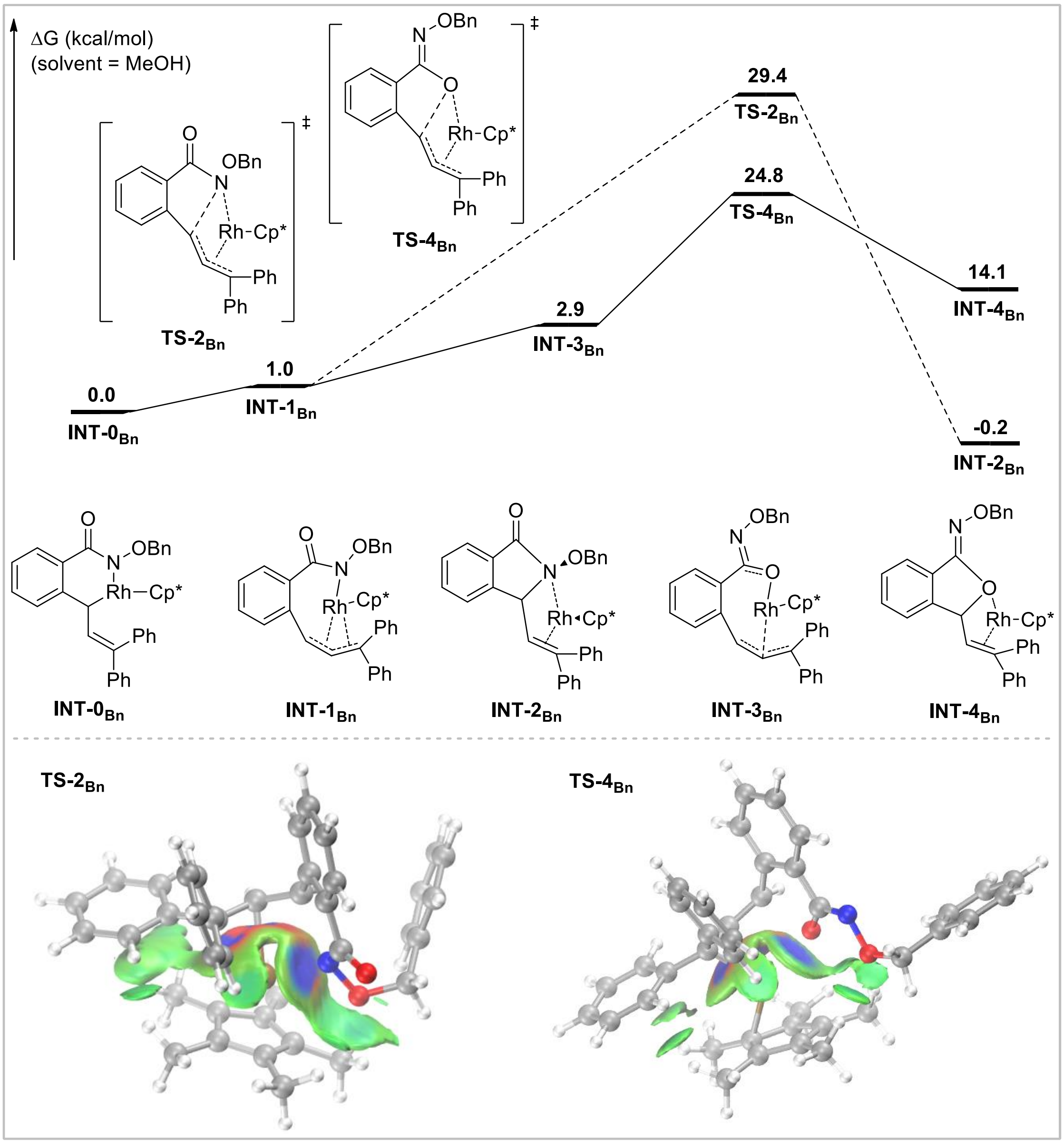

Figure S2. Computed Gibbs free energy changes from -OBn substituted substrate and weak interaction (IGMH) analysis using Multiwfn (version 3.7), isovalue = 0.005.

Table S1. Comparison of the key structures and the reaction barriers using different basis-sets.

OPT: **B3LYP**/6-31G(d)+ SDD for Rh	△G^1(**INT1**) (kcal/mol)	△G^2(**TS-4**) (kcal/mol)	|△G^1|+|△G^2| (kcal/mol)
SP: SMD(MeOH) **M06** /6-311++G(d,p)+SDD for Rh	-3.4	24.5	27.9
SP: SMD(MeOH)**TPSSTPSS** /6-311++G(d,p)+SDD for Rh	-0.8	26.8	27.6

SP: SMD(MeOH) **M11L** /6-311++G(d,p)+SDD for Rh	-2.1	22.6	24.7
SP: SMD(MeOH) **M06L** /6-311++G(d,p)+SDD for Rh	-3.2	23.3	26.5
SP: SMD(MeOH) **M062X** /6-311++G(d,p)+SDD for Rh	-2.2	26.8	29.0
SP: SMD(MeOH) **wb97xd** /6-311++G(d,p)+SDD for Rh	-4.5	27.0	31.5

Table S2. Energy, enthalpy and free energy corrections of the structures calculated at B3LYP/BS1, single point energies at the M06 SMD/BS2//B3LYP/BS1 level (in Hartree) and imaginary frequencies of the transition states.

Structures	corr. to ZPE	corr. to *E*	corr. to *H*	corr. to *G*	SP_{SMD}	Imaginary frequency
**INT-0**	0.585362	0.622003	0.622948	0.515015	-1593.01863818	–
**TS-1**	0.585142	0.620954	0.621898	0.517323	-1593.01942684	-9.11
**INT-1**	0.585870	0.622226	0.623170	0.518043	-1593.02712446	–
**INT-1'**	0.650630	0.692924	0.693868	0.576204	-1822.07153555	–
**TS-2**	0.583711	0.619585	0.620529	0.517637	-1592.97207636	-174.07
**TS-2'**	0.648013	0.689414	0.690358	0.574713	-1822.01444335	-263.68
**INT-2**	0.585598	0.621659	0.622603	0.518216	-1593.00959719	–

**INT-2'**	0.650298	0.691629	0.692573	0.576446	-1822.06518743	–
**TS-3**	0.583764	0.620031	0.620975	0.514932	-1592.98563087	-118.81
**INT-3**	0.585558	0.621898	0.622842	0.517390	-1593.02042012	–
**INT-3'**	0.650017	0.692104	0.693048	0.575391	-1822.07046070	–
**TS-4**	0.583157	0.619464	0.620408	0.515468	-1592.98011872	-198.01
**TS-4'**	0.647995	0.689728	0.690672	0.572221	-1822.01436648	-219.84
**INT-4**	0.585176	0.621689	0.622633	0.516480	-1592.99887038	–
**INT-4'**	0.648855	0.691482	0.692427	0.570278	-1822.04577289	–
**PC-1**	0.362111	0.383758	0.384702	0.308552	-1092.50703612	–
**PC-2**	0.362496	0.384095	0.385039	0.309632	-1092.53169836	–
**HOAc**	0.062071	0.066602	0.067547	0.034989	-229.041981773	–
**AgOAc**	0.050630	0.056762	0.057706	0.018490	-375.459440779	–
**Cp*Rh(OAc)$_2$ + 2Ag**	0.326508	0.355834	0.356778	0.261855	-1251.43067945	–
**INT-0$_{Bn}$**	0.666709	0.707776	0.708720	0.589387	-1823.95171115	–
**INT-1$_{Bn}$**	0.667674	0.708328	0.709273	0.593619	-1823.95432126	–

**INT-3$_{Bn}$**	0.667631	0.707994	0.708938	0.593225	-1823.95085975	–
**TS-4$_{Bn}$**	0.664678	0.705284	0.706228	0.589374	-1823.91215871	-199.05
**TS-2$_{Bn}$**	0.665271	0.705525	0.706469	0.592215	-1823.90771590	-143.68
**INT-4$_{Bn}$**	0.666767	0.707521	0.708466	0.590289	-1823.93015799	–
**INT-2$_{Bn}$**	0.668002	0.708490	0.709434	0.593507	-1823.95619738	–

Cartesian coordinates of the optimized structures

INT-0

C	-2.24198300	1.85060900	1.65316300
C	-1.83445400	0.73117600	0.88372300
C	-0.80682300	1.17612600	-0.06191300
C	-1.37364400	2.95949700	1.28987800
C	-0.56066500	2.54002000	0.14858700
C	-2.47061700	-0.62799600	0.85968000
H	-3.02484200	-0.83380800	1.78005500
H	-3.18316700	-0.70507400	0.02612500
H	-1.73040300	-1.42393300	0.73081200
C	-0.15851600	0.29460000	-1.08582000
H	0.10345400	-0.68218900	-0.66586100
H	-0.83876500	0.11314600	-1.93006800
H	0.75409600	0.74618300	-1.48328200
C	0.37450500	3.40290900	-0.64624600
H	1.34993500	2.92438600	-0.77154200
H	-0.03787000	3.60581100	-1.64366600

H	0.54287200	4.36625800	-0.16008000
C	-1.52544600	4.36821600	1.78251200
H	-2.28281200	4.90106200	1.18914000
H	-1.84245300	4.39425400	2.82847800
H	-0.58912400	4.92616600	1.70562000
C	-3.38686800	1.91719400	2.62030200
H	-3.18321600	2.60851300	3.44349100
H	-4.29823600	2.26526500	2.11467800
H	-3.61037600	0.93862100	3.05593000
Rh	-0.11830900	1.45025900	2.20678100
C	0.24565200	1.63664300	7.73392800
C	-0.33334000	1.14964300	6.56639700
C	0.29501400	1.27580000	5.31269400
C	1.53304900	1.94576500	5.25558700
C	2.11608700	2.42247600	6.44174100
C	1.48685200	2.27444200	7.67184200
H	-0.26707500	1.51693900	8.68523300
H	-1.29393500	0.64080900	6.61703000
H	3.07981300	2.91215500	6.35853900
H	1.95588300	2.65792500	8.57417400
C	2.32747600	2.23194900	4.01100100
O	3.50680700	2.59256400	4.10273200
N	1.67128700	2.12648200	2.80949500
O	2.47506200	2.49108100	1.69930800
C	2.82930800	3.87820300	1.69199900
H	3.56420600	4.09707800	2.46767500
H	3.25246300	4.05799500	0.69915200
H	1.93916200	4.50673100	1.83014600
C	-0.42876200	0.68921200	4.14103000

H	-1.50388700	0.79674000	4.30961900
C	-0.18584700	-0.69679300	3.66899400
H	-1.05217100	-1.09000600	3.14376800
C	0.85653100	-1.57894400	3.76339300
C	2.15867800	-1.31104400	4.45126900
C	2.33655500	-1.74545000	5.77516900
C	3.24266200	-0.72555100	3.78287000
C	3.56086000	-1.57764200	6.42011000
H	1.50579600	-2.21157000	6.29817400
C	4.46963000	-0.55890500	4.42954800
H	3.12040500	-0.37720200	2.76175800
C	4.63228800	-0.98487800	5.74723700
H	3.67795800	-1.90933000	7.44872000
H	5.28859100	-0.07757100	3.90404300
H	5.58587300	-0.84972800	6.25084700
C	0.69596600	-2.94798900	3.19640600
C	1.80996700	-3.65733800	2.70403200
C	-0.55814000	-3.59233300	3.14104400
C	1.66988500	-4.92657000	2.14500800
H	2.79286200	-3.20078800	2.75423500
C	-0.69666700	-4.86328000	2.58706100
H	-1.42919700	-3.10818300	3.57249600
C	0.41604400	-5.53713100	2.07813400
H	2.54766700	-5.44174400	1.76318000
H	-1.67534400	-5.33663400	2.57047900
H	0.30905800	-6.53029700	1.65031200

TS-1

C	-2.23346200	1.87348900	1.68841500

C	-1.88542500	0.70524200	0.96083300
C	-0.83777900	1.05988300	0.00124300
C	-1.31142700	2.92219200	1.28419400
C	-0.51957200	2.41621000	0.16370600
C	-2.58999200	-0.61994900	0.99136500
H	-3.14212400	-0.76529800	1.92483000
H	-3.31639100	-0.69016300	0.16932400
H	-1.89214800	-1.45635400	0.88210000
C	-0.23694800	0.11069800	-0.99122800
H	-0.03370800	-0.86637100	-0.54046000
H	-0.92187000	-0.05674100	-1.83466900
H	0.70246700	0.49649400	-1.39601800
C	0.45431100	3.20011900	-0.66555000
H	1.40884800	2.67550300	-0.76438900
H	0.05318100	3.37327500	-1.67311700
H	0.66170900	4.17684200	-0.22360700
C	-1.39280500	4.35589900	1.71853700
H	-2.11147500	4.90539600	1.09307900
H	-1.72196400	4.44115800	2.75770500
H	-0.42619700	4.85895800	1.63543400
C	-3.36988700	2.03412700	2.65450100
H	-3.12828600	2.74783900	3.44770000
H	-4.26605500	2.40461700	2.13765500
H	-3.63813100	1.08602300	3.13065100
Rh	-0.13306400	1.38060700	2.25812300
C	0.19037100	1.73321900	7.78304600
C	-0.38374000	1.21867500	6.62506400
C	0.25450900	1.30585900	5.37256100
C	1.49696200	1.96741000	5.30486200

C	2.07344800	2.47318900	6.48190400
C	1.43555300	2.36217000	7.71152100
H	-0.32952400	1.64249600	8.73365100
H	-1.34855500	0.71860400	6.68268200
H	3.04049100	2.95483600	6.39101300
H	1.90085300	2.76789200	8.60607100
C	2.30713100	2.21043300	4.05973600
O	3.48706200	2.57159100	4.15739700
N	1.66600600	2.06286400	2.85891100
O	2.48991200	2.35264500	1.74110800
C	2.88441100	3.72700200	1.67003200
H	3.62243000	3.95988200	2.43844700
H	3.31425700	3.84804000	0.67103200
H	2.01325600	4.38744800	1.77625100
C	-0.47084500	0.69716900	4.21557800
H	-1.54608700	0.80299600	4.38130100
C	-0.21701800	-0.66939900	3.70685500
H	-1.09440500	-1.07192300	3.20885200
C	0.85900700	-1.52199900	3.71947400
C	2.15924000	-1.25089300	4.40919400
C	2.30166300	-1.62441700	5.75568400
C	3.26947700	-0.72442800	3.73401800
C	3.52026900	-1.46352600	6.41325200
H	1.44856500	-2.03929500	6.28558900
C	4.48890900	-0.56149500	4.39507300
H	3.17204600	-0.41143000	2.69887400
C	4.61874700	-0.93308600	5.73284500
H	3.61058300	-1.75058400	7.45779300
H	5.32933100	-0.12479500	3.86438700

H	5.56749800	-0.80109000	6.24622000
C	0.72292000	-2.86400600	3.08627400
C	1.84724100	-3.52726200	2.55387000
C	-0.51859900	-3.53162900	3.00505400
C	1.72852300	-4.76910100	1.93202800
H	2.82225500	-3.05759500	2.62378700
C	-0.63515200	-4.77600500	2.38967900
H	-1.39761400	-3.09088000	3.46529400
C	0.48702600	-5.40086300	1.84047600
H	2.61438800	-5.24667400	1.52121200
H	-1.60427400	-5.26765600	2.35618100
H	0.39697200	-6.37308200	1.36359300

INT-1

C	-2.16318400	1.74143000	1.78754500
C	-1.93903300	0.44936100	1.20719900
C	-0.83969000	0.56037200	0.26602000
C	-1.12876700	2.61638500	1.31430600
C	-0.33898300	1.87774600	0.33292300
C	-2.82314600	-0.75680300	1.34745500
H	-3.35695100	-0.76421600	2.30336700
H	-3.58263400	-0.77576200	0.55326900
H	-2.25001500	-1.68617400	1.27480200
C	-0.37486600	-0.52477800	-0.65742700
H	-0.27014800	-1.48676300	-0.14599000
H	-1.09255500	-0.66636600	-1.47828300
H	0.59257600	-0.27828600	-1.10336300
C	0.72973000	2.44668100	-0.55272800
H	1.62546400	1.82021500	-0.55621000

H	0.36330300	2.52700600	-1.58514400
H	1.02856800	3.44553300	-0.23102500
C	-1.02327300	4.08724400	1.59263100
H	-1.56767100	4.66930000	0.83535300
H	-1.44108700	4.33948300	2.57113100
H	0.01714100	4.42073200	1.58821200
C	-3.30191600	2.12462200	2.68729200
H	-3.01955800	2.91955700	3.38346200
H	-4.15291000	2.49020500	2.09580900
H	-3.66102100	1.27516900	3.27684200
Rh	-0.13127300	0.99539900	2.50320000
C	-0.01053800	2.19934000	7.97016200
C	-0.56195500	1.54962700	6.87117300
C	0.11364700	1.46145700	5.63642800
C	1.36583300	2.09629900	5.51348600
C	1.91416700	2.74243800	6.63370500
C	1.24521900	2.79814800	7.85055700
H	-0.55709700	2.23764700	8.90907800
H	-1.53744900	1.07676300	6.96563200
H	2.88997900	3.19706500	6.50593000
H	1.69436900	3.31177200	8.69663500
C	2.22896400	2.16840900	4.27524500
O	3.42042700	2.49341900	4.39616700
N	1.61394100	1.96205100	3.08173100
O	2.51939100	1.96758500	1.97896300
C	3.06752800	3.26108900	1.71720900
H	3.79277100	3.53617300	2.48439100
H	3.54928700	3.17549300	0.73763100
H	2.27592600	4.02072500	1.66926300

C	-0.62095300	0.71613500	4.57554800
H	-1.69352300	0.85987500	4.71071400
C	-0.37503100	-0.57647600	3.97291300
H	-1.28234600	-1.08962800	3.66511500
C	0.83437500	-1.13071700	3.50896900
C	2.13290400	-0.98379300	4.24409400
C	2.14078600	-1.36152500	5.59933700
C	3.35065100	-0.63134400	3.64396400
C	3.32918000	-1.39968000	6.32732400
H	1.20639300	-1.63964300	6.07879800
C	4.53645500	-0.66295500	4.37576000
H	3.36455300	-0.25377000	2.62833900
C	4.53455600	-1.05608000	5.71440100
H	3.30931300	-1.69520100	7.37309100
H	5.46252400	-0.35340400	3.90015400
H	5.46224200	-1.07705400	6.28006400
C	0.74287300	-2.34014600	2.62300100
C	1.69223000	-2.58110000	1.61249700
C	-0.26792500	-3.30373600	2.80645000
C	1.62243800	-3.71979100	0.81101200
H	2.48877100	-1.86511200	1.44782800
C	-0.33669500	-4.44549500	2.00611600
H	-0.98880400	-3.18354800	3.60982400
C	0.60619600	-4.65913800	1.00010000
H	2.36849000	-3.87258900	0.03542800
H	-1.12146100	-5.17641300	2.18387400
H	0.55660300	-5.54946100	0.37933400

INT-1'

C	-3.64102200	0.70630800	1.40111100
C	-2.84806100	0.75883100	2.59179800
C	-2.05505700	-0.44869100	2.68566400
C	-3.28167500	-0.50503000	0.70672300
C	-2.31287200	-1.21267400	1.50746700
C	-2.92932800	1.82161400	3.64590500
H	-3.09083500	2.81044500	3.20782800
H	-3.76738100	1.61467600	4.32590000
H	-2.01470700	1.85990300	4.24092900
C	-1.28964700	-0.88802400	3.89641900
H	-0.76697700	-0.04975900	4.35983800
H	-1.98033300	-1.31996300	4.63572400
H	-0.53929300	-1.64618400	3.66283400
C	-1.76481400	-2.57427800	1.20160200
H	-0.76142600	-2.70008700	1.61469500
H	-2.40431100	-3.35565700	1.63564400
H	-1.71439300	-2.75876300	0.12401500
C	-3.97821800	-1.06353400	-0.49793400
H	-4.78479200	-1.73868900	-0.17798400
H	-4.42350900	-0.28080800	-1.11424100
H	-3.30352600	-1.64182000	-1.13518100
C	-4.73375900	1.66163400	1.02373200
H	-4.85450300	1.73218800	-0.06020200
H	-5.69655600	1.33359600	1.44158800
H	-4.53735400	2.66707600	1.40826700
Rh	-1.42281600	0.82236600	0.83664800
C	3.27225200	3.15301400	-0.11211300
C	2.08361000	2.45199600	-0.28746500
C	1.88165800	1.16278000	0.24007300

C	2.95590000	0.60167900	0.99006800
C	4.16081400	1.30447100	1.13008400
C	4.32825500	2.57641400	0.59358200
H	3.37123500	4.14544500	-0.54403600
H	1.29660900	2.91090700	-0.86050800
H	4.96386100	0.82362700	1.67798800
H	5.26772000	3.10649300	0.72373800
C	2.97209300	-0.72354400	1.71092600
O	3.97581300	-1.42421700	1.75616100
N	1.78193900	-1.06882500	2.32442900
O	1.87361600	-2.04359200	3.34122400
C	1.97986700	-3.36053700	2.79942400
H	2.93321100	-3.49399000	2.28356000
H	1.92376100	-4.02267100	3.66783100
H	1.14886500	-3.57826400	2.11596100
C	0.68412500	0.35674900	-0.08782700
H	0.84653300	-0.69566300	0.12124900
C	-0.37023200	0.52322900	-1.03903000
H	-0.73861600	-0.43491100	-1.39588700
C	-1.22077800	1.61841800	-1.39443800
C	-0.84865800	3.07428800	-1.56394100
C	-0.13786400	3.42620300	-2.72741400
C	-1.30325900	4.10124300	-0.72776800
C	0.14580900	4.75869000	-3.02197500
H	0.19909000	2.64213800	-3.40086100
C	-1.02602500	5.43778000	-1.02780700
H	-1.84207800	3.84756600	0.17551800
C	-0.29849400	5.77276100	-2.16963100
H	0.70683400	5.00394200	-3.92003500

H	-1.38005900	6.21898000	-0.35988900
H	-0.08257700	6.81326700	-2.39728900
C	-2.35871000	1.28663400	-2.33089200
C	-3.50422200	2.10588300	-2.37440300
C	-2.29149700	0.22609100	-3.25366200
C	-4.54999000	1.84820600	-3.25843100
H	-3.56710300	2.96180000	-1.71038000
C	-3.33479900	-0.02882000	-4.14654900
H	-1.40140000	-0.39330200	-3.30791800
C	-4.47553200	0.77289100	-4.14865200
H	-5.42199400	2.49730400	-3.25933400
H	-3.24360400	-0.85062900	-4.85222600
H	-5.28584600	0.57698300	-4.84530900
H	1.20804500	-0.28427300	2.66344100
C	0.82694700	3.84746600	3.07309100
H	0.92113500	3.86111300	4.16235300
H	1.82806800	3.97743600	2.64614300
H	0.19705300	4.67254900	2.73264000
C	0.28462600	2.49683700	2.61584700
O	0.62576200	1.46498300	3.21610400
O	-0.49533300	2.56791800	1.58363700

TS-2

C	-3.07508100	-1.82179200	-1.24777300
C	-3.21307900	-0.37269600	-1.19087600
C	-2.92977900	0.05098000	0.16550800
C	-2.59971900	-2.26164700	-0.00656500
C	-2.44441600	-1.09225200	0.86123800
C	-3.94543700	0.43328300	-2.22374700

H	-3.65948700	0.15264800	-3.24159800
H	-5.02843400	0.26504700	-2.12878300
H	-3.76494700	1.50391500	-2.11584900
C	-3.21286700	1.40423700	0.74827600
H	-3.10592400	2.19751500	0.00418300
H	-4.24341400	1.44981700	1.13002900
H	-2.54464900	1.63476800	1.58341400
C	-2.02584900	-1.15704900	2.30177900
H	-1.80561200	-0.16266800	2.69993500
H	-2.82460700	-1.59129600	2.92050200
H	-1.13100400	-1.77310500	2.42218800
C	-2.40538700	-3.68364700	0.42482900
H	-2.06774600	-4.32028000	-0.39949700
H	-1.67071300	-3.75624700	1.22757400
H	-3.35203700	-4.10245700	0.79658900
C	-3.42376200	-2.67223100	-2.43313300
H	-3.16749700	-2.17787800	-3.37606900
H	-2.90686600	-3.63638900	-2.40570900
H	-4.50329900	-2.88023700	-2.46526600
Rh	-1.01737800	-0.58885300	-0.84081200
C	4.72408600	0.49921200	-2.45057100
C	3.45141600	0.04554700	-2.79598300
C	2.60538700	-0.47400800	-1.80676300
C	3.03958000	-0.51632100	-0.48008800
C	4.30918000	-0.04650700	-0.13303400
C	5.15108200	0.46196900	-1.11789700
H	5.38539700	0.88265800	-3.22322600
H	3.11868300	0.08389100	-3.83013300
H	4.61511800	-0.11108500	0.90631700

H	6.14202100	0.82251100	-0.85538400
C	2.16702400	-1.20483100	0.50844900
O	2.58236400	-1.57255100	1.60915400
N	0.94002900	-1.53191800	-0.03819700
O	0.39591100	-2.69787300	0.55436300
C	1.19329800	-3.85560400	0.30893800
H	2.16657600	-3.78319800	0.80273500
H	0.62750500	-4.69027900	0.73231600
H	1.32520000	-4.01318200	-0.77047000
C	1.25511300	-0.97856300	-2.19280200
H	1.21688600	-1.97622400	-2.62578500
C	0.20630600	-0.10243100	-2.55328000
H	-0.43095400	-0.43747800	-3.36672200
C	-0.11651400	1.15086600	-1.88451400
C	0.87199300	1.93342300	-1.03667600
C	1.77793200	2.80241100	-1.67062700
C	0.83861200	1.92858200	0.36381800
C	2.63754700	3.61210200	-0.93149600
H	1.80530900	2.84103200	-2.75633700
C	1.69327500	2.74600000	1.10934400
H	0.14246700	1.26529500	0.86700900
C	2.59733700	3.58903900	0.46548600
H	3.33684700	4.26606900	-1.44649800
H	1.65040200	2.71641500	2.19503300
H	3.26404400	4.22394000	1.04325700
C	-1.05539700	2.08345900	-2.60363700
C	-1.56720100	3.21823200	-1.94060400
C	-1.40314400	1.92642000	-3.95893100
C	-2.41332100	4.12024300	-2.58247600

H	-1.28120800	3.39751700	-0.90959700
C	-2.24625800	2.83196600	-4.60594000
H	-0.99157400	1.10446400	-4.53569800
C	-2.76564000	3.93058300	-3.92158900
H	-2.79037000	4.98114700	-2.03595400
H	-2.48509800	2.68101200	-5.65590500
H	-3.41878900	4.63712500	-4.42632100

TS-2'

C	-4.15044300	0.34983700	1.19146800
C	-3.66567300	0.76505000	2.46045600
C	-2.70952700	-0.23243800	2.94742300
C	-3.38229500	-0.80622300	0.80334300
C	-2.56756500	-1.21189700	1.94544000
C	-4.12563300	1.94503600	3.26132200
H	-4.84187200	2.55549600	2.70470100
H	-4.60923300	1.62373100	4.19363200
H	-3.27330100	2.58226100	3.51960000
C	-2.06953400	-0.22098500	4.30506400
H	-1.94059000	0.80210000	4.66900600
H	-2.68729600	-0.75528300	5.04168900
H	-1.08129900	-0.68962400	4.28853600
C	-1.78467900	-2.48680300	2.04397200
H	-0.99081500	-2.40619500	2.78933700
H	-2.43953000	-3.32084600	2.33628100
H	-1.32613900	-2.76026100	1.08805400
C	-3.64308800	-1.65064300	-0.41136900
H	-4.47120600	-2.35090200	-0.22905000
H	-3.90679600	-1.04134600	-1.27974600

H	-2.76875000	-2.25146600	-0.68309900
C	-5.30015300	0.93806200	0.43258900
H	-5.15639100	0.86197900	-0.64747400
H	-6.23227700	0.40794700	0.67853500
H	-5.45157300	1.99426100	0.67492300
Rh	-1.83044400	0.80761500	1.09124200
C	3.53736900	2.11903200	-2.23366500
C	2.34016000	1.49599000	-1.88611200
C	2.23062000	0.85528000	-0.64603700
C	3.33616500	0.86016700	0.22317000
C	4.53815800	1.48629700	-0.12556200
C	4.63392200	2.12063000	-1.35863000
H	3.61878100	2.61279500	-3.19792200
H	1.49739300	1.50859400	-2.56830300
H	5.36860000	1.46538100	0.57333200
H	5.55596900	2.61711000	-1.64668000
C	3.14626000	0.12696000	1.48255500
O	4.00071900	-0.32139900	2.21995200
N	1.72330800	-0.08220500	1.65544000
O	1.37348200	-1.13311300	2.51436600
C	1.86438400	-2.40788600	2.08958200
H	2.95640800	-2.43551800	2.08414300
H	1.48234000	-3.10676200	2.83589700
H	1.46518700	-2.67965200	1.10447700
C	1.04431900	0.04020500	-0.24565100
H	1.24273100	-1.02406000	-0.35609200
C	-0.34032700	0.33244000	-0.47839300
H	-0.84738600	-0.57750500	-0.79117700
C	-1.08537400	1.55146500	-0.85089600

C	-0.44682500	2.91719100	-0.88713500
C	-0.75535500	3.84580000	-1.90574500
C	0.52937700	3.32182600	0.04461300
C	-0.15156200	5.10293000	-1.96675500
H	-1.46897800	3.58694400	-2.67726300
C	1.13635400	4.57337000	-0.01507000
H	0.82885200	2.65185600	0.83376700
C	0.79654100	5.48260300	-1.01855300
H	-0.42499300	5.78260200	-2.77046900
H	1.88426000	4.83314500	0.73023300
H	1.26637800	6.46171100	-1.06353000
C	-2.10430300	1.32525300	-1.94231400
C	-3.28513000	2.09058700	-1.99666400
C	-1.88913700	0.40280600	-2.98146400
C	-4.20655500	1.93584000	-3.03057900
H	-3.47057700	2.81536500	-1.20925600
C	-2.81822300	0.23242100	-4.01247900
H	-0.97263300	-0.18044100	-2.99648900
C	-3.98268400	0.99794300	-4.04440200
H	-5.10674300	2.54573200	-3.04226300
H	-2.61900700	-0.49145400	-4.79911200
H	-4.70324200	0.87359000	-4.84828200
H	1.25393900	0.78979500	2.13087600
C	-0.15456300	3.85336100	3.97374900
H	0.59687800	3.74839300	4.75937400
H	0.06639700	4.75760200	3.39367300
H	-1.14890600	3.97956800	4.41105000
C	-0.12147900	2.65494800	3.03130200
O	0.93482900	1.96491800	3.01182100

O	-1.14672100	2.49569800	2.30088800

INT-2

C	-3.15987300	-1.76588700	-1.12370100
C	-3.34526600	-0.32053900	-1.08306000
C	-3.12192600	0.12864400	0.28683100
C	-2.66045700	-2.15476900	0.12922600
C	-2.61594400	-0.97308100	1.00186800
C	-4.06427900	0.46899800	-2.13783200
H	-3.79118800	0.14300200	-3.14545300
H	-5.15232900	0.34253300	-2.03252800
H	-3.84521300	1.53671900	-2.06911700
C	-3.42031200	1.50384100	0.80986200
H	-3.19039400	2.27686100	0.07010300
H	-4.48548400	1.60592000	1.06531600
H	-2.84606900	1.72769600	1.71459500
C	-2.20320500	-1.01454500	2.44645900
H	-2.08539300	-0.00832200	2.85976200
H	-2.95178700	-1.53680200	3.05976500
H	-1.25074000	-1.53936600	2.57840800
C	-2.33390700	-3.54571900	0.58715600
H	-2.17893800	-4.22202000	-0.25865300
H	-1.42483500	-3.56349000	1.19465400
H	-3.15092700	-3.95778100	1.19766700
C	-3.48097500	-2.64790100	-2.29542200
H	-3.24261600	-2.15839400	-3.24555000
H	-2.92254100	-3.58861000	-2.25799100
H	-4.55114000	-2.90242800	-2.32390200
Rh	-1.17043200	-0.54000100	-0.78895600

C	4.79741100	0.38834700	-2.39321400
C	3.54354200	-0.09579800	-2.77570800
C	2.67723800	-0.54461300	-1.78216800
C	3.06318900	-0.49455100	-0.44007500
C	4.30688400	0.00433200	-0.04781300
C	5.17693900	0.44446700	-1.04263000
H	5.49400600	0.72703100	-3.15575800
H	3.25847000	-0.12455600	-3.82409200
H	4.57608100	0.03369200	1.00353300
H	6.15608800	0.83188800	-0.77618200
C	2.02744900	-1.10909100	0.40814300
O	2.03860800	-1.35052700	1.59431000
N	0.93877000	-1.50296500	-0.48525300
O	0.58273200	-2.87566400	-0.31591100
C	1.68859900	-3.78371500	-0.40947000
H	2.39278100	-3.65223300	0.41824800
H	1.22683300	-4.77120600	-0.34182600
H	2.21180500	-3.69594000	-1.37026400
C	1.28189600	-1.12362600	-1.90710800
H	1.28235700	-2.03961900	-2.50586300
C	0.14018100	-0.23365700	-2.38942200
H	-0.20599700	-0.47326000	-3.39031500
C	-0.14363800	1.07581600	-1.82885700
C	0.83013000	1.86227800	-0.96579600
C	1.80203400	2.66795100	-1.58782500
C	0.74761600	1.90510900	0.43290800
C	2.67686800	3.45407600	-0.84065200
H	1.86562000	2.67675100	-2.67276600
C	1.61863900	2.69766900	1.18766700

H	-0.01128200	1.30401300	0.92537100
C	2.58990300	3.47174800	0.55491200
H	3.42516600	4.05853600	-1.34771100
H	1.53199900	2.70710300	2.27133300
H	3.26859500	4.08840300	1.13875800
C	-1.03373200	2.00400100	-2.61763500
C	-1.51919300	3.18515600	-2.01756600
C	-1.38865000	1.78558300	-3.96246500
C	-2.33113100	4.08096600	-2.71084300
H	-1.24509400	3.40346100	-0.99041800
C	-2.19791000	2.68357200	-4.66074800
H	-1.01952000	0.91230800	-4.48975800
C	-2.68078000	3.83629000	-4.04119400
H	-2.68605200	4.97823800	-2.20939800
H	-2.44257900	2.48117400	-5.70097300
H	-3.30823300	4.53606700	-4.58656700

INT-2'

C	-4.17112200	0.30517500	1.19934900
C	-3.76014000	0.59373700	2.50423300
C	-2.73338900	-0.38363000	2.90390400
C	-3.30733100	-0.76615600	0.70586600
C	-2.52375000	-1.26843000	1.82946100
C	-4.30209000	1.65339200	3.41833400
H	-4.93854600	2.36206800	2.88116600
H	-4.90097500	1.21538800	4.22950100
H	-3.49467400	2.22765900	3.88658600
C	-2.18902700	-0.50975600	4.29836400
H	-2.00489200	0.46603800	4.75880500

H	-2.89716000	-1.04151500	4.95127400
H	-1.24650500	-1.06535500	4.31940700
C	-1.70942200	-2.52955000	1.83166900
H	-0.94670000	-2.51484600	2.61556800
H	-2.34434800	-3.41082700	2.00676000
H	-1.19908300	-2.68533800	0.87549200
C	-3.54650900	-1.51909800	-0.57234000
H	-4.36386100	-2.24396100	-0.44313300
H	-3.82002900	-0.85201900	-1.39327700
H	-2.66174300	-2.08297700	-0.88480700
C	-5.30769200	0.91915500	0.44014300
H	-5.05748100	1.07455900	-0.61285500
H	-6.19398900	0.26778400	0.46965600
H	-5.60104600	1.88680800	0.85951200
Rh	-1.78428300	0.76883300	1.10675200
C	3.31497800	1.24471600	-3.03412100
C	2.18267300	0.70413700	-2.41665500
C	2.19244800	0.55401600	-1.03308700
C	3.31123500	0.95512400	-0.29575200
C	4.44317300	1.49574700	-0.90102600
C	4.43677300	1.63543800	-2.28847400
H	3.32384800	1.36706800	-4.11393800
H	1.31741500	0.42147000	-3.00801700
H	5.29447500	1.79607100	-0.29734900
H	5.30061100	2.05417900	-2.79703300
C	3.07238300	0.70394200	1.14248800
O	3.82894100	0.81035300	2.09594700
N	1.74079100	0.28959700	1.19965900
O	1.26546600	-0.35380600	2.33745900

C	1.99994700	-1.55116700	2.67041800
H	3.03666900	-1.30658600	2.90773400
H	1.48999400	-1.95495000	3.54723800
H	1.94317900	-2.26964500	1.84556200
C	1.13558800	-0.03956900	-0.10973800
H	1.18612600	-1.13547000	-0.20759500
C	-0.34305400	0.30871000	-0.36131600
H	-0.78023800	-0.55823000	-0.85468900
C	-0.96293800	1.54621900	-0.81846600
C	-0.27031800	2.88176200	-0.77136900
C	-0.52501600	3.88117500	-1.73602500
C	0.71191900	3.18788500	0.19205000
C	0.13550500	5.11009900	-1.71802500
H	-1.23968500	3.69844400	-2.52844800
C	1.37757100	4.41091900	0.20935500
H	0.95665200	2.45350900	0.94362700
C	1.09078700	5.39068600	-0.74255200
H	-0.09682800	5.84660800	-2.48376400
H	2.13137700	4.59448700	0.97188400
H	1.60806400	6.34651800	-0.72928800
C	-1.98604200	1.41253400	-1.91869900
C	-3.13041200	2.23357500	-1.95328600
C	-1.81523100	0.51068600	-2.98341800
C	-4.05409900	2.15453100	-2.99424600
H	-3.28773500	2.94055700	-1.14352100
C	-2.74516800	0.41697000	-4.02303000
H	-0.93741700	-0.12876400	-3.00563700
C	-3.87025900	1.23986800	-4.03679700
H	-4.92511200	2.80560600	-2.98881000

H	-2.57872400	-0.29386300	-4.82900800
H	-4.59186700	1.17560800	-4.84679900
H	0.56206200	0.96960000	3.30150800
C	-0.42728600	3.89766500	4.27836100
H	-0.55506900	3.63743100	5.33355600
H	0.52295100	4.43568200	4.18613800
H	-1.24064000	4.53974400	3.93982800
C	-0.37092700	2.65165900	3.43244300
O	0.46794600	1.74334700	3.91400000
O	-1.02441500	2.52258300	2.40152300

TS-3

C	-1.82646600	-2.86942200	-0.29805000
C	-2.45662000	-1.58903900	-0.56749100
C	-2.58618200	-0.91993500	0.67550600
C	-1.83014500	-3.06994300	1.15568600
C	-2.23495500	-1.87193000	1.74703300
C	-2.97142100	-1.15795900	-1.90853300
H	-2.21541100	-1.24115200	-2.69672900
H	-3.81176200	-1.80296900	-2.20010600
H	-3.33924100	-0.12938600	-1.89513800
C	-3.17964100	0.43582400	0.91864600
H	-2.68345500	0.94183600	1.75235300
H	-3.09324800	1.08467100	0.04398100
H	-4.24544000	0.35037900	1.17482300
C	-2.30793400	-1.55340400	3.20901200
H	-3.35112900	-1.52126800	3.55294100
H	-1.77564000	-2.29628300	3.80726900
H	-1.86247500	-0.57641900	3.42433600

C	-1.44406000	-4.34473900	1.83509000
H	-2.29158400	-5.04483300	1.83022200
H	-0.60996000	-4.83145200	1.32420300
H	-1.14624000	-4.17818900	2.87177900
C	-1.56982300	-3.94069600	-1.31532700
H	-0.74106300	-4.58591700	-1.01210400
H	-2.46016900	-4.57365000	-1.44445900
H	-1.32059700	-3.51388200	-2.29089900
Rh	-0.38980000	-1.37798500	0.30760700
C	4.02627900	0.11758500	-2.59436100
C	2.63272100	0.07836800	-2.59728000
C	1.92770600	-0.29405900	-1.44411000
C	2.64835300	-0.64407800	-0.30095400
C	4.04580800	-0.60529600	-0.28997000
C	4.73578800	-0.21882600	-1.43743300
H	4.56089200	0.41391700	-3.49320200
H	2.08233900	0.35342800	-3.49406500
H	4.58717800	-0.88214100	0.61108100
H	5.82205300	-0.18691200	-1.43369500
C	1.87779300	-1.08105100	0.91715500
O	1.57864900	-0.29924900	1.84701100
N	1.51337600	-2.40931500	0.82987300
O	1.17537600	-2.90247700	2.14848700
C	1.97703900	-4.04912100	2.37716700
H	3.04509600	-3.79505200	2.41150800
H	1.66666200	-4.43895600	3.35212000
H	1.82262900	-4.81416300	1.60392700
C	0.41999400	-0.33649100	-1.41396200
H	0.05276800	-0.98167100	-2.22211600

C	-0.32850600	0.93923400	-1.47172800
H	-1.36631300	0.77290800	-1.74063600
C	-0.02526800	2.26092600	-1.31104500
C	1.28215600	2.86042900	-0.91613200
C	1.84077800	3.86849300	-1.72621700
C	1.94745100	2.51429200	0.26951200
C	3.03770900	4.48923800	-1.37783300
H	1.32860900	4.16047400	-2.63923000
C	3.14287600	3.14564600	0.62200300
H	1.54270400	1.74559000	0.91882700
C	3.69396500	4.13110300	-0.19676700
H	3.45612700	5.25606900	-2.02518300
H	3.64084800	2.85829700	1.54439300
H	4.62433600	4.61961700	0.08195200
C	-1.12117500	3.24669000	-1.57016400
C	-1.28001200	4.37208800	-0.73862700
C	-2.03083600	3.08628200	-2.63309400
C	-2.32167000	5.27612100	-0.94032200
H	-0.58208700	4.52729500	0.07844900
C	-3.07110300	3.99326700	-2.83833400
H	-1.89866600	2.25730600	-3.32306000
C	-3.22545800	5.09100800	-1.98962200
H	-2.42766600	6.12925700	-0.27489400
H	-3.75127700	3.85053100	-3.67457000
H	-4.03243100	5.80074500	-2.15134400

INT-3

C	0.80114700	-3.05470800	0.12395800
C	1.44719800	-2.93779900	1.39208200

C	2.67881400	-2.21729800	1.18843200
C	1.69330500	-2.48548400	-0.88947200
C	2.85718300	-2.01054500	-0.23925900
C	0.95458200	-3.48705900	2.69883200
H	-0.13365100	-3.59355700	2.70571000
H	1.38572200	-4.47997200	2.88786100
H	1.23470000	-2.84708600	3.54183000
C	3.72971000	-1.95963900	2.22971000
H	4.29953500	-1.05196400	2.01140400
H	3.29468100	-1.85403300	3.22883400
H	4.44305600	-2.79471200	2.27349000
C	4.07215100	-1.39940900	-0.86896200
H	4.92824900	-2.08506300	-0.80284900
H	3.90511800	-1.17738600	-1.92600200
H	4.35877300	-0.46490900	-0.37651700
C	1.36481500	-2.44339800	-2.35020000
H	1.29862500	-3.45888800	-2.76274600
H	0.39676400	-1.95133800	-2.50024400
H	2.12353500	-1.89879700	-2.91921400
C	-0.48395700	-3.76368900	-0.18410500
H	-1.04125700	-3.23981700	-0.96604500
H	-0.28245400	-4.78634700	-0.53465500
H	-1.12650100	-3.83718600	0.69824100
Rh	0.96881100	-0.87203800	0.55829000
C	-3.39439300	1.88338400	2.82752300
C	-2.11480100	1.34445000	2.90552400
C	-1.54021900	0.62957100	1.83705000
C	-2.30506900	0.43292000	0.67038900
C	-3.59986000	0.98128200	0.60462200

C	-4.14293700	1.69923600	1.66255000
H	-3.80252000	2.44002300	3.66731100
H	-1.53173500	1.47901600	3.81430000
H	-4.16816000	0.82347200	-0.30448700
H	-5.14377300	2.11491100	1.57903200
C	-1.79183900	-0.33002200	-0.51044100
O	-0.51812500	-0.35156700	-0.79400100
N	-2.71960200	-0.88608800	-1.24127900
O	-2.15247500	-1.57960400	-2.34285200
C	-3.21934000	-2.09672400	-3.11648400
H	-3.85785500	-1.29200400	-3.50412500
H	-2.75356900	-2.62964900	-3.95134100
H	-3.84082300	-2.79124300	-2.53507900
C	-0.17744500	0.06139600	2.12226600
H	-0.20434000	-0.57769400	3.00734500
C	1.09398600	0.72550700	1.99112600
H	1.84071300	0.43786300	2.72560200
C	1.52799100	1.47039700	0.86353900
C	0.59897200	2.38449800	0.11063600
C	-0.00597500	3.43043400	0.82686400
C	0.38724300	2.31847000	-1.27508100
C	-0.79934300	4.37981800	0.18264300
H	0.15020400	3.49910400	1.89972000
C	-0.40234400	3.27034100	-1.92047100
H	0.80287300	1.49358300	-1.84079100
C	-0.99787000	4.30469200	-1.19629600
H	-1.26060100	5.17625500	0.76064000
H	-0.56372100	3.19184500	-2.99239000
H	-1.61655000	5.04143300	-1.70198700

C	2.98582700	1.81190800	0.74781300
C	3.53303300	2.23109600	-0.48143700
C	3.85995700	1.77307400	1.85415000
C	4.88070800	2.56552300	-0.60488900
H	2.89254800	2.30178900	-1.35229200
C	5.20772800	2.11333500	1.73293300
H	3.48573300	1.51103700	2.83817500
C	5.73110300	2.50675000	0.50057300
H	5.26416600	2.87987200	-1.57217200
H	5.84524600	2.08465200	2.61304100
H	6.77963400	2.77527900	0.40665500

INT-3'

C	-1.49794600	-1.66194100	1.49533400
C	-1.98612100	-1.85401200	0.17066700
C	-2.84954700	-0.74033300	-0.14620800
C	-2.09859600	-0.43593600	2.01573600
C	-2.98137700	0.08843000	1.03794100
C	-1.70380200	-3.03097800	-0.71558000
H	-0.69171400	-3.41700200	-0.56993000
H	-2.41273800	-3.84347300	-0.50185300
H	-1.82047000	-2.77416700	-1.77328900
C	-3.70211400	-0.61981200	-1.37542500
H	-3.90558400	0.42215700	-1.63311400
H	-3.23484000	-1.09574400	-2.24274700
H	-4.66831000	-1.11845400	-1.21415900
C	-3.93422100	1.23167200	1.20585800
H	-4.93928400	0.85570500	1.44554500
H	-3.62948000	1.89356400	2.02191700

H	-4.01872100	1.83483300	0.29789000
C	-1.89213100	0.07558400	3.40990900
H	-2.55461300	-0.44807400	4.11301600
H	-0.86354500	-0.09086500	3.73957000
H	-2.10266500	1.14595200	3.48647100
C	-0.74857800	-2.65810100	2.32783500
H	0.01039700	-2.17764800	2.94800800
H	-1.45504100	-3.18155700	2.98879800
H	-0.24564300	-3.40147100	1.70809000
Rh	-0.80556100	0.12748200	0.23060500
C	4.28852000	1.21533500	-1.08226400
C	2.89755500	1.18024200	-1.17189500
C	2.18976100	-0.03193100	-1.14436500
C	2.94565400	-1.22702200	-0.99932700
C	4.34000300	-1.18406500	-0.93228300
C	5.01983600	0.03271800	-0.97437100
H	4.79801700	2.17502200	-1.11483600
H	2.35959300	2.10848500	-1.28670800
H	4.88365500	-2.11883800	-0.83904300
H	6.10481300	0.05507800	-0.92060200
C	2.27154600	-2.55015800	-0.86129300
O	1.52325700	-2.76802600	0.22803500
N	2.44087700	-3.41545200	-1.79861100
O	1.74749500	-4.61482900	-1.50677300
C	1.98940300	-5.52478200	-2.56441500
H	3.05884300	-5.75351900	-2.66054900
H	1.44169500	-6.43481100	-2.30223000
H	1.62255400	-5.13622300	-3.52401700
C	0.73676400	-0.15749200	-1.40009200

H	0.51387200	-1.13687800	-1.81831100
C	-0.27953900	0.78146000	-1.78087400
H	-1.02996700	0.32433000	-2.42018700
C	-0.66499500	2.05673300	-1.28353500
C	0.22912600	3.18338000	-0.82425500
C	0.91286500	3.90507300	-1.82081200
C	0.27411100	3.66354800	0.49042000
C	1.65073800	5.04590300	-1.50639900
H	0.86146000	3.56539400	-2.85237300
C	1.00669900	4.81109400	0.80423200
H	-0.23968300	3.11682100	1.26987200
C	1.70077800	5.50389600	-0.18796200
H	2.17692600	5.58014700	-2.29323000
H	1.03477100	5.16147800	1.83290300
H	2.27079700	6.39497500	0.06123800
C	-1.95996900	2.61484400	-1.81948300
C	-2.69067600	3.56526800	-1.07964500
C	-2.44113800	2.29049100	-3.10315200
C	-3.86193100	4.13217300	-1.57966500
H	-2.32837700	3.86836500	-0.10288800
C	-3.61057100	2.86210400	-3.60769500
H	-1.88149700	1.60990700	-3.73740100
C	-4.33379700	3.78063900	-2.84659700
H	-4.40328500	4.85775600	-0.97802100
H	-3.94548100	2.59691900	-4.60721500
H	-5.24149000	4.22915600	-3.24060500
H	1.58720800	-1.99661200	0.86843700
C	2.59646000	1.23806800	2.83756200
H	2.96552500	0.69728100	3.71256100

H	3.45389600	1.47789500	2.19764200
H	2.12165900	2.17578200	3.13591500
C	1.64378700	0.35527100	2.03846200
O	1.82288800	-0.87882000	2.04733900
O	0.73754400	1.00235700	1.39449200

TS-4

C	1.14697400	-3.28506800	-0.01624600
C	2.01872200	-3.12975400	1.07636400
C	3.01358100	-2.12289600	0.70589300
C	1.62803400	-2.43568800	-1.11294600
C	2.82687400	-1.79683100	-0.69377900
C	1.96195400	-3.83924300	2.39673700
H	0.96278400	-4.23483300	2.59926700
H	2.66424300	-4.68514300	2.41886000
H	2.23367400	-3.17642100	3.22500300
C	4.21921700	-1.76832600	1.52547900
H	4.63912100	-0.80360900	1.23266900
H	3.98130800	-1.71463100	2.59241700
H	5.00360800	-2.52925200	1.40172500
C	3.76233100	-0.98578700	-1.53792000
H	4.60419100	-1.60374900	-1.88184600
H	3.26074500	-0.58902700	-2.42542600
H	4.17845700	-0.13957500	-0.98450900
C	0.99428200	-2.38538400	-2.47101400
H	1.13457500	-3.33847300	-3.00103200
H	-0.08154300	-2.20026500	-2.38779600
H	1.43167700	-1.59629000	-3.08961900
C	-0.07680900	-4.14533300	-0.10924700

H	-0.93599600	-3.55883000	-0.45178600
H	0.07967200	-4.96745800	-0.82175600
H	-0.33312200	-4.58765800	0.85755000
Rh	1.10216900	-1.04150200	0.56651300
C	-3.30225200	2.90994400	2.46725400
C	-2.19420800	2.11380500	2.75674500
C	-1.86110600	1.04876200	1.91052500
C	-2.64723700	0.78654500	0.78303000
C	-3.75410100	1.58911600	0.49028000
C	-4.07967400	2.64857600	1.33324900
H	-3.55869700	3.73533300	3.12600300
H	-1.58807500	2.31842500	3.63563000
H	-4.34319600	1.36860500	-0.39371500
H	-4.94072400	3.27296400	1.11019000
C	-2.19061700	-0.38575900	-0.00076700
O	-1.19194400	-1.03091400	0.55578400
N	-2.84315000	-0.68558200	-1.08160600
O	-2.30486000	-1.83756600	-1.70747300
C	-3.14873800	-2.16822600	-2.79751700
H	-3.20574600	-1.34569800	-3.52219600
H	-2.69836400	-3.04648200	-3.26965200
H	-4.16288300	-2.41208500	-2.45592400
C	-0.72280000	0.14587800	2.25601600
H	-0.96159400	-0.61156700	3.00221400
C	0.65424700	0.42214300	2.13203300
H	1.27316800	0.00841700	2.92393600
C	1.30607700	1.10814500	1.02361200
C	0.52645600	2.02856800	0.10439800
C	0.24302300	3.33873400	0.52946800

C	0.15005200	1.66236200	-1.19387000
C	-0.41400900	4.24007900	-0.30488600
H	0.54207000	3.64847800	1.52752000
C	-0.50591700	2.56512400	-2.03604600
H	0.36665500	0.65552800	-1.53863000
C	-0.79155600	3.85592900	-1.59501400
H	-0.63130300	5.24405700	0.05104600
H	-0.79682500	2.25137300	-3.03511600
H	-1.30393700	4.55825700	-2.24739500
C	2.72123500	1.57260500	1.22586100
C	3.45415000	2.09556200	0.14002900
C	3.36162300	1.56024200	2.48055100
C	4.76308000	2.54946600	0.29298100
H	2.98010600	2.15349400	-0.83461500
C	4.67139200	2.01684400	2.63615300
H	2.82758000	1.21795800	3.36100700
C	5.38594400	2.50846300	1.54320100
H	5.29550800	2.94465500	-0.56871600
H	5.12808600	1.99966900	3.62285100
H	6.40387500	2.86829600	1.66554700

TS-4'

C	-2.03186600	-1.82934800	1.19208000
C	-2.55122000	-1.69539900	-0.10491300
C	-3.25272100	-0.40992000	-0.18422600
C	-2.40427200	-0.62689800	1.94243200
C	-3.24759400	0.18500500	1.13227500
C	-2.43197300	-2.69463600	-1.21812600
H	-1.47624200	-3.22475100	-1.17786900

H	-3.23725800	-3.44098700	-1.16057700
H	-2.50724700	-2.21432100	-2.19827300
C	-4.15609200	-0.00091900	-1.31044500
H	-4.33618900	1.07560600	-1.31772900
H	-3.74602900	-0.27241800	-2.28757100
H	-5.12805800	-0.50610500	-1.21332700
C	-4.04607700	1.37410200	1.57362600
H	-5.06895600	1.07494300	1.84629000
H	-3.60182200	1.85446600	2.45092200
H	-4.12314400	2.12595100	0.78388000
C	-2.10345200	-0.40206900	3.39494500
H	-2.85277700	-0.89952500	4.02678700
H	-1.12663900	-0.80743700	3.67330600
H	-2.10841700	0.66225300	3.64667700
C	-1.30629100	-3.02322000	1.74145600
H	-0.59699700	-2.75185700	2.52680200
H	-2.01803000	-3.73976700	2.17716400
H	-0.74122900	-3.54539400	0.96404800
Rh	-1.14887800	0.13018700	0.19931500
C	4.03816000	1.54851200	-3.16348600
C	2.67952700	1.35518200	-2.90902600
C	2.25303200	0.21553300	-2.21895600
C	3.20455800	-0.73905000	-1.81999900
C	4.56341700	-0.55030100	-2.07908700
C	4.97854200	0.60074500	-2.74663800
H	4.36190900	2.44066000	-3.69241300
H	1.95508300	2.07613000	-3.26383200
H	5.27226700	-1.30256000	-1.74907400
H	6.03528900	0.76158400	-2.94314500

C	2.60788400	-1.88925900	-1.11381400
O	1.31565700	-1.73777000	-0.86198200
N	3.36152100	-2.89396300	-0.79465800
O	2.60441700	-3.87928300	-0.11798300
C	3.49029700	-4.89889600	0.30285300
H	4.23699900	-4.51747100	1.01235300
H	2.86645000	-5.65025000	0.79629700
H	4.01099300	-5.35387500	-0.55025600
C	0.82632000	-0.18226000	-2.08836200
H	0.58079100	-1.00487200	-2.75494500
C	-0.34410800	0.58096000	-1.79177200
H	-1.18494900	0.20537500	-2.37025000
C	-0.65308800	1.83040200	-1.08449400
C	0.39192600	2.86411300	-0.67852300
C	0.84803900	3.75262300	-1.67286000
C	0.80375000	3.10673200	0.63729900
C	1.72527100	4.79325800	-1.37966400
H	0.48208800	3.63943100	-2.69017300
C	1.68225400	4.15392400	0.93779500
H	0.44136900	2.47001500	1.43057400
C	2.15559300	4.99573500	-0.06581400
H	2.06104800	5.45503000	-2.17436000
H	1.98759200	4.31233900	1.96950800
H	2.83661300	5.80912500	0.17097300
C	-1.87103100	2.57020100	-1.59551600
C	-2.51451400	3.51005500	-0.76582600
C	-2.34844100	2.44197800	-2.91203600
C	-3.60513200	4.25099900	-1.21451400
H	-2.14084400	3.65890300	0.24307000

C	-3.44169900	3.18593400	-3.36612000
H	-1.84833100	1.77171600	-3.60468300
C	-4.08294900	4.08853600	-2.51963700
H	-4.08019800	4.96467000	-0.54567000
H	-3.78115200	3.06415000	-4.39196800
H	-4.93032900	4.67000700	-2.87257200
H	1.33509800	-1.76141600	0.74916900
C	2.12475600	-0.07869500	3.41638300
H	2.03380400	-0.89157400	4.14341000
H	3.19136500	0.04257900	3.19638600
H	1.73150300	0.84987700	3.83159000
C	1.39736300	-0.43632800	2.14295900
O	1.65351500	-1.64641700	1.71398000
O	0.64148700	0.36742400	1.58506900

INT-4

C	1.26691500	-3.36724400	-0.08228200
C	2.07253900	-3.12376500	1.04225500
C	3.05704300	-2.11690700	0.64313100
C	1.82059300	-2.62676100	-1.23008200
C	2.96517000	-1.93388200	-0.80719100
C	1.97319600	-3.75226400	2.40173500
H	0.97117700	-4.14925600	2.59133600
H	2.68338600	-4.58555100	2.50864400
H	2.19887100	-3.03299400	3.19574200
C	4.22149600	-1.67799100	1.48185700
H	4.60409700	-0.70467500	1.16568600
H	3.95193400	-1.59997800	2.53934100
H	5.04395200	-2.40462300	1.40270100

C	3.94057200	-1.15798000	-1.64135800
H	4.85944100	-1.73784600	-1.81209000
H	3.52437700	-0.90829900	-2.62218100
H	4.23301400	-0.22214600	-1.15455200
C	1.25423900	-2.70511200	-2.61857200
H	1.43723800	-3.69179300	-3.06787700
H	0.17043300	-2.54429800	-2.61310600
H	1.69918300	-1.95433200	-3.27831400
C	0.05809000	-4.25269400	-0.17326800
H	-0.79206500	-3.71536100	-0.60888600
H	0.25513000	-5.13052600	-0.80503500
H	-0.24885200	-4.61612500	0.81171000
Rh	1.17067200	-1.09496000	0.40435500
C	-3.16606500	2.99758600	2.84063800
C	-2.11303500	2.09003500	2.98686000
C	-1.94009000	1.11517200	2.00929900
C	-2.81093100	1.04053600	0.91966800
C	-3.86423300	1.94207500	0.76494300
C	-4.03233600	2.92490600	1.73998800
H	-3.31607500	3.77001700	3.59012400
H	-1.44357400	2.15234700	3.84078100
H	-4.52555700	1.87262500	-0.09266800
H	-4.84307800	3.64210800	1.64721400
C	-2.38827000	-0.08116100	0.08139800
O	-1.29027200	-0.67431900	0.64463200
N	-2.96658600	-0.45812700	-0.99726700
O	-2.32760800	-1.56578100	-1.58212500
C	-3.06971500	-1.93978600	-2.73594600
H	-3.10923300	-1.11997000	-3.46321100

H	-2.53590600	-2.79186700	-3.16464300
H	-4.09144800	-2.23681500	-2.47008300
C	-0.94025900	-0.01702300	1.93141800
H	-1.15716300	-0.76002500	2.70931000
C	0.55323600	0.21588700	1.91403700
H	1.01832800	0.05841000	2.88314800
C	1.23393400	1.01773200	0.91474900
C	0.47104500	1.92058200	-0.03836100
C	0.15907900	3.22988700	0.37316600
C	0.10723600	1.54092100	-1.33736500
C	-0.50772700	4.11454900	-0.47152600
H	0.44793100	3.55257500	1.36998900
C	-0.56159100	2.42548300	-2.18934500
H	0.34949300	0.53777500	-1.67835800
C	-0.87288100	3.71465100	-1.76047600
H	-0.74192400	5.11802300	-0.12464900
H	-0.83727600	2.10105000	-3.18960400
H	-1.39240500	4.40335900	-2.42180600
C	2.60819200	1.54616300	1.22097400
C	3.40920400	2.05355000	0.17660500
C	3.14832300	1.59193800	2.51952000
C	4.68894600	2.55160100	0.41303800
H	3.01150000	2.05839500	-0.83380600
C	4.42990000	2.09201500	2.75927700
H	2.55870600	1.25216200	3.36508000
C	5.21327500	2.57028700	1.70881300
H	5.27714200	2.93229100	-0.41861100
H	4.81067400	2.11675500	3.77771600
H	6.20929100	2.96228000	1.89629200

INT-4'

C	-2.80493500	-1.78093200	1.23864700
C	-3.03233200	-1.65901500	-0.14326700
C	-3.43427000	-0.27533800	-0.38229400
C	-3.21114700	-0.52670400	1.89534400
C	-3.66842200	0.36075300	0.91695000
C	-2.92662600	-2.73080100	-1.18984100
H	-2.30795200	-3.56784300	-0.85086100
H	-3.91639200	-3.13806100	-1.44441700
H	-2.48780400	-2.35048300	-2.11881500
C	-3.98581700	0.23720200	-1.68225100
H	-3.88438100	1.32142400	-1.76871300
H	-3.48014200	-0.21253500	-2.54319000
H	-5.05472100	-0.00833200	-1.76879000
C	-4.31151500	1.70104100	1.11057700
H	-5.39049600	1.65007100	0.90268000
H	-4.19345200	2.06152100	2.13713000
H	-3.88938300	2.45812500	0.44196300
C	-3.16143000	-0.31403700	3.38027900
H	-3.82598400	-1.01106900	3.90967500
H	-2.14722000	-0.47183000	3.76608200
H	-3.46259800	0.70172800	3.65173900
C	-2.37122000	-3.01088300	1.98190500
H	-1.69695900	-2.76133100	2.80739700
H	-3.23254800	-3.53704500	2.41894900
H	-1.85120800	-3.71820300	1.32833900
Rh	-1.38603300	-0.13892000	0.35005800
C	1.93045700	0.09320700	-5.40258900

C	1.05752300	-0.22784900	-4.35847300
C	1.58270800	-0.37652700	-3.07748200
C	2.95072300	-0.21843600	-2.85331700
C	3.83115800	0.10314400	-3.88750500
C	3.30357500	0.25872700	-5.16893600
H	1.53973400	0.21528600	-6.40904300
H	-0.00409000	-0.35949100	-4.54980100
H	4.89129400	0.22454800	-3.68954200
H	3.96198400	0.50936100	-5.99586400
C	3.19362200	-0.44729200	-1.43405500
O	2.00088000	-0.72241500	-0.80850400
N	4.33732000	-0.40687400	-0.86209200
O	4.23428600	-0.67692000	0.50659300
C	5.52235400	-0.57020000	1.09424900
H	5.91664000	0.44832600	0.99750600
H	5.37865200	-0.81487300	2.14945100
H	6.22516200	-1.27863300	0.63902400
C	0.89694200	-0.73247700	-1.77217600
H	0.54293500	-1.77188700	-1.80751800
C	-0.28761500	0.14447300	-1.40044300
H	-1.00789400	0.03598200	-2.21509800
C	-0.33075600	1.42990600	-0.73017300
C	0.87885300	2.03699200	-0.04101300
C	1.92178500	2.55720200	-0.82822000
C	0.95066400	2.22914900	1.34586400
C	3.01109400	3.20867500	-0.25098900
H	1.86977900	2.45949100	-1.90940400
C	2.03651300	2.88915000	1.92776900
H	0.14979500	1.85108700	1.97153500

C	3.07453700	3.37688300	1.13418400
H	3.80693600	3.59157700	-0.88513100
H	2.06604700	3.02370400	3.00671700
H	3.91940900	3.88974100	1.58703000
C	-1.20465300	2.51677300	-1.30688100
C	-1.68653200	3.55747900	-0.48803300
C	-1.50680000	2.58799300	-2.67850800
C	-2.45600500	4.59621900	-1.00804100
H	-1.44582700	3.54691100	0.57091700
C	-2.28192900	3.62589600	-3.20280800
H	-1.10758700	1.83688000	-3.35333000
C	-2.76676200	4.63453200	-2.37105400
H	-2.81343100	5.38150900	-0.34610500
H	-2.49212100	3.65065800	-4.26964000
H	-3.36488800	5.44576600	-2.77746000
H	1.91695800	-1.47220600	0.79911000
C	1.10591900	-1.87610200	3.89463700
H	1.15005800	-2.96957800	3.92311600
H	2.04209200	-1.50203200	4.32349100
H	0.26391800	-1.51697700	4.48692300
C	0.98430100	-1.39991100	2.46692000
O	1.91226000	-1.92189200	1.68267700
O	0.11932400	-0.60942100	2.09924400

PC-1

C	-1.22025700	1.86471700	0.21071300
C	-0.17442400	1.03932600	0.62864300
C	-0.07375600	0.64523500	1.95976100

C	-1.03542300	1.10666200	2.86412700
C	-2.07989800	1.94128000	2.44063700
C	-2.18601000	2.32816600	1.10477400
C	-1.06496600	2.07747800	-1.22795400
C	0.70217900	0.72042100	-0.56642500
H	0.72336500	-0.01349000	2.29341800
H	-0.97500600	0.81102800	3.90794500
H	-2.81640400	2.28575500	3.16126000
H	-2.99135000	2.96825600	0.75900800
O	0.03194500	1.40265000	-1.67684600
N	-1.85015200	2.79244800	-1.94597700
O	-1.45436900	2.80744400	-3.29127000
C	-2.35957600	3.62771700	-4.01304400
H	-3.38408300	3.23854300	-3.95590000
H	-2.01164100	3.60306300	-5.04914100
H	-2.34620900	4.66028200	-3.64162600
C	0.80435600	-0.73846500	-0.90299000
H	-0.15585700	-1.22106900	-1.07194200
C	1.93327200	-1.46305000	-1.05762000
C	3.29684600	-0.91440700	-0.79136400
C	3.63561100	-0.34151300	0.44495600
C	4.28467300	-0.97545500	-1.79053100
C	4.91334500	0.17551900	0.67059300
H	2.89759900	-0.31964000	1.24181100
C	5.55710700	-0.45272100	-1.56908500
H	4.04268700	-1.42622300	-2.74870400
C	5.87645300	0.12558900	-0.33718800
H	5.15480900	0.61126900	1.63645200
H	6.30198100	-0.49712200	-2.35914400

H	6.87073700	0.52811900	-0.16374700
C	1.85128600	-2.87816200	-1.52265300
C	2.72266200	-3.85328900	-1.00608000
C	0.89945300	-3.27561000	-2.47828500
C	2.62720000	-5.18420900	-1.41033000
H	3.47159500	-3.56443700	-0.27461200
C	0.80751000	-4.60573300	-2.88580900
H	0.24632600	-2.52931200	-2.92107500
C	1.66823600	-5.56669500	-2.35071000
H	3.30380000	-5.92361200	-0.98986200
H	0.06983000	-4.88921000	-3.63192000
H	1.59842400	-6.60274200	-2.67104700
H	1.69456400	1.17286200	-0.47628400

PC-2

C	-1.10845900	2.38905000	0.25200800
C	-0.11917400	1.44664000	0.54551000
C	-0.02282300	0.90591700	1.82415500
C	-0.91401600	1.35805900	2.80381000
C	-1.89064900	2.32004800	2.50863200
C	-2.00012400	2.84444300	1.21975900
C	-1.00589300	2.78144200	-1.17698100
C	0.71521500	1.16775300	-0.69647500
H	0.71904800	0.14794100	2.06043800
H	-0.84926700	0.95471300	3.81088700
H	-2.56972500	2.65154700	3.28923100
H	-2.75389700	3.58273000	0.96261100
C	0.86304100	-0.29243100	-1.02997700
H	-0.08280400	-0.81309300	-1.16541200

C	2.01261600	-0.98017200	-1.20190400
C	3.36299700	-0.37965000	-0.98029400
C	3.71319400	0.22550800	0.23744500
C	4.32726300	-0.42512800	-2.00282700
C	4.97810600	0.78840100	0.42141400
H	2.99447900	0.24092900	1.05194000
C	5.58709400	0.14222100	-1.82259800
H	4.07739900	-0.90090000	-2.94688500
C	5.91742600	0.75217900	-0.60918500
H	5.22847300	1.24946000	1.37313400
H	6.31330400	0.10805600	-2.63037600
H	6.90168600	1.19025400	-0.46768400
C	1.96956400	-2.40745100	-1.63605700
C	2.89881600	-3.33541100	-1.13307200
C	0.99874400	-2.86782600	-2.54315200
C	2.84224300	-4.67859700	-1.50267100
H	3.66334000	-2.99982100	-0.43886300
C	0.94421000	-4.21016700	-2.91574700
H	0.29623600	-2.16134400	-2.97594300
C	1.86374200	-5.12286500	-2.39432200
H	3.56470500	-5.37942000	-1.09256500
H	0.18921600	-4.54102100	-3.62415900
H	1.82359700	-6.16843600	-2.68751000
H	1.69771500	1.64918700	-0.62160700
O	-1.59791200	3.65129400	-1.78951500
N	-0.07773400	1.88235800	-1.71681700
O	0.63342100	2.28407100	-2.84885800
C	-0.01521900	1.82137300	-4.03637300
H	-0.08935100	0.72711200	-4.04482000

H	-1.00861800	2.26942100	-4.13398400
H	0.62647000	2.15724700	-4.85509100

HOAc

C	-0.09234800	0.12567600	-0.00017000
O	-0.64606900	1.20160200	0.00006000
O	-0.77752000	-1.04692700	0.00005800
C	1.39689700	-0.10922500	-0.00002600
H	1.68352300	-0.69397400	-0.88019800
H	1.91626800	0.84909000	-0.00331500
H	1.68408200	-0.68801400	0.88394300
H	-1.72245400	-0.80320700	-0.00020300

AgOAc

C	-0.17280800	0.17262400	-0.00051100
O	0.79702800	-0.57351900	0.33267300
O	-0.10503500	1.43974800	-0.06178800
C	-1.51089600	-0.47689700	-0.32979800
H	-2.23070300	-0.22437400	0.45658800
H	-1.89749100	-0.07099100	-1.26894500
H	-1.41341700	-1.56127900	-0.39661500
Ag	2.10624200	1.32917200	0.61423300

Cp*Rh(OAc)$_2$ + 2Ag

C	1.99232600	-0.57850800	-1.10358200
C	2.19947000	0.55453800	-0.18397400
C	2.04851300	0.08625100	1.16793100
C	1.47635400	-1.23091300	1.06807300

C	1.54421900	-1.66005600	-0.33922000
C	2.55469700	0.76676100	2.40945000
H	3.61984500	0.52810000	2.55944900
H	2.46651900	1.85380100	2.34376200
H	2.01417500	0.44140500	3.30155300
C	1.17986000	-2.16165700	2.20959900
H	0.96423400	-1.61032800	3.12854100
H	0.31235700	-2.79241400	1.99212100
H	2.03294300	-2.82750100	2.40728500
C	1.13506800	-3.02401300	-0.81049500
H	1.81909800	-3.79570100	-0.43188100
H	0.13106200	-3.27957800	-0.45120300
H	1.13039100	-3.09320400	-1.90223300
C	2.21370600	-0.51600400	-2.58649400
H	1.84235600	-1.41388400	-3.08913000
H	1.70751900	0.34551300	-3.03547100
H	3.28299700	-0.42327900	-2.82278000
C	2.76752400	1.87966800	-0.60064300
H	2.61034500	2.63930500	0.16955000
H	3.84864000	1.80249000	-0.78658000
H	2.29499700	2.24031300	-1.51896700
Rh	0.08668000	0.16388100	0.13354800
C	-0.97130500	2.14847900	-2.30059600
O	-1.48206200	1.30498100	-3.11334500
O	-0.63099100	1.93618200	-1.10315700
C	-2.89787600	-1.04331000	1.06533800
O	-3.01885900	-0.31670300	2.06118000
O	-1.89917400	-1.02273300	0.22616200
C	-3.98885100	-2.06239400	0.75137600

H	-4.68161100	-1.63394900	0.01585400
H	-3.56254300	-2.97359500	0.32250600
H	-4.55610000	-2.29710100	1.65417900
C	-0.76623100	3.55250200	-2.85676200
H	-0.07049000	3.51045400	-3.70225600
H	-1.71613800	3.93737000	-3.24131900
H	-0.37486100	4.22431200	-2.09128500
Ag	-1.16707000	1.26070500	2.21466500
Ag	-1.94463800	-0.45958300	-1.92571400

INT-0$_{Bn}$

C	-1.63253800	1.36363100	1.24361200
C	-0.46444300	0.81764200	0.63517300
C	0.43264600	1.92653400	0.27065400
C	-1.39106900	2.76964700	1.42209800
C	-0.14582400	3.11696500	0.72684600
C	-0.24461600	-0.60937100	0.23291700
H	-0.86170300	-1.30060600	0.81051700
H	-0.48887200	-0.74393200	-0.83041900
H	0.79805700	-0.91038600	0.37045000
C	1.74791200	1.79055200	-0.43608700
H	2.13746100	0.77166000	-0.36195800
H	1.64865400	2.02837100	-1.50406000
H	2.49417800	2.46380300	-0.00379900
C	0.39236200	4.50228300	0.54331800
H	1.48366300	4.50244300	0.48668800
H	0.00773500	4.94414900	-0.38669600
H	0.09542700	5.16205000	1.36380100
C	-2.36811200	3.75532400	1.98746100

H	-3.01465400	4.15192300	1.19101800
H	-3.01312600	3.29261900	2.73931100
H	-1.86060800	4.60414400	2.45425100
C	-2.90578000	0.65046600	1.59739400
H	-3.22832600	0.86095600	2.62324900
H	-3.71801600	0.96811500	0.93000000
H	-2.80557400	-0.43267700	1.49387200
Rh	0.15744800	1.73974300	2.58150300
C	-0.27640700	0.75367700	8.06479300
C	-0.70792000	0.56365500	6.75620300
C	0.06722800	0.95636500	5.64912900
C	1.30640800	1.57347100	5.89964700
C	1.74571000	1.74568300	7.22496000
C	0.96663900	1.34612500	8.30252600
H	-0.90378500	0.43704800	8.89447300
H	-1.66620100	0.08274100	6.57220600
H	2.71666000	2.20429800	7.37518000
H	1.32154300	1.49692600	9.31853800
C	2.24250900	2.11047700	4.85953700
O	3.41639300	2.36428100	5.14129900
N	1.70637800	2.39704700	3.61195700
O	2.64258300	3.05780200	2.77378800
C	2.87160200	4.42523300	3.17963800
H	1.97189100	5.02429000	2.98728200
H	3.09375300	4.44335300	4.24957500
C	-0.51127700	0.66743500	4.28668700
H	-1.56152700	0.98652100	4.30793600
C	-0.50858700	-0.75000900	3.82799700
H	-1.35079700	-0.95961600	3.17272100

C	0.29696500	-1.82027300	4.06918700
C	1.57784000	-1.77656400	4.83716700
C	1.71379100	-2.50641700	6.03034200
C	2.69180000	-1.07526100	4.35386100
C	2.91754900	-2.50772000	6.73277600
H	0.86337800	-3.06736600	6.40871400
C	3.90041900	-1.07860900	5.05390100
H	2.60702600	-0.51677100	3.42621200
C	4.01584200	-1.79317900	6.24620300
H	2.99896800	-3.06658100	7.66177200
H	4.74230500	-0.50876600	4.67222200
H	4.95397100	-1.79238800	6.79499800
C	-0.08567500	-3.15000000	3.51208400
C	0.90009500	-4.06301900	3.08826500
C	-1.43314400	-3.54409800	3.38695000
C	0.55398400	-5.29438000	2.53383400
H	1.94720300	-3.79483400	3.18899000
C	-1.77954400	-4.77518300	2.83127300
H	-2.21513200	-2.88996300	3.76237500
C	-0.78777400	-5.65730200	2.39702500
H	1.33755900	-5.97362500	2.20715800
H	-2.82784600	-5.05434500	2.75677200
H	-1.05666300	-6.62048700	1.97157600
C	4.05170100	4.92760300	2.38976900
C	3.95567800	6.04858800	1.55879000
C	5.28222000	4.26223300	2.50020500
C	5.07054700	6.50687800	0.85078000
H	3.00619900	6.57262600	1.47080500
C	6.39198100	4.71345700	1.78823300

H	5.34891400	3.39549900	3.15143500
C	6.28953900	5.83787300	0.96224800
H	4.98411100	7.38247000	0.21238100
H	7.34148000	4.19235500	1.88061500
H	7.15737000	6.19037300	0.41074300

INT-1$_{Bn}$

C	-2.01348700	-2.38195500	-2.42274900
C	-2.73196400	-1.14487500	-2.30411300
C	-3.00522400	-0.91294800	-0.89631600
C	-1.72705700	-2.84614900	-1.09742200
C	-2.38444900	-1.94177100	-0.15616200
C	-3.32366300	-0.35292400	-3.43517600
H	-2.74501900	-0.46402300	-4.35805600
H	-4.34571800	-0.69310600	-3.65279900
H	-3.37793200	0.71377600	-3.19732200
C	-3.85034600	0.19693500	-0.34929400
H	-3.72750500	1.12485400	-0.91470800
H	-4.91491300	-0.07571600	-0.39063600
H	-3.60473300	0.40972000	0.69463700
C	-2.49500700	-2.15168300	1.32203300
H	-2.48614100	-1.20258000	1.86504000
H	-3.43300800	-2.67220600	1.56329000
H	-1.66974400	-2.75602600	1.69595400
C	-1.06926100	-4.14468600	-0.74010200
H	-0.41452400	-4.49371900	-1.54317700
H	-0.46494800	-4.05510000	0.16423600
H	-1.82575500	-4.92269200	-0.56328200
C	-1.66633100	-3.08514900	-3.70252000

H	-1.54046000	-2.38336500	-4.53317800
H	-0.74190000	-3.66200600	-3.61002500
H	-2.46606800	-3.78320700	-3.98684200
Rh	-0.67746200	-0.87590900	-1.35398600
C	4.25094700	-1.25247300	-3.98337900
C	2.88598100	-0.98782100	-3.97073500
C	2.16523300	-0.82541500	-2.76949700
C	2.85043300	-0.99058600	-1.54984100
C	4.23289200	-1.24228900	-1.57796300
C	4.93320700	-1.37304700	-2.77125300
H	4.77499900	-1.36055400	-4.92961600
H	2.35482800	-0.88131800	-4.91444800
H	4.73494900	-1.32668200	-0.62111700
H	6.00092100	-1.57592400	-2.75663100
C	2.26254100	-0.92147700	-0.15840200
O	3.04792900	-0.77818500	0.80011800
N	0.93513200	-1.14493300	-0.03383700
C	0.72078300	-0.51502400	-2.94572100
H	0.35447200	-0.99455800	-3.85408400
C	-0.02751700	0.69789000	-2.69719000
H	-0.86602500	0.82886700	-3.37476000
C	0.00628200	1.56619700	-1.58838300
C	1.27478600	1.96452400	-0.89561000
C	2.28726400	2.52337800	-1.69680300
C	1.45603900	1.95013300	0.49486200
C	3.43735100	3.06485300	-1.12441600
H	2.16081200	2.54432100	-2.77574400
C	2.60901800	2.48790300	1.06457300
H	0.73375600	1.44451300	1.12567800

C	3.59825300	3.05628300	0.26162500
H	4.20604600	3.49138800	-1.76358900
H	2.74355600	2.43841400	2.14123500
H	4.49578200	3.47272400	0.71111000
C	-1.11987300	2.55110900	-1.44801900
C	-1.53903400	3.00769100	-0.18438200
C	-1.76336900	3.09394300	-2.57764700
C	-2.56777300	3.93927500	-0.05533700
H	-1.05693600	2.62492600	0.70707800
C	-2.79146600	4.02978800	-2.44946400
H	-1.43292400	2.81619000	-3.57401200
C	-3.20454500	4.45499000	-1.18643900
H	-2.87069500	4.26485400	0.93647400
H	-3.25628900	4.43861200	-3.34306200
H	-4.00169100	5.18624700	-1.08493800
C	1.03597600	-1.73167100	2.39183600
H	0.35417000	-1.48678900	3.21358900
H	2.01728800	-1.29948200	2.57848200
C	1.14697700	-3.23371000	2.25751000
C	2.18954400	-3.81580200	1.51879600
C	0.25216900	-4.08069400	2.92847800
C	2.30457200	-5.20309500	1.42327400
H	2.92003800	-3.16805000	1.04551800
C	0.37036900	-5.47018400	2.84293900
H	-0.53663300	-3.64716100	3.54007600
C	1.39329900	-6.03527200	2.07980400
H	3.11659200	-5.63669600	0.84483100
H	-0.33007200	-6.10766200	3.37679800
H	1.48966100	-7.11577600	2.00920200

O	0.41248800	-1.00195800	1.29274100

INT-3$_{Bn}$

C	0.23349300	-2.87530200	0.14366400
C	0.92161700	-2.84171500	1.39496600
C	2.23102500	-2.28688100	1.15672700
C	1.16771500	-2.43013200	-0.89395300
C	2.39979500	-2.11143900	-0.27605800
C	0.39628400	-3.32408000	2.71510800
H	-0.69652800	-3.30437800	2.74394800
H	0.71329600	-4.35964500	2.90098900
H	0.76634000	-2.71837500	3.54883000
C	3.33227000	-2.15027100	2.16839300
H	3.97462700	-1.29212600	1.94910800
H	2.93820200	-2.02725100	3.18217700
H	3.96789500	-3.04700200	2.17340000
C	3.67719700	-1.69425100	-0.94027100
H	4.43666000	-2.48167600	-0.83701100
H	3.53298900	-1.51397200	-2.00871900
H	4.09221700	-0.78024500	-0.50296100
C	0.80947400	-2.34671100	-2.34586600
H	0.56815200	-3.34113300	-2.74366700
H	-0.07145600	-1.70663000	-2.47564400
H	1.62902000	-1.93550700	-2.94179000
C	-1.13744100	-3.41486600	-0.13386600
H	-1.63835300	-2.83432200	-0.91282000
H	-1.07874400	-4.45747900	-0.47780700
H	-1.77022000	-3.39704600	0.75792600
Rh	0.69064900	-0.73313800	0.56821900

C	-3.26360900	2.51242200	2.93385900
C	-2.06776200	1.80527600	2.98825000
C	-1.59410500	1.05399300	1.89528000
C	-2.38080100	0.99401300	0.72794300
C	-3.58968300	1.71483000	0.68561700
C	-4.03018400	2.46722200	1.76693600
H	-3.59290100	3.09209900	3.79242800
H	-1.47098100	1.83294800	3.89763800
H	-4.17409600	1.66572200	-0.22545400
H	-4.96534100	3.01714400	1.70013400
C	-1.98201700	0.19646100	-0.47458300
O	-0.72760200	-0.01077600	-0.76427400
N	-2.98232600	-0.19246100	-1.21864200
O	-2.52455600	-0.91708000	-2.35168000
C	-3.65943500	-1.34358100	-3.10737300
H	-4.38052400	-0.51839400	-3.15101900
H	-3.26844800	-1.51950000	-4.11553400
C	-0.31365500	0.31061700	2.15685900
H	-0.41525800	-0.33330100	3.03282700
C	1.03138900	0.80840600	2.02129000
H	1.74505300	0.41003900	2.73666300
C	1.54616600	1.50270200	0.89555600
C	0.74146100	2.54315200	0.16356700
C	0.27742200	3.64473300	0.90160700
C	0.52152000	2.53163800	-1.22227400
C	-0.38708700	4.70080600	0.27822300
H	0.44225600	3.67173200	1.97501100
C	-0.13877200	3.58982200	-1.84685300
H	0.82289600	1.66941900	-1.80450200

C	-0.59480700	4.67848300	-1.10123400
H	-0.74110700	5.53894500	0.87270100
H	-0.31043800	3.55330100	-2.91940900
H	-1.11351400	5.49862500	-1.59070800
C	3.03594400	1.65856900	0.77732700
C	3.63571300	1.95496000	-0.46255300
C	3.89058000	1.56449000	1.89478200
C	5.01462000	2.12099200	-0.58431400
H	3.01302800	2.06074600	-1.34279500
C	5.27047800	1.73489000	1.77475100
H	3.47825600	1.39500400	2.88414000
C	5.84447800	2.00860900	0.53279100
H	5.43965700	2.34465900	-1.55941000
H	5.89464900	1.66880100	2.66235100
H	6.91823000	2.14534600	0.43935500
C	-4.31442700	-2.60551200	-2.58195500
C	-4.18606600	-3.81416200	-3.27717700
C	-5.05772100	-2.58984600	-1.39129500
C	-4.78473700	-4.98371300	-2.80210700
H	-3.61478300	-3.83922000	-4.20318700
C	-5.65042700	-3.75747200	-0.91053100
H	-5.15221000	-1.65738600	-0.84408100
C	-5.51798800	-4.95773500	-1.61518500
H	-4.67852600	-5.91166000	-3.35866000
H	-6.22308500	-3.73040100	0.01334900
H	-5.98644000	-5.86513000	-1.24224900

TS-4$_{Bn}$

C	1.03164300	-3.24844700	-0.05330100

C	1.92164000	-3.13255600	1.02929200
C	2.93974700	-2.14907300	0.65752300
C	1.52274700	-2.40014700	-1.14624900
C	2.74435800	-1.80106000	-0.73614900
C	1.85981400	-3.85742700	2.34109300
H	0.84720400	-4.20743200	2.56059900
H	2.52031200	-4.73660000	2.33649600
H	2.18086300	-3.22001700	3.17155300
C	4.16886300	-1.84135700	1.46087500
H	4.59756800	-0.87375000	1.19123100
H	3.95704000	-1.82130100	2.53438900
H	4.93743700	-2.60974600	1.29231200
C	3.69058100	-1.00457200	-1.58182700
H	4.51294800	-1.63936000	-1.94184400
H	3.18954600	-0.58515600	-2.45920400
H	4.13336300	-0.17565200	-1.02300700
C	0.87177100	-2.31446000	-2.49425200
H	0.97610000	-3.26449900	-3.03765600
H	-0.19672000	-2.09860100	-2.39325700
H	1.32285600	-1.53058800	-3.10954000
C	-0.21553100	-4.07487900	-0.14209800
H	-1.05713100	-3.46615600	-0.48870100
H	-0.08302500	-4.90472000	-0.85041600
H	-0.48521100	-4.50466700	0.82673000
Rh	1.06062700	-1.01384300	0.56202000
C	-3.16232100	3.06185700	2.61878800
C	-2.08403000	2.21506500	2.87494200
C	-1.80426800	1.15998100	1.99767900
C	-2.61318400	0.95806200	0.87457800

C	-3.69071700	1.81033300	0.61543100
C	-3.96283600	2.86057800	1.48856500
H	-3.37809000	3.87966200	3.30115100
H	-1.46023100	2.37272700	3.75111900
H	-4.30035900	1.63386200	-0.26441200
H	-4.80125000	3.52358900	1.29268500
C	-2.21560200	-0.21595000	0.06130000
O	-1.24143200	-0.91733300	0.59391200
N	-2.89203500	-0.45988900	-1.01828600
O	-2.40897500	-1.62405400	-1.67909900
C	-3.28608800	-1.91451400	-2.75799500
H	-3.45160200	-1.00791100	-3.35447700
H	-2.72297900	-2.62787600	-3.37443300
C	-0.69744000	0.20530400	2.30230100
H	-0.95036500	-0.55490200	3.04101000
C	0.68709500	0.43409300	2.15710200
H	1.30471400	-0.01059400	2.93326700
C	1.34219800	1.11948400	1.05048300
C	0.57839700	2.07701600	0.15593100
C	0.33715600	3.38721300	0.60650700
C	0.18125300	1.74521700	-1.14550500
C	-0.29983100	4.32214900	-0.20605700
H	0.65377500	3.67020200	1.60700800
C	-0.45498700	2.68170900	-1.96574700
H	0.36706900	0.73937800	-1.51074500
C	-0.69909500	3.97207400	-1.49937400
H	-0.48448400	5.32560400	0.16916300
H	-0.76287500	2.39491900	-2.96781000
H	-1.19601900	4.70053500	-2.13481700

C	2.77315300	1.53975000	1.23852000
C	3.50562600	2.05590600	0.14923200
C	3.43033800	1.49126000	2.48354200
C	4.82945800	2.46897000	0.28906500
H	3.01947600	2.14147800	-0.81736800
C	4.75514800	1.90705700	2.62612100
H	2.89874700	1.15235700	3.36679000
C	5.46839200	2.39229100	1.52944800
H	5.36115400	2.86013100	-0.57490200
H	5.22512900	1.86297600	3.60570300
H	6.49820900	2.72013000	1.64173300
C	-4.61476000	-2.52054600	-2.34470200
C	-4.76698800	-3.17228000	-1.11643500
C	-5.70164400	-2.47373200	-3.22577900
C	-5.98331700	-3.76793500	-0.77811100
H	-3.93125300	-3.19683900	-0.42420400
C	-6.91601500	-3.07580900	-2.89327800
H	-5.59866900	-1.95820600	-4.17907600
C	-7.06067500	-3.72553400	-1.66555600
H	-6.09030200	-4.26494300	0.18298800
H	-7.75088800	-3.02922600	-3.58812300
H	-8.00723900	-4.18956500	-1.40067100

TS-2$_{Bn}$

C	-3.05085900	-2.00748800	-1.00190000
C	-3.11412200	-0.56123000	-1.17886300
C	-2.96351600	0.05644500	0.12232100
C	-2.74022800	-2.26610400	0.33554700
C	-2.60846200	-0.98127900	1.02974000

C	-3.69475700	0.09381000	-2.39832400
H	-3.30034500	-0.33798100	-3.32291400
H	-4.78612100	-0.04452300	-2.41113500
H	-3.49541200	1.16599700	-2.42657900
C	-3.25313500	1.48829600	0.46340300
H	-3.02103000	2.15836600	-0.36787200
H	-4.31962000	1.61681600	0.70009000
H	-2.68244900	1.82289700	1.33482500
C	-2.35757700	-0.84117200	2.50343300
H	-2.10931600	0.18954200	2.77173600
H	-3.24916600	-1.12775500	3.08017700
H	-1.52893600	-1.47905300	2.82196400
C	-2.68802800	-3.60234000	1.00937400
H	-2.42989800	-4.40503900	0.31162600
H	-1.95750900	-3.60820300	1.81951200
H	-3.66905600	-3.84938300	1.44136200
C	-3.31775000	-3.01045300	-2.08480500
H	-2.89334000	-2.69220200	-3.04298000
H	-2.89993300	-3.99198700	-1.84209100
H	-4.39803500	-3.14304100	-2.24321900
Rh	-0.97809700	-0.76331900	-0.54354300
C	4.91741800	0.11737800	-1.51571700
C	3.69383000	-0.37203900	-1.97162400
C	2.71940400	-0.78086200	-1.05100200
C	2.97626900	-0.67577700	0.31807300
C	4.19678400	-0.16873000	0.77324700
C	5.16697900	0.22784100	-0.14286800
H	5.67854500	0.41325600	-2.23304400
H	3.49828200	-0.45043600	-3.03796400

H	4.36478200	-0.11695300	1.84434100
H	6.12015300	0.61590800	0.20624000
C	1.97659800	-1.25009700	1.25979100
O	2.25241500	-1.48499600	2.44169700
N	0.83143400	-1.64354200	0.60546900
C	1.43270800	-1.34056800	-1.55490800
H	1.43745500	-2.39175700	-1.83338300
C	0.44969900	-0.52579300	-2.15501800
H	-0.09900300	-0.97513400	-2.97767100
C	0.07058100	0.81091000	-1.71559500
C	0.95868300	1.70594800	-0.86780600
C	1.93760800	2.49387400	-1.49939100
C	0.76114000	1.88352600	0.50755100
C	2.70842600	3.40188800	-0.77688100
H	2.09272700	2.39062400	-2.57003700
C	1.52701200	2.79924300	1.23505700
H	0.00530700	1.28519600	1.00632900
C	2.50457100	3.56054100	0.59680600
H	3.46679100	3.99020600	-1.28747800
H	1.35689400	2.91098000	2.30279600
H	3.10221800	4.27214300	1.16060300
C	-0.75238400	1.63254300	-2.67299300
C	-1.29860400	2.86495700	-2.25730500
C	-0.94802900	1.27170700	-4.02008800
C	-2.03668700	3.66765900	-3.12484300
H	-1.12500900	3.20089500	-1.24057900
C	-1.68078500	2.07778500	-4.89316800
H	-0.50161500	0.36374100	-4.41198400
C	-2.23961300	3.27677200	-4.45105200

H	-2.44513000	4.60875700	-2.76496000
H	-1.80217200	1.76871100	-5.92851300
H	-2.80739400	3.90545900	-5.13146600
C	0.99038800	-3.89343400	1.46549500
H	0.27939600	-4.58919900	1.92396400
H	1.77321100	-3.66113500	2.19137600
C	1.57706700	-4.48279800	0.20293700
C	2.95803500	-4.44290600	-0.02335400
C	0.75419500	-5.08102700	-0.76281800
C	3.50792900	-4.98263200	-1.18931200
H	3.60391100	-3.97884600	0.71781300
C	1.29757600	-5.62038800	-1.92873500
H	-0.31887400	-5.12485300	-0.59439900
C	2.67867800	-5.57067100	-2.14552300
H	4.58197100	-4.94084300	-1.34979500
H	0.64777200	-6.08720700	-2.66485300
H	3.10374700	-5.99375100	-3.05200500
O	0.17028100	-2.72247700	1.24829900

INT-4$_{Bn}$

C	1.10746600	-3.32729300	-0.12317900
C	1.93870500	-3.13350700	0.99211800
C	2.95273500	-2.15615200	0.59412100
C	1.66912400	-2.58728500	-1.26725700
C	2.84472700	-1.94406000	-0.85145100
C	1.84001700	-3.78501500	2.34064500
H	0.82549800	-4.14102000	2.54464300
H	2.51405600	-4.65122600	2.41527700
H	2.11503000	-3.09313800	3.14336200

C	4.14246700	-1.77275900	1.42415800
H	4.57223500	-0.82173300	1.10120500
H	3.88071700	-1.67496300	2.48192200
H	4.92655800	-2.54066800	1.34593900
C	3.83277900	-1.18542400	-1.68629100
H	4.73223700	-1.78915200	-1.87605100
H	3.41297600	-0.90894600	-2.65828100
H	4.15715700	-0.26593300	-1.18890500
C	1.07092100	-2.61223100	-2.64421600
H	1.17643000	-3.60354200	-3.10757000
H	0.00084300	-2.37794800	-2.61179800
H	1.55133900	-1.88535200	-3.30567900
C	-0.13069900	-4.17108800	-0.21208300
H	-0.96215600	-3.60393400	-0.64573700
H	0.03393400	-5.05341800	-0.84680100
H	-0.44789800	-4.52636400	0.77268500
Rh	1.10081600	-1.06126200	0.40576700
C	-3.02321100	3.18279400	2.94935600
C	-2.00913900	2.22875200	3.07325100
C	-1.89085200	1.25777300	2.08350300
C	-2.77736300	1.23411400	1.00451000
C	-3.79231000	2.18171000	0.87192300
C	-3.90536400	3.16016300	1.85913100
H	-3.13022700	3.95266500	3.70879300
H	-1.32759000	2.25255100	3.91950300
H	-4.46729700	2.14892100	0.02286800
H	-4.68530700	3.91272600	1.78457100
C	-2.41764700	0.10214300	0.15068100
O	-1.34093900	-0.54641900	0.69393500

N	-3.02870100	-0.23632000	-0.92222600
O	-2.43944800	-1.36422600	-1.53246000
C	-3.24018400	-1.74131900	-2.65126700
H	-3.43445800	-0.86154900	-3.27667100
H	-2.58964100	-2.42328000	-3.21307400
C	-0.94225000	0.08419300	1.98101800
H	-1.17888800	-0.65589700	2.75581800
C	0.55967800	0.25342900	1.94227500
H	1.03246900	0.06348300	2.90191500
C	1.25851600	1.04009100	0.94359900
C	0.52052500	1.98467000	0.01140400
C	0.25824100	3.29753900	0.44568100
C	0.13700400	1.63941400	-1.29157300
C	-0.38063000	4.21904800	-0.38101200
H	0.56349600	3.59356400	1.44584300
C	-0.50376400	2.56106900	-2.12559200
H	0.34345100	0.63453700	-1.65035600
C	-0.76641300	3.85330900	-1.67413800
H	-0.57677100	5.22459800	-0.01703000
H	-0.79550500	2.26334300	-3.12959500
H	-1.26422400	4.57083800	-2.32136700
C	2.65722900	1.50846900	1.23628300
C	3.46293800	1.99867000	0.18744000
C	3.21630800	1.51565500	2.52760800
C	4.76415900	2.44380600	0.41227300
H	3.05167000	2.03353500	-0.81690000
C	4.51932600	1.96269700	2.75576900
H	2.62520600	1.18819700	3.37694500
C	5.30630600	2.42465000	1.70064600

H	5.35522500	2.81293800	-0.42252500
H	4.91445700	1.95930700	3.76902000
H	6.31910800	2.77558100	1.87927600
C	-4.53884500	-2.43101600	-2.28396200
C	-4.65355600	-3.17792300	-1.10592800
C	-5.63096000	-2.36922600	-3.15679000
C	-5.83873000	-3.85207100	-0.80913000
H	-3.81340500	-3.21728700	-0.41898700
C	-6.81419900	-3.05062200	-2.86646000
H	-5.55713700	-1.78037500	-4.06909000
C	-6.92150100	-3.79413800	-1.68960200
H	-5.91717200	-4.42396100	0.11207100
H	-7.65430100	-2.99198500	-3.55376200
H	-7.84384400	-4.32020000	-1.45774100

INT-2$_{Bn}$

C	-2.92106600	-2.13046000	-1.43248200
C	-3.14775300	-0.68572300	-1.48198100
C	-3.38675100	-0.21734200	-0.11733800
C	-2.82915400	-2.48526300	-0.07980900
C	-3.08994300	-1.28937700	0.73738200
C	-3.55400300	0.05842400	-2.72125300
H	-2.96750800	-0.24906400	-3.59219700
H	-4.61233800	-0.13775200	-2.94956000
H	-3.43144900	1.13717400	-2.60765900
C	-3.90245100	1.13886100	0.26194400
H	-3.48751400	1.92331400	-0.37693300
H	-4.99717300	1.18210500	0.16207700
H	-3.65993800	1.38914000	1.29970500

C	-3.17266000	-1.30321300	2.23765800
H	-3.12904100	-0.29280300	2.65450400
H	-4.11172100	-1.76141500	2.58090200
H	-2.35651700	-1.88123700	2.68632300
C	-2.67736500	-3.86504600	0.49339500
H	-2.35720300	-4.59030800	-0.25943500
H	-1.95262600	-3.90107900	1.31457700
H	-3.63438600	-4.22017000	0.90256600
C	-2.86452600	-3.03769400	-2.62774600
H	-2.35561500	-2.56183000	-3.47232800
H	-2.33615900	-3.97055200	-2.40641500
H	-3.87431500	-3.30713000	-2.97125200
Rh	-1.17252100	-0.80828900	-0.59152300
C	4.66810400	0.08688300	-3.65060300
C	3.36514000	-0.39372900	-3.47374100
C	2.94385200	-0.69878100	-2.18374800
C	3.81584200	-0.53988100	-1.10186700
C	5.11335900	-0.06623900	-1.26760700
C	5.53513300	0.24908300	-2.56093300
H	5.01308700	0.33871000	-4.65000800
H	2.70237200	-0.51552100	-4.32671300
H	5.76483000	0.05340900	-0.40704100
H	6.54169200	0.62282500	-2.72744900
C	3.11625000	-0.90703100	0.15889300
O	3.49539800	-0.83923000	1.31706600
N	1.90913700	-1.42649400	-0.28337300
C	1.60621900	-1.24642800	-1.70276700
H	1.43345700	-2.23453800	-2.15289500
C	0.35416300	-0.41158600	-1.96778000

H	0.01356900	-0.56278900	-2.99027400
C	0.02869400	0.85081100	-1.33588900
C	0.91744300	1.50946500	-0.29367500
C	2.02525600	2.26236100	-0.72400600
C	0.62464400	1.50105000	1.07738100
C	2.82276400	2.95706700	0.18419600
H	2.25749600	2.30504700	-1.78463700
C	1.41960100	2.19922900	1.99007200
H	-0.23724600	0.93802400	1.42361700
C	2.52322900	2.92756300	1.54821100
H	3.67796900	3.52414600	-0.17503400
H	1.17408100	2.16968800	3.04875700
H	3.14441100	3.46758100	2.25796700
C	-0.71101500	1.89000100	-2.13915400
C	-1.34121200	2.96920100	-1.48623700
C	-0.75015200	1.88470000	-3.54534400
C	-2.00848000	3.96458700	-2.19726800
H	-1.29230400	3.02704800	-0.40327400
C	-1.41610800	2.88201800	-4.26142300
H	-0.23452200	1.10529600	-4.09731100
C	-2.05745000	3.92510700	-3.59394200
H	-2.48516000	4.77969300	-1.65796300
H	-1.41984900	2.84667800	-5.34836800
H	-2.57200500	4.70372900	-4.15059400
C	0.99373700	-2.65889500	1.51515900
H	-0.00275900	-2.74656100	1.95818200
H	1.69993500	-2.31928000	2.27553700
C	1.45984900	-3.95058200	0.89082000
C	2.56131700	-4.61814200	1.43776100

C	0.80323200	-4.51347100	-0.21305500
C	2.99086000	-5.83644300	0.90662900
H	3.09100400	-4.17590700	2.27784000
C	1.23930200	-5.72293300	-0.75294800
H	-0.04022900	-3.99025100	-0.65382200
C	2.33100100	-6.39098000	-0.19032400
H	3.84697200	-6.34391900	1.34294500
H	0.72589700	-6.14795000	-1.61172900
H	2.66705000	-7.33559200	-0.60965400
O	0.81671500	-1.56090600	0.57193400

3. Supplementary References

[S1] Rakshit, S.; Grohmann, C.; Besset, T.; Glorius, F., Rh(III)-Catalyzed Directed C−H Olefination Using an Oxidizing Directing Group: Mild, Efficient, and Versatile. *J. Am. Chem. Soc.* **2011,** *133,* 2350-2353.

[S2] Chowdhury, D.; Koner, M.; Ghosh, S.; Baidya, M., Regioselective Annulation of Allenylphosphine Oxides with Aromatic Amides under Ruthenium(II) Catalysis. *Org. Lett.* **2022,** *24,* 3604-3608.

[S3] Xu, Y.; Zheng, G.; Yang, X.; Li, X., Rhodium(iii)-catalyzed chemodivergent annulations between N-methoxybenzamides and sulfoxonium ylides via C-H activation. *Chem. Commun.* **2018,** *54,* 670-673.

[S4] Xue, J.-W.; Zeng, M.; Jiang, H.; Li, K.; Chen, Z.; Yin, G., Palladium(II)/Lewis Acid-Catalyzed Oxidative Olefination/Annulation of N-Methoxybenzamides: Identifying the Active Intermediates through NMR Characterizations. *J. Org. Chem.* **2020,** *85,* 8760-8772.

[S5] Wang, C.-Q.; Ye, L.; Feng, C.; Loh, T.-P., C-F Bond Cleavage Enabled Redox-Neutral [4+1] Annulation via C-H Bond Activation. *J. Am. Chem. Soc.* **2017,** *139,* 1762-

1765.

[S6] Huang, Q.; Chen, Y.; Zhou, X.; Dai, L.; Lu, Y., Nickel-Hydride-Catalyzed Diastereo- and Enantioselective Hydroalkylation of Cyclopropenes. *Angew. Chem. Int. Ed.* **2022,** *61*, e202210560.

[S7] Ramachandran, K.; Anbarasan, P., Cobalt-catalyzed multisubstituted allylation of the chelation-assisted C-H bond of (hetero)arenes with cyclopropenes. *Chem. Sci.* **2021,** *12,* 13442-13449.

[S8] Meyer, S.; Göbel, L.; Livingstone, K.; Roblick, C.; Daniliuc, C. G.; Gilmour, R., Cyclopropene activation via I(I)/I(III) catalysis: Proof of principle and application in direct tetrafluorination. *Tetrahedron.* **2022,** *126*, 132925.

[S9] Smyrnov, V.; Muriel, B.; Waser, J., Synthesis of Quinolines via the Metal-free Visible-Light-Mediated Radical Azidation of Cyclopropenes. *Org. Lett.* **2021,** *23,* 5435-5439.

[S10] Zhang, Y.; Li, Y.; Zhou, W.; Zhang, M.; Zhang, Q.; Jia, R.; Zhao, J., Assembly of polysubstituted chiral cyclopropylamines via highly enantioselective Cu-catalyzed three-component cyclopropene alkenylamination. *Chem. Commun.* **2020,** *56,* 12250-12253.

[S11] Qin, W.-W.; Sang, C.-Y.; Zhang, L.-L.; Wei, W.; Tian, H.-Z.; Liu, H.-X.; Chen, S.-W.; Hui, L., Synthesis and biological evaluation of 2,4-diaminopyrimidines as selective Aurora A kinase inhibitors. *Eur. J. Med. Chem.* **2015,** *95,* 174-184.

[S12] Matthew R. Smith, J. Y. K., Marco A. Ciufolini Pd-arylurea complexes for the Heck arylation of crotonic and cinnamic substrates. *Tetrahedron Lett.* **2013,** *54,* 2042-2045.

[S13] Wu, X.; Zhou, J., Selective arylation at the vinylic site of cyclic olefins. *Chem. Commun.* **2013,** *49,* 4794-4796.

[S14] Zhou, T.; Li, L.; Li, B.; Song, H.; Wang, B. Syntheses, Structures, and Reactions of Cyclometalated Rhodium, Iridium, and Ruthenium Complexes of N-Methoxy-4-nitrobenzamide. *Organometallics* **2018**, *37*, 476−481.

[S15] Zhu, H.-T.; Fan, M.-J.; Yang, D.-S.; Wang, X.-L.; Ke, S.; Zhang, C.-Y.; Guan, Z.-

H. An iodine-promoted Meyer–Schuster rearrangement for the synthesis of α-iodo unsaturated ketones. *Org. Chem. Front.* **2015**, *2*, 506-509.

[S16] Frisch, M. J. T.; G. W.; Schlegel, H. B.; Scuseria, G. E.; Robb, M. A.; Cheeseman, J. R.; Scalmani, G.; Barone, V.; Mennucci, B.; Petersson, G. A.; Nakatsuji, H.; Caricato, M.; Li, X.; Hratchian, H. P.; Izmaylov, A. F.; Bloino, J.; Zheng, G.; Sonnenberg, J. L.; Hada, M.; Ehara, M.; Toyota, K.; Fukuda, R.; Hasegawa, J.; Ishida, M.; Nakajima, T.; Honda, Y.; Kitao, O.; Nakai, H.; Vreven, T.; Montgomery, J. A.; Peralta, Jr., J. E.; Ogliaro, F.; Bearpark, M.; Heyd, J. J.; Brothers, E.; Kudin, K. N.; Staroverov, V. N.; Keith, T.; Kobayashi, R.; Normand, J.; Raghavachari, K.; Rendell, A.; Burant, J. C.; Iyengar, S. S.; Tomasi, J.; Cossi, M.; Rega, N.; Millam, J. M.; Klene, M.; Knox, J. E.; Cross, J. B.; Bakken, V.; Adamo, C.; Jaramillo, J.; Gomperts, R.; Stratmann, R. E.; Yazyev, O.; Austin, A. J.; Cammi, R.; Pomelli, C.; Ochterski, J. W.; Martin, R. L.; Morokuma, K.; Zakrzewski, V. G.; Voth, G. A.; Salvador, P.; Dannenberg, J. J.; Dapprich, S.; Daniels, A. D.; Farkas, O.; Foresman, J. B.; Ortiz, J. V.; Cioslowski J.; Fox, D. J. Gaussian 09, revision D. 01, Gaussian, Inc., Wallingford CT, 2013.

[S17] (a) Lee, C.; Yang, W.; Parr, R. G. Development of the Colle-Salvetti Correlation-Energy Formula into a Functional of the Electron-Density. Phys. Rev. B: Condens. *Matter Mater. Phys.* **1988,** *37,* 785-789. (b) Becke, A. D. Density-Functional Thermochemistry. III. The Role of Exact Exchange. *J. Chem. Phys.* **1993,** *98,* 5648-5652.

[S18] Andrae, D.; Häußermann, U.; Dolg, M.; Stoll, H.; Preuß, H., Energy-Adjusted ab Initio Pseudo Potentials for the Second and Third Row Transition Elements. *Theor. Chim. Acta.* **1990,** *77,* 123-141.

[S19] (a) Gonzalez, C.; Schlegel, H. B., An Improved Algorithm for Reaction Path Following. *J. Chem. Phys.* **1989,** *90,* 2154-2161. (b) Gonzalez, C.; Schlegel, H. B., Reaction Path Following in Massweighted Internal Coordinates. *J. Phys. Chem.* **1990,** *94,* 5523-5527.

[S20] Marenich, A. V.; Cramer, C. J.; Truhlar, D. G., Universal Solvation Model Based on Solute Electron Density and on a Continuum Model of the Solvent Defined by the

Bulk Dielectric Constant and Atomic Surface Tensions. *J. Phys. Chem. B.* **2009,** *113,* 6378-6396.

[S21] Zhao, Y.; Truhlar, D. G., A New Local Density Functional for Main-Group Thermochemistry, Transition Metal Bonding, Thermochemical Kinetics, and Noncovalent Interactions. *J. Chem. Phys.* **2006,** *125,* 194101.

[S22] Legault, C. Y. CYLview, 1.0b; Université de Sherbrooke, **2009,** (http://www.cylview.org).

4. ^{1}H NMR, ^{13}C NMR, and ^{19}F NMR Spectra

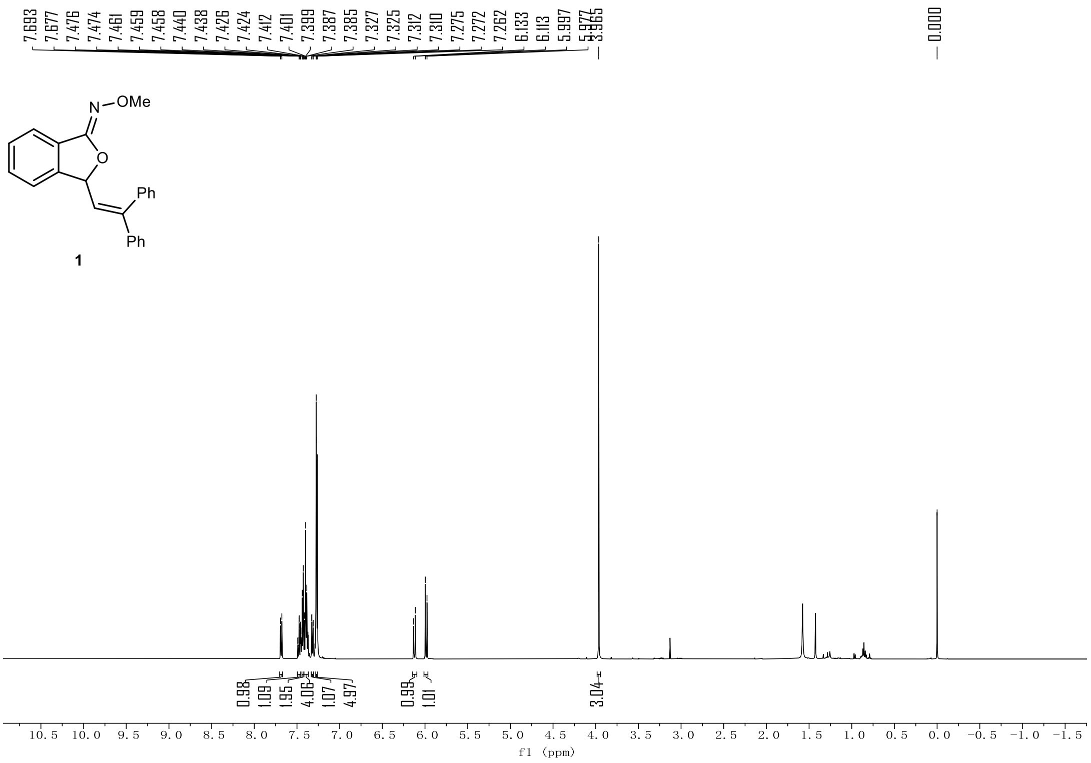

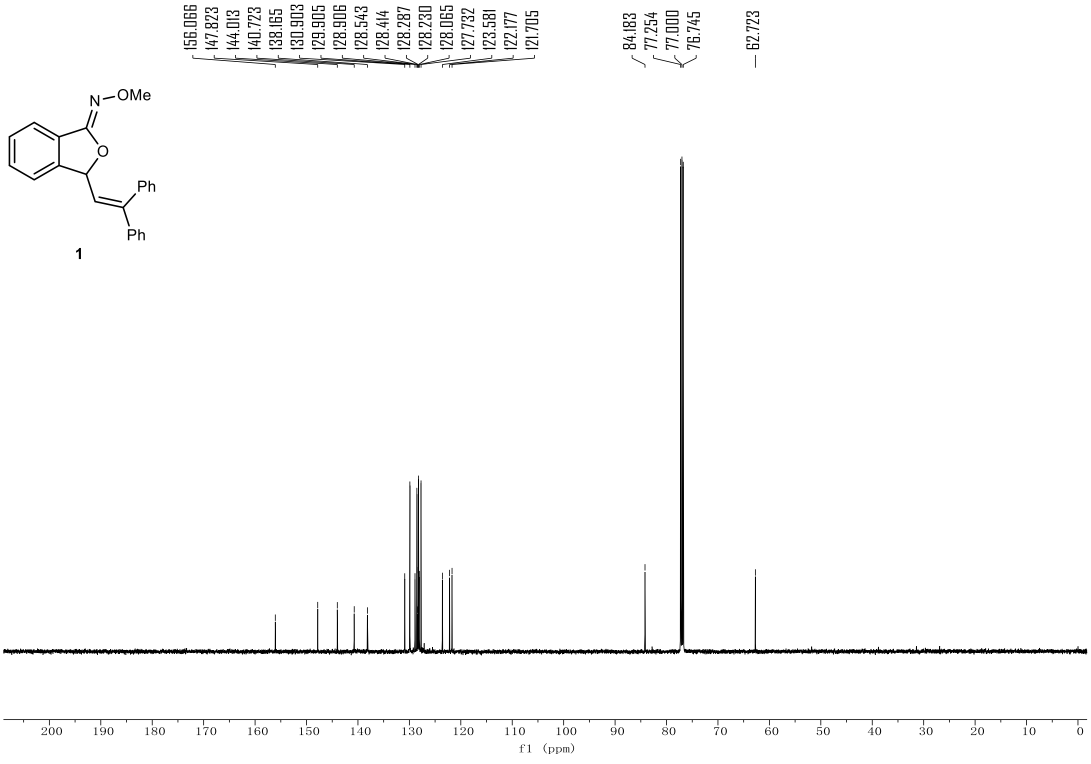

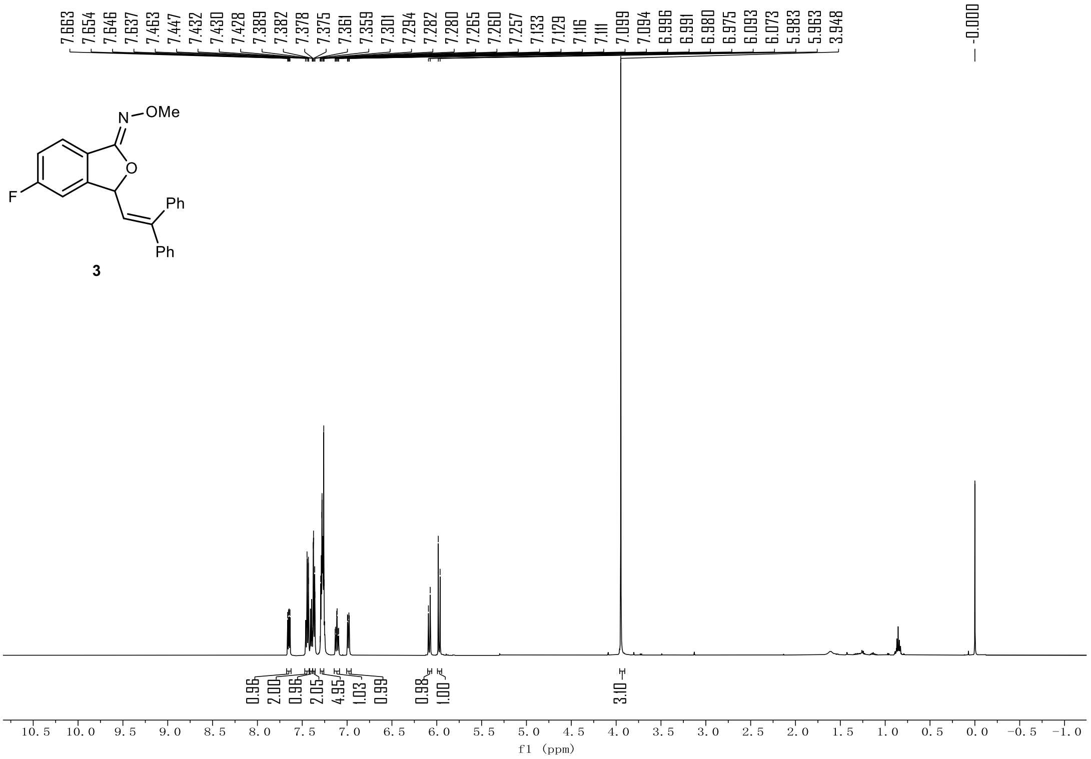
7.663
7.654
7.646
7.637
7.463
7.447
7.432
7.430
7.428
7.389
7.382
7.378
7.375
7.361
7.359
7.301
7.294
7.282
7.280
7.265
7.260
7.257
7.133
7.129
7.116
7.111
7.099
7.094
6.996
6.991
6.980
6.975
6.093
6.073
5.983
5.963
3.948
-0.000
N
OMe
O
F
Ph
Ph
3
0.96
2.00
0.96
2.05
4.95
1.03
0.99
0.98
1.00
3.10
10.5 10.0 9.5 9.0 8.5 8.0 7.5 7.0 6.5 6.0 5.5 5.0 4.5 4.0 3.5 3.0 2.5 2.0 1.5 1.0 0.5 0.0 -0.5 -1.0
f1 (ppm)

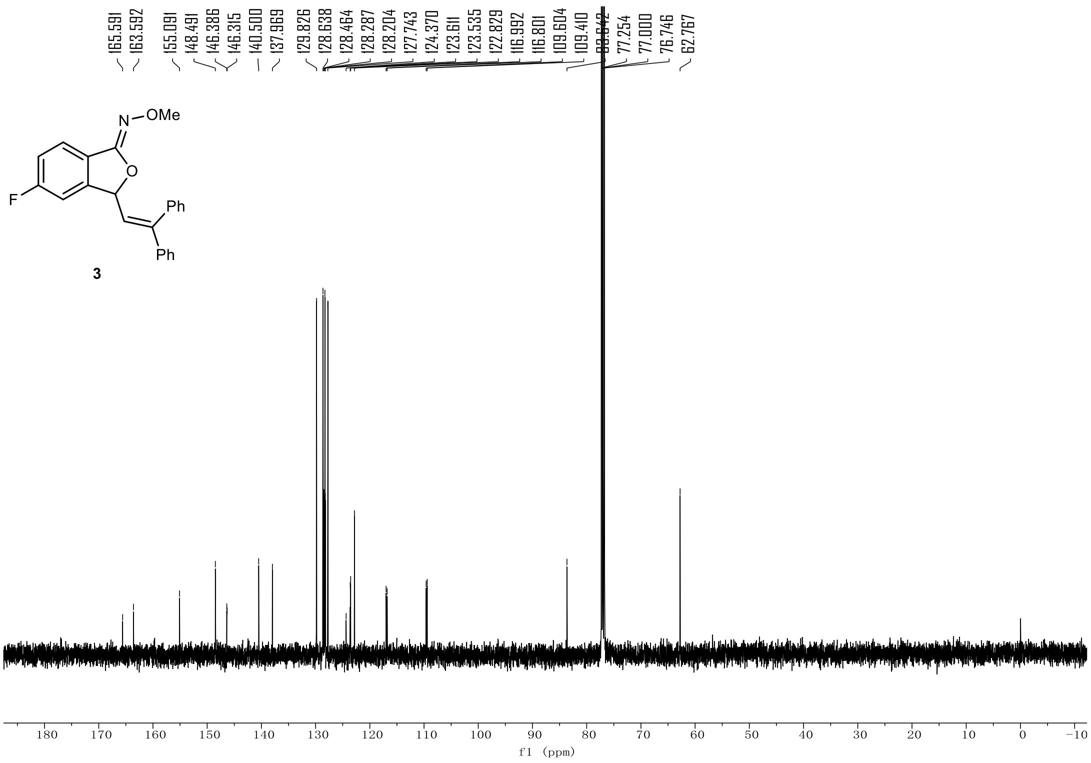
165.591
163.592
155.091
148.491
146.386
146.315
140.500
137.969
129.826
128.638
128.464
128.287
128.204
127.743
124.370
123.611
123.535
122.829
116.992
116.801
109.604
109.410
83.642
77.254
77.000
76.746
62.767
N
OMe
O
F
Ph
Ph
3
180 170 160 150 140 130 120 110 100 90 80 70 60 50 40 30 20 10 0 -10
f1 (ppm)

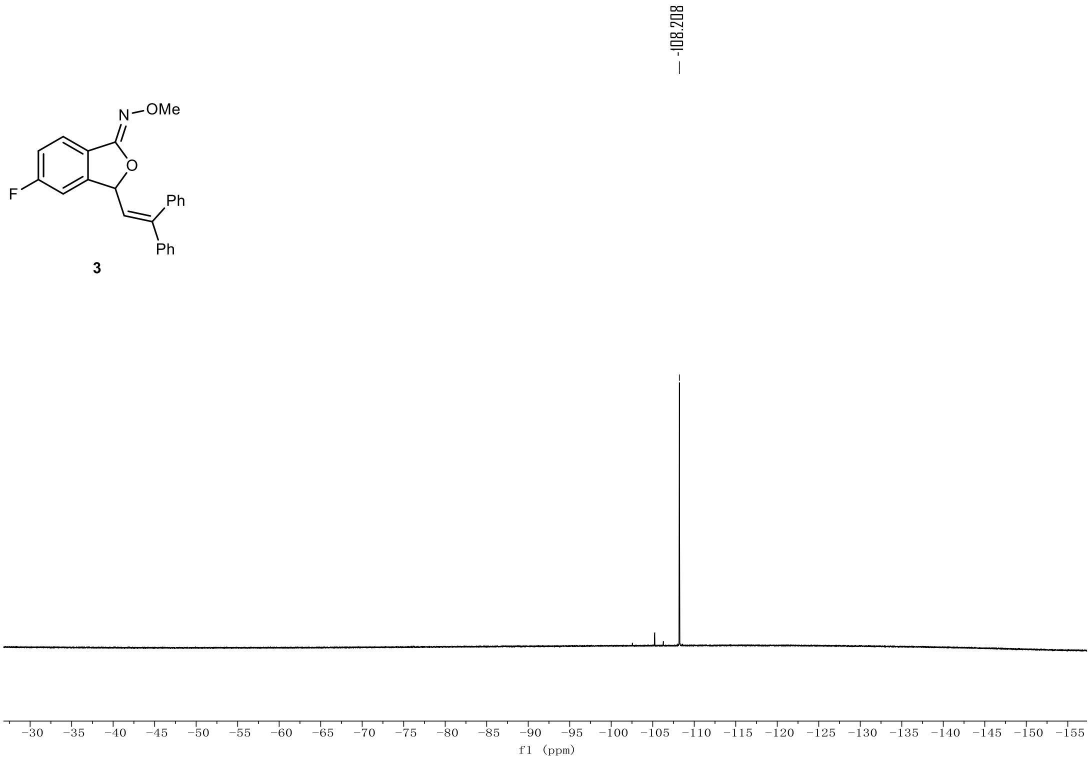
-108.208
N–OMe
O
F
Ph
Ph
3
f1 (ppm)

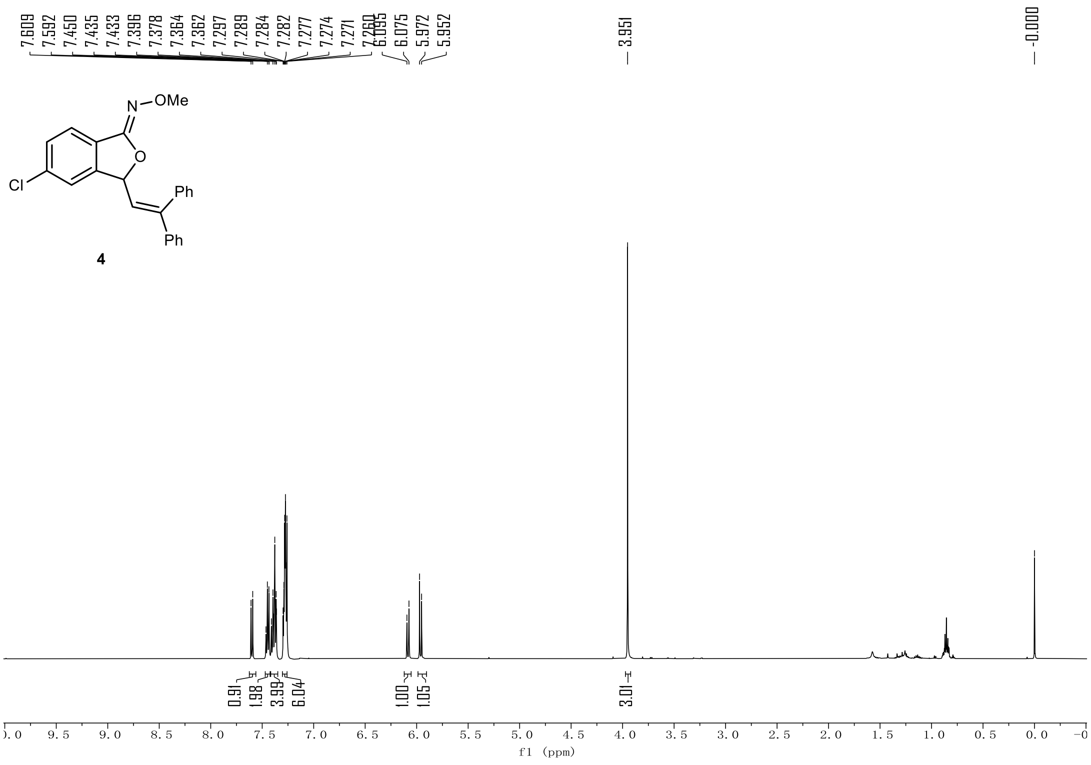
7.609 7.592 7.450 7.435 7.433 7.396 7.378 7.364 7.362 7.297 7.289 7.284 7.282 7.277 7.274 7.271 7.260 6.095 6.075 5.972 5.952
3.951
-0.000
N–OMe
O
Cl
Ph
Ph
4
0.91 1.98 3.99 6.04 1.00 1.05 3.01
f1 (ppm)

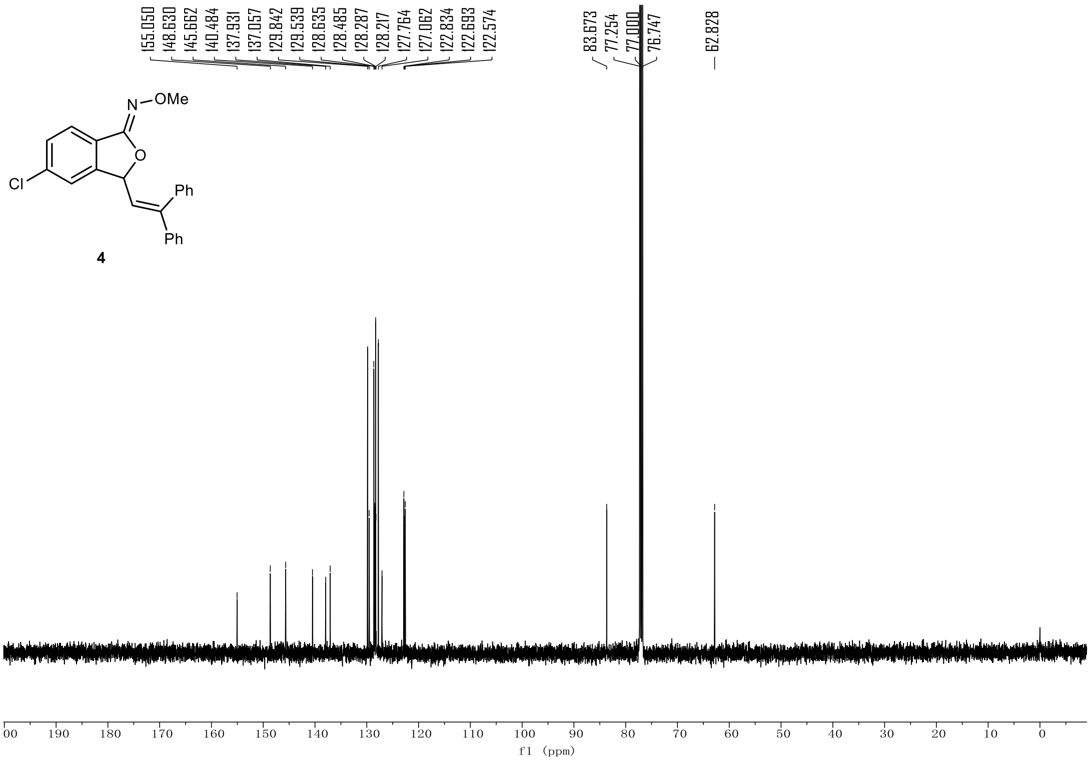
155.050
148.630
145.662
140.484
137.931
137.057
129.842
129.539
128.635
128.485
128.287
128.217
127.764
127.062
122.834
122.693
122.574
83.673
77.254
77.000
76.747
62.828
N
OMe
O
Cl
Ph
Ph
4
f1 (ppm)

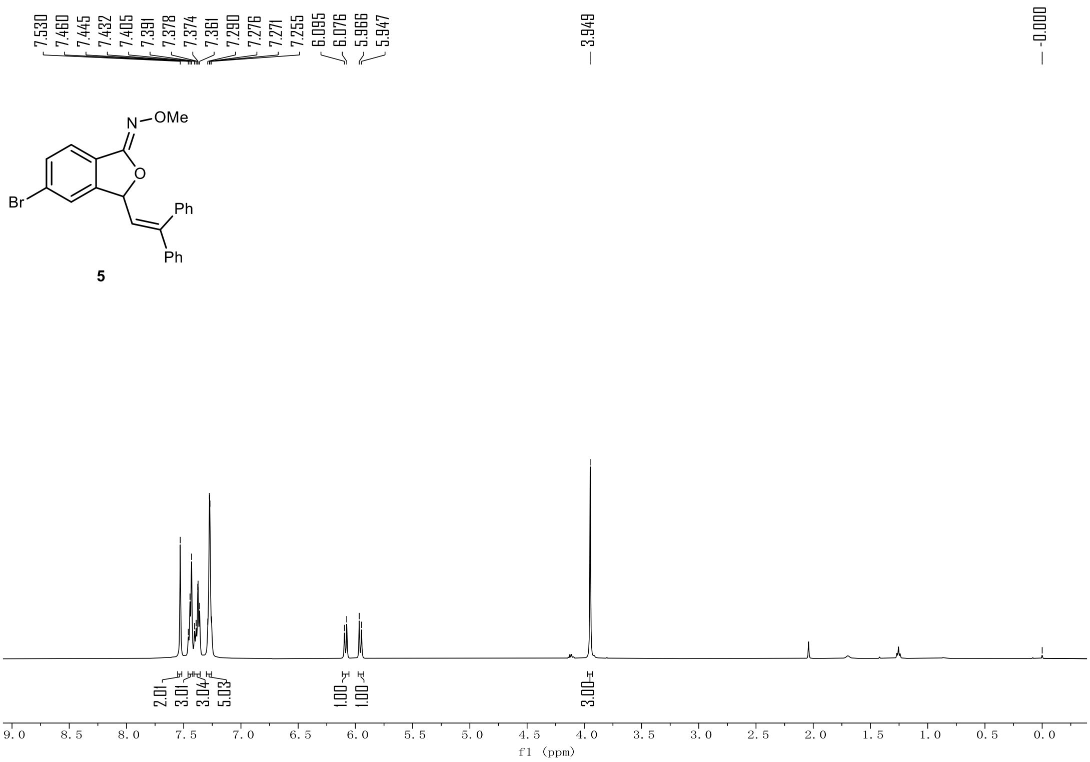
7.530
7.460
7.445
7.432
7.405
7.391
7.378
7.374
7.361
7.290
7.276
7.271
7.255
6.095
6.076
5.966
5.947
3.949
-0.000
N
OMe
O
Br
Ph
Ph
5
2.01
3.01
3.04
5.03
1.00
1.00
3.00
f1 (ppm)

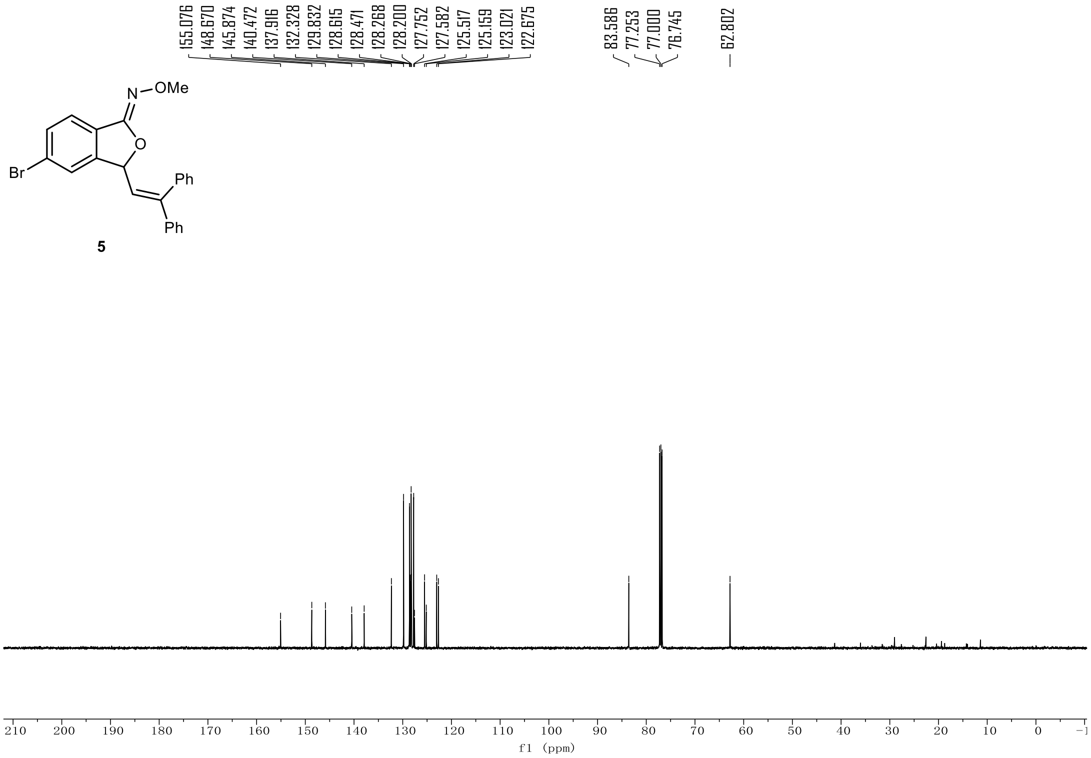
155.076
148.670
145.874
140.472
137.916
132.328
129.832
128.615
128.471
128.268
128.200
127.752
127.582
125.517
125.159
123.021
122.675
83.586
77.253
77.000
76.745
62.802
N
OMe
O
Br
Ph
Ph
f1 (ppm)

5

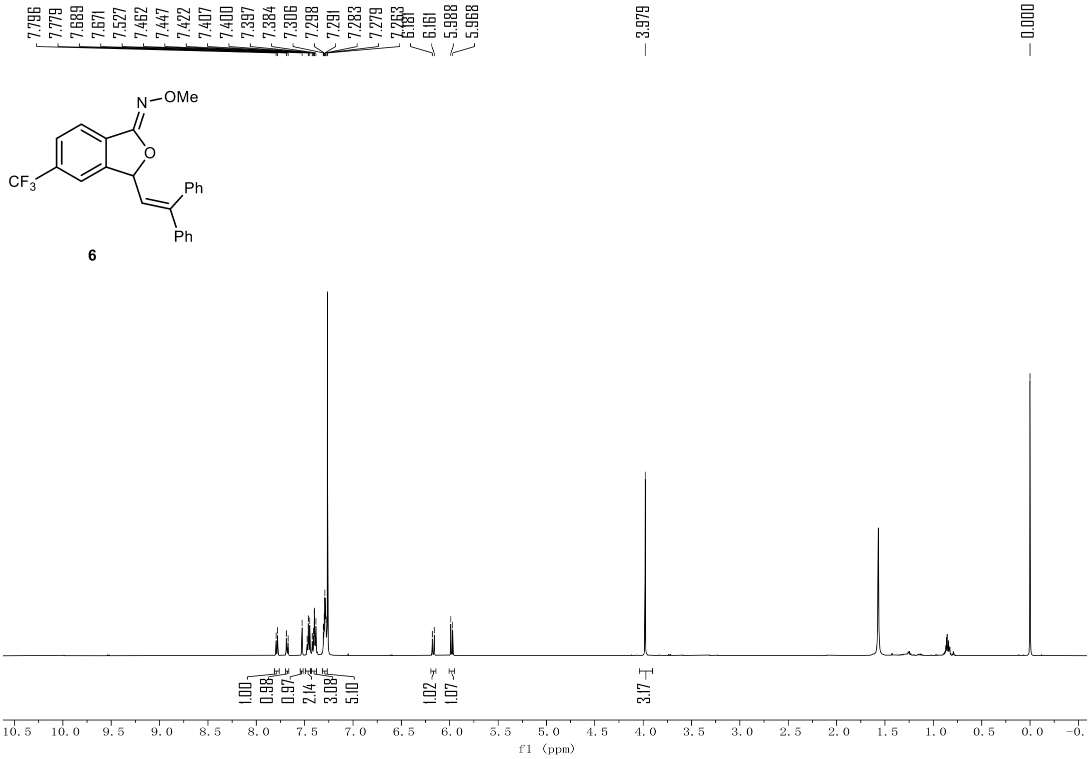
7.796
7.779
7.689
7.671
7.527
7.462
7.447
7.422
7.407
7.400
7.397
7.384
7.306
7.298
7.291
7.283
7.279
7.263
6.181
6.161
5.988
5.968
3.979
0.000
N
OMe
O
CF3
Ph
Ph
1.00
0.98
0.97
2.14
3.08
5.10
1.02
1.07
3.17
f1 (ppm)

6

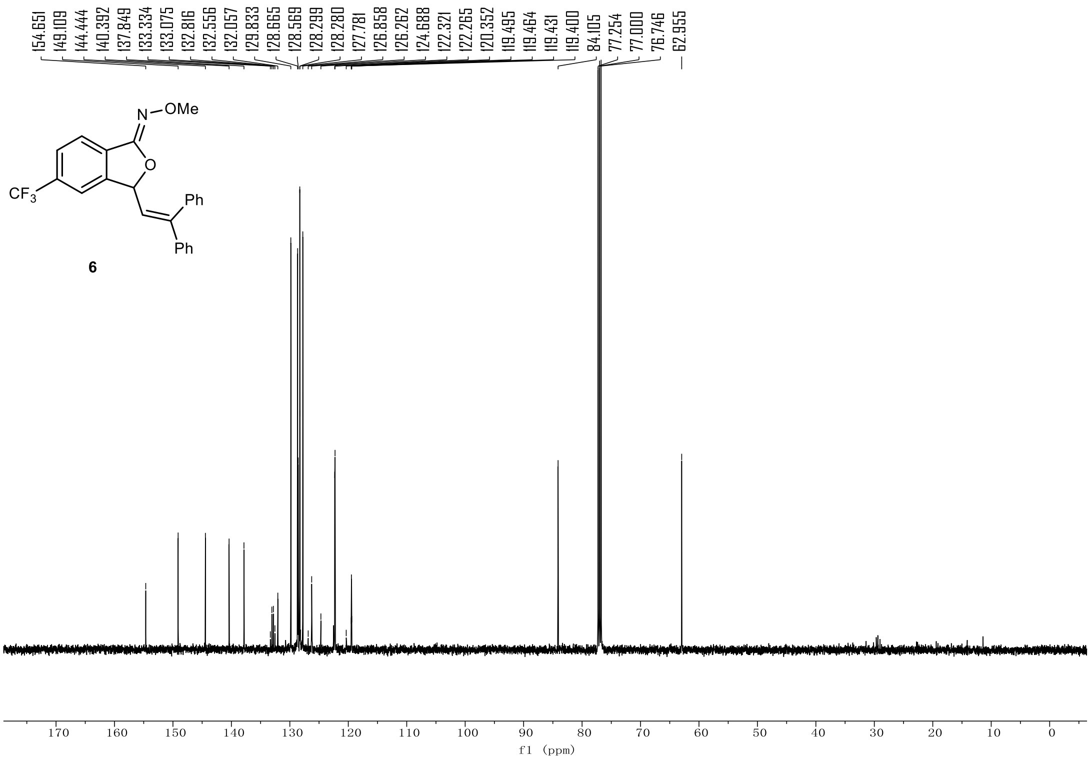

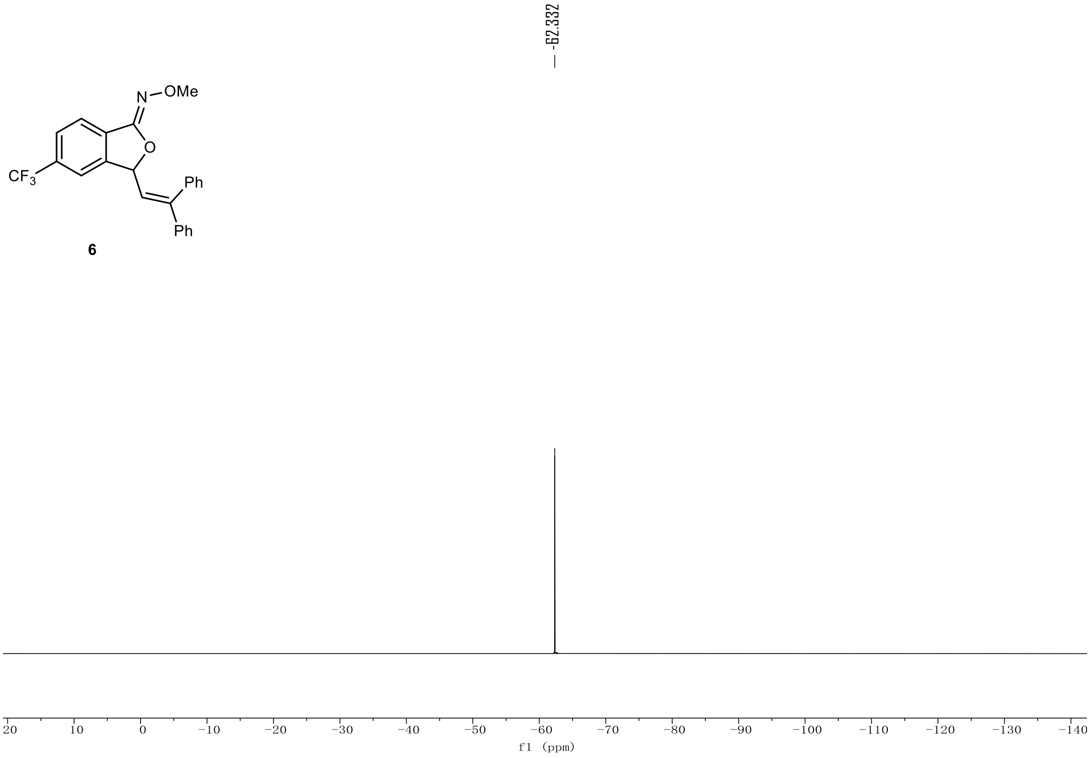

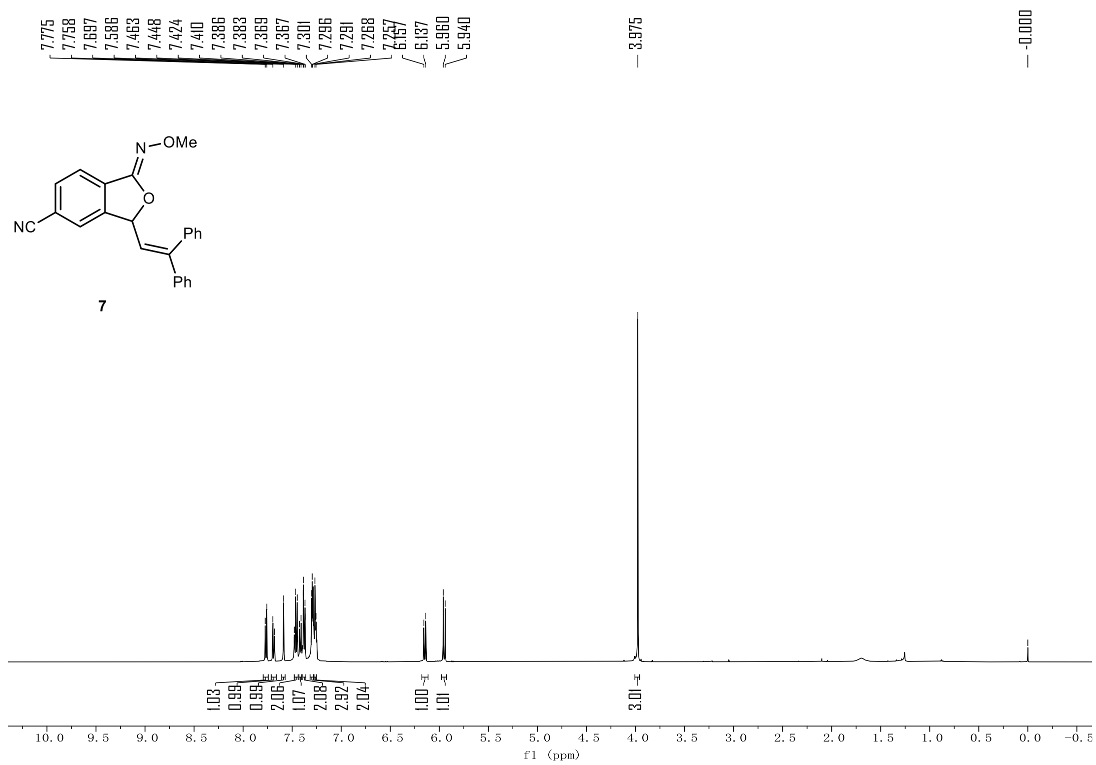

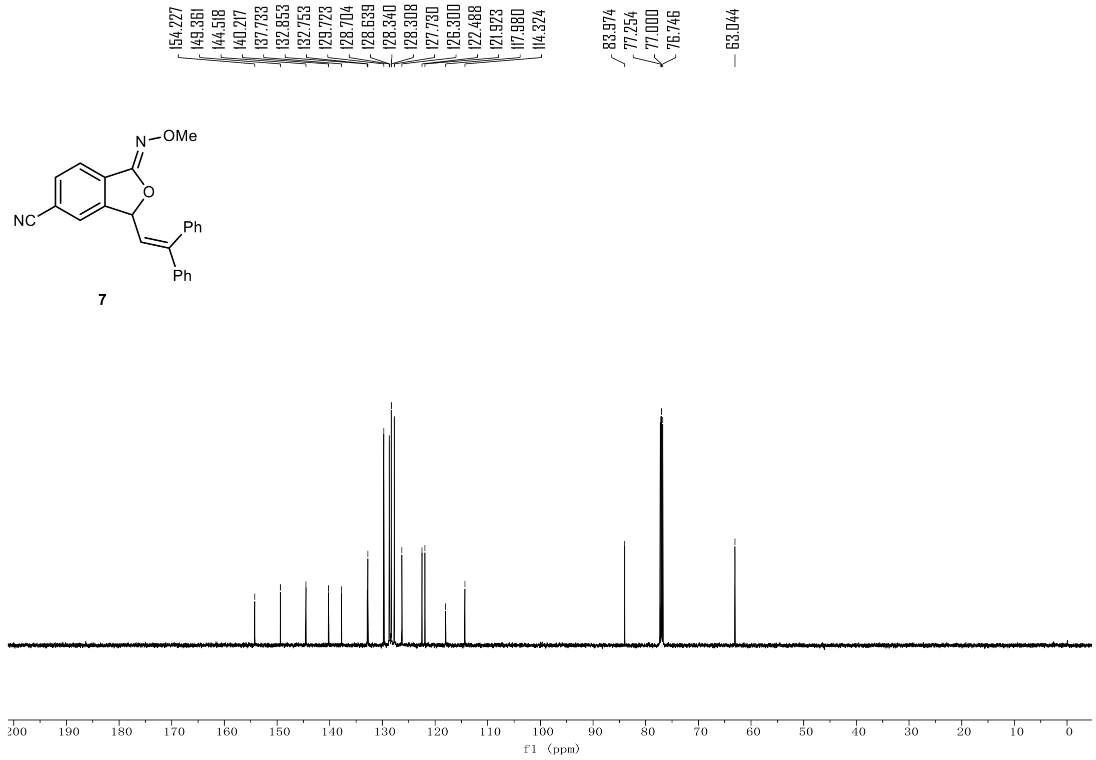

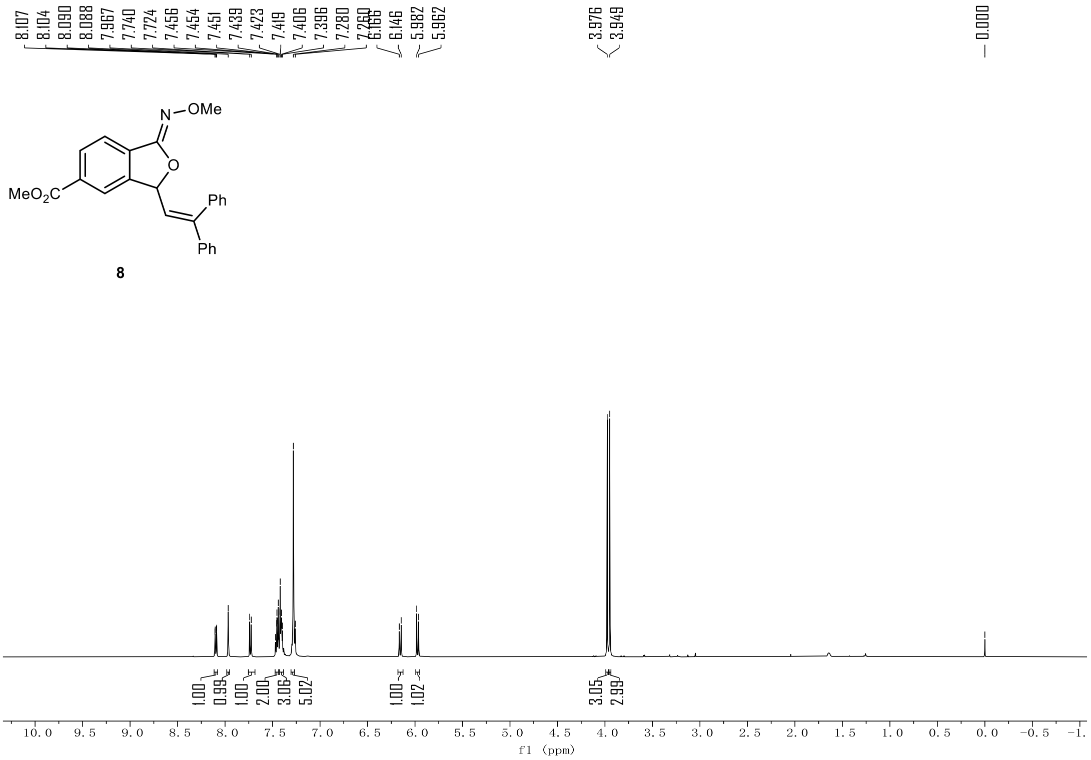

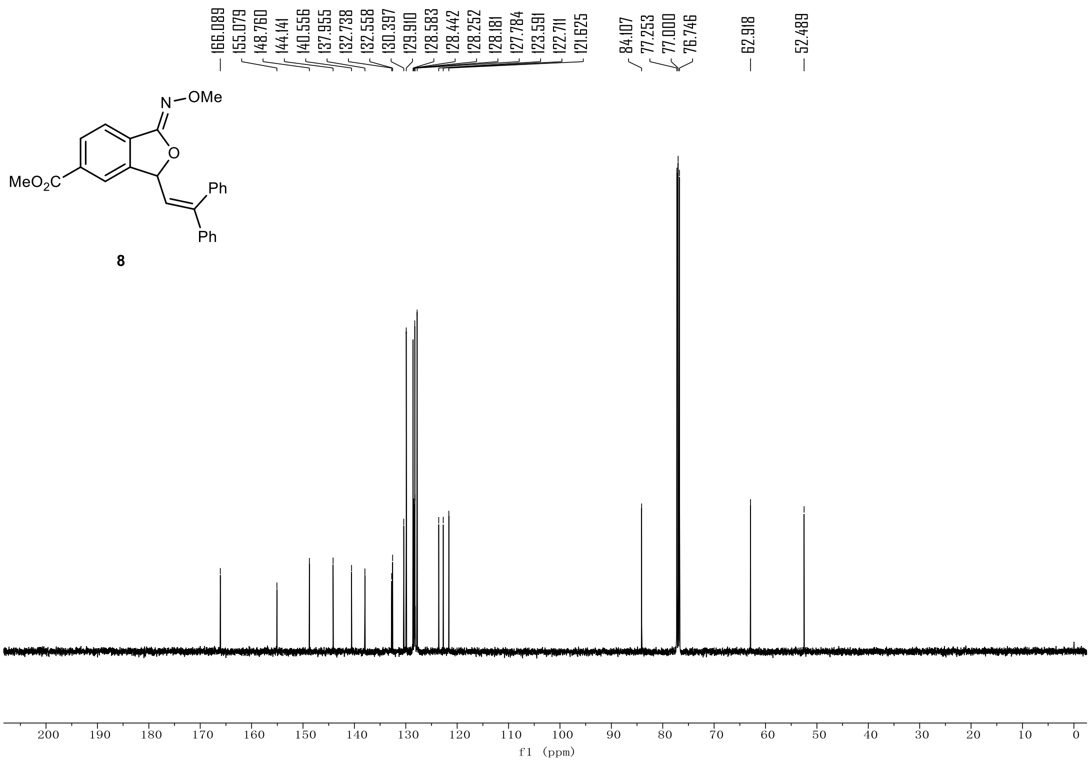

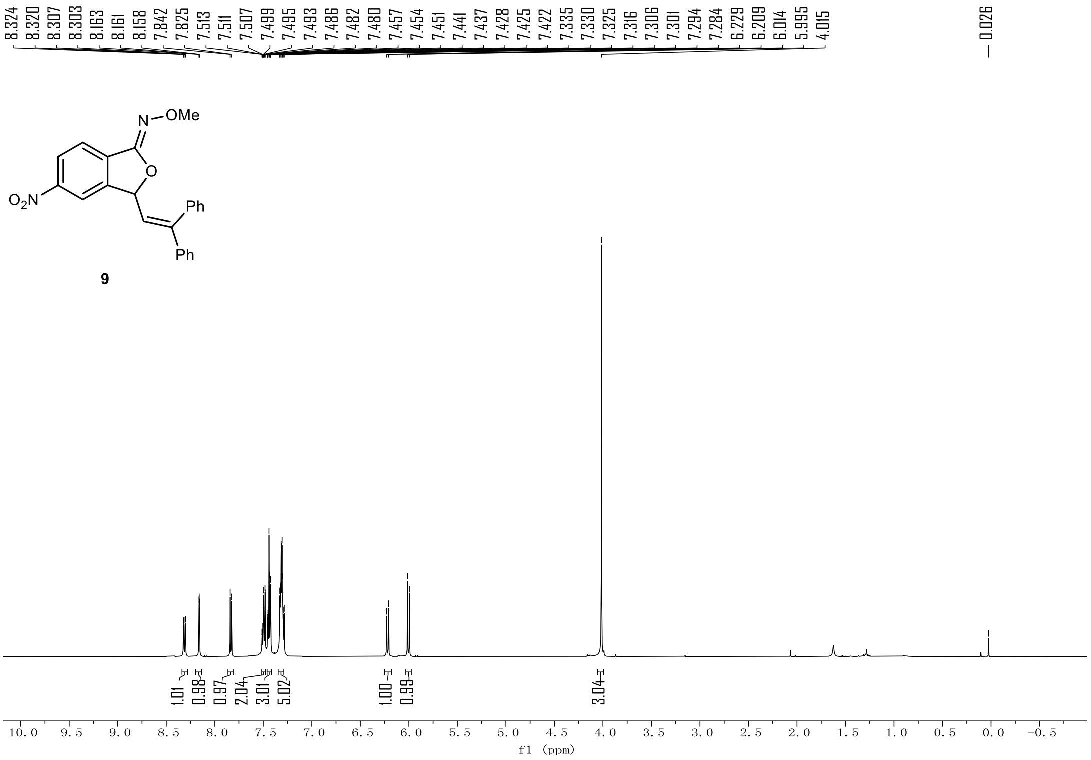
8.324 8.320 8.307 8.303 8.163 8.161 8.158 7.842 7.825 7.513 7.511 7.507 7.499 7.495 7.493 7.486 7.482 7.480 7.457 7.454 7.451 7.441 7.437 7.428 7.425 7.422 7.335 7.330 7.325 7.316 7.306 7.301 7.294 7.284 6.229 6.209 6.014 5.995 4.015
0.026
N
OMe
O
O2N
Ph
Ph
9
1.01 0.98 0.97 2.04 3.01 5.02 1.00 0.99 3.04
f1 (ppm)

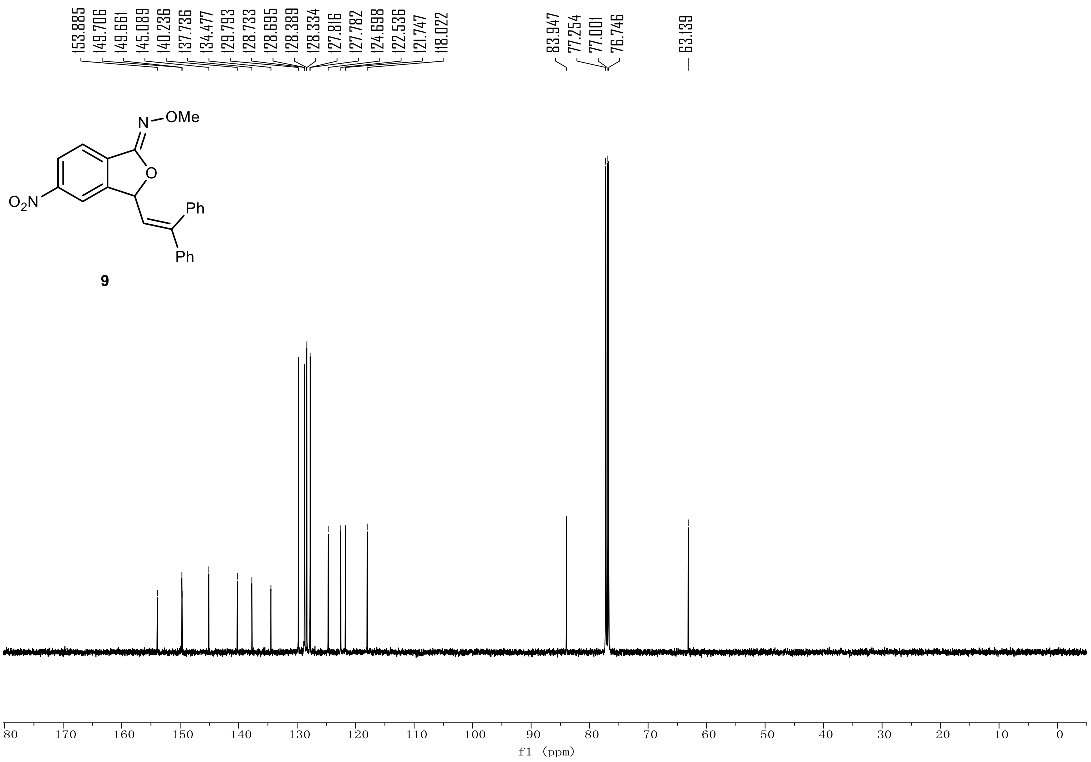
153.885 149.706 149.661 145.089 140.236 137.736 134.477 129.793 128.733 128.695 128.389 128.334 127.816 127.782 124.698 122.536 121.747 118.022
83.947 77.254 77.001 76.746
63.139
N
OMe
O
O2N
Ph
Ph
9
f1 (ppm)

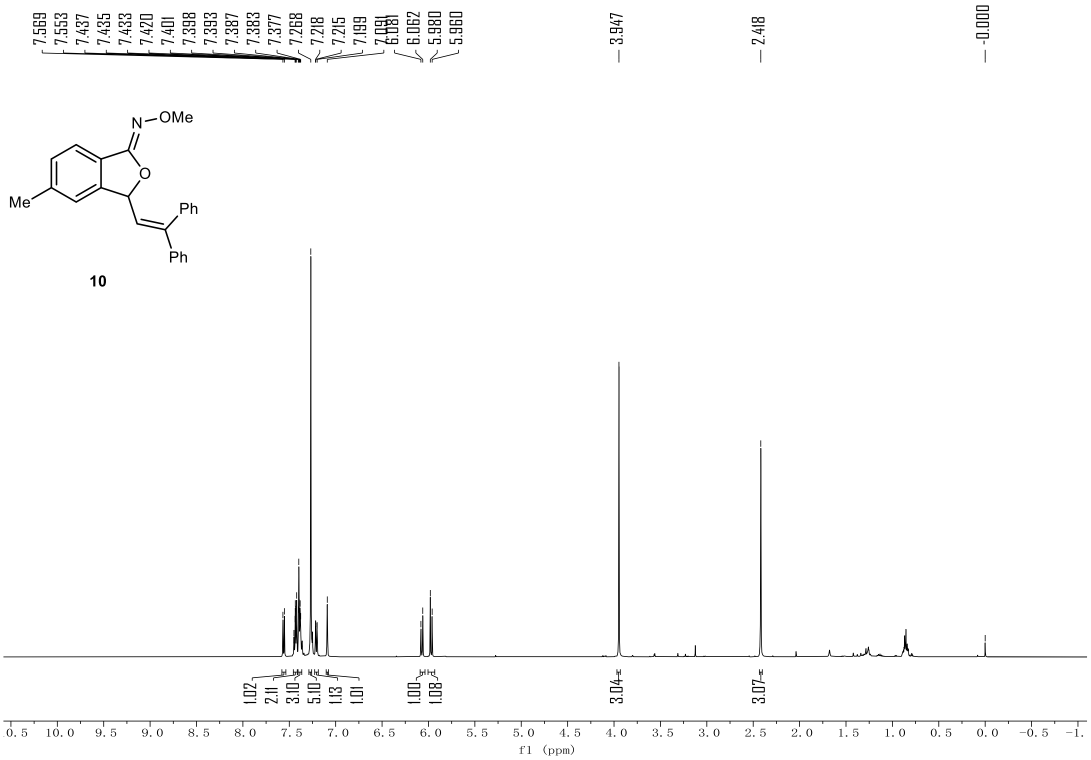
7.569
7.553
7.437
7.435
7.433
7.420
7.401
7.398
7.393
7.387
7.383
7.377
7.268
7.218
7.215
7.199
7.091
6.081
6.062
5.980
5.960
3.947
2.418
-0.000
N
OMe
O
Me
Ph
Ph
10
1.02
2.11
3.10
5.10
1.13
1.01
1.00
1.08
3.04
3.07
f1 (ppm)

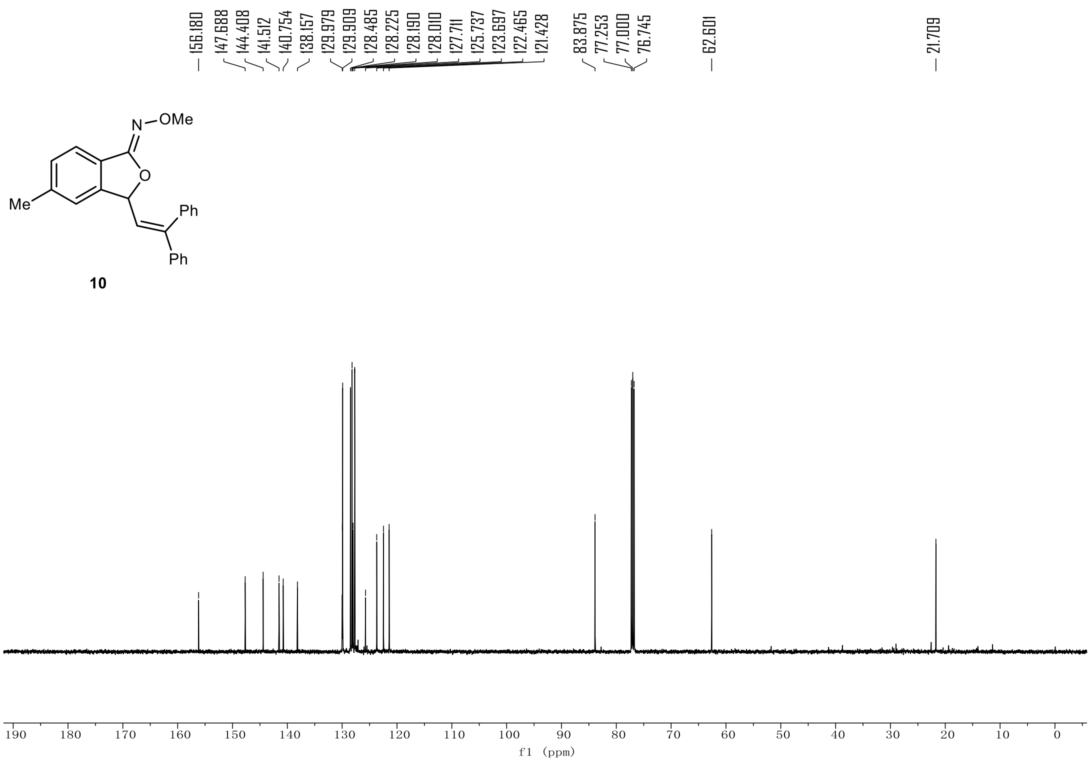
156.180
147.688
144.408
141.512
140.754
138.157
129.979
129.909
128.485
128.225
128.190
128.010
127.711
125.737
123.697
122.465
121.428
83.875
77.253
77.000
76.745
62.601
21.709
N
OMe
O
Me
Ph
Ph
10
f1 (ppm)

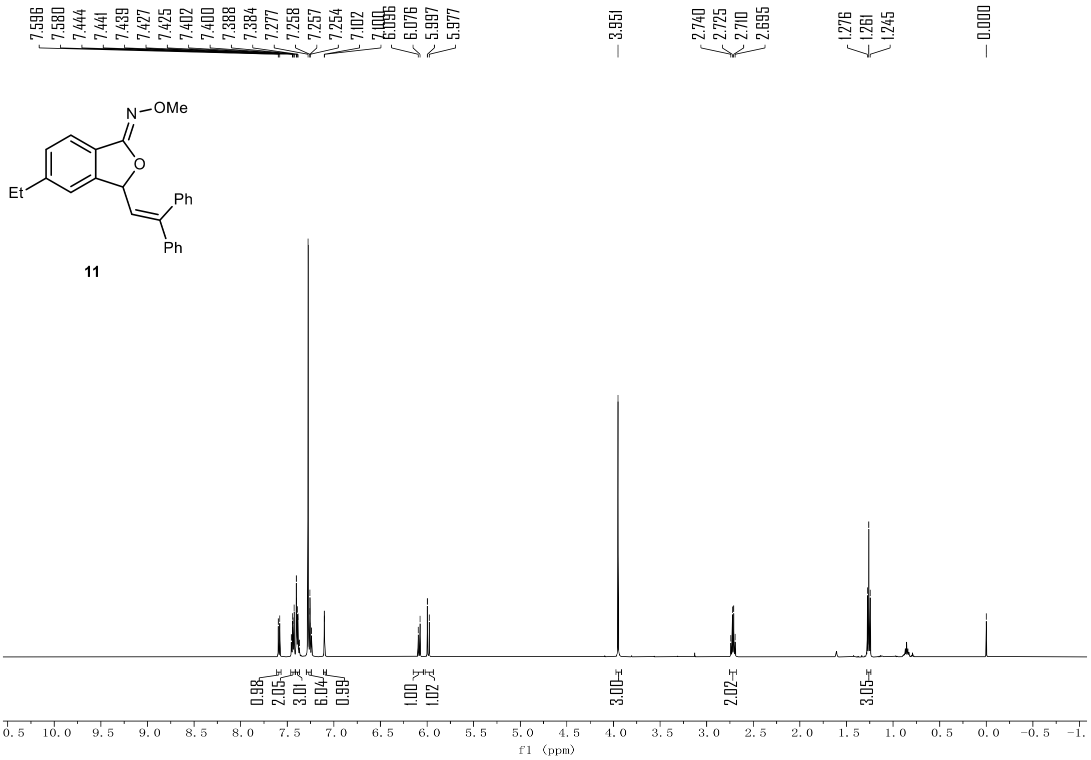
7.596
7.580
7.444
7.441
7.439
7.427
7.425
7.402
7.400
7.388
7.384
7.277
7.258
7.257
7.254
7.102
7.100
6.096
6.076
5.997
5.977
3.951
2.740
2.725
2.710
2.695
1.276
1.261
1.245
0.000
N
OMe
O
Et
Ph
Ph
11
0.98
2.05
3.01
6.04
0.99
1.00
1.02
3.00
2.02
3.05
f1 (ppm)

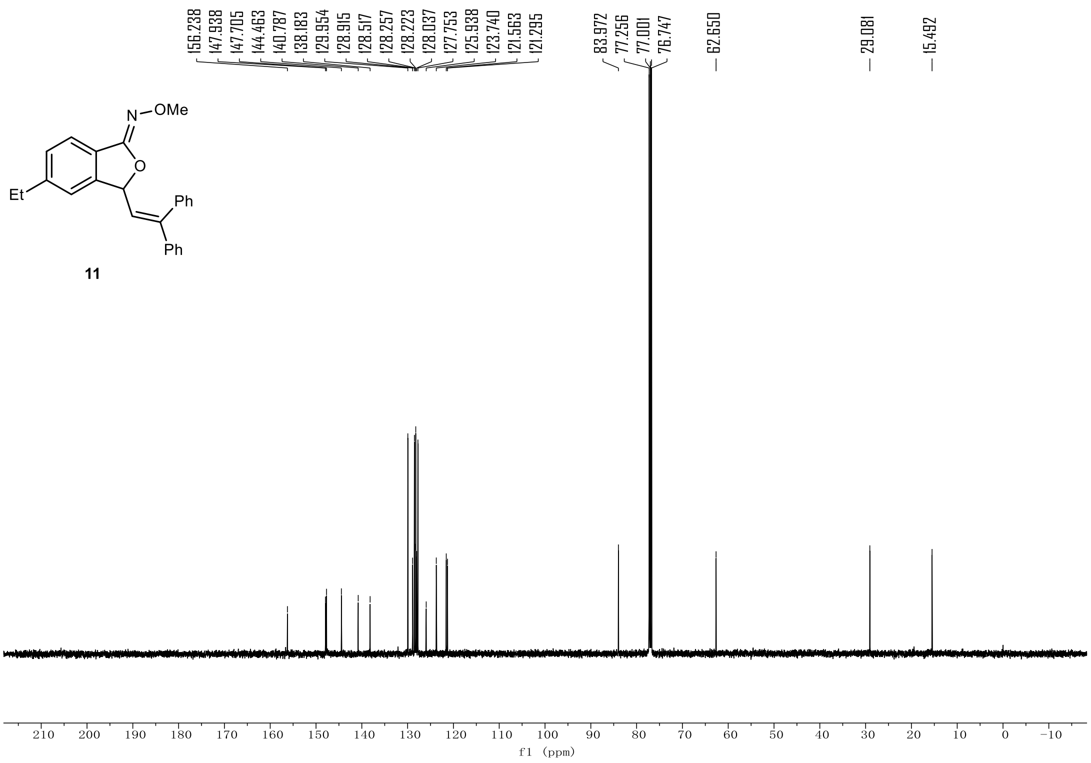
156.238
147.938
147.705
144.463
140.787
138.183
129.954
128.915
128.517
128.257
128.223
128.037
127.753
125.938
123.740
121.563
121.295
83.972
77.256
77.001
76.747
62.650
29.081
15.492
N
OMe
O
Et
Ph
Ph
11
f1 (ppm)

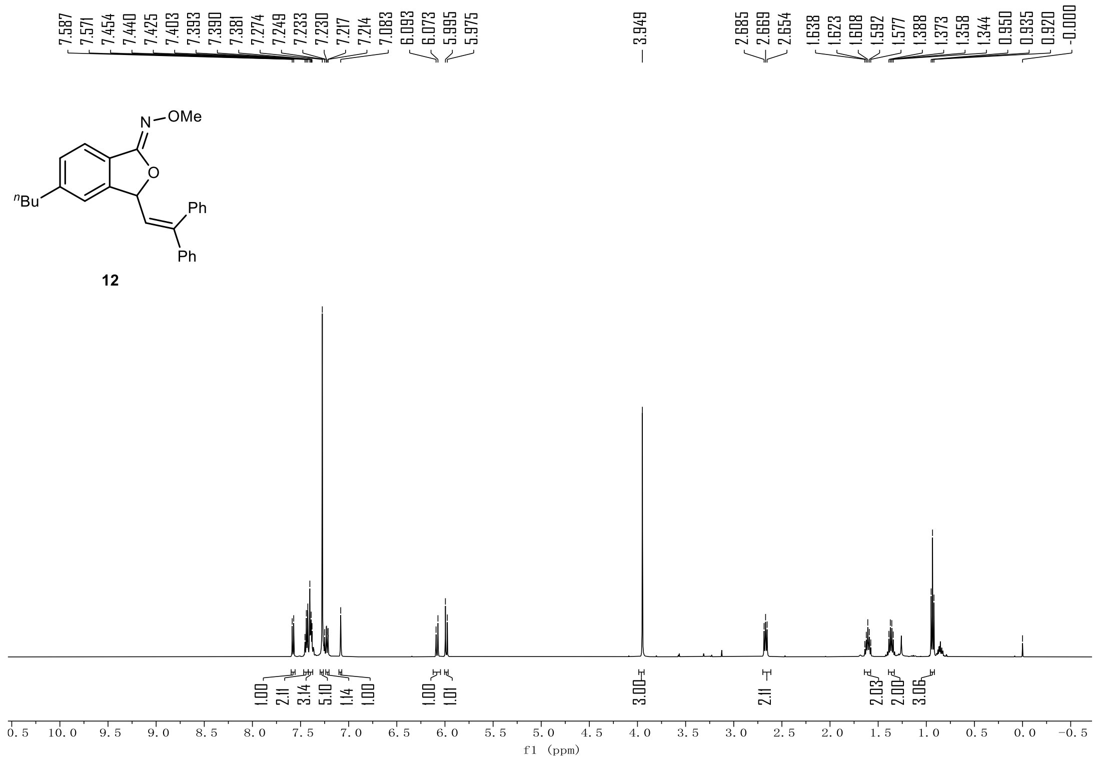
7.587 7.571 7.454 7.440 7.425 7.403 7.393 7.390 7.381 7.274 7.249 7.233 7.230 7.217 7.214 7.083 6.093 6.073 5.995 5.975
3.949
2.685 2.669 2.654 1.638 1.623 1.608 1.592 1.577 1.388 1.373 1.358 1.344 0.950 0.935 0.920 -0.000
N–OMe
O
nBu
Ph
Ph
12
1.00 2.11 3.14 5.10 1.14 1.00 1.00 1.01 3.00 2.11 2.03 2.00 3.06
f1 (ppm)

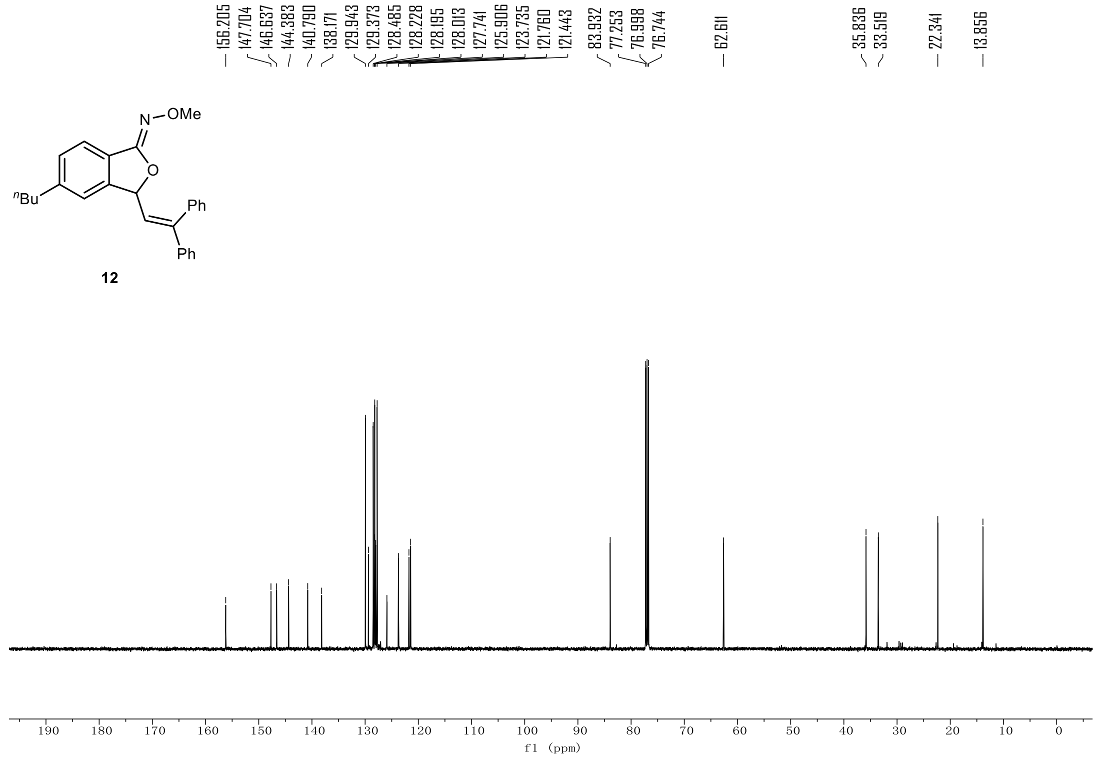
156.205 147.704 146.637 144.383 140.790 138.171 129.943 129.373 128.485 128.228 128.195 128.013 127.741 125.906 123.735 121.760 121.443 83.932 77.253 76.998 76.744 62.611 35.836 33.519 22.341 13.856
N–OMe
O
nBu
Ph
Ph
12
f1 (ppm)

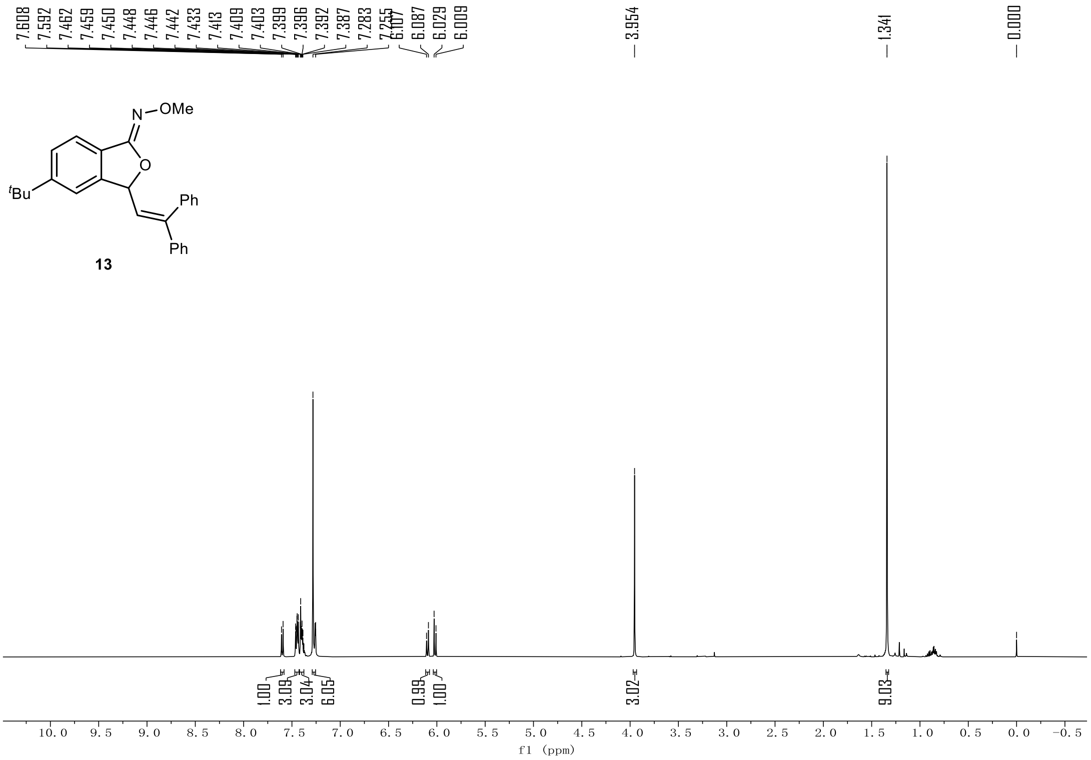

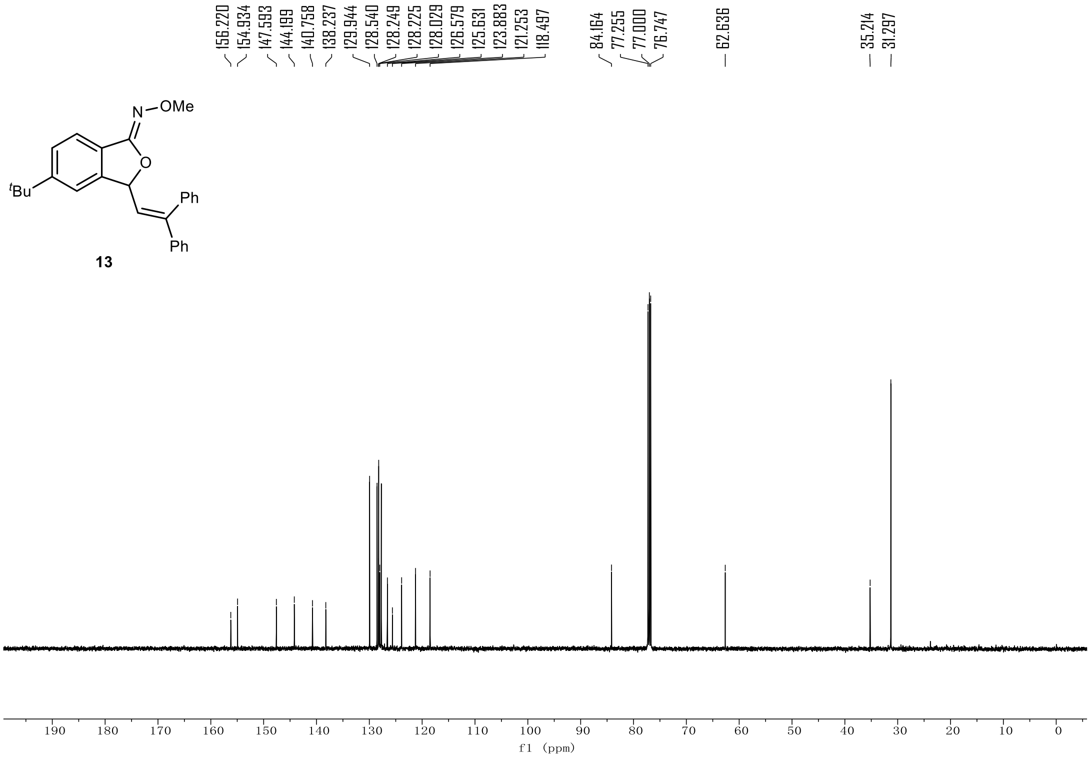

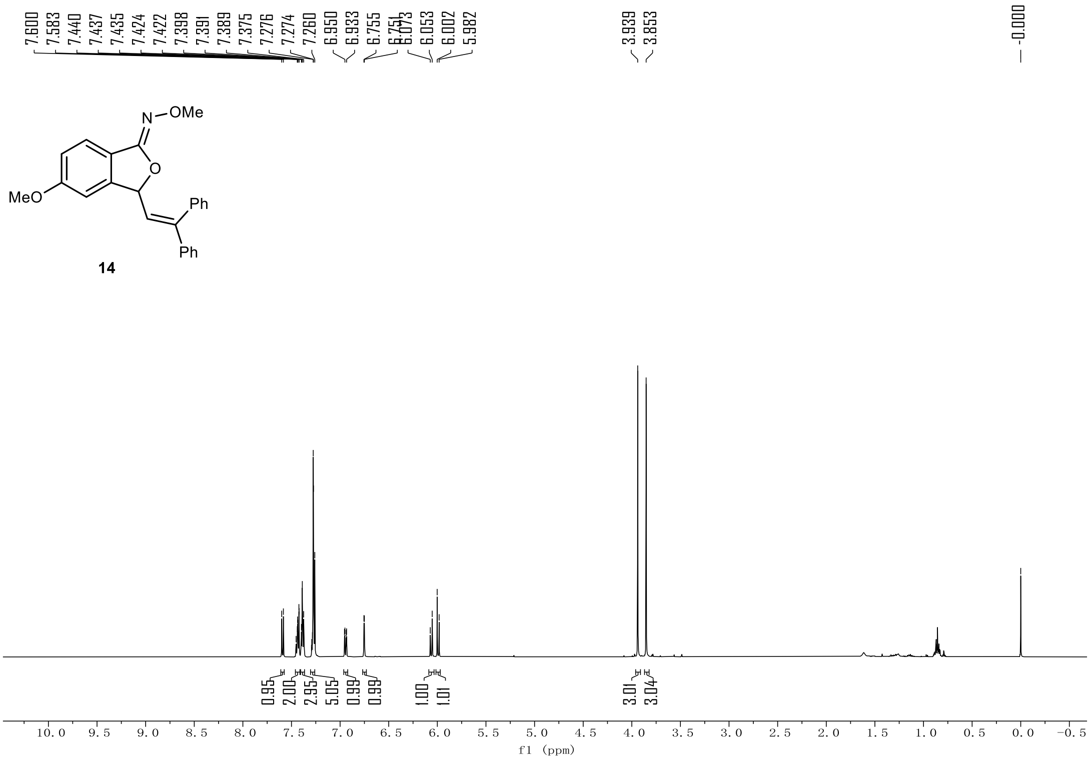

14

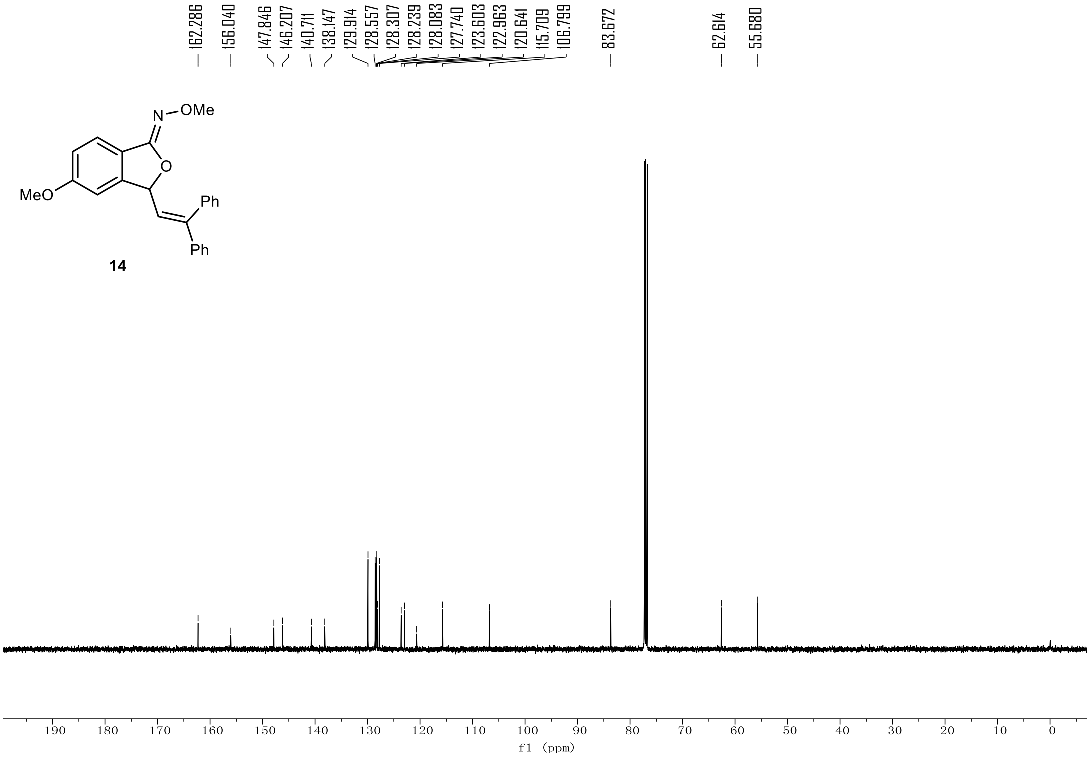

14

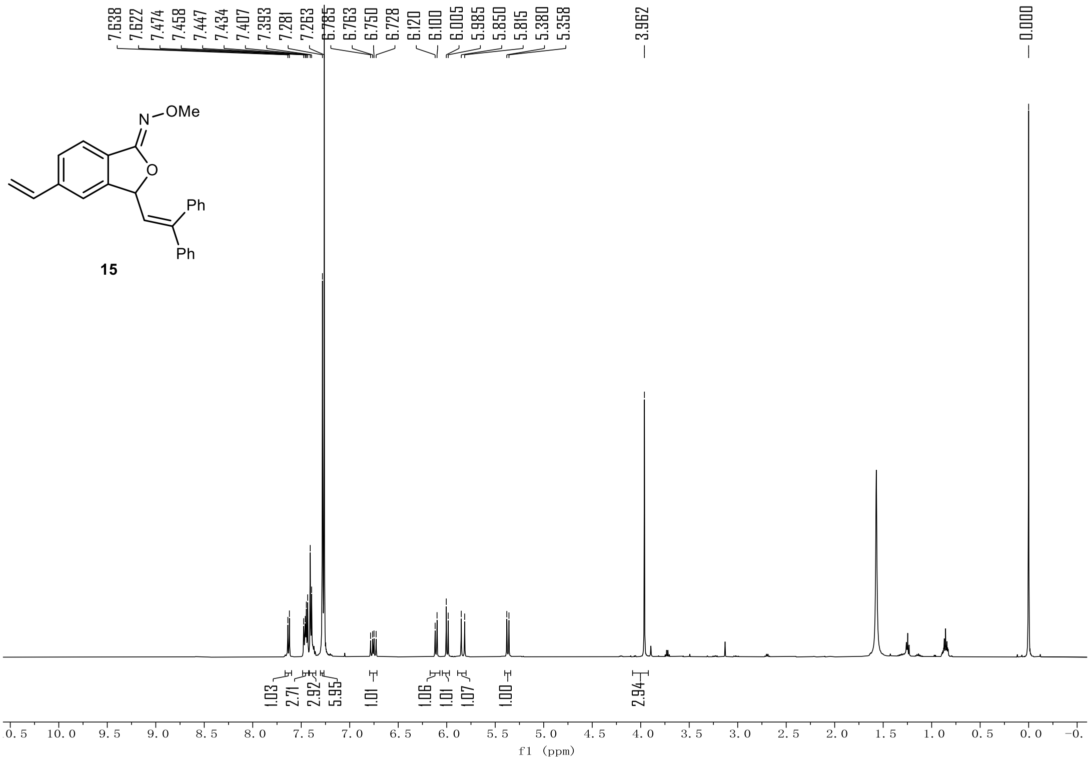
7.638
7.622
7.474
7.458
7.447
7.434
7.407
7.393
7.281
7.263
6.785
6.763
6.750
6.728
6.120
6.100
6.005
5.985
5.850
5.815
5.380
5.358
3.962
0.000
N–OMe
O
Ph
Ph
15
1.03
2.71
2.92
5.95
1.01
1.06
1.01
1.07
1.00
2.94
f1 (ppm)

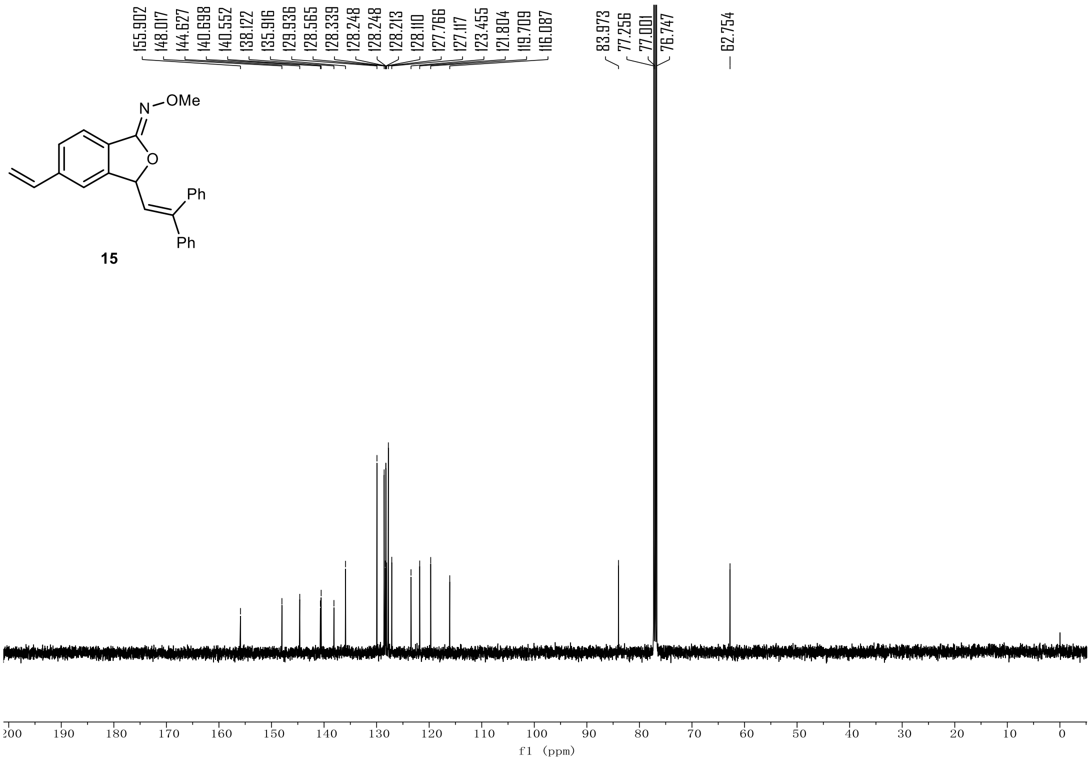
155.902
148.017
144.627
140.698
140.552
138.122
135.916
129.936
128.565
128.339
128.248
128.213
128.110
127.766
127.117
123.455
121.804
119.709
116.087
83.973
77.256
77.001
76.747
62.754
N–OMe
O
Ph
Ph
15
f1 (ppm)

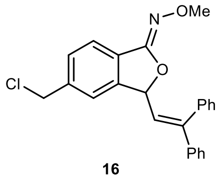

16

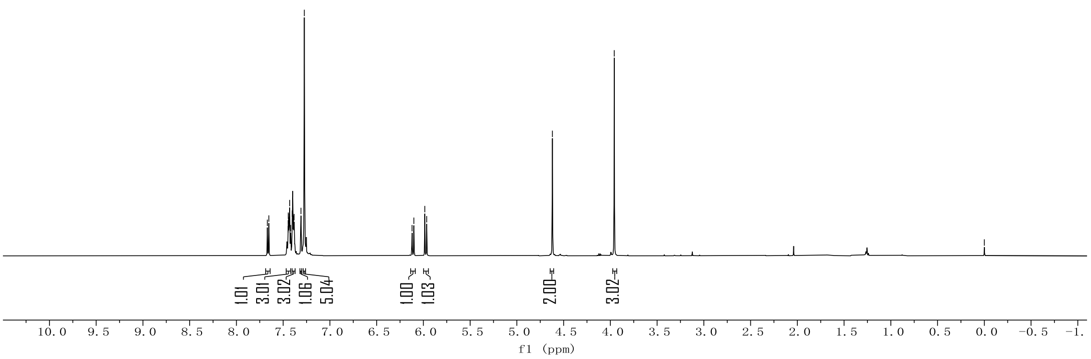
7.669
7.653
7.448
7.445
7.443
7.438
7.435
7.430
7.420
7.403
7.399
7.397
7.393
7.388
7.383
7.310
7.273
6.121
6.102
5.984
5.965
4.620
3.959
0.000
1.01
3.01
3.02
1.06
5.04
1.00
1.03
2.00
3.02
f1 (ppm)

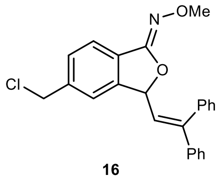

16

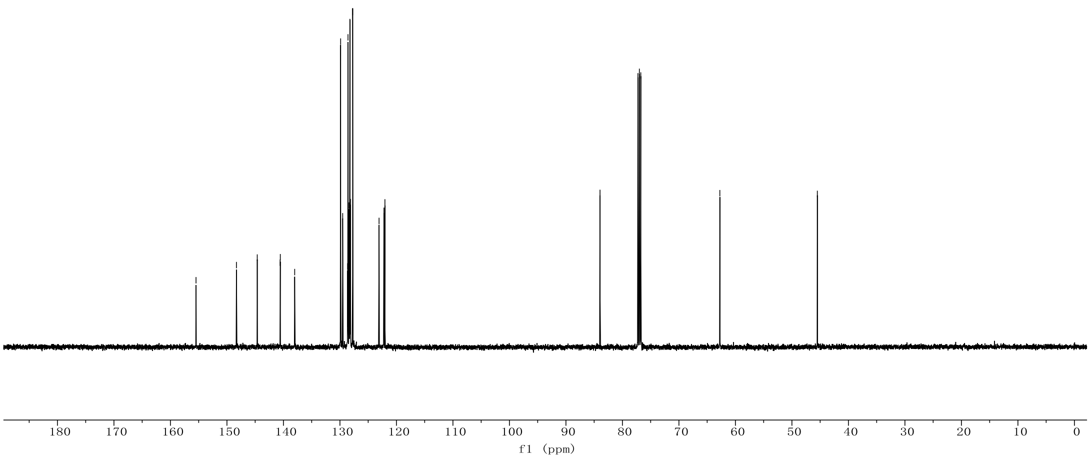
155.472
148.316
144.633
140.584
140.552
138.009
129.874
129.511
128.643
128.571
128.379
128.237
128.135
127.741
123.076
122.209
122.039
83.965
77.257
77.003
76.749
62.758
45.501
f1 (ppm)

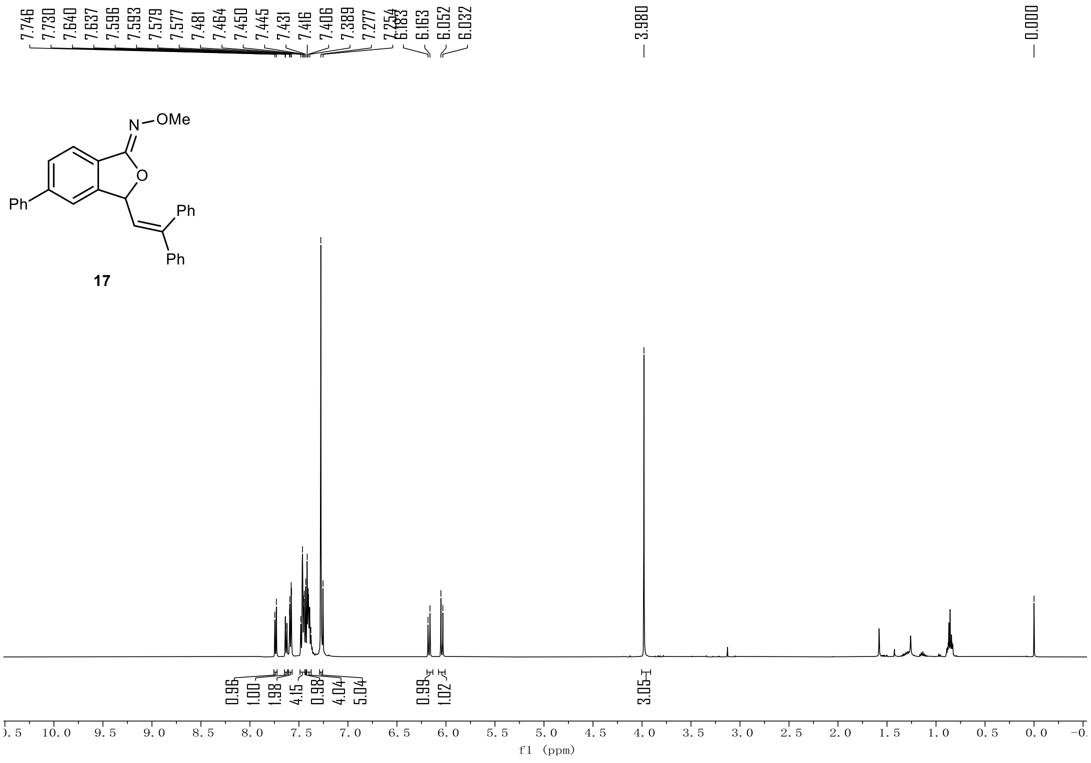

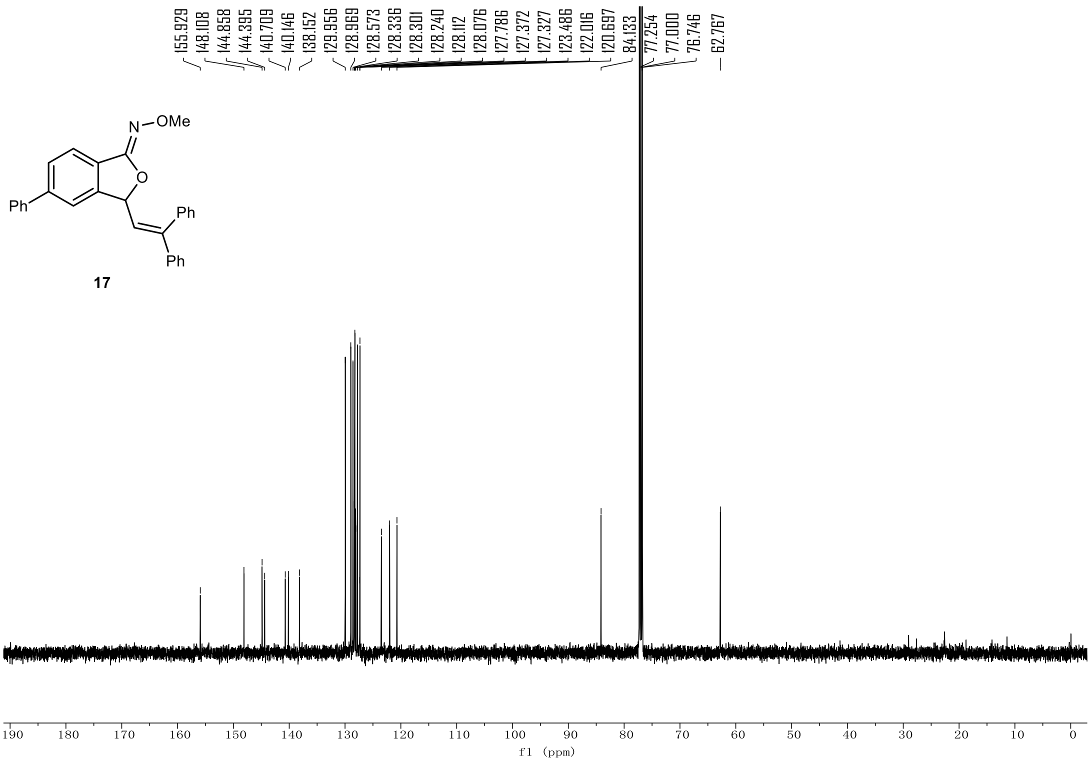

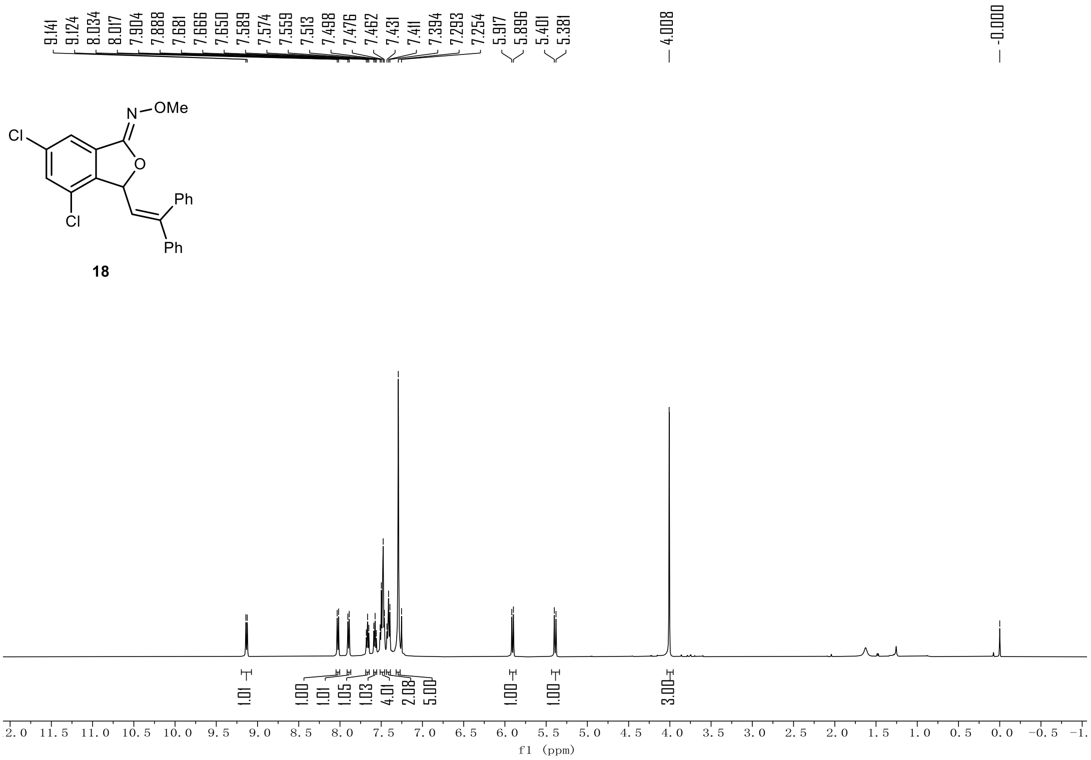
9.141
9.124
8.034
8.017
7.904
7.888
7.681
7.666
7.650
7.589
7.574
7.559
7.513
7.498
7.476
7.462
7.431
7.411
7.394
7.293
7.254
5.917
5.896
5.401
5.381
4.008
-0.000
N
OMe
Cl
O
Cl
Ph
Ph
18
1.01
1.00
1.01
1.05
1.03
4.01
2.08
5.00
1.00
1.00
3.00
f1 (ppm)

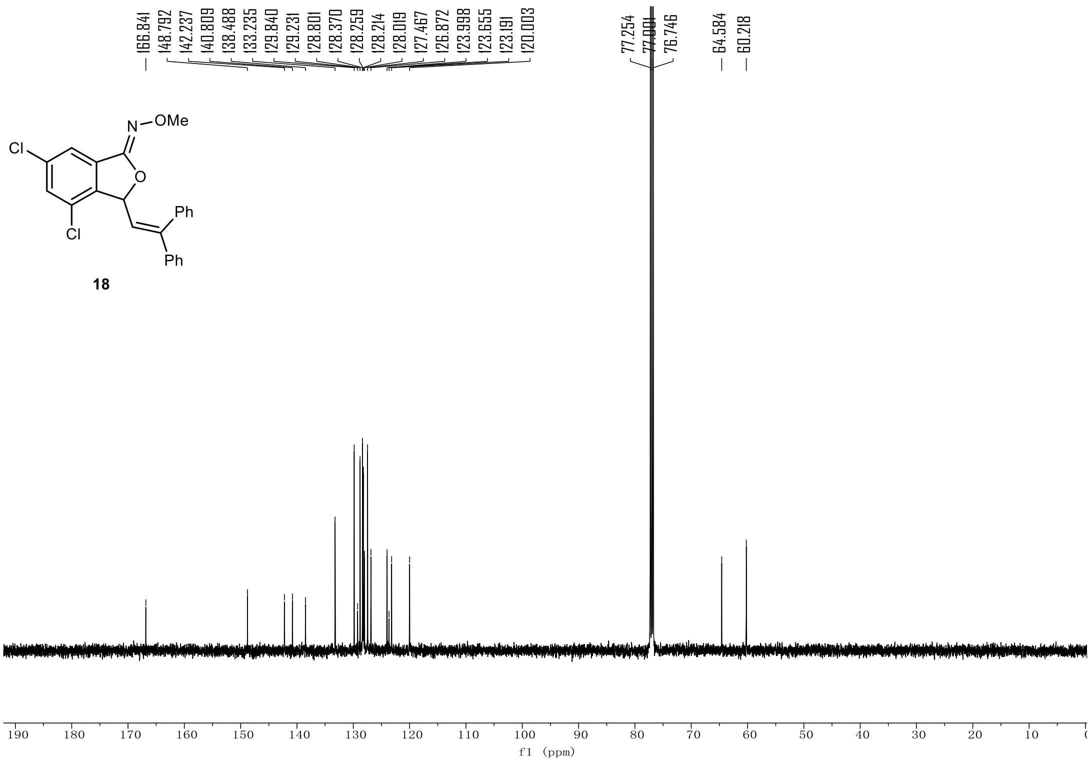
166.841
148.792
142.237
140.809
138.488
133.235
129.840
129.231
128.801
128.370
128.259
128.214
128.019
127.467
126.872
123.998
123.655
123.191
120.003
77.254
77.001
76.746
64.584
60.218
N
OMe
Cl
O
Cl
Ph
Ph
18
f1 (ppm)

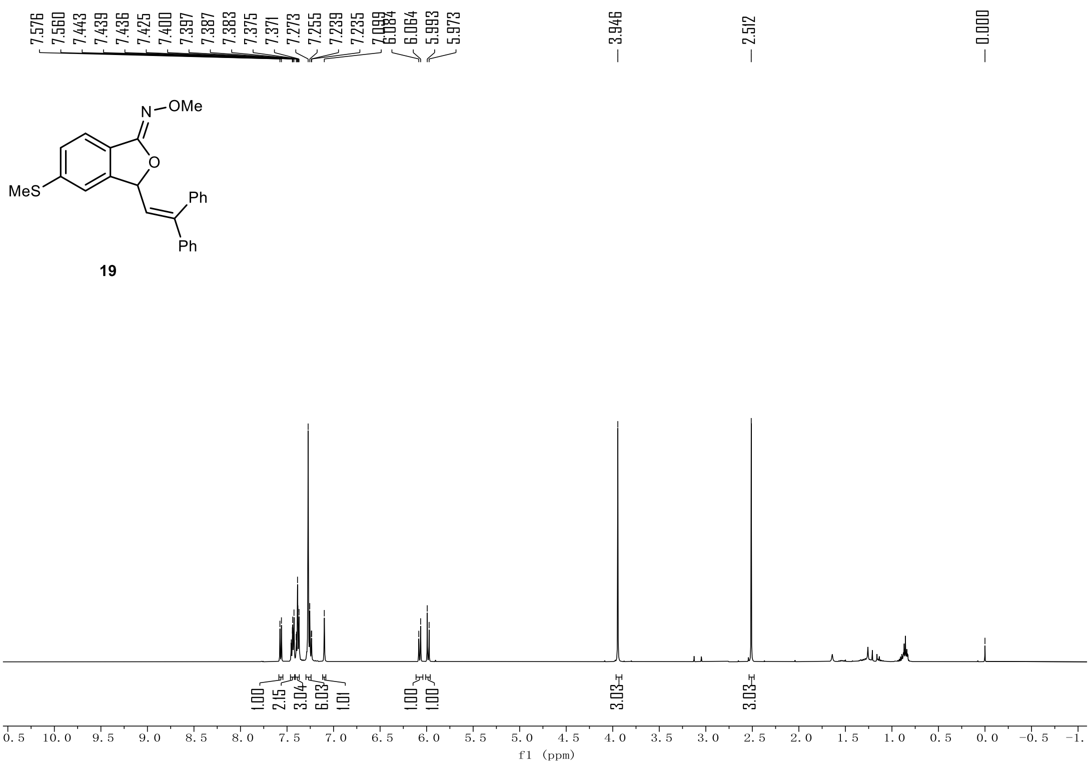
N–OMe
O
MeS
Ph
Ph
19
f1 (ppm)

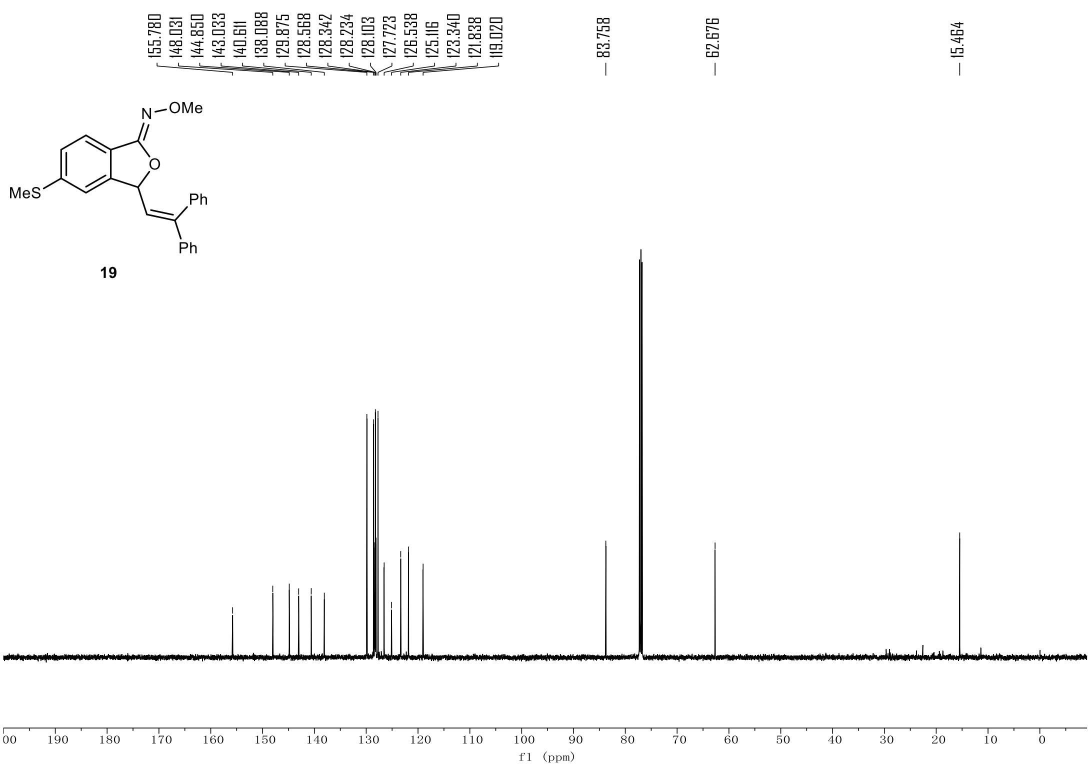
N–OMe
O
MeS
Ph
Ph
19
f1 (ppm)

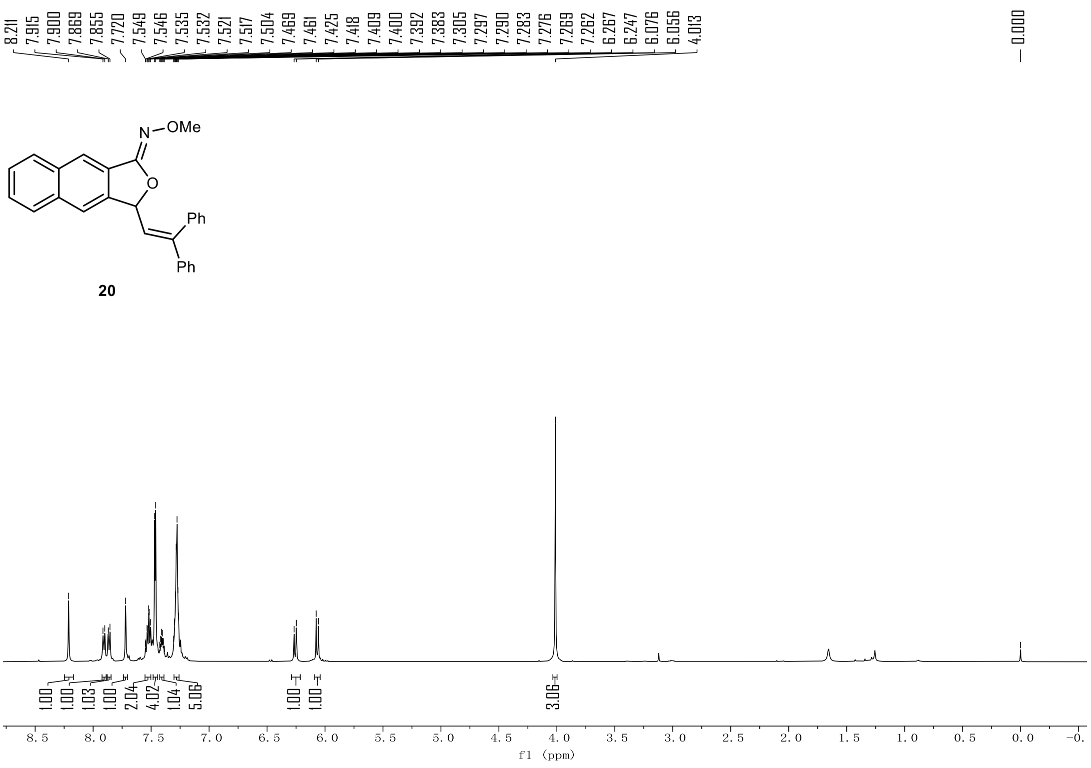
8.211 7.915 7.900 7.869 7.855 7.720 7.549 7.546 7.535 7.532 7.521 7.517 7.504 7.469 7.461 7.425 7.418 7.409 7.400 7.392 7.383 7.305 7.297 7.290 7.283 7.276 7.269 7.262 6.267 6.247 6.076 6.056 4.013
0.000
N
OMe
O
Ph
Ph
20
1.00 1.00 1.03 1.00 2.04 4.02 1.04 5.06
1.00 1.00
3.06
f1 (ppm)

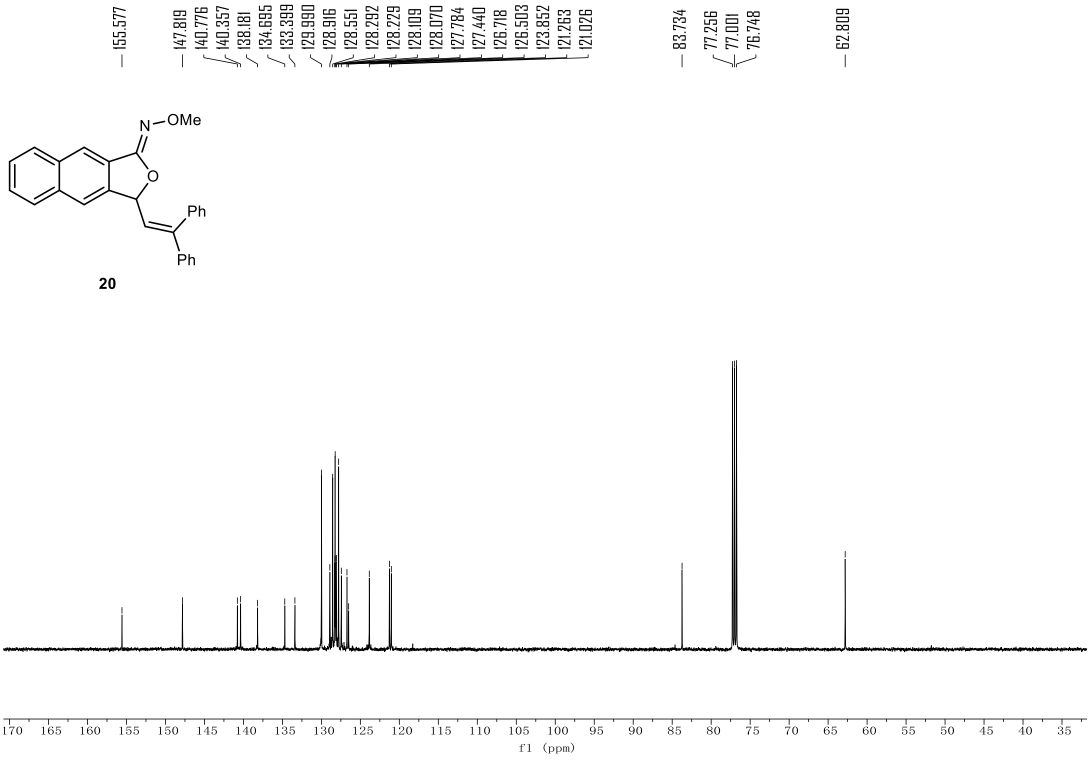
155.577
147.819 140.776 140.357 138.181 134.695 133.399 129.990 128.916 128.551 128.292 128.229 128.109 128.070 127.784 127.440 126.718 126.503 123.852 121.263 121.026
83.734
77.256 77.001 76.748
62.809
N
OMe
O
Ph
Ph
20
f1 (ppm)

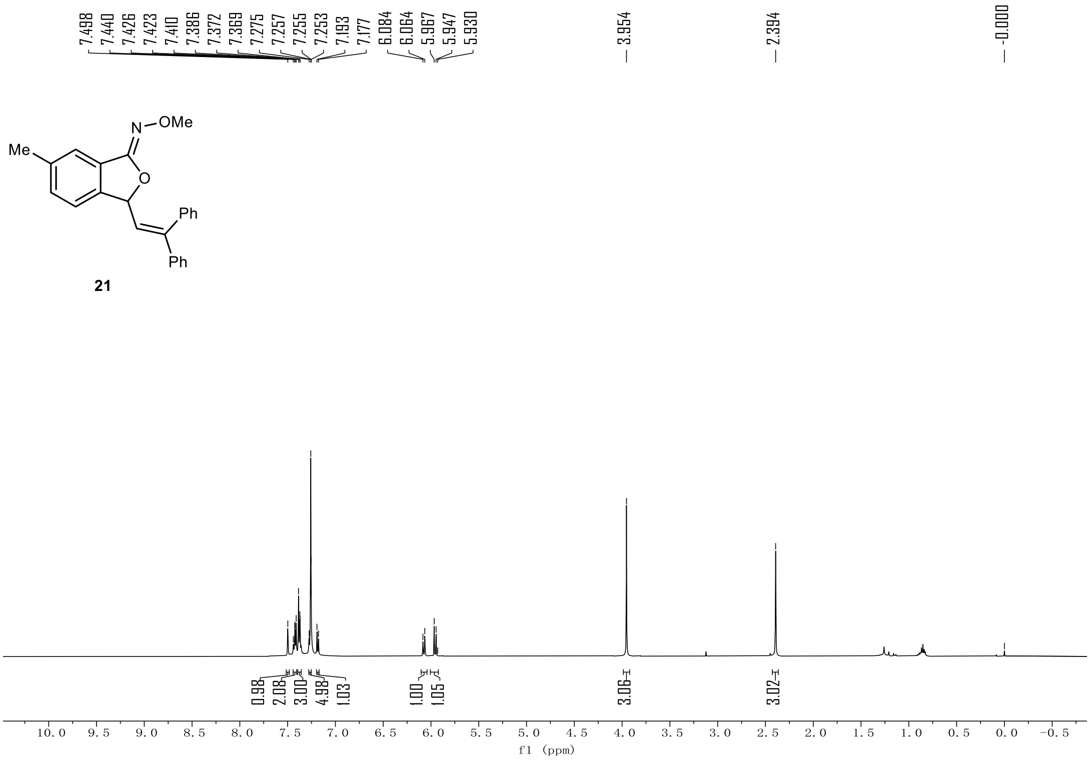
7.498
7.440
7.426
7.423
7.410
7.386
7.372
7.369
7.275
7.257
7.255
7.253
7.193
7.177
6.084
6.064
5.967
5.947
5.930
3.954
2.394
-0.000
Me
N
OMe
O
Ph
Ph
21
0.98
2.08
3.00
4.98
1.03
1.00
1.05
3.06
3.02
f1 (ppm)

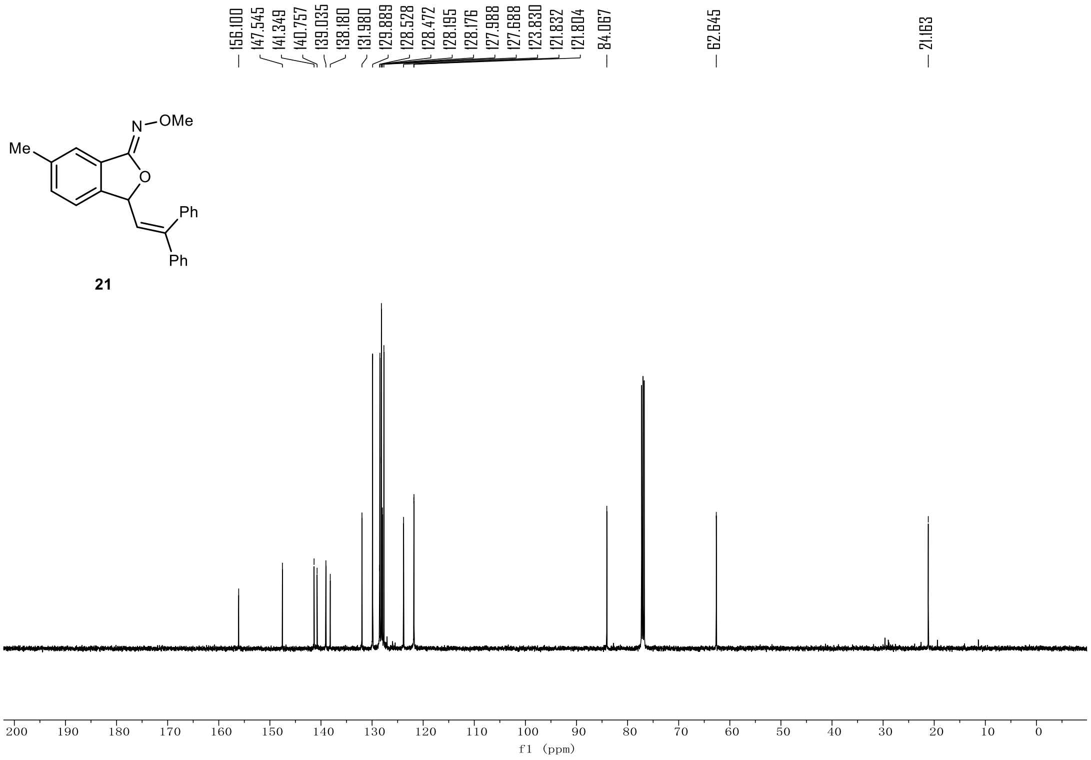
156.100
147.545
141.349
140.757
139.035
138.180
131.980
129.889
128.528
128.472
128.195
128.176
127.988
127.688
123.830
121.832
121.804
84.067
62.645
21.163
Me
N
OMe
O
Ph
Ph
21
f1 (ppm)

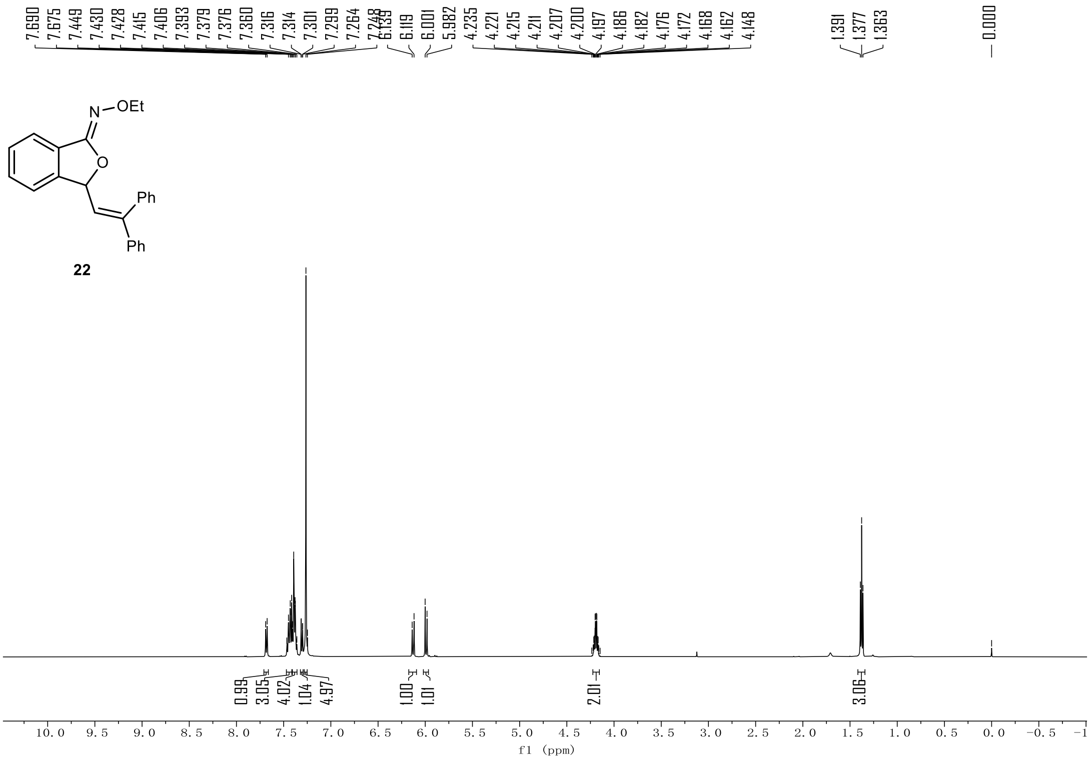
7.690 7.675 7.449 7.430 7.428 7.415 7.406 7.393 7.379 7.376 7.360 7.316 7.314 7.301 7.299 7.264 7.248 6.139 6.119 6.001 5.982 4.235 4.221 4.215 4.211 4.207 4.200 4.197 4.186 4.182 4.176 4.172 4.168 4.162 4.148 1.391 1.377 1.363 0.000
N–OEt
O
Ph
Ph
22
0.99 3.05 4.02 1.04 4.97 1.00 1.01 2.01 3.06
10.0 9.5 9.0 8.5 8.0 7.5 7.0 6.5 6.0 5.5 5.0 4.5 4.0 3.5 3.0 2.5 2.0 1.5 1.0 0.5 0.0 -0.5 -1
f1 (ppm)

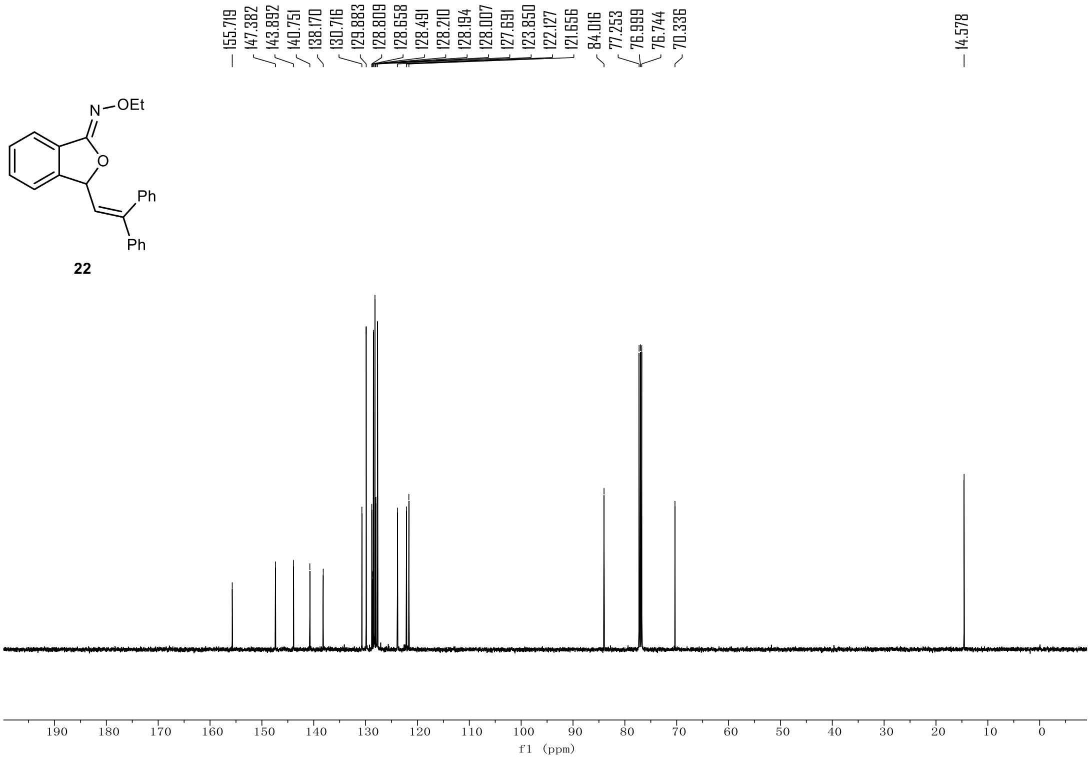
155.719 147.382 143.892 140.751 138.170 130.716 129.883 128.809 128.658 128.491 128.210 128.194 128.007 127.691 123.850 122.127 121.656 84.016 77.253 76.999 76.744 70.336 14.578
N–OEt
O
Ph
Ph
22
190 180 170 160 150 140 130 120 110 100 90 80 70 60 50 40 30 20 10 0
f1 (ppm)

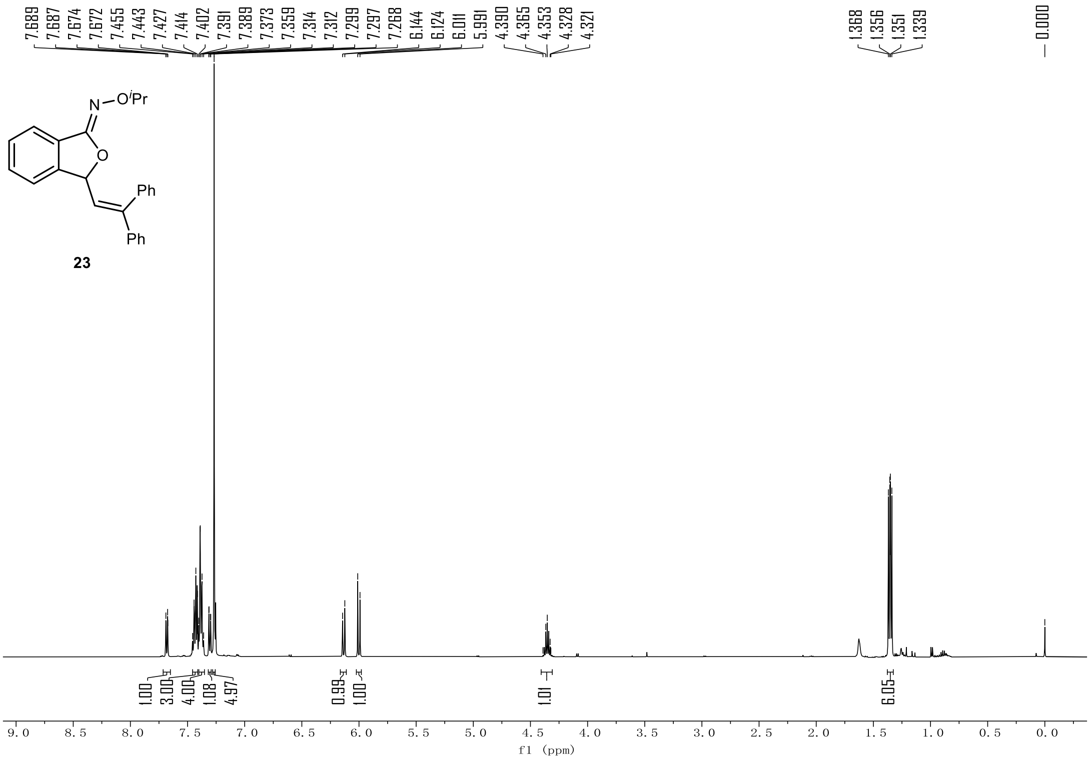

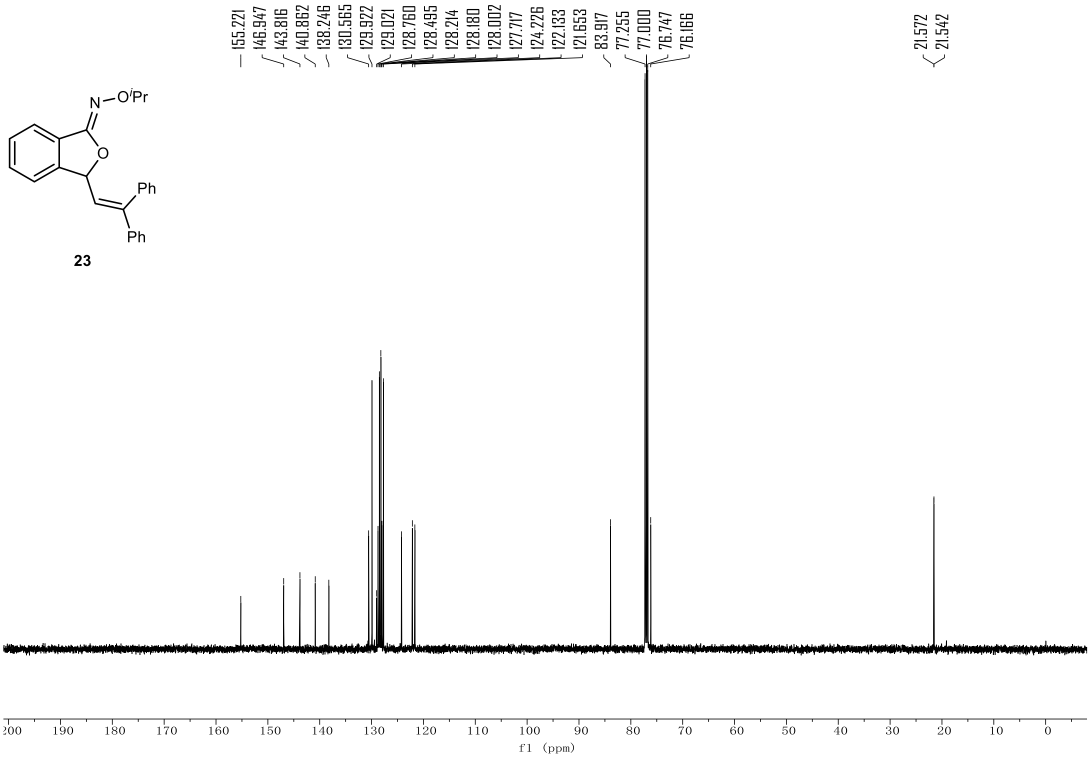

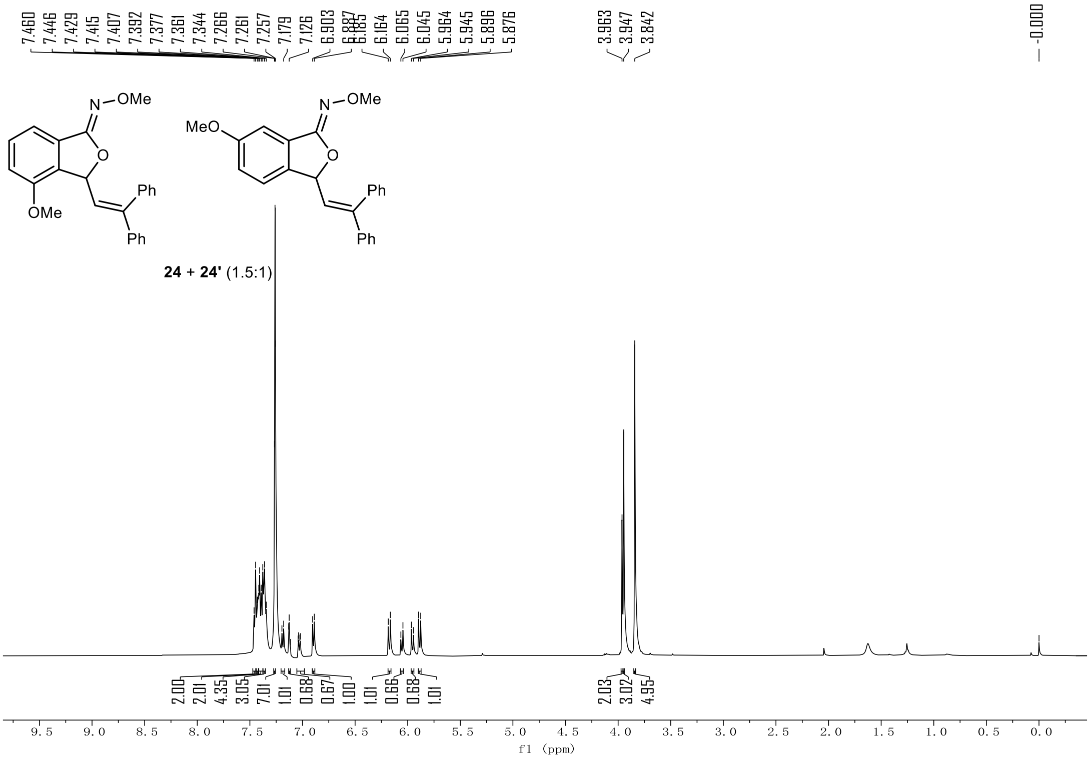
7.460
7.446
7.429
7.415
7.407
7.392
7.377
7.361
7.344
7.266
7.261
7.257
7.179
7.126
6.903
6.887
6.185
6.164
6.065
6.045
5.964
5.945
5.896
5.876
3.963
3.947
3.842
-0.000
N–OMe
O
Ph
OMe
Ph
MeO
24 + 24' (1.5:1)
2.00
2.01
4.35
3.05
7.01
1.01
0.68
0.67
1.00
1.01
0.66
0.68
1.01
2.03
3.02
4.95
f1 (ppm)

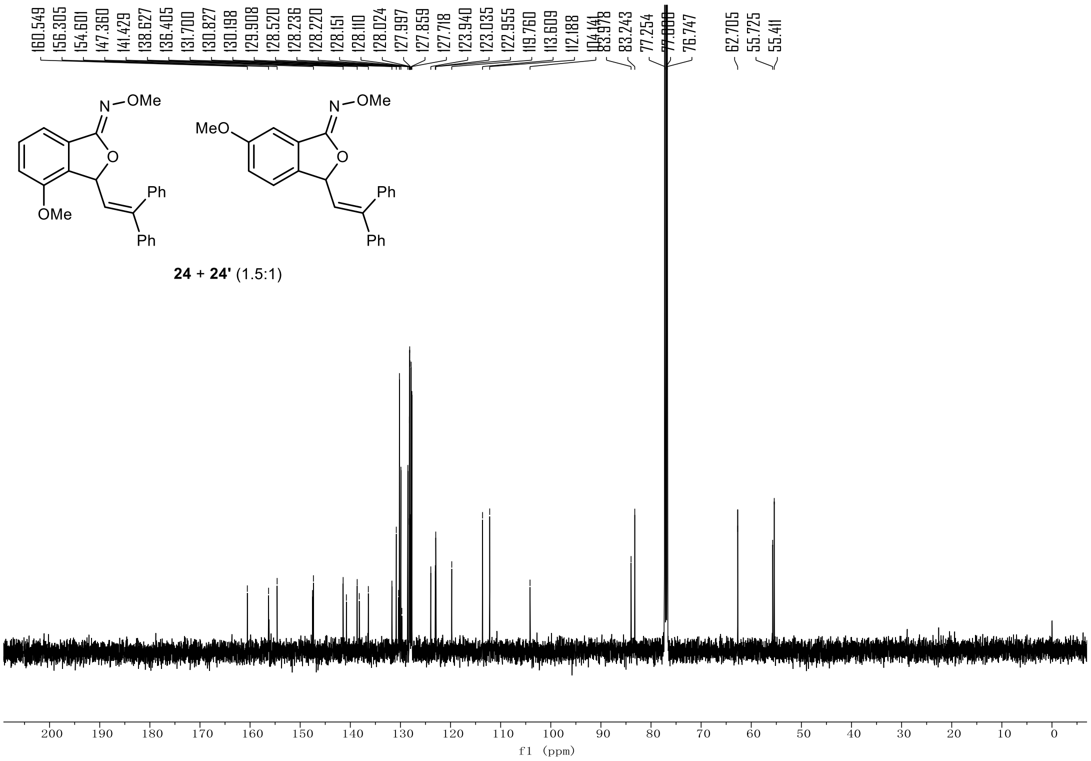
160.549
156.305
154.601
147.360
141.429
138.627
136.405
131.700
130.827
130.198
129.908
128.520
128.236
128.220
128.151
128.110
128.024
127.997
127.859
127.718
123.940
123.035
122.955
119.760
113.609
112.188
104.141
83.978
83.243
77.254
76.747
62.705
55.725
55.411
N–OMe
O
Ph
OMe
Ph
MeO
24 + 24' (1.5:1)
f1 (ppm)

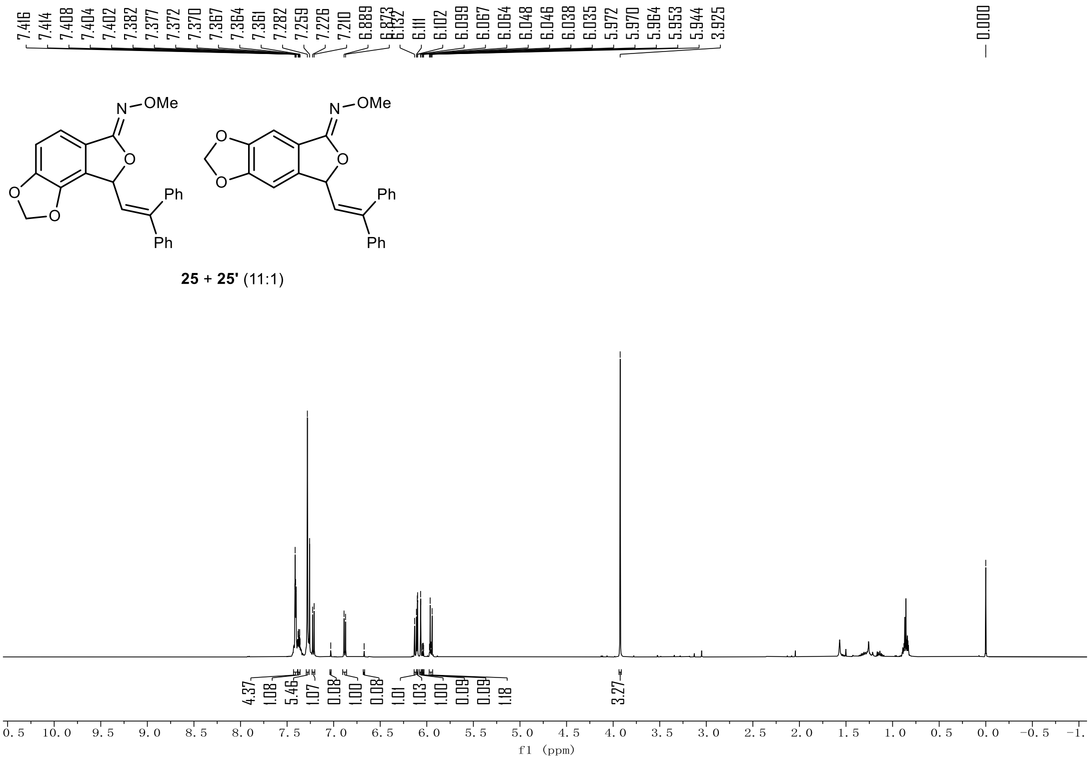

25 + 25' (11:1)

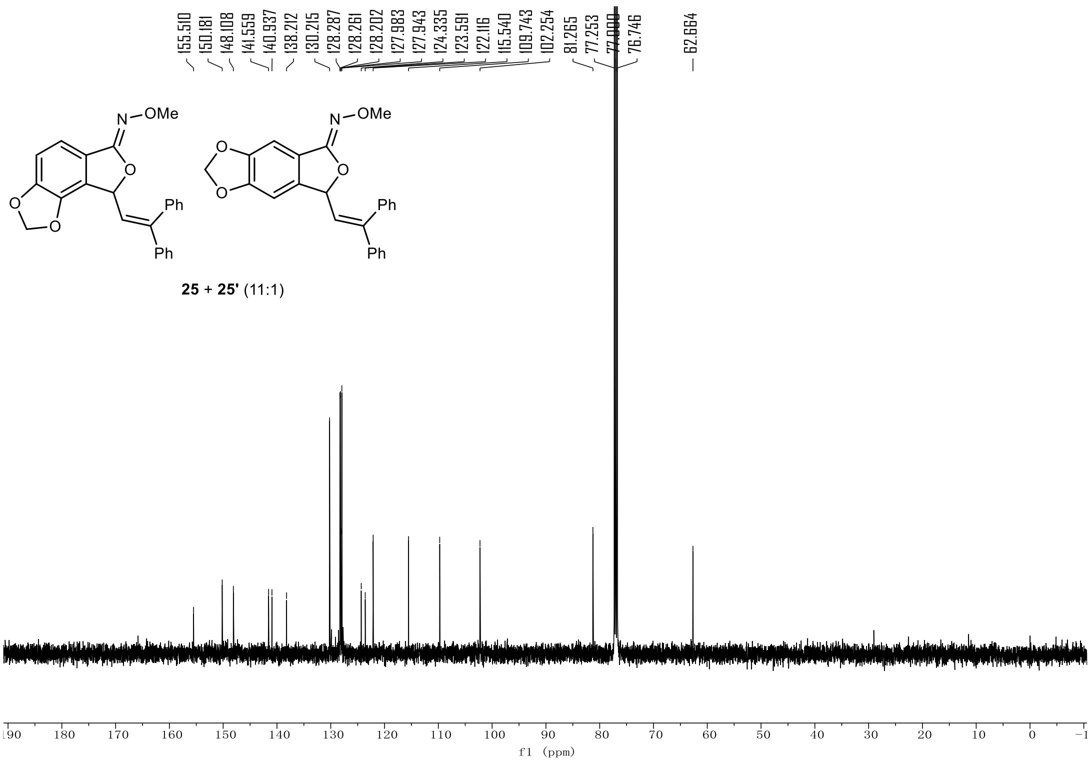

25 + 25' (11:1)

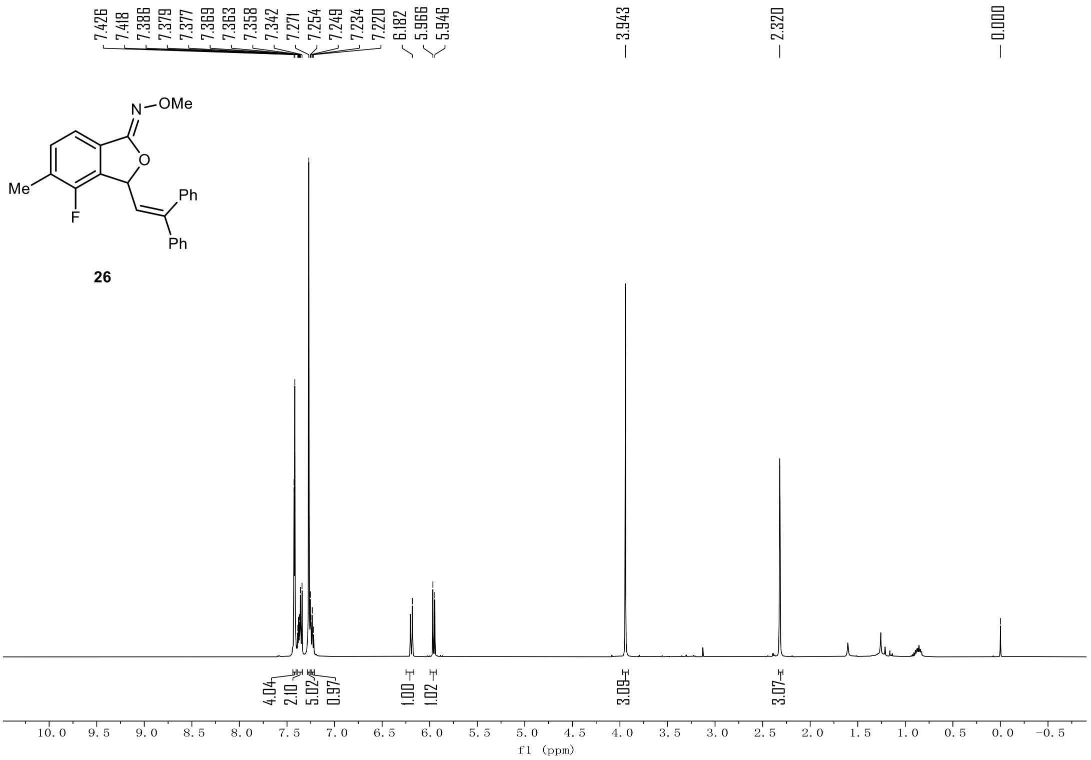
7.426
7.418
7.386
7.379
7.377
7.369
7.363
7.358
7.342
7.271
7.254
7.249
7.234
7.220
6.182
5.966
5.946
3.943
2.320
0.000
N–OMe
O
Me
F
Ph
Ph
26
4.04
2.10
5.02
0.97
1.00
1.02
3.09
3.07
10.0 9.5 9.0 8.5 8.0 7.5 7.0 6.5 6.0 5.5 5.0 4.5 4.0 3.5 3.0 2.5 2.0 1.5 1.0 0.5 0.0 -0.5
f1 (ppm)

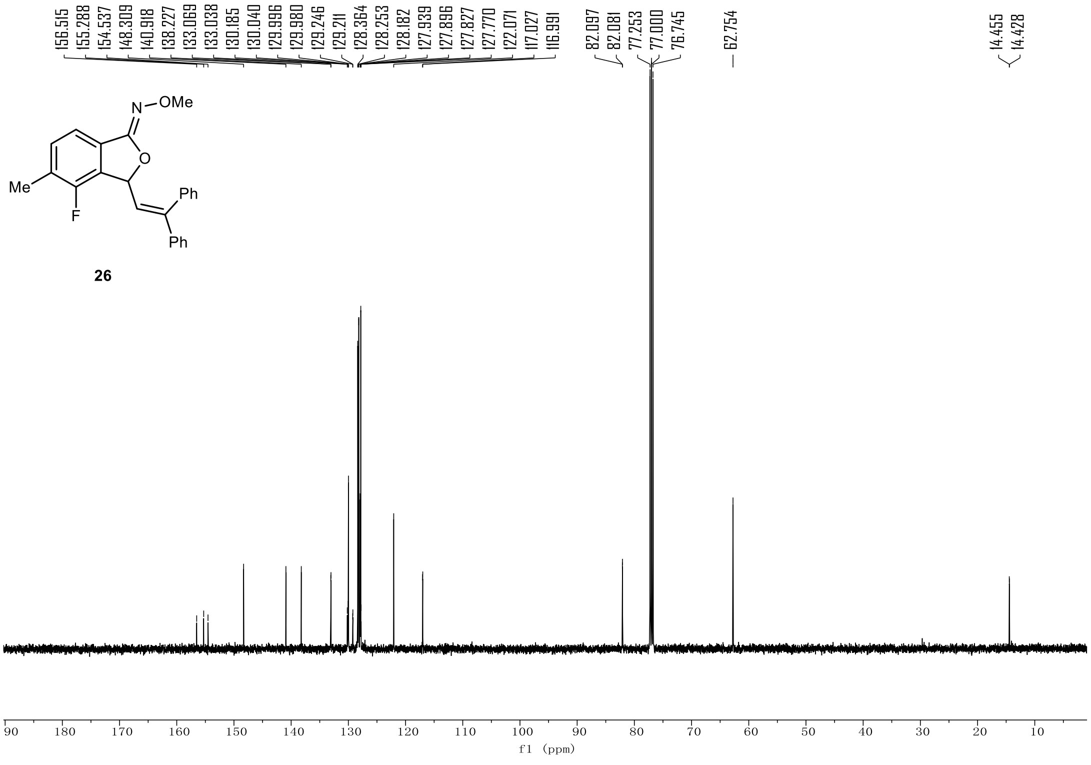
156.515
155.288
154.537
148.309
140.918
138.227
133.069
133.038
130.185
130.040
129.996
129.980
129.246
129.211
128.364
128.253
128.182
127.939
127.896
127.827
127.770
122.071
117.027
116.991
82.097
82.081
77.253
77.000
76.745
62.754
14.455
14.428
N–OMe
O
Me
F
Ph
Ph
26
90 180 170 160 150 140 130 120 110 100 90 80 70 60 50 40 30 20 10
f1 (ppm)

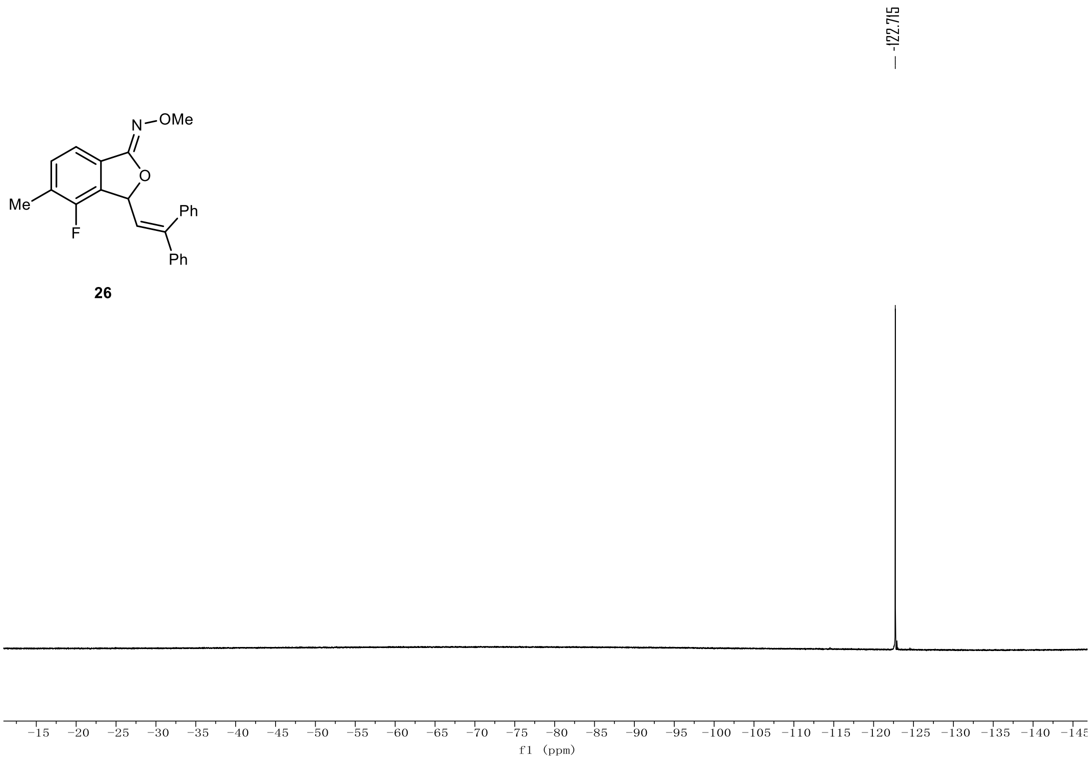
-122.715
N—OMe
O
Me
F
Ph
Ph
26
f1 (ppm)

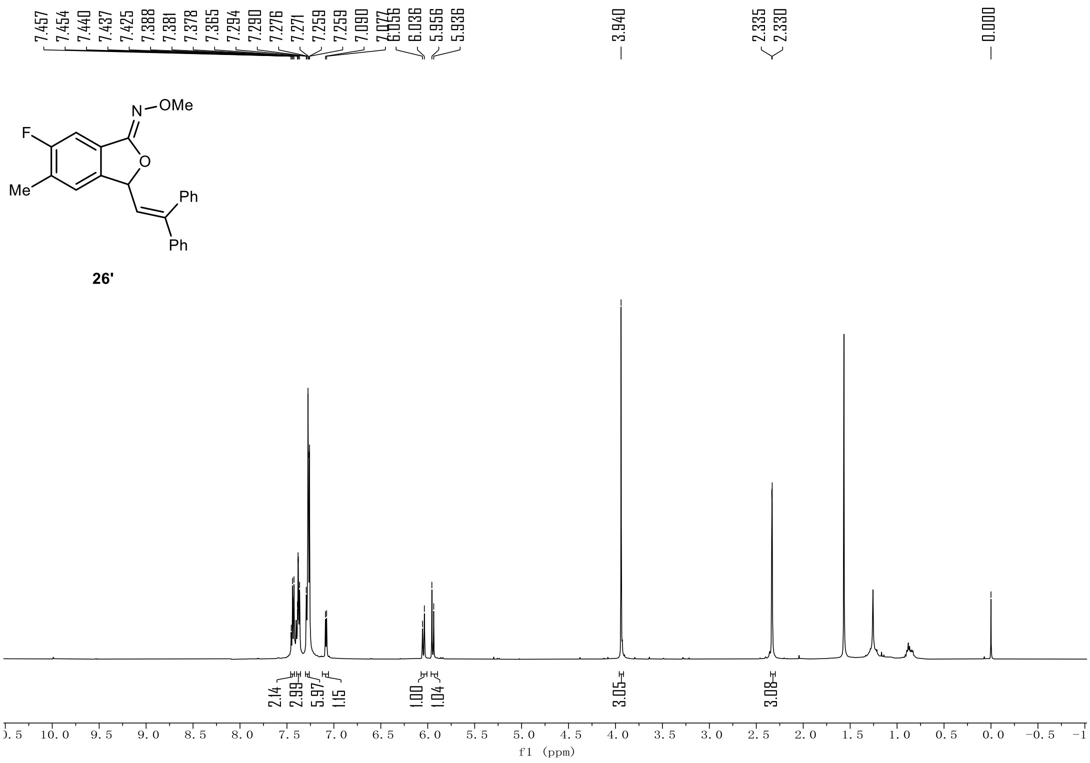
7.457
7.454
7.440
7.437
7.425
7.388
7.381
7.378
7.365
7.294
7.290
7.276
7.271
7.259
7.259
7.090
7.077
6.056
6.036
5.956
5.936
3.940
2.335
2.330
0.000
N—OMe
F
O
Me
Ph
Ph
26'
2.14
2.99
5.97
1.15
1.00
1.04
3.05
3.08
f1 (ppm)

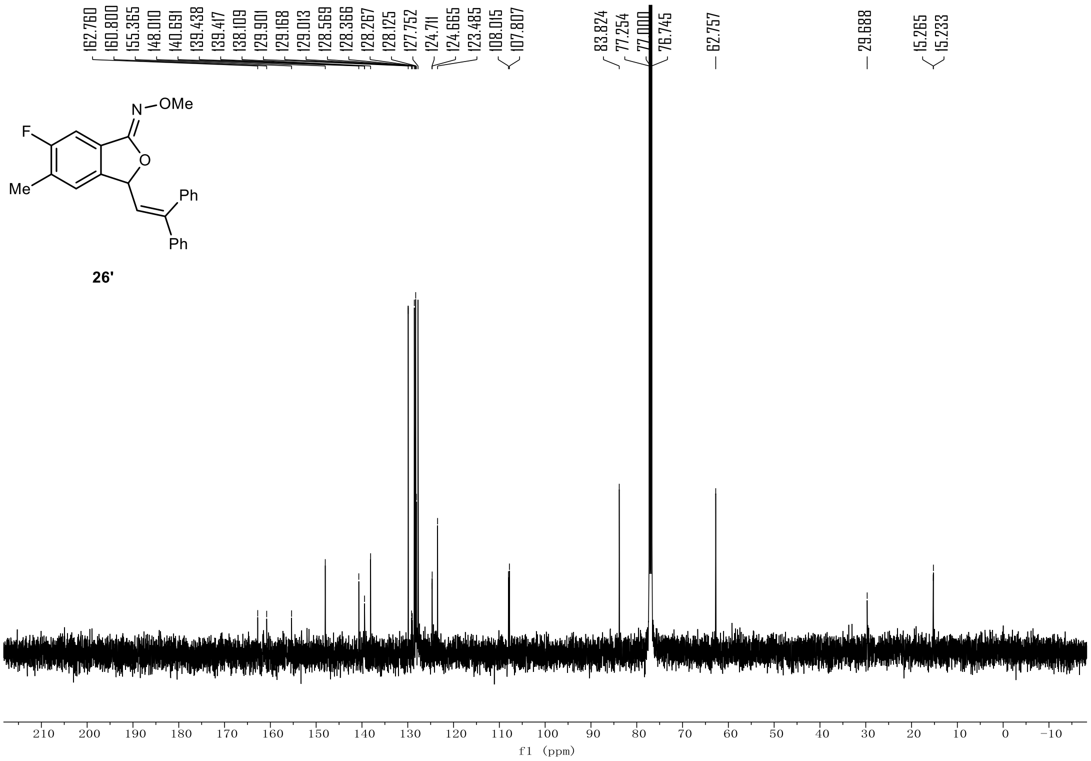
N–OMe
F
O
Me
Ph
Ph
26'
162.760
160.800
155.365
148.010
140.691
139.438
139.417
138.109
129.901
129.168
129.013
128.569
128.366
128.267
128.125
127.752
124.711
124.665
123.485
108.015
107.807
83.824
77.254
77.000
76.745
62.757
29.688
15.265
15.233
f1 (ppm)

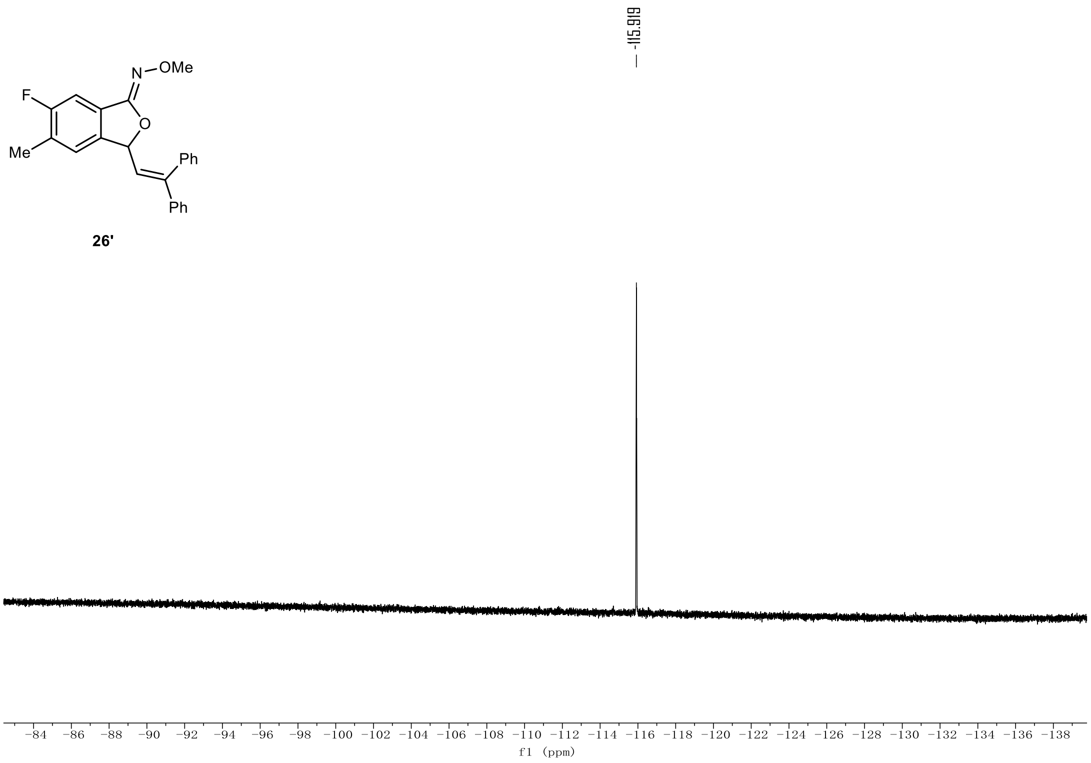
N–OMe
F
O
Me
Ph
Ph
26'
-115.919
f1 (ppm)

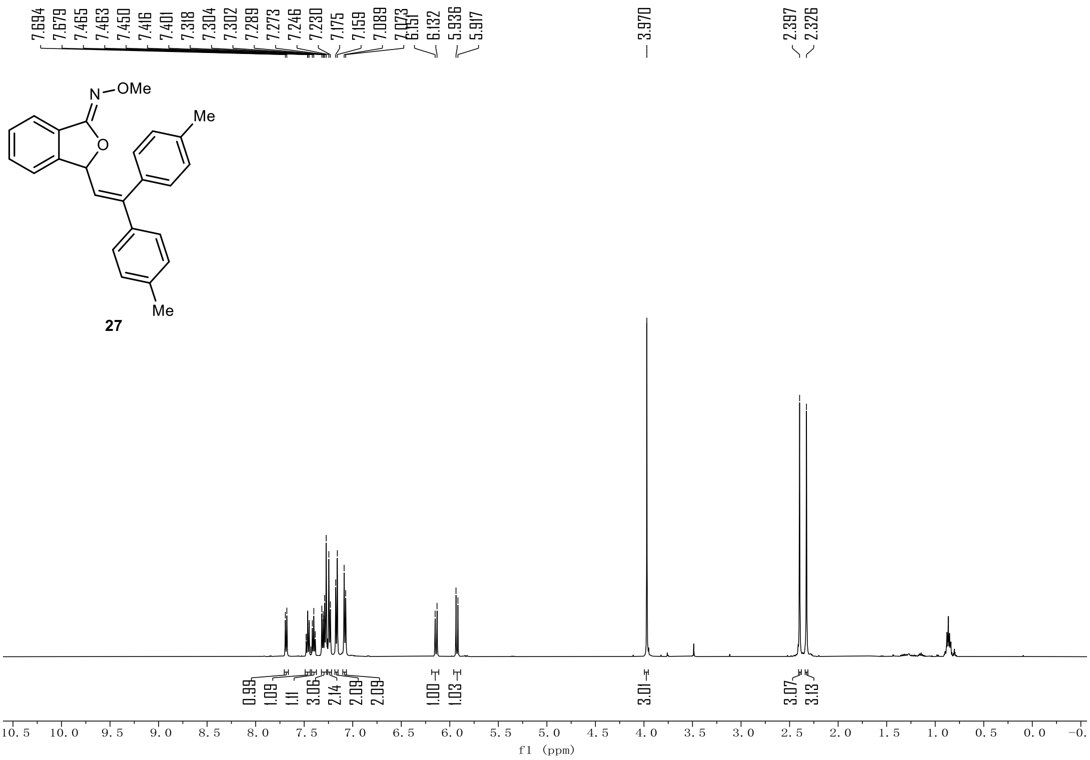

7.694
7.679
7.465
7.463
7.450
7.416
7.401
7.318
7.304
7.302
7.289
7.273
7.246
7.230
7.175
7.159
7.089
7.073
6.151
6.132
5.936
5.917
3.970
2.397
2.326
N
OMe
O
Me
Me
27
0.99
1.09
1.11
3.06
2.14
2.09
2.09
1.00
1.03
3.01
3.07
3.13
10.5 10.0 9.5 9.0 8.5 8.0 7.5 7.0 6.5 6.0 5.5 5.0 4.5 4.0 3.5 3.0 2.5 2.0 1.5 1.0 0.5 0.0 -0.
f1 (ppm)

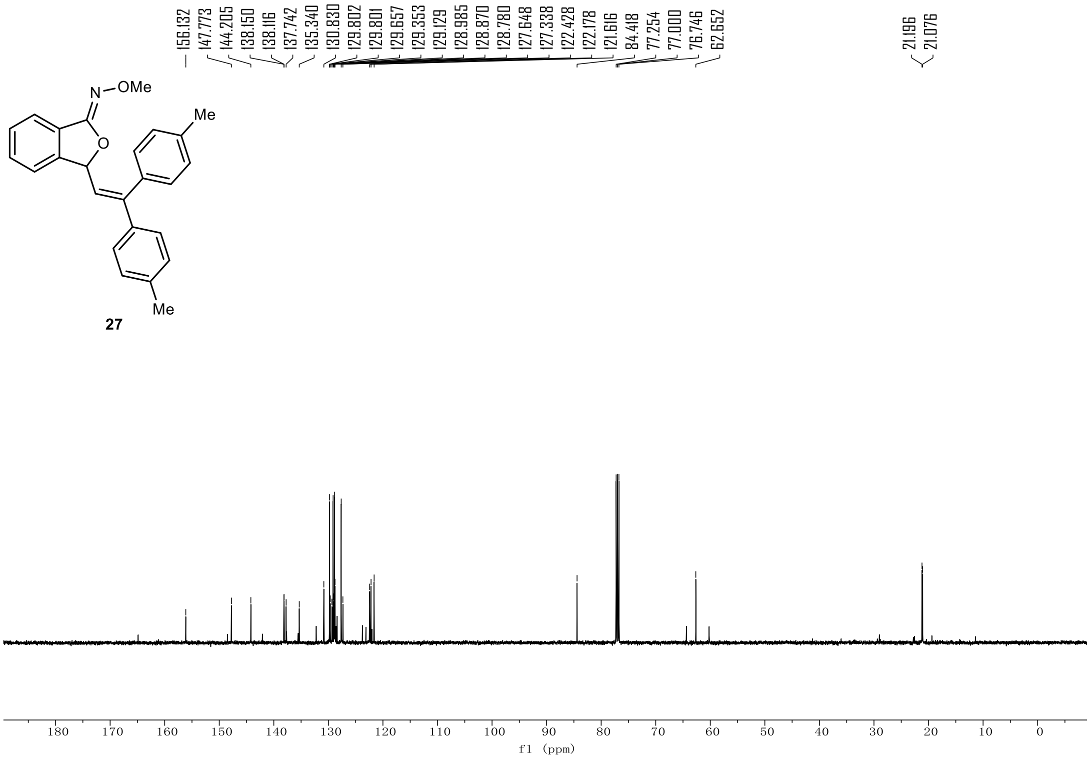

156.132
147.773
144.205
138.150
138.116
137.742
135.340
130.830
129.802
129.801
129.657
129.353
129.129
128.985
128.870
128.780
127.648
127.338
122.428
122.178
121.616
84.418
77.254
77.000
76.746
62.652
21.196
21.076
N
OMe
O
Me
Me
27
180 170 160 150 140 130 120 110 100 90 80 70 60 50 40 30 20 10 0
f1 (ppm)

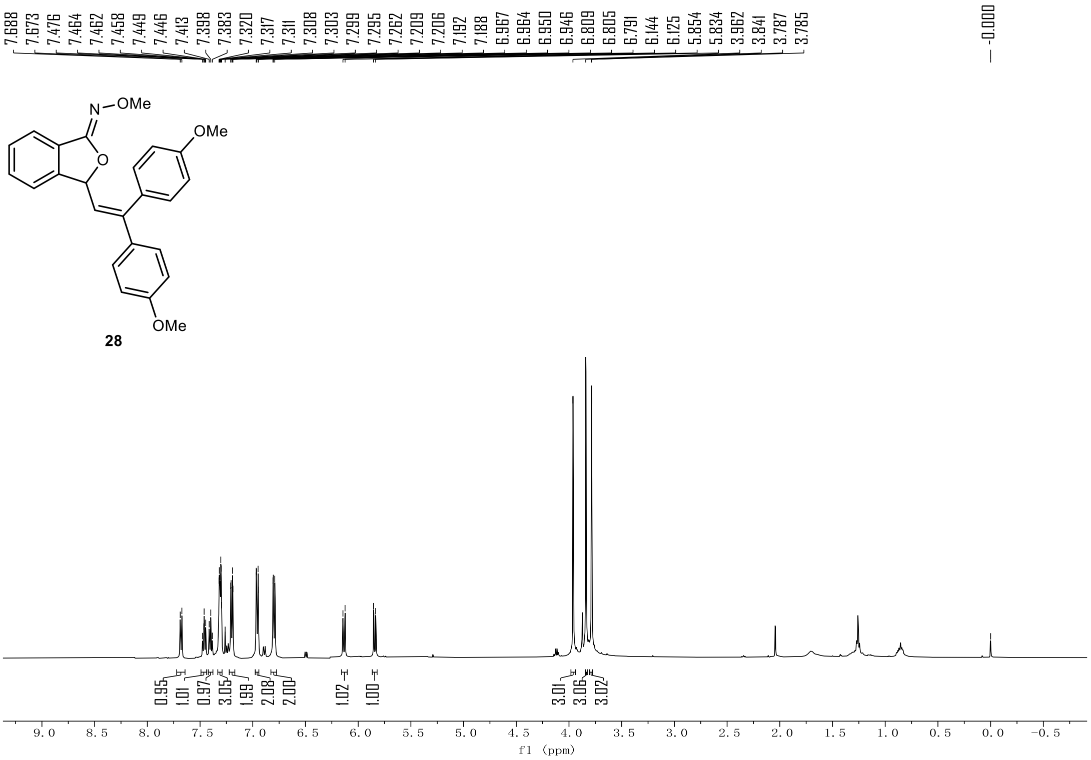
7.688
7.673
7.476
7.464
7.462
7.458
7.449
7.446
7.413
7.398
7.383
7.320
7.317
7.311
7.308
7.303
7.299
7.295
7.262
7.209
7.206
7.192
7.188
6.967
6.964
6.950
6.946
6.809
6.805
6.791
6.144
6.125
5.854
5.834
3.962
3.841
3.787
3.785
-0.000
N
OMe
O
OMe
OMe
28
0.95
1.01
0.97
3.05
1.99
2.08
2.00
1.02
1.00
3.01
3.06
3.02
f1 (ppm)

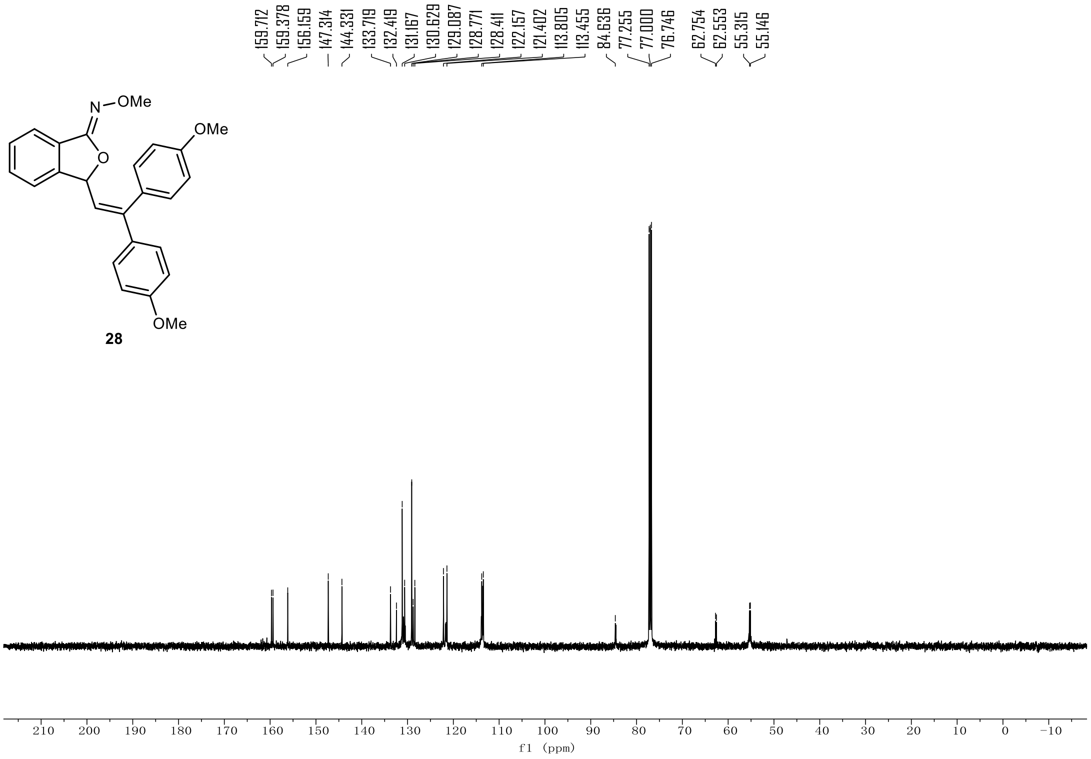
159.712
159.378
156.159
147.314
144.331
133.719
132.419
131.167
130.629
129.087
128.771
128.411
122.157
121.402
113.805
113.455
84.636
77.255
77.000
76.746
62.754
62.553
55.315
55.146
N
OMe
O
OMe
OMe
28
f1 (ppm)

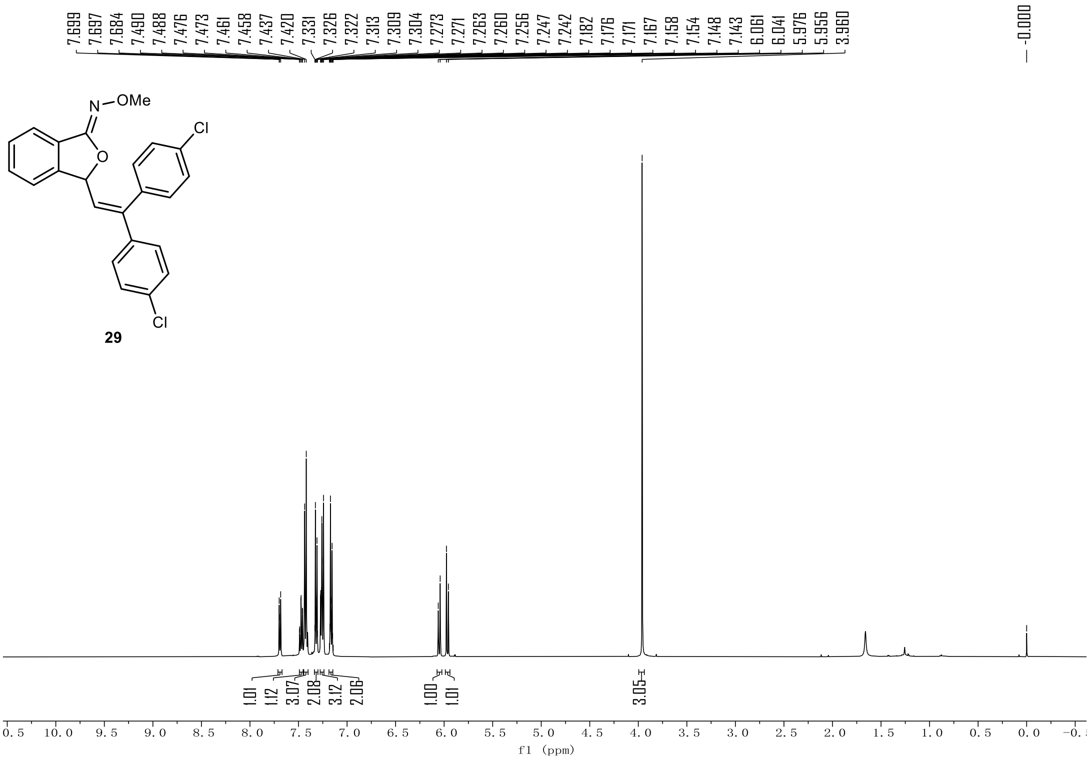
N–OMe
O
Cl
Cl
29
7.699
7.697
7.684
7.490
7.488
7.476
7.473
7.461
7.458
7.437
7.420
7.331
7.326
7.322
7.313
7.309
7.304
7.273
7.271
7.263
7.260
7.256
7.247
7.242
7.182
7.176
7.171
7.167
7.158
7.154
7.148
7.143
6.061
6.041
5.976
5.956
3.960
-0.000
1.01
1.12
3.07
2.08
3.12
2.06
1.00
1.01
3.05
f1 (ppm)

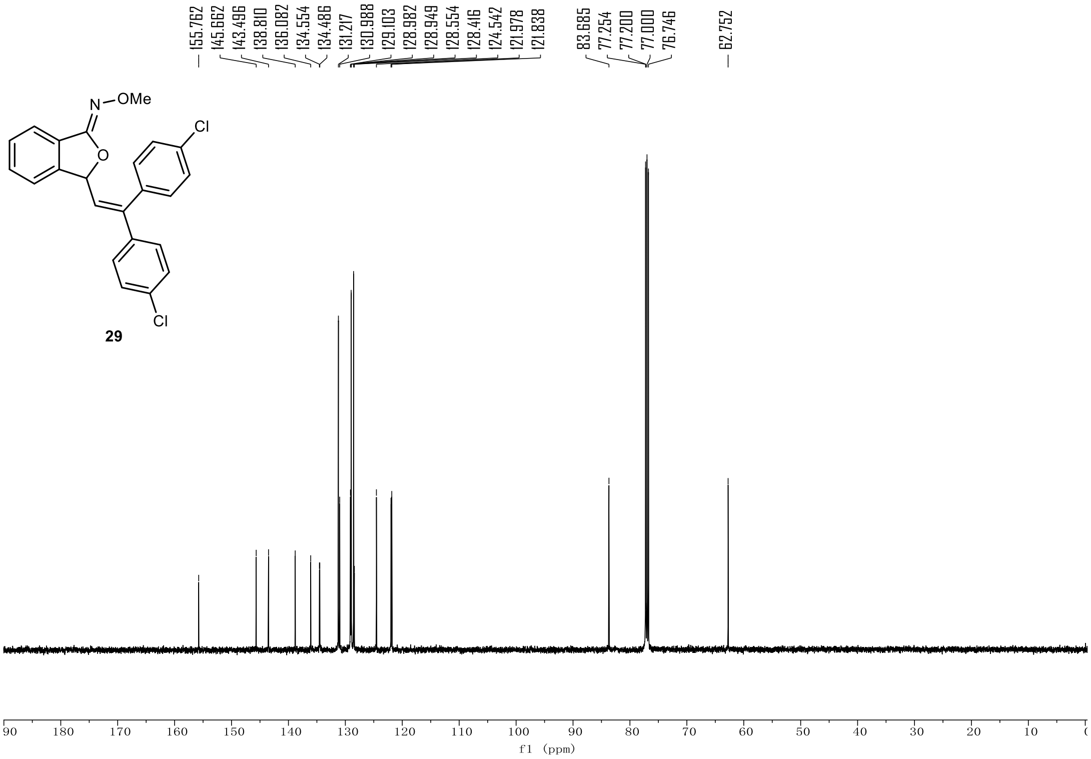
N–OMe
O
Cl
Cl
29
155.762
145.662
143.496
138.810
136.082
134.554
134.486
131.217
130.988
129.103
128.982
128.949
128.554
128.416
124.542
121.978
121.838
83.685
77.254
77.200
77.000
76.746
62.752
f1 (ppm)

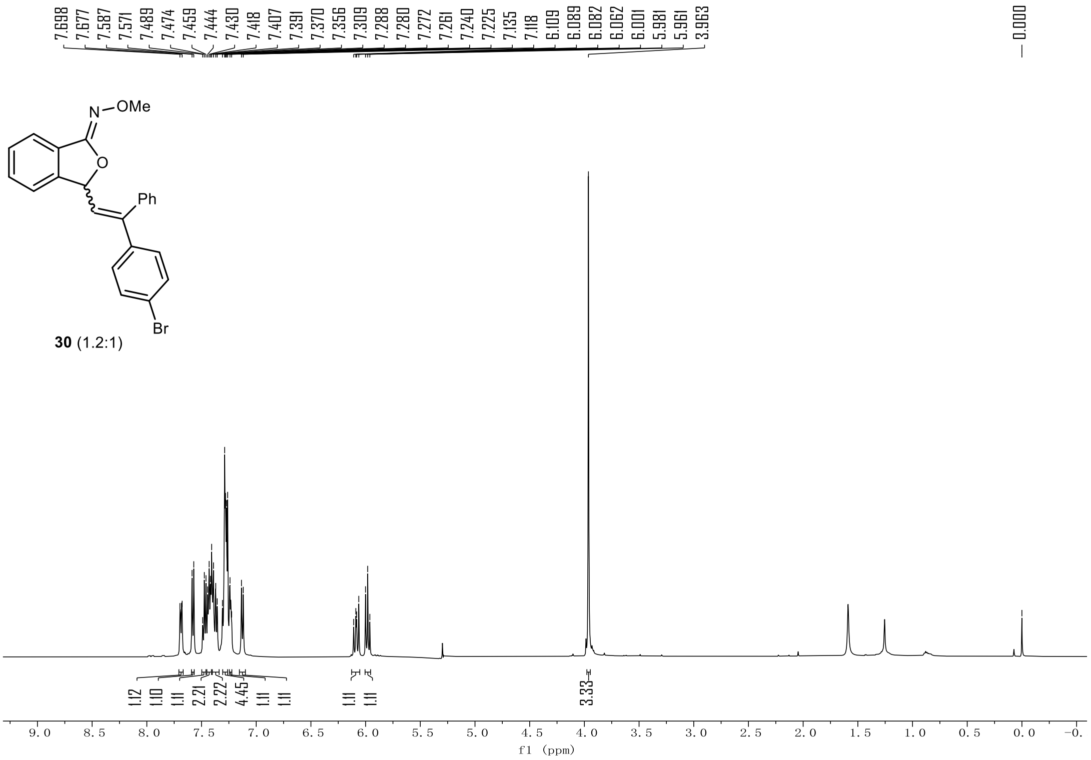
7.698
7.677
7.587
7.571
7.489
7.474
7.459
7.444
7.430
7.418
7.407
7.391
7.370
7.356
7.309
7.288
7.280
7.272
7.261
7.240
7.225
7.135
7.118
6.109
6.089
6.082
6.062
6.001
5.981
5.961
3.963
0.000
N–OMe
O
Ph
Br
30 (1.2:1)
1.12
1.10
1.11
2.21
2.22
4.45
1.11
1.11
1.11
1.11
3.33
9.0 8.5 8.0 7.5 7.0 6.5 6.0 5.5 5.0 4.5 4.0 3.5 3.0 2.5 2.0 1.5 1.0 0.5 0.0 -0.
f1 (ppm)

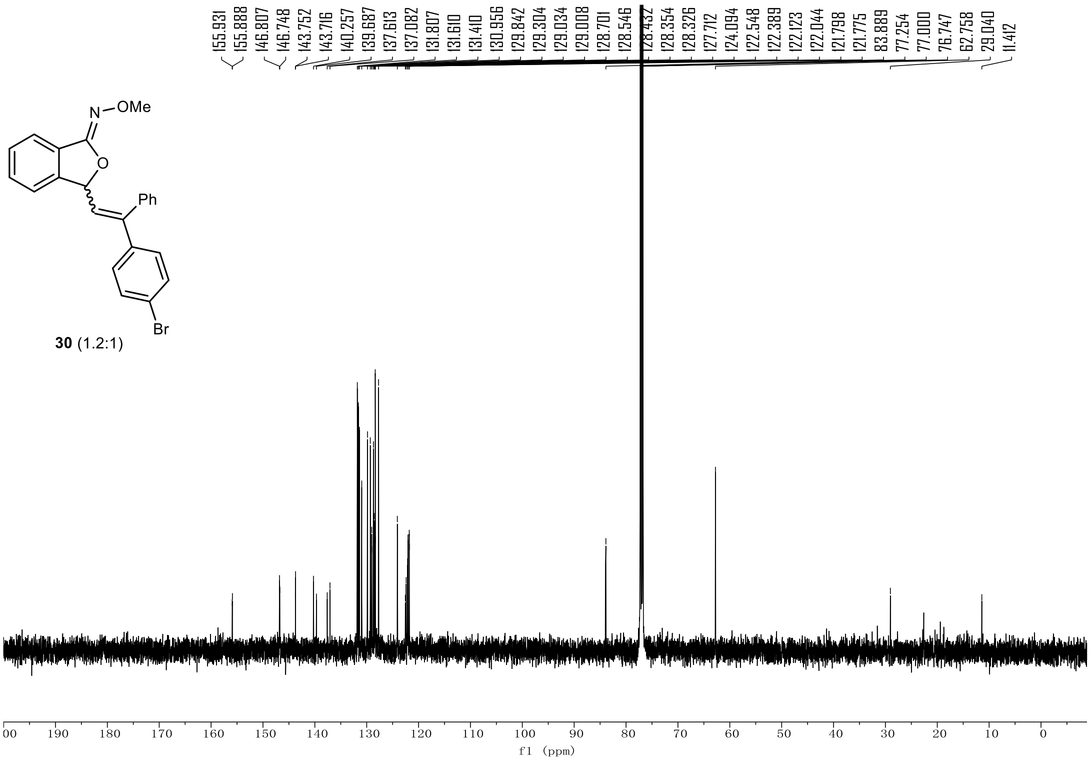
155.931
155.888
146.807
146.748
143.752
143.716
140.257
139.687
137.613
137.082
131.807
131.610
131.410
130.956
129.842
129.304
129.034
129.008
128.701
128.546
128.432
128.354
128.326
127.712
124.094
122.548
122.389
122.123
122.044
121.798
121.775
83.889
77.254
77.000
76.747
62.758
29.040
11.412
N–OMe
O
Ph
Br
30 (1.2:1)
00 190 180 170 160 150 140 130 120 110 100 90 80 70 60 50 40 30 20 10 0
f1 (ppm)

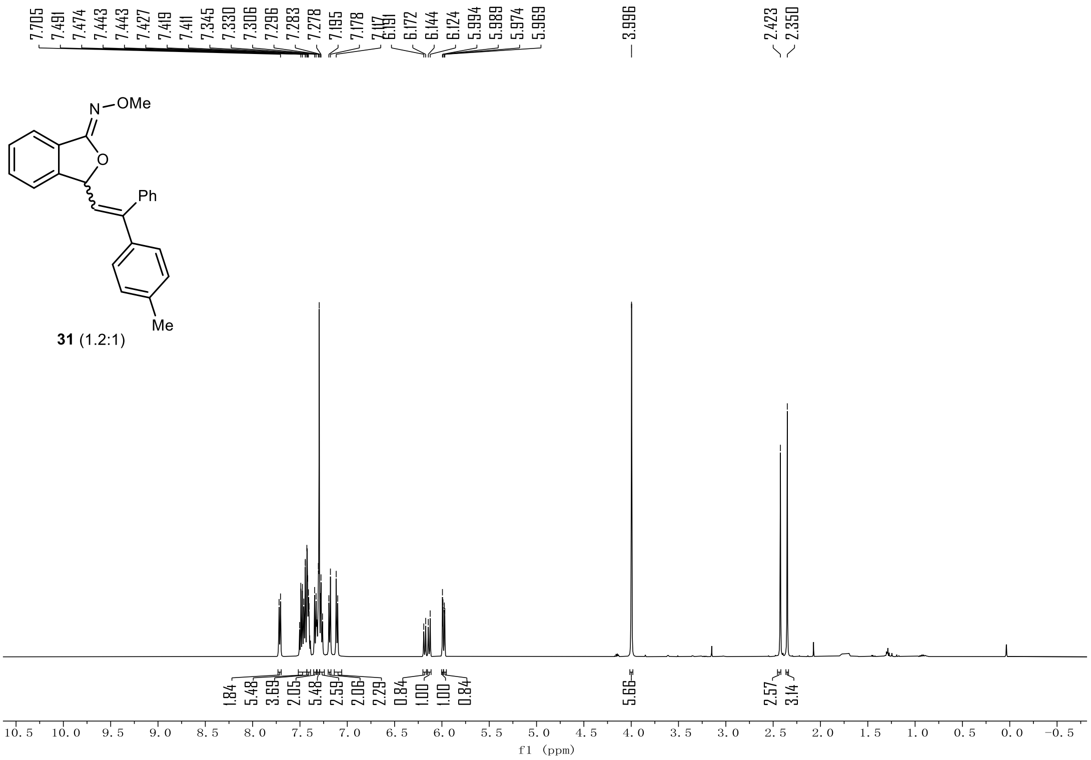
7.705 7.491 7.474 7.443 7.443 7.427 7.419 7.411 7.345 7.330 7.306 7.296 7.283 7.278 7.195 7.178 7.117 6.191 6.172 6.144 6.124 5.994 5.989 5.974 5.969
3.996
2.423 2.350
N–OMe
O
Ph
Me
31 (1.2:1)
1.84 5.48 3.69 2.05 5.48 2.59 2.06 2.29 0.84 1.00 1.00 0.84
5.66
2.57 3.14
10.5 10.0 9.5 9.0 8.5 8.0 7.5 7.0 6.5 6.0 5.5 5.0 4.5 4.0 3.5 3.0 2.5 2.0 1.5 1.0 0.5 0.0 -0.5
f1 (ppm)

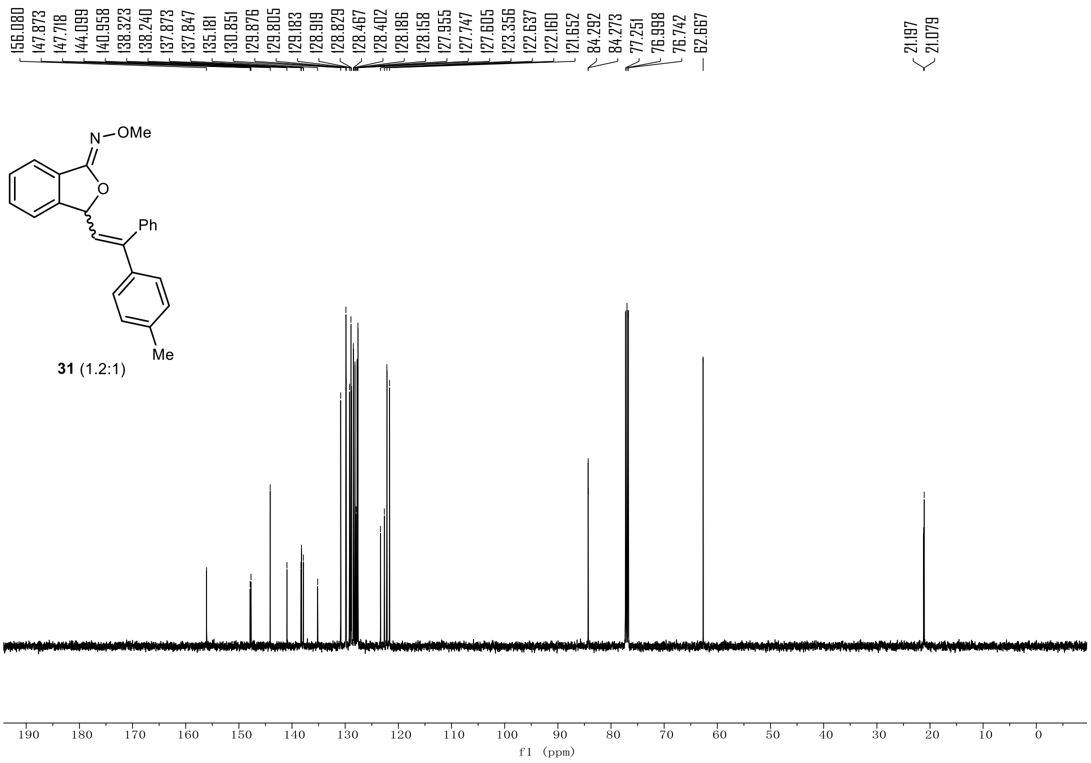
156.080 147.873 147.718 144.099 140.958 138.323 138.240 137.873 137.847 135.181 130.851 129.876 129.805 129.183 128.919 128.829 128.467 128.402 128.186 128.158 127.955 127.747 127.605 123.356 122.637 122.160 121.652
84.292 84.273 77.251 76.998 76.742 62.667
21.197 21.079
N–OMe
O
Ph
Me
31 (1.2:1)
190 180 170 160 150 140 130 120 110 100 90 80 70 60 50 40 30 20 10 0
f1 (ppm)

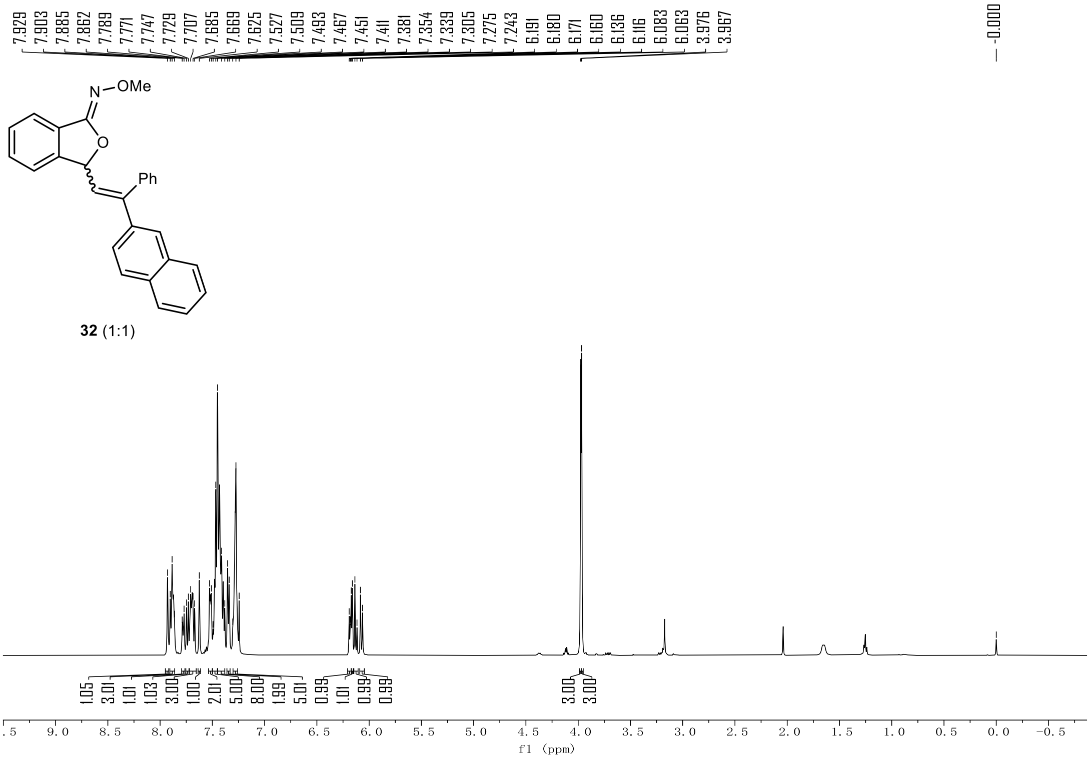
7.929 7.903 7.885 7.862 7.789 7.771 7.747 7.729 7.707 7.685 7.669 7.625 7.527 7.509 7.493 7.467 7.451 7.411 7.381 7.354 7.339 7.305 7.275 7.243 6.191 6.180 6.171 6.160 6.136 6.116 6.083 6.063 3.976 3.967
-0.000
N
OMe
O
Ph
1.05 3.01 1.01 1.03 3.00 1.00 2.01 5.00 8.00 1.99 5.01 0.99 1.01 0.99 0.99
3.00 3.00
f1 (ppm)

32 (1:1)

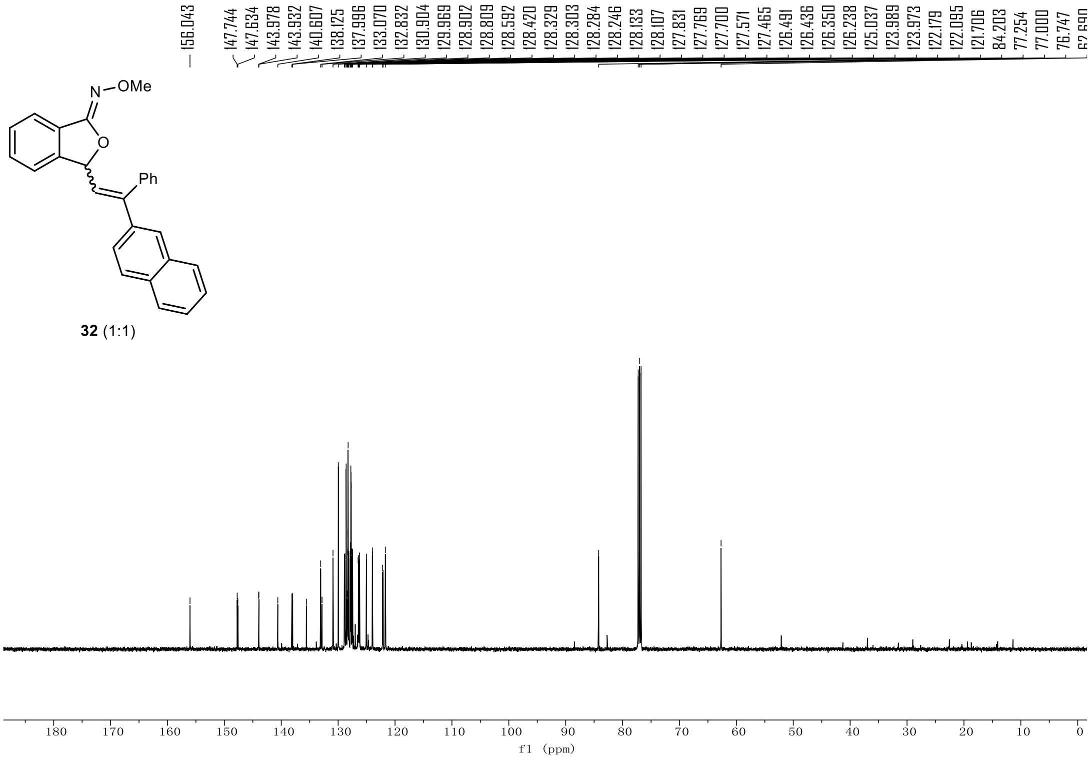
156.043 147.744 147.634 143.978 143.932 140.607 138.125 137.996 133.070 132.832 130.904 129.969 128.902 128.809 128.592 128.420 128.329 128.303 128.284 128.246 128.133 128.107 127.831 127.769 127.700 127.571 127.465 126.491 126.436 126.350 126.238 125.037 123.989 123.973 122.179 122.095 121.706 84.203 77.254 77.000 76.747
N
OMe
O
Ph
f1 (ppm)

32 (1:1)

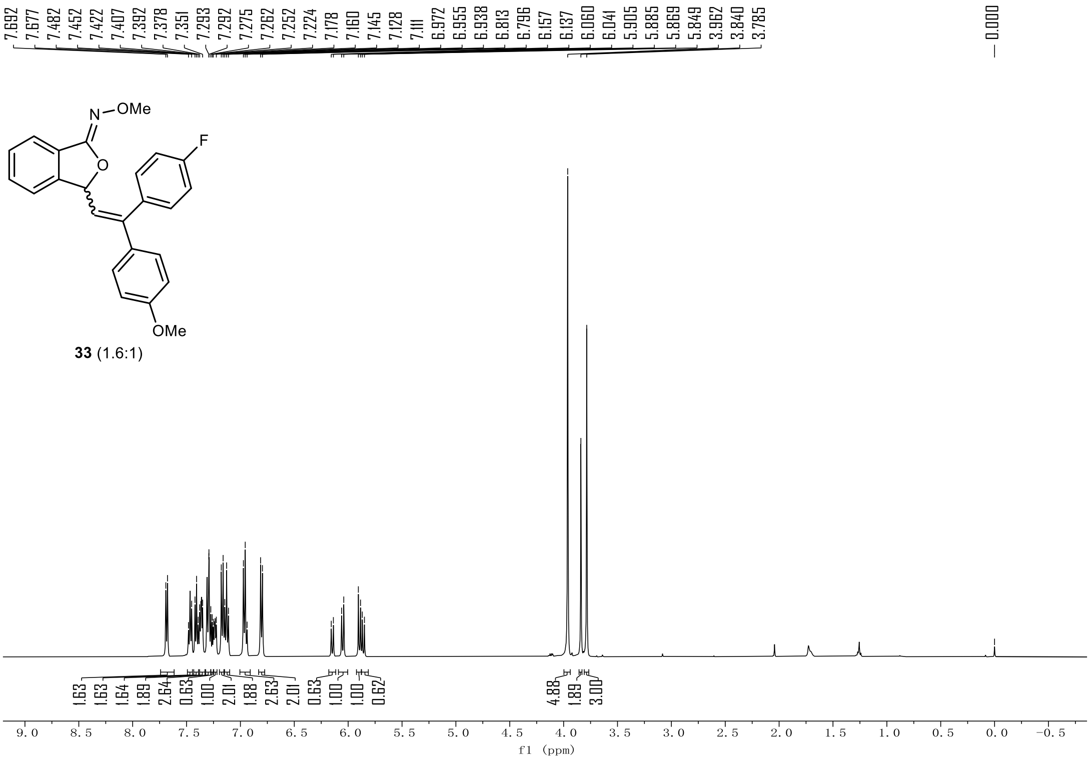
7.692
7.677
7.482
7.452
7.422
7.407
7.392
7.378
7.351
7.293
7.292
7.275
7.262
7.252
7.224
7.178
7.160
7.145
7.128
7.111
6.972
6.955
6.938
6.813
6.796
6.157
6.137
6.060
6.041
5.905
5.885
5.869
5.849
3.962
3.840
3.785
0.000
N–OMe
F
O
OMe
33 (1.6:1)
1.63
1.63
1.64
1.89
2.64
0.63
1.00
2.01
1.88
2.63
2.01
0.63
1.00
1.00
0.62
4.88
1.89
3.00
f1 (ppm)

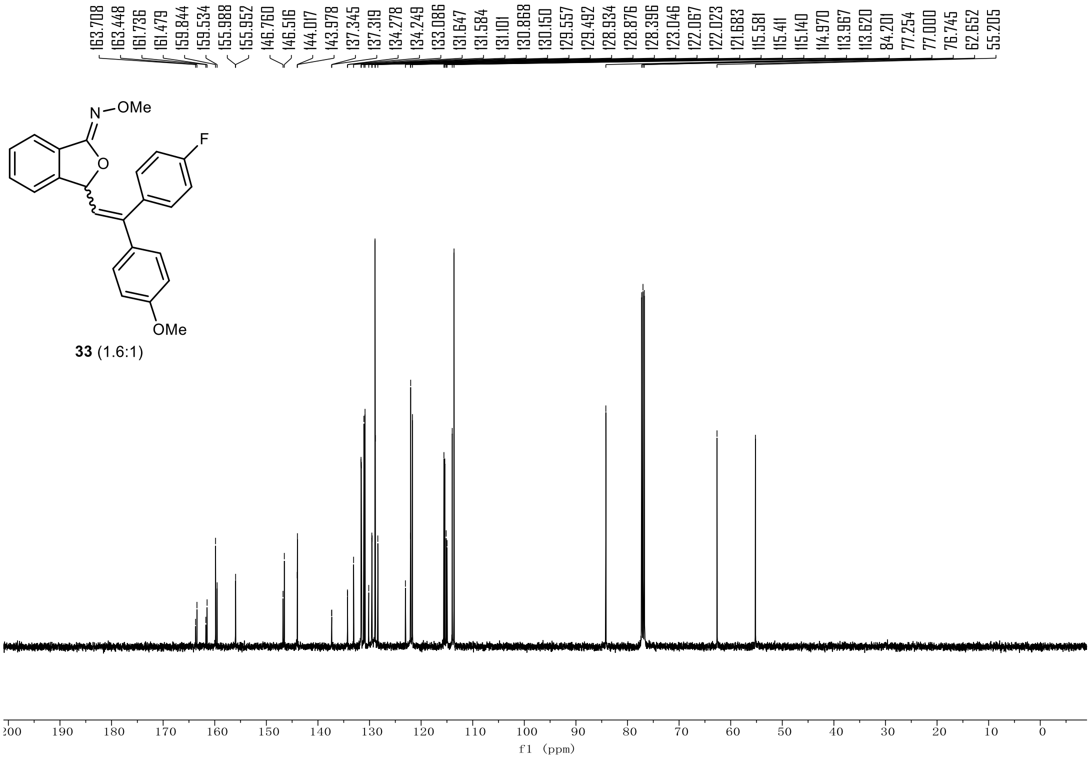
163.708
163.448
161.736
161.479
159.844
159.534
155.988
155.952
146.760
146.516
144.017
143.978
137.345
137.319
134.278
134.249
133.086
131.647
131.584
131.101
130.868
130.150
129.557
129.492
128.934
128.876
128.396
123.046
122.067
122.023
121.683
115.581
115.411
115.140
114.970
113.967
113.620
84.201
77.254
77.000
76.745
62.652
55.205
N–OMe
F
O
OMe
33 (1.6:1)
f1 (ppm)

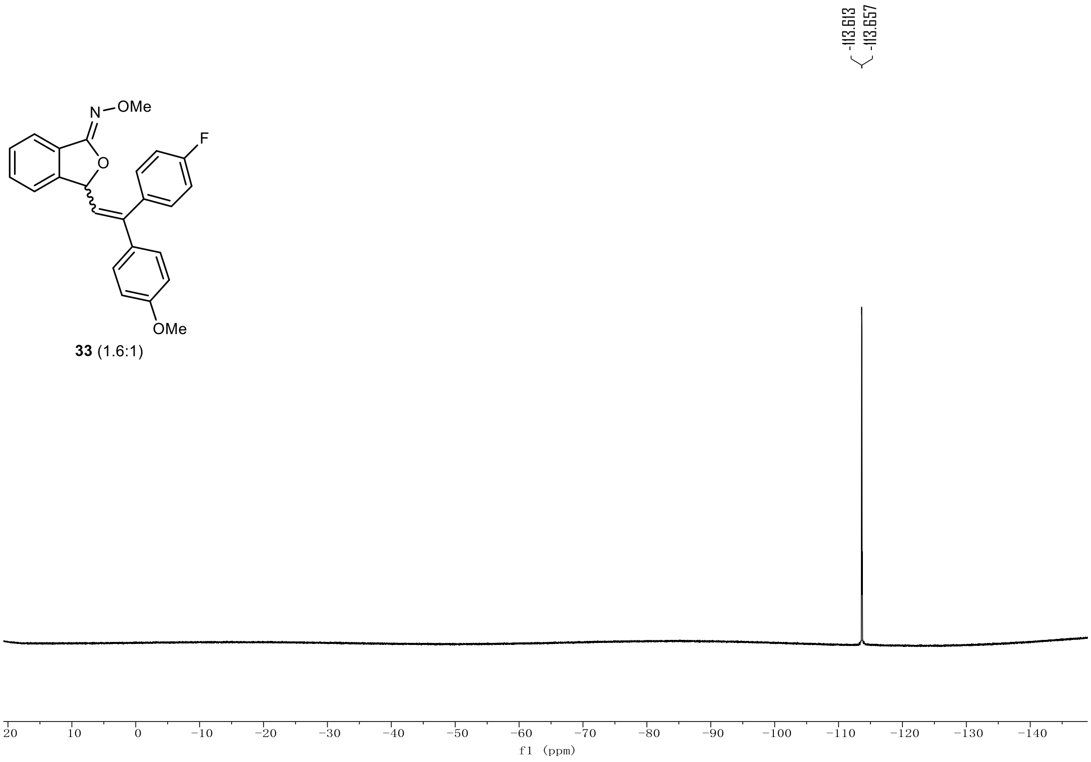
-113.613
-113.657
N
OMe
F
O
OMe
33 (1.6:1)
f1 (ppm)

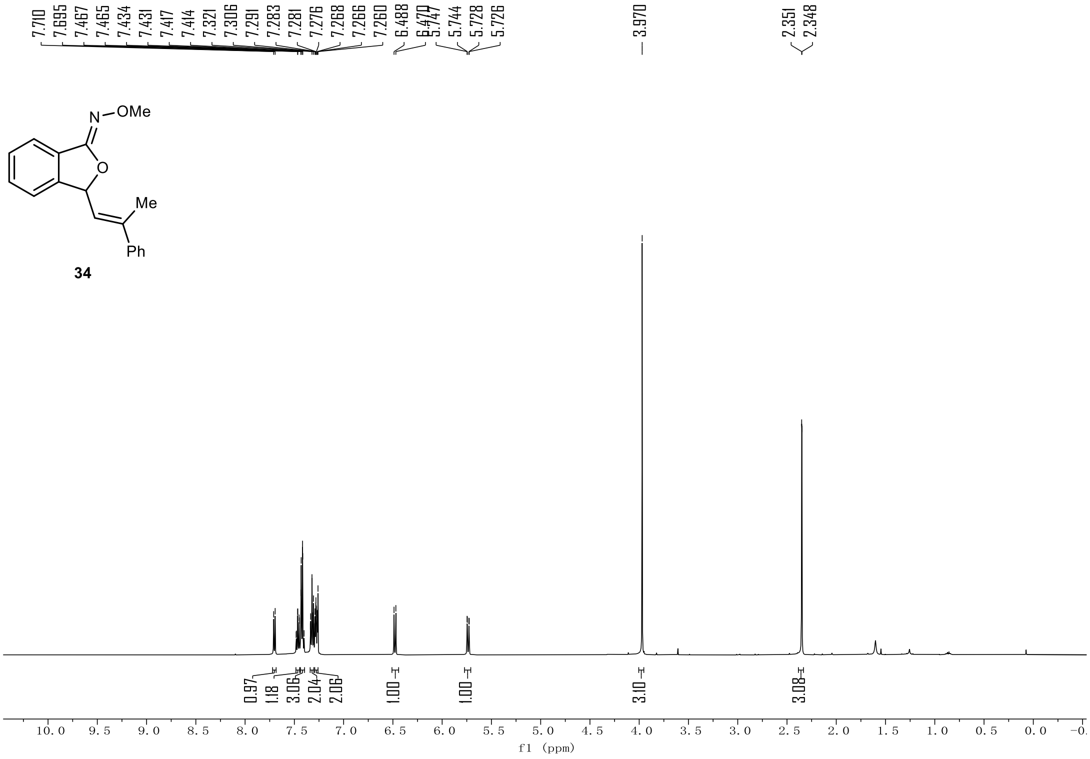
7.710
7.695
7.467
7.465
7.434
7.431
7.417
7.414
7.321
7.306
7.291
7.283
7.281
7.276
7.268
7.266
7.260
6.488
6.470
5.747
5.744
5.728
5.726
3.970
2.351
2.348
N
OMe
O
Me
Ph
34
0.97
1.18
3.06
2.04
2.06
1.00
1.00
3.10
3.08
f1 (ppm)

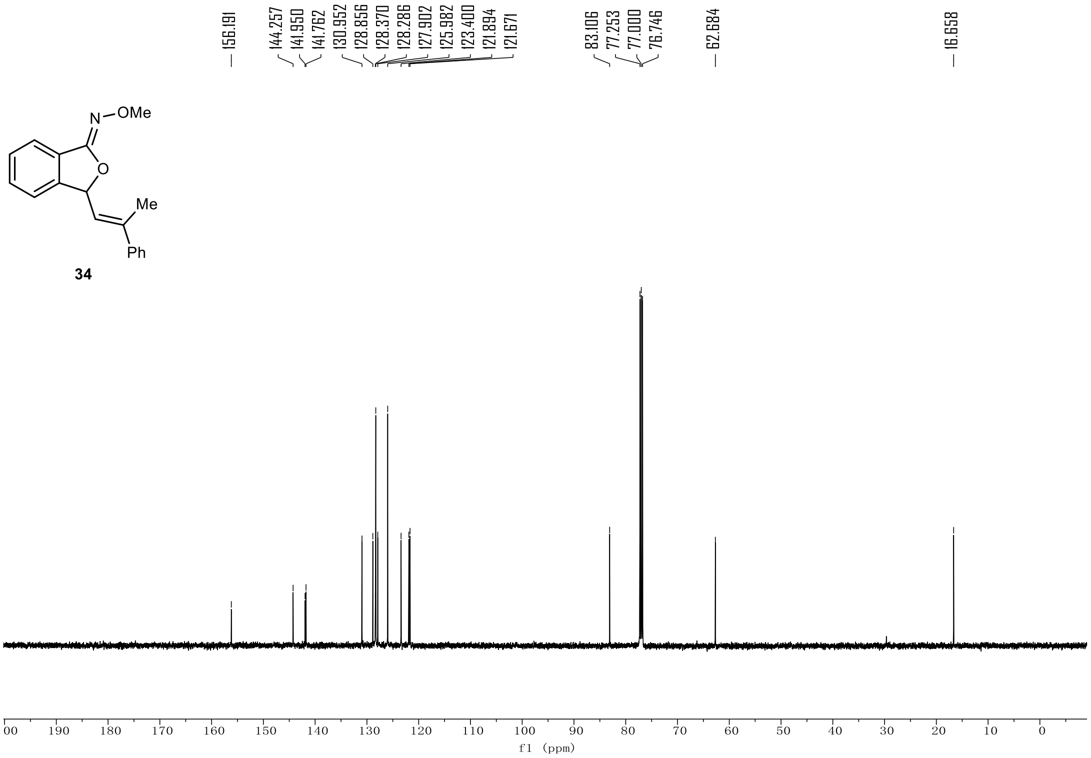

156.191
144.257
141.950
141.762
130.952
128.856
128.370
128.286
127.902
125.982
123.400
121.894
121.671
83.106
77.253
77.000
76.746
62.684
16.658
N–OMe
O
Me
Ph
f1 (ppm)

34

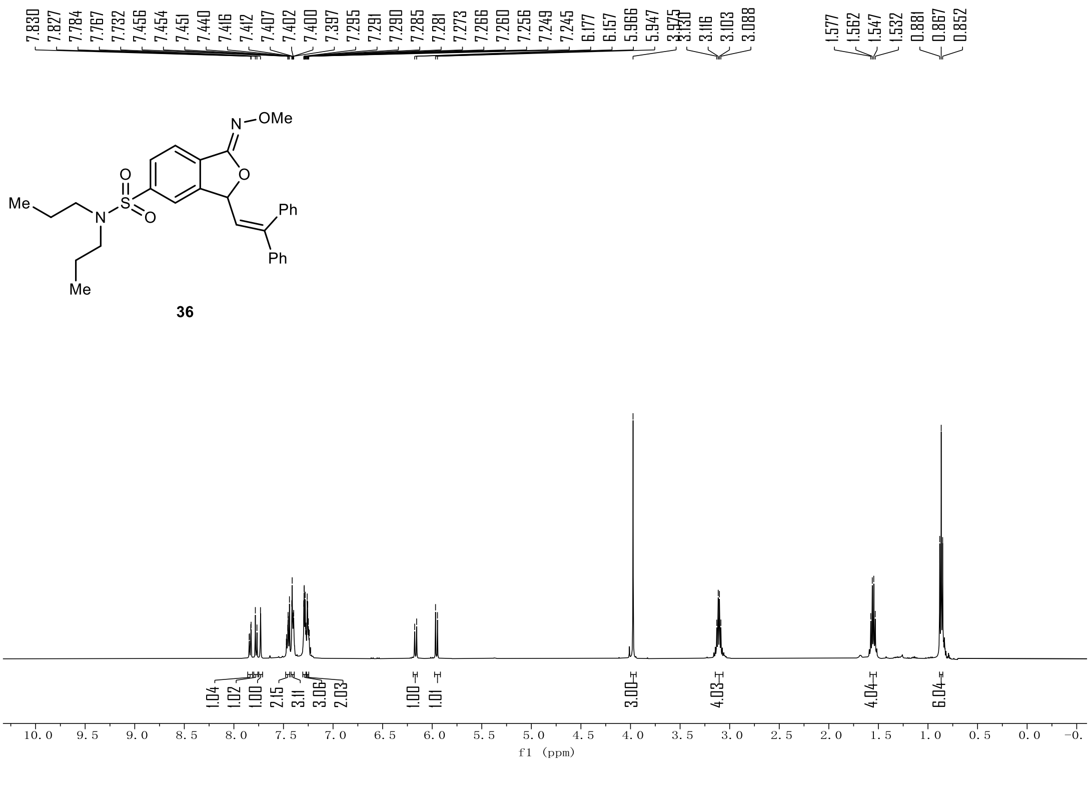

7.830
7.827
7.784
7.767
7.732
7.456
7.454
7.451
7.440
7.416
7.412
7.407
7.402
7.400
7.397
7.295
7.291
7.290
7.285
7.281
7.273
7.266
7.260
7.256
7.249
7.245
6.177
6.157
5.966
5.947
3.975
3.130
3.116
3.103
3.088
1.577
1.562
1.547
1.532
0.881
0.867
0.852
N–OMe
O
S
N
Me
Me
Ph
Ph
1.04
1.02
1.00
2.15
3.11
3.06
2.03
1.00
1.01
3.00
4.03
4.04
6.04
f1 (ppm)

36

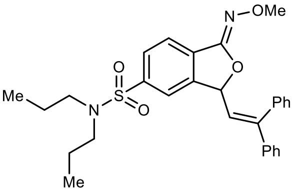
N–OMe
O
Me
N
S
O
O
Ph
Ph
Me

36

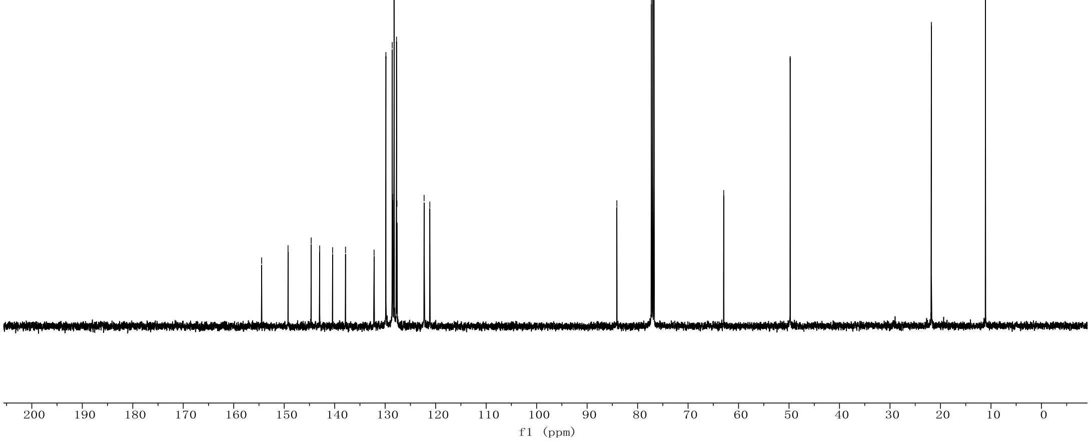
154.471
149.247
144.657
142.973
140.417
137.861
132.203
129.856
128.606
128.540
128.252
127.760
127.667
122.294
122.246
121.153
84.103
77.255
77.000
76.746
62.942
49.779
21.810
11.092
200 190 180 170 160 150 140 130 120 110 100 90 80 70 60 50 40 30 20 10 0
f1 (ppm)

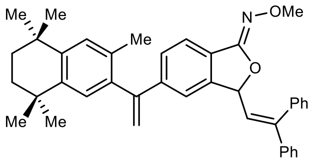
Me
Me
Me
N–OMe
O
Me
Me
Ph
Ph

37

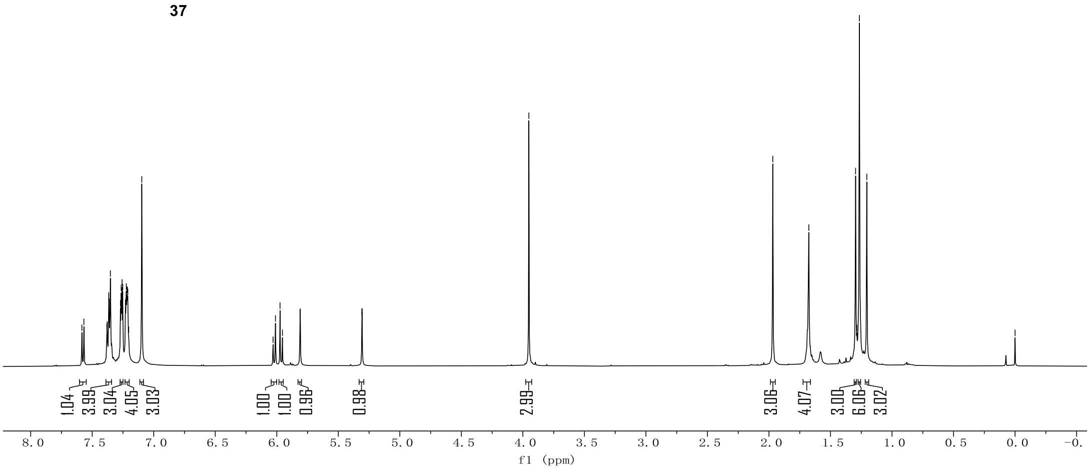
7.584
7.568
7.381
7.378
7.366
7.363
7.358
7.352
7.271
7.264
7.257
7.252
7.230
7.226
7.223
7.218
7.214
7.211
7.203
7.097
6.030
6.011
5.974
5.954
5.811
5.809
5.309
5.306
3.951
1.969
1.676
1.296
1.286
1.265
1.205
0.000
1.04
3.99
3.04
4.05
3.03
1.00
1.00
0.96
0.98
2.99
3.06
4.07
3.00
6.06
3.02
8.0 7.5 7.0 6.5 6.0 5.5 5.0 4.5 4.0 3.5 3.0 2.5 2.0 1.5 1.0 0.5 0.0 -0.
f1 (ppm)

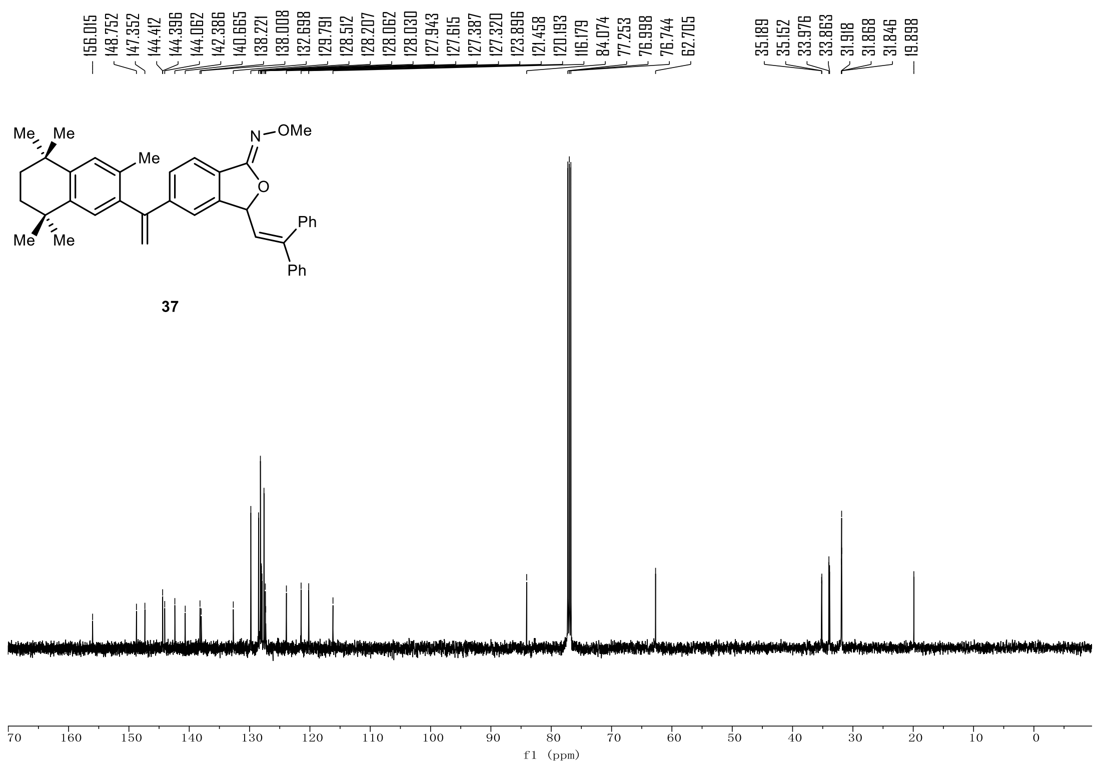
156.015
148.752
147.352
144.412
144.396
144.062
142.386
140.665
138.221
138.008
132.698
129.791
128.512
128.207
128.062
128.030
127.943
127.615
127.387
127.320
123.896
121.458
120.193
116.179
84.074
77.253
76.998
76.744
62.705
35.189
35.152
33.976
33.863
31.918
31.868
31.846
19.898
Me
Me
Me
Me
Me
N–OMe
O
Ph
Ph
37
70 160 150 140 130 120 110 100 90 80 70 60 50 40 30 20 10 0
f1 (ppm)

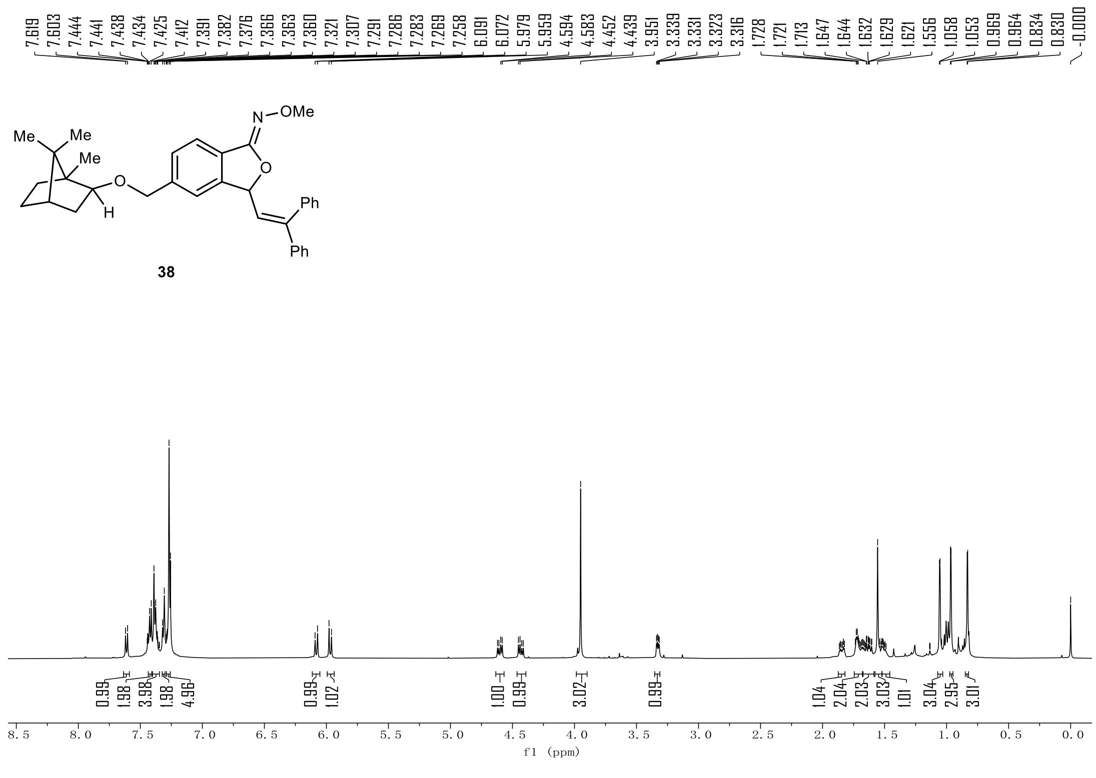
7.619
7.603
7.444
7.441
7.438
7.434
7.425
7.412
7.391
7.382
7.376
7.366
7.363
7.360
7.321
7.307
7.291
7.286
7.283
7.269
7.258
6.091
6.072
5.979
5.959
4.594
4.583
4.452
4.439
3.951
3.339
3.331
3.323
3.316
1.728
1.721
1.713
1.647
1.644
1.632
1.629
1.621
1.556
1.058
1.053
0.969
0.964
0.834
0.830
-0.000
Me
Me
Me
O
H
N–OMe
O
Ph
Ph
38
0.99
1.98
3.98
1.98
4.96
0.99
1.02
1.00
0.99
3.02
0.99
1.04
2.04
2.03
3.03
1.01
3.04
2.95
3.01
8.5 8.0 7.5 7.0 6.5 6.0 5.5 5.0 4.5 4.0 3.5 3.0 2.5 2.0 1.5 1.0 0.5 0.0
f1 (ppm)

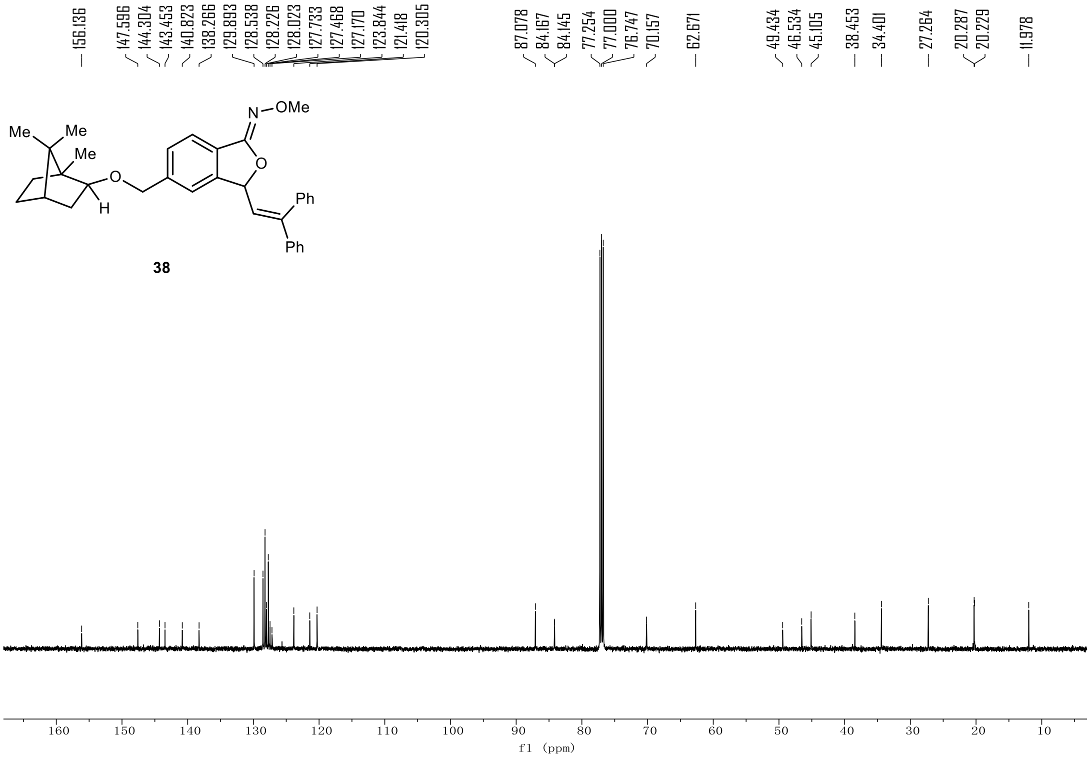
156.136
147.596
144.304
143.453
140.823
138.266
129.893
128.538
128.226
128.023
127.733
127.468
127.170
123.844
121.418
120.305
87.078
84.167
84.145
77.254
77.000
76.747
70.157
62.671
49.434
46.534
45.105
38.453
34.401
27.264
20.287
20.229
11.978
N–OMe
Me
Me
Me
O
O
H
Ph
Ph
38
160 150 140 130 120 110 100 90 80 70 60 50 40 30 20 10
f1 (ppm)

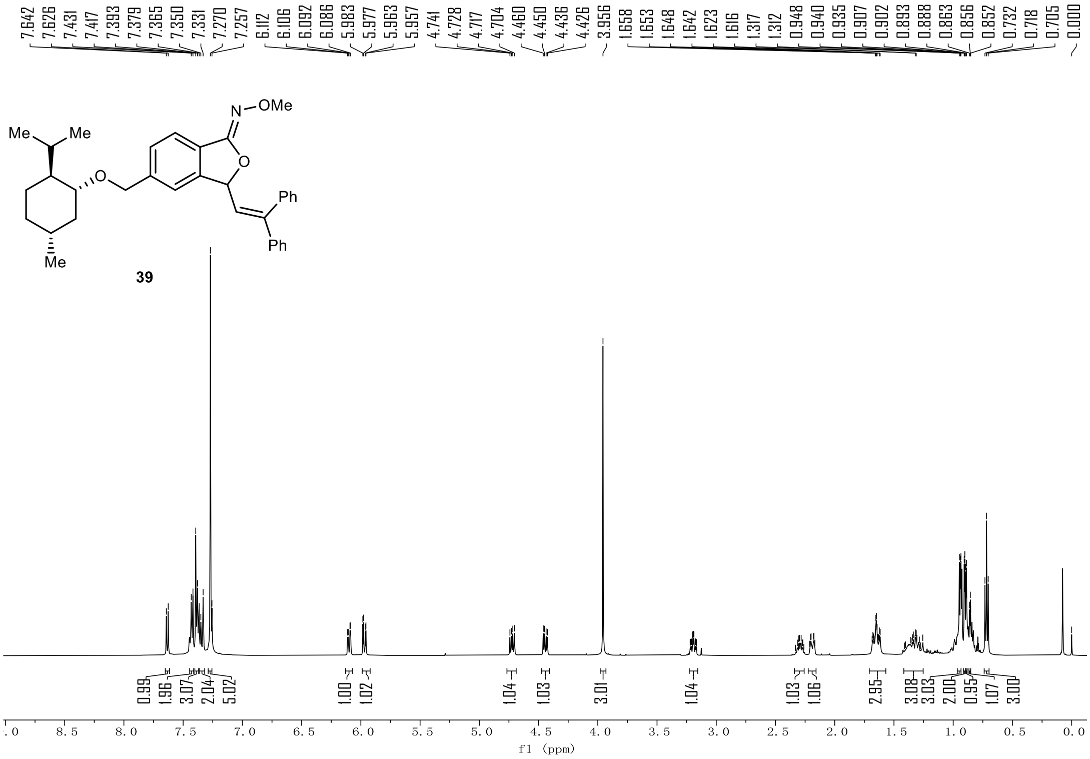
7.642
7.626
7.431
7.417
7.393
7.379
7.365
7.350
7.331
7.270
7.257
6.112
6.106
6.092
6.086
5.983
5.977
5.963
5.957
4.741
4.728
4.717
4.704
4.460
4.450
4.436
4.426
3.956
1.658
1.653
1.648
1.642
1.623
1.616
1.317
1.312
0.948
0.940
0.935
0.907
0.902
0.893
0.888
0.863
0.856
0.852
0.732
0.718
0.705
0.000
N–OMe
Me
Me
O
O
Me
Ph
Ph
39
0.99
1.96
3.07
2.04
5.02
1.00
1.02
1.04
1.03
3.01
1.04
1.03
1.06
2.95
3.08
3.03
2.00
0.95
1.07
3.00
8.5 8.0 7.5 7.0 6.5 6.0 5.5 5.0 4.5 4.0 3.5 3.0 2.5 2.0 1.5 1.0 0.5 0.0
f1 (ppm)

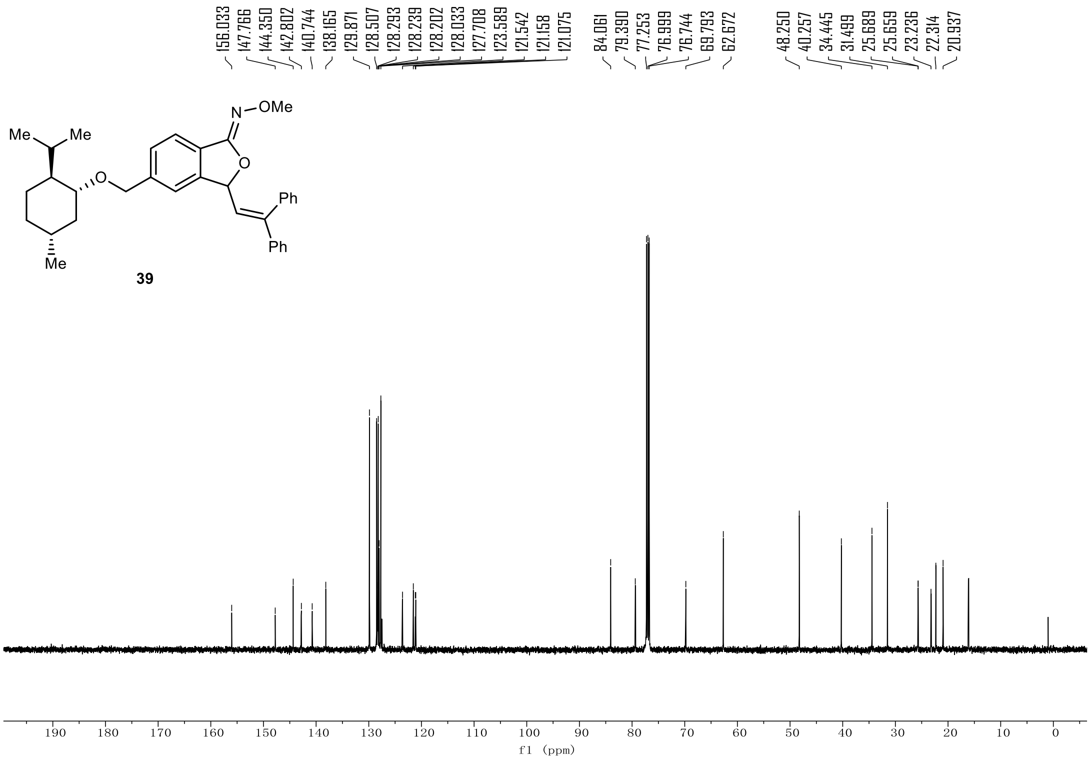
156.033
147.766
144.350
142.802
140.744
138.165
129.871
128.507
128.293
128.239
128.202
128.033
127.708
123.589
121.542
121.158
121.075
84.061
79.390
77.253
76.999
76.744
69.793
62.672
48.250
40.257
34.445
31.499
25.689
25.659
23.236
22.314
20.937
N–OMe
Me
Me
O
O
Ph
Ph
Me
f1 (ppm)

39

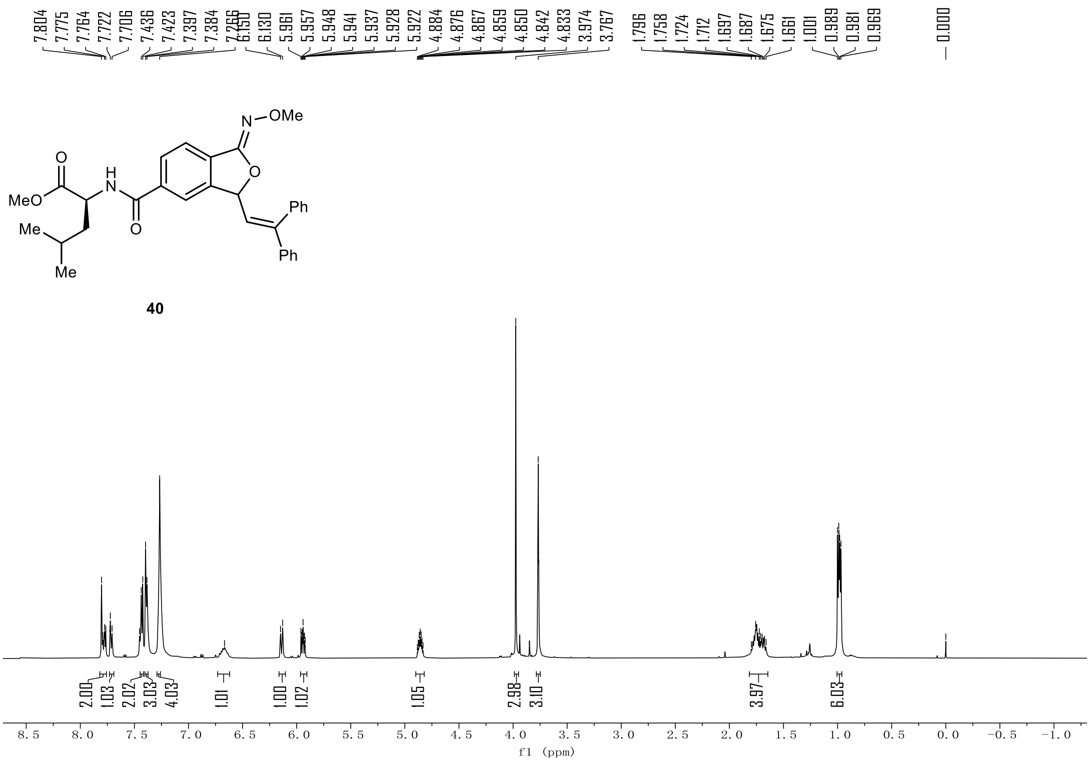
7.804
7.775
7.764
7.722
7.706
7.436
7.423
7.397
7.384
7.266
6.150
6.130
5.961
5.957
5.948
5.941
5.937
5.928
5.922
4.884
4.876
4.867
4.859
4.850
4.842
4.833
3.974
3.767
1.796
1.758
1.724
1.712
1.697
1.687
1.675
1.661
1.001
0.989
0.981
0.969
0.000
N–OMe
O
MeO
H
N
O
O
Ph
Ph
Me
Me
2.00
1.03
2.02
3.03
4.03
1.01
1.00
1.02
1.05
2.98
3.10
3.97
6.03
f1 (ppm)

40

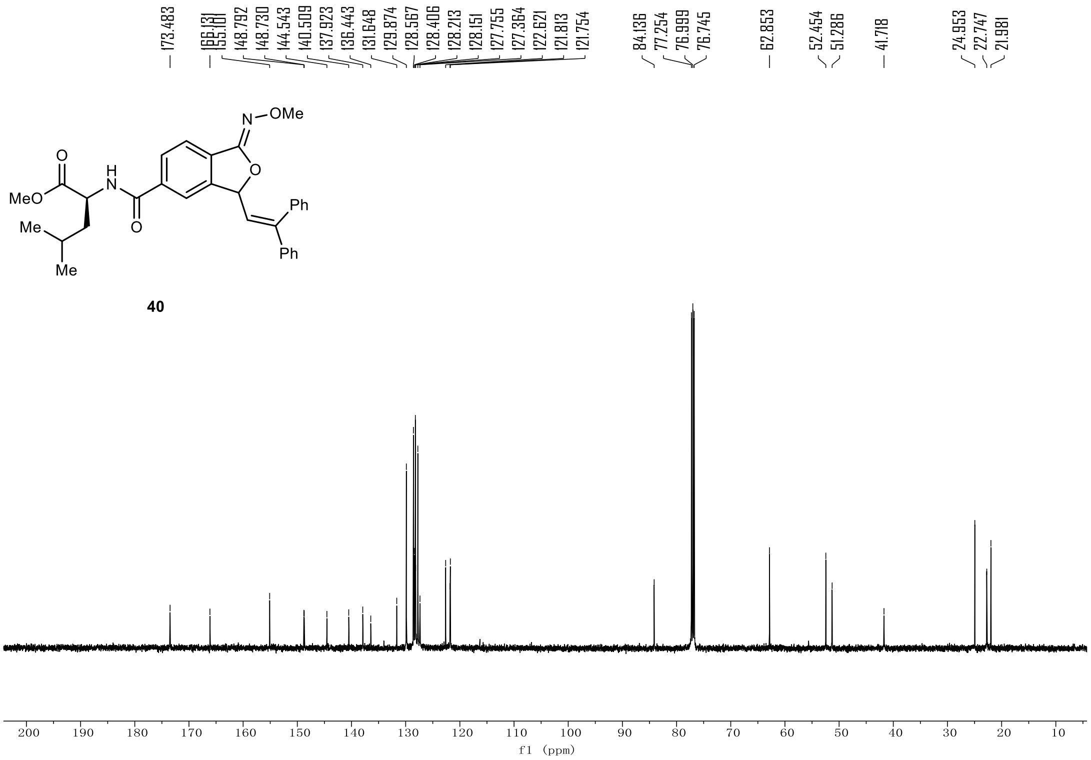

173.483
166.131
155.101
148.792
148.730
144.543
140.509
137.923
136.443
131.648
129.874
128.567
128.406
128.213
128.151
127.755
127.364
122.621
121.813
121.754
84.136
77.254
76.999
76.745
62.853
52.454
51.286
41.718
24.953
22.747
21.981
N–OMe
MeO
Me
Me
Ph
Ph
f1 (ppm)

40

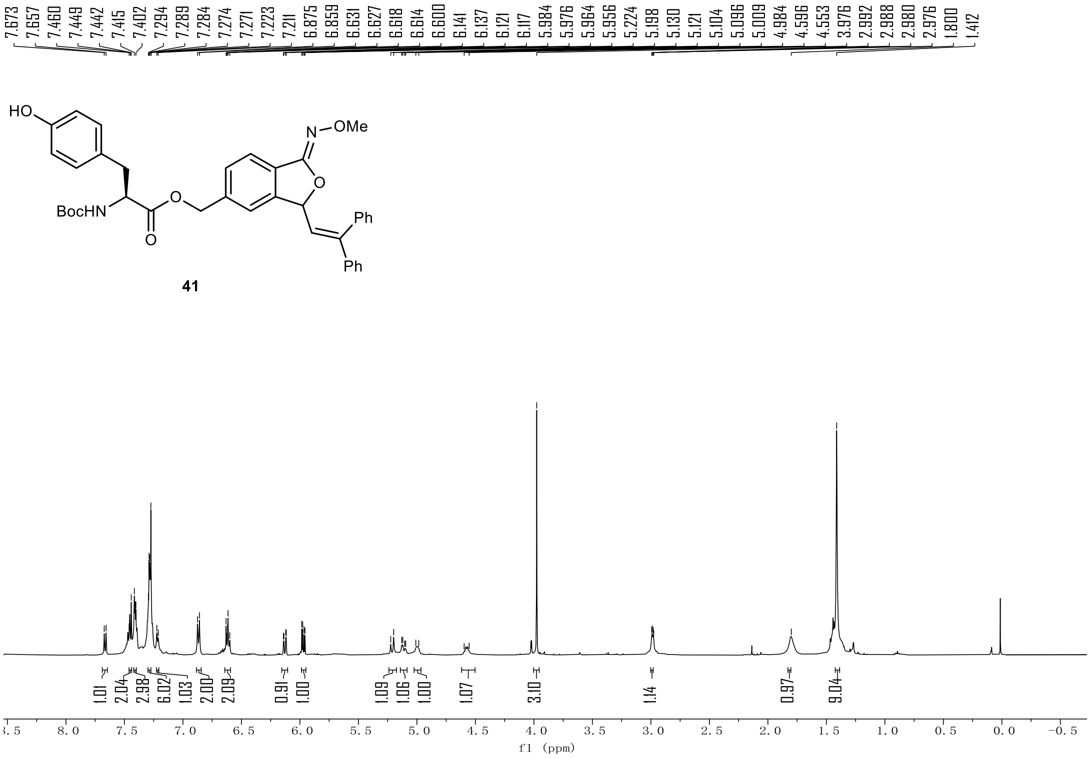

7.673
7.657
7.460
7.449
7.442
7.415
7.402
7.294
7.289
7.284
7.274
7.271
7.223
7.211
6.875
6.859
6.631
6.627
6.618
6.614
6.600
6.141
6.137
6.121
6.117
5.984
5.976
5.964
5.956
5.224
5.198
5.130
5.121
5.104
5.096
5.009
4.984
4.596
4.553
3.976
2.992
2.988
2.980
2.976
1.800
1.412
HO
N–OMe
BocHN
Ph
Ph
1.01
2.04
2.98
6.02
1.03
2.00
2.09
0.91
1.00
1.09
1.06
1.00
1.07
3.10
1.14
0.97
9.04
f1 (ppm)

41

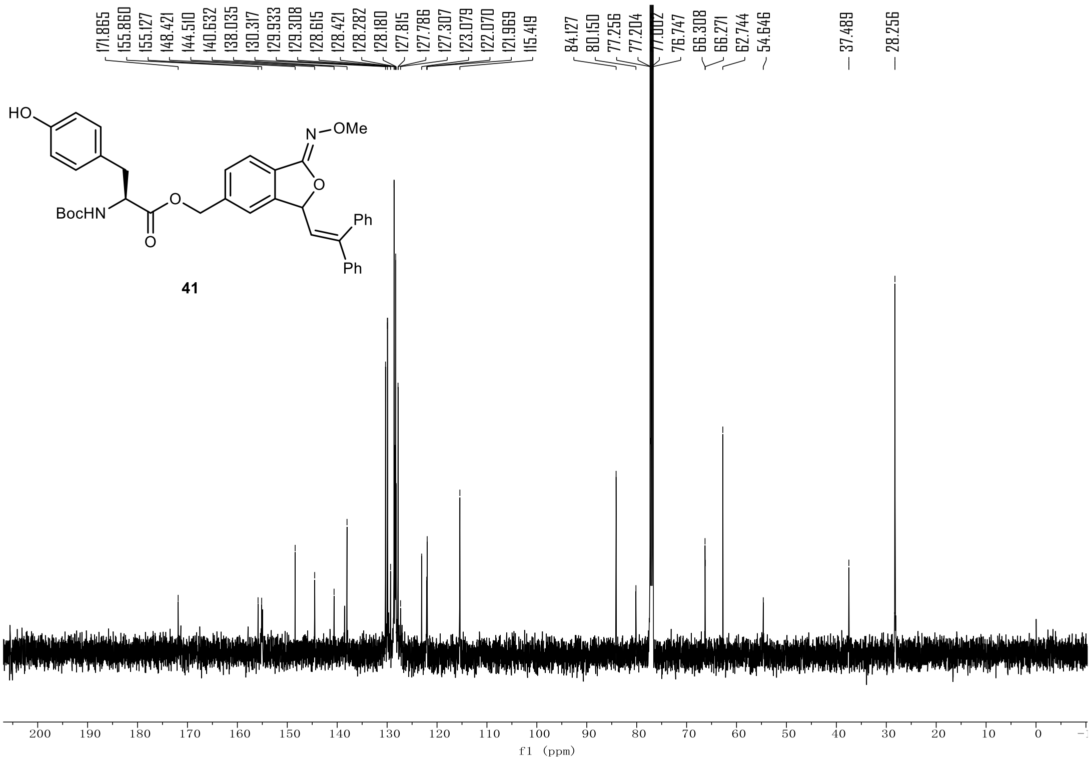

HO
BocHN
O
N
OMe
Ph
Ph
41
f1 (ppm)

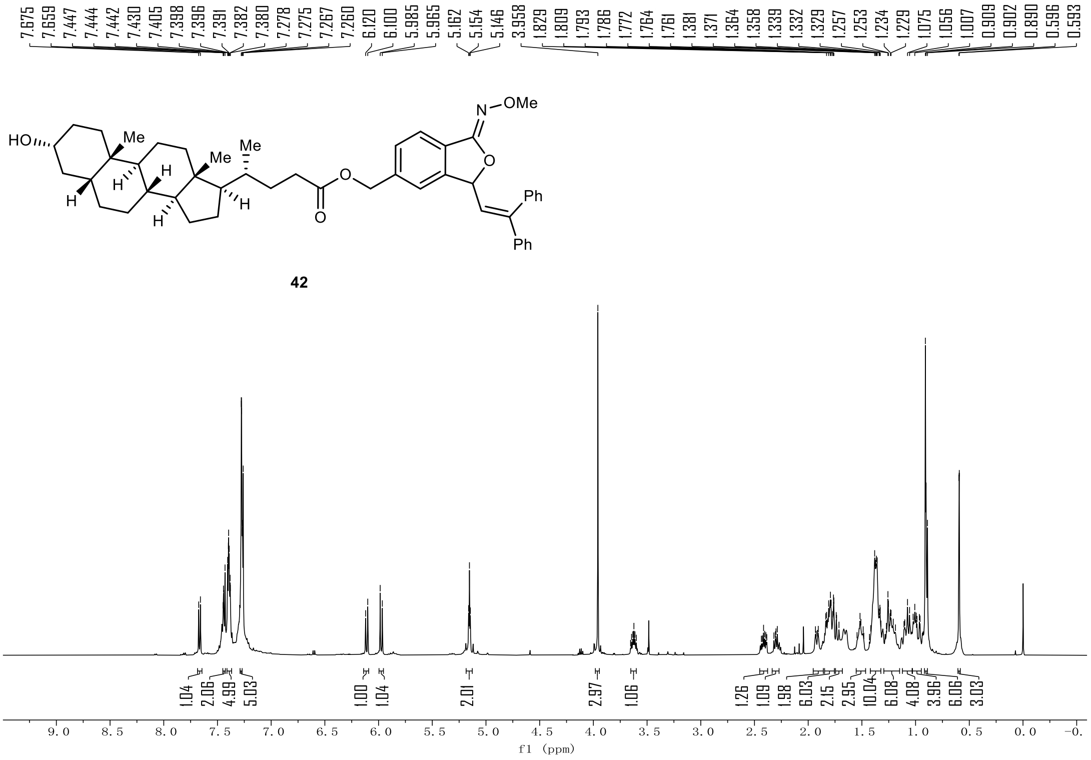

HO
Me
Me
Me
H
H
H
H
O
N
OMe
Ph
Ph
42
f1 (ppm)

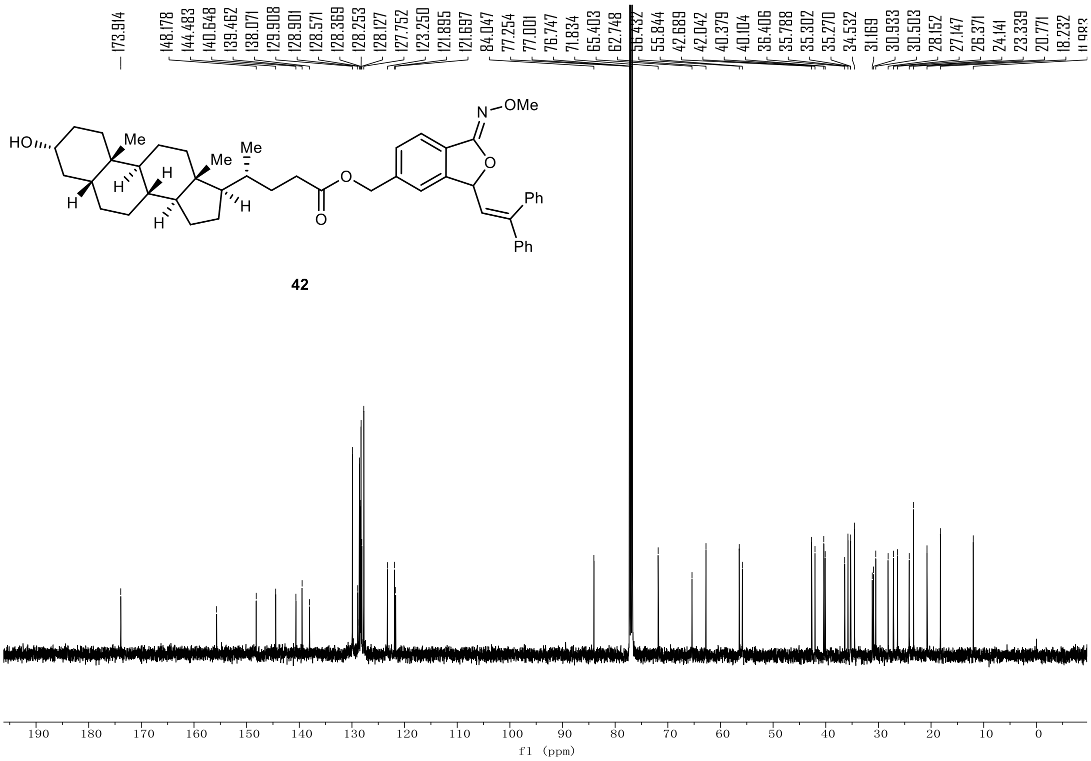

173.914
148.178
144.483
140.648
139.462
138.071
129.908
128.901
128.571
128.369
128.253
128.127
127.752
123.250
121.895
121.697
84.047
77.254
77.001
76.747
71.834
65.403
62.748
56.432
55.844
42.689
42.042
40.379
40.104
36.406
35.788
35.302
35.270
34.532
31.169
30.933
30.503
28.152
27.147
26.371
24.141
23.339
20.771
18.232
HO
Me
H
N
OMe
O
Ph
f1 (ppm)

42

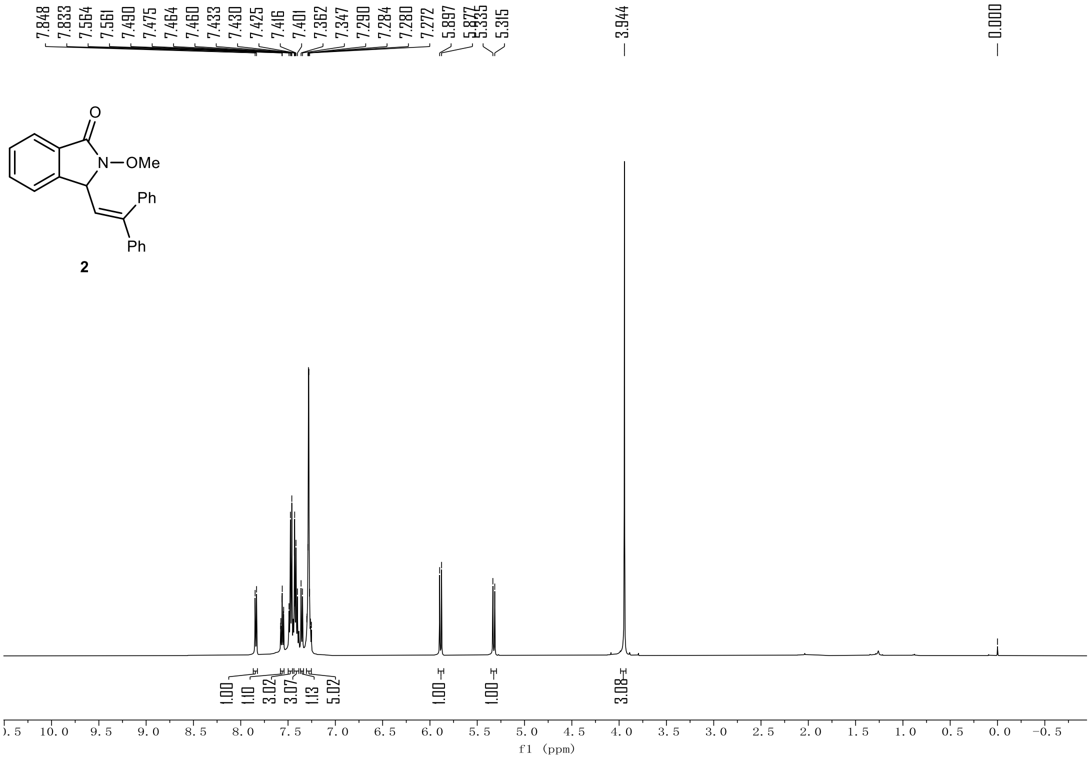

7.848
7.833
7.564
7.561
7.490
7.475
7.464
7.460
7.433
7.430
7.425
7.416
7.401
7.362
7.347
7.290
7.284
7.280
7.272
5.897
5.877
5.335
5.315
3.944
0.000
O
N
OMe
Ph
1.00
1.10
3.02
3.07
1.13
5.02
1.00
1.00
3.08
f1 (ppm)

2

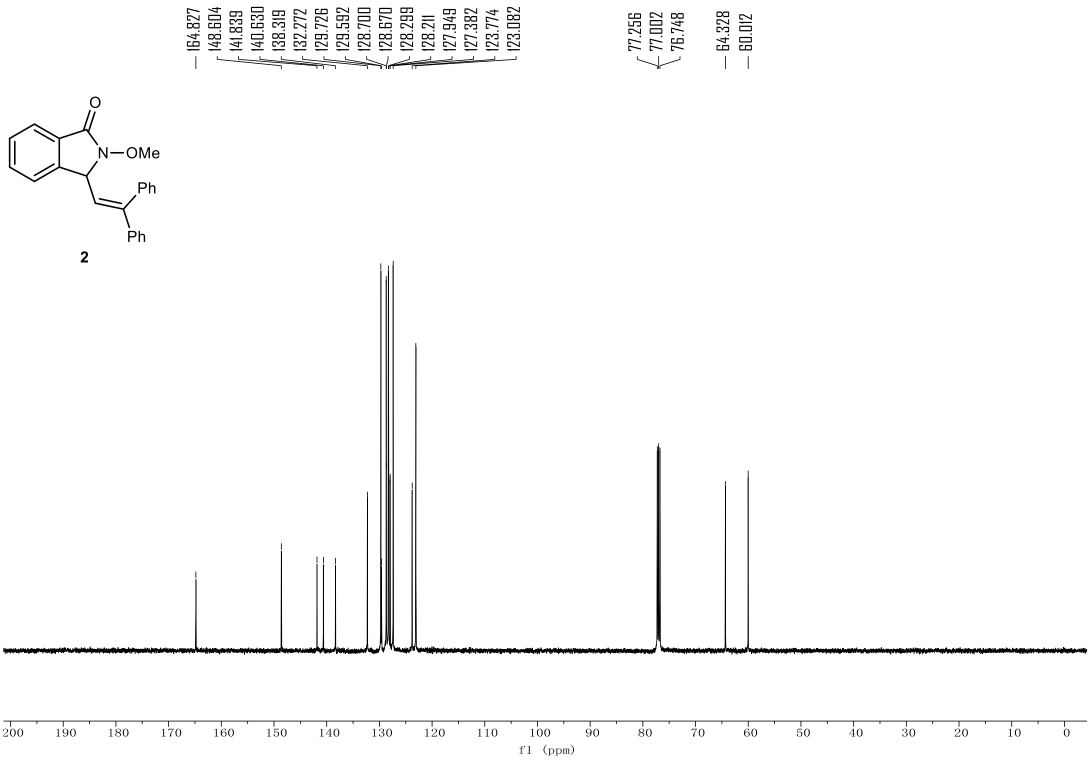

2

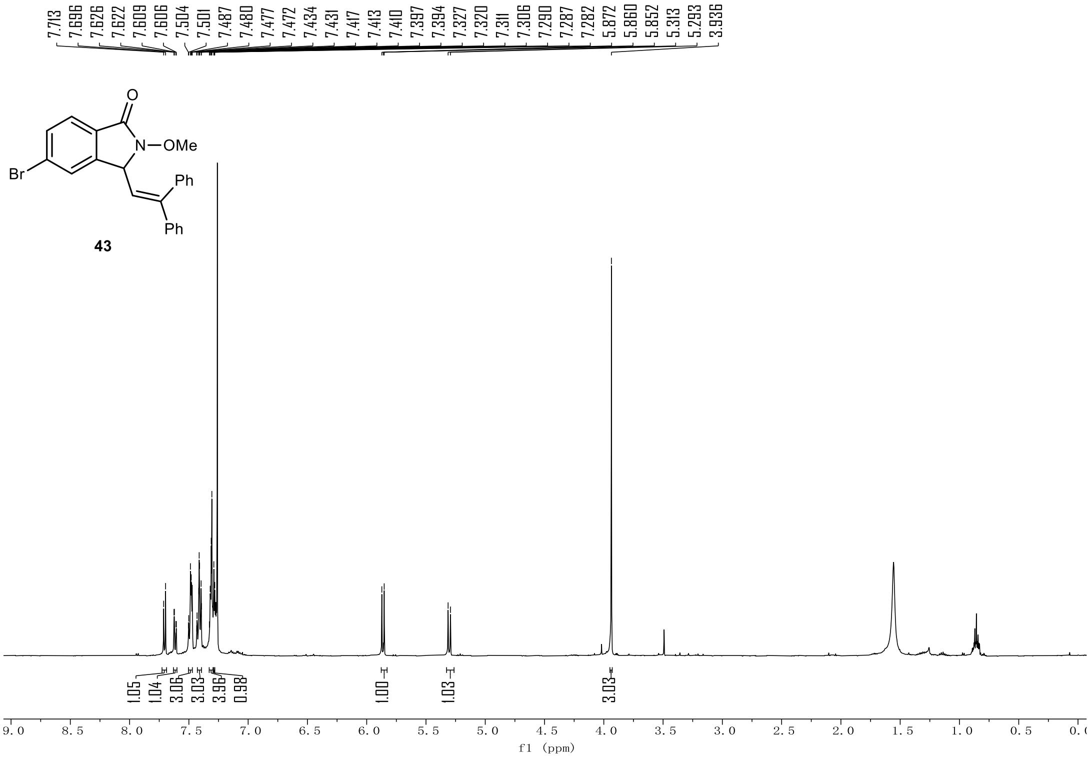

43

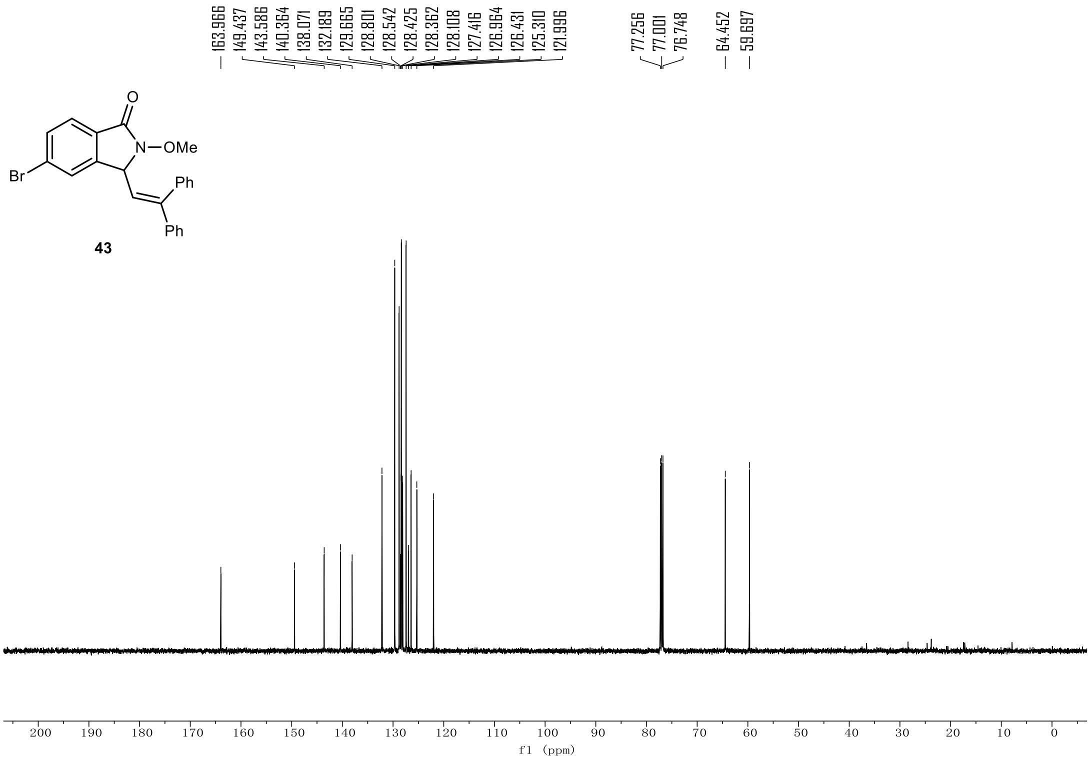
163.966
149.437
143.586
140.364
138.071
132.189
129.665
128.801
128.542
128.425
128.362
128.108
127.416
126.964
126.431
125.310
121.996
77.256
77.001
76.748
64.452
59.697
O
N−OMe
Br
Ph
Ph
43
200 190 180 170 160 150 140 130 120 110 100 90 80 70 60 50 40 30 20 10 0
f1 (ppm)

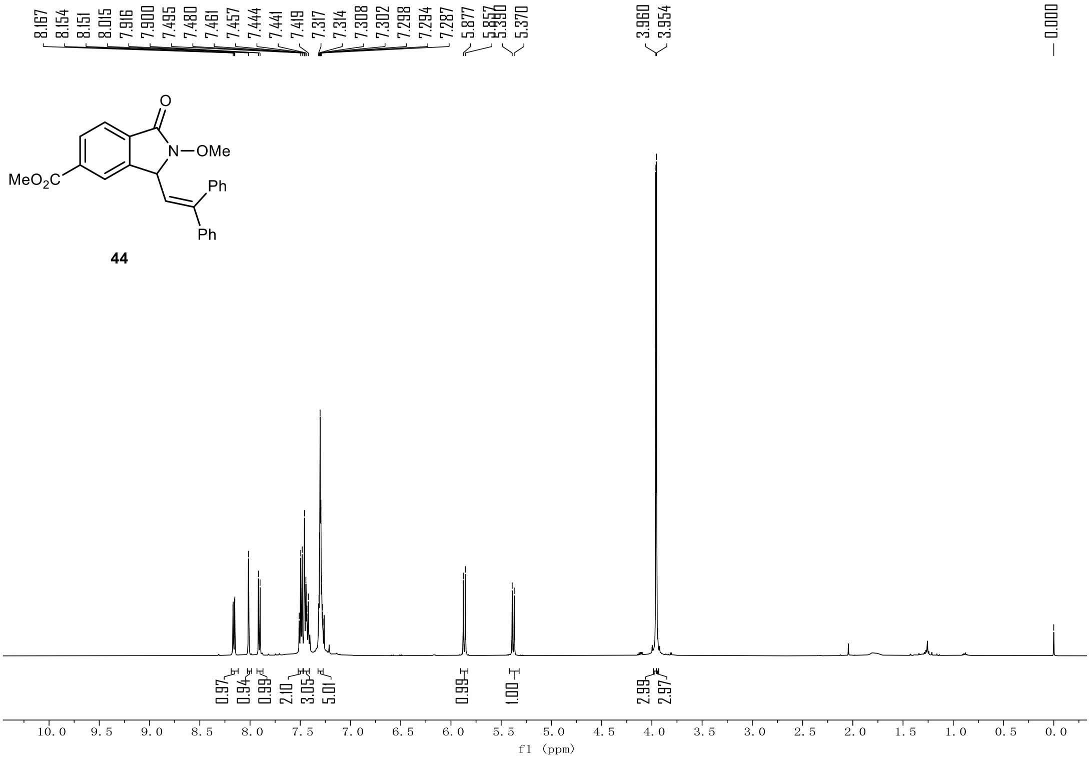
8.167
8.154
8.151
8.015
7.916
7.900
7.495
7.480
7.461
7.457
7.444
7.441
7.419
7.317
7.314
7.308
7.302
7.298
7.294
7.287
5.877
5.857
5.390
5.370
3.960
3.954
0.000
O
N−OMe
MeO_2C
Ph
Ph
44
0.97
0.94
0.99
2.10
3.05
5.01
0.99
1.00
2.99
2.97
10.0 9.5 9.0 8.5 8.0 7.5 7.0 6.5 6.0 5.5 5.0 4.5 4.0 3.5 3.0 2.5 2.0 1.5 1.0 0.5 0.0
f1 (ppm)

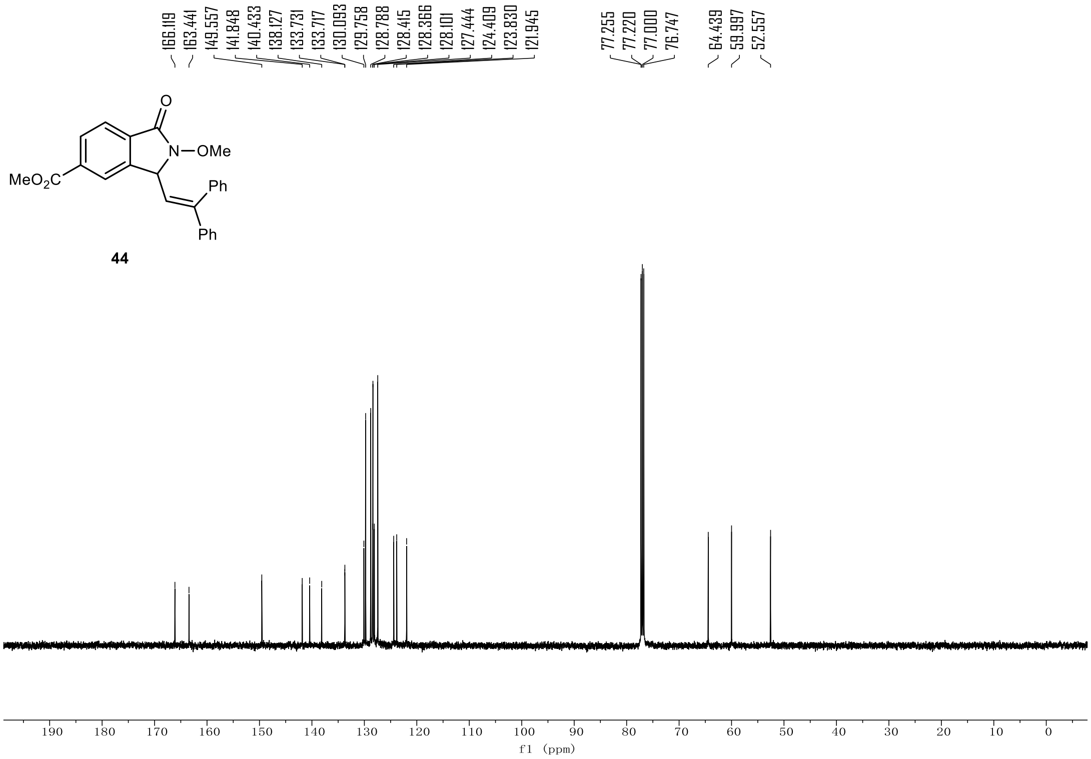
166.119
163.441
149.557
141.848
140.433
138.127
133.731
133.717
130.093
129.758
128.788
128.415
128.366
128.101
127.444
124.409
123.830
121.945
77.255
77.220
77.000
76.747
64.439
59.997
52.557
O
N—OMe
MeO2C
Ph
Ph
190 180 170 160 150 140 130 120 110 100 90 80 70 60 50 40 30 20 10 0
f1 (ppm)

44

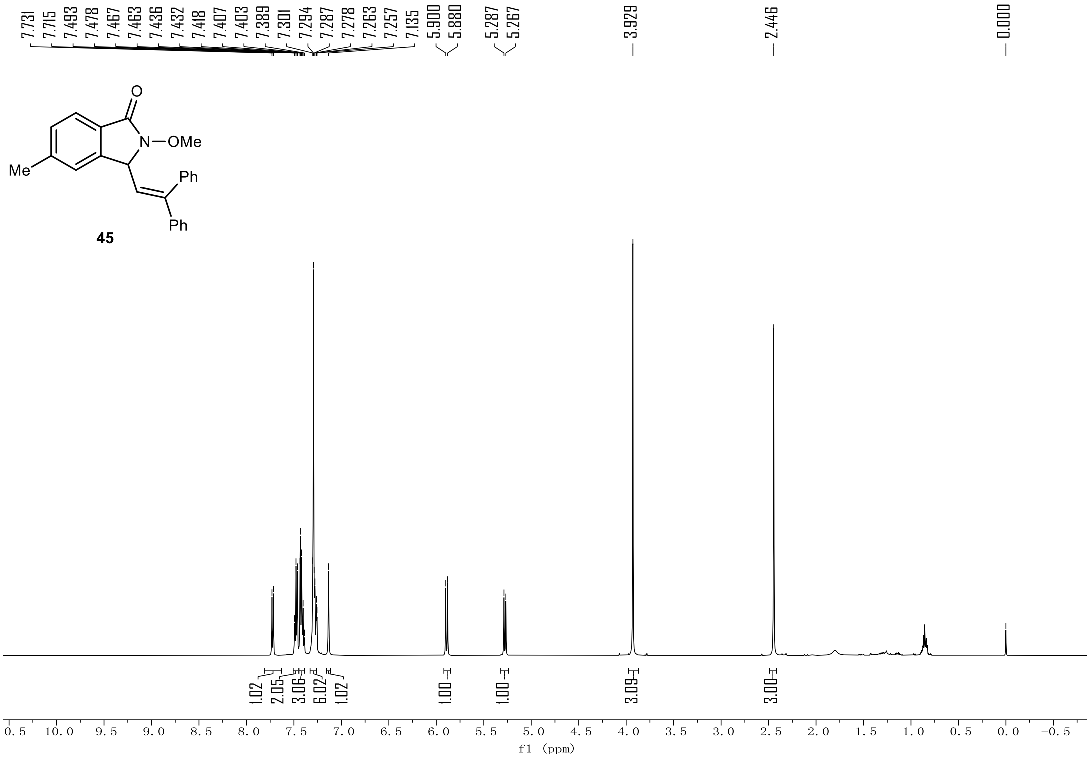
7.731
7.715
7.493
7.478
7.467
7.463
7.436
7.432
7.418
7.407
7.403
7.389
7.301
7.294
7.287
7.278
7.263
7.257
7.135
5.900
5.880
5.287
5.267
3.929
2.446
0.000
O
N—OMe
Me
Ph
Ph
1.02
2.05
3.06
6.02
1.02
1.00
1.00
3.09
3.00
0.5 10.0 9.5 9.0 8.5 8.0 7.5 7.0 6.5 6.0 5.5 5.0 4.5 4.0 3.5 3.0 2.5 2.0 1.5 1.0 0.5 0.0 -0.5
f1 (ppm)

45

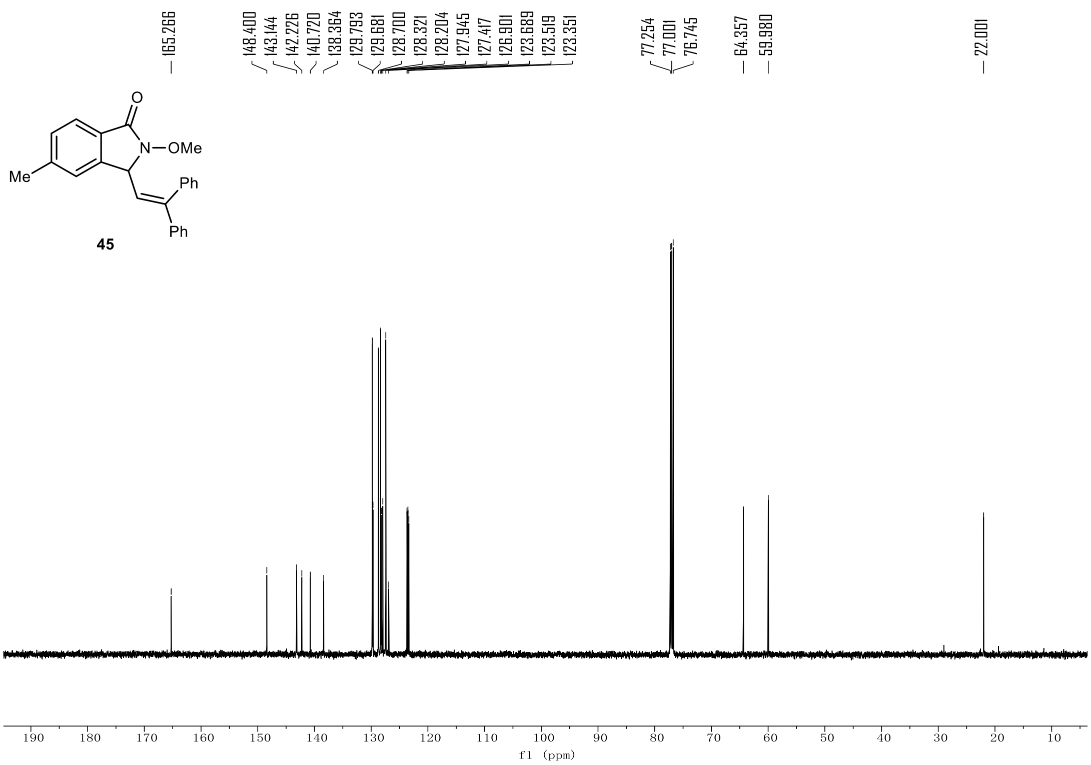
165.266
148.400
143.144
142.226
140.720
138.364
129.793
129.681
128.700
128.321
128.204
127.945
127.417
126.901
123.689
123.519
123.351
77.254
77.001
76.745
64.357
59.980
22.001
O
N−OMe
Me
Ph
Ph
45
f1 (ppm)

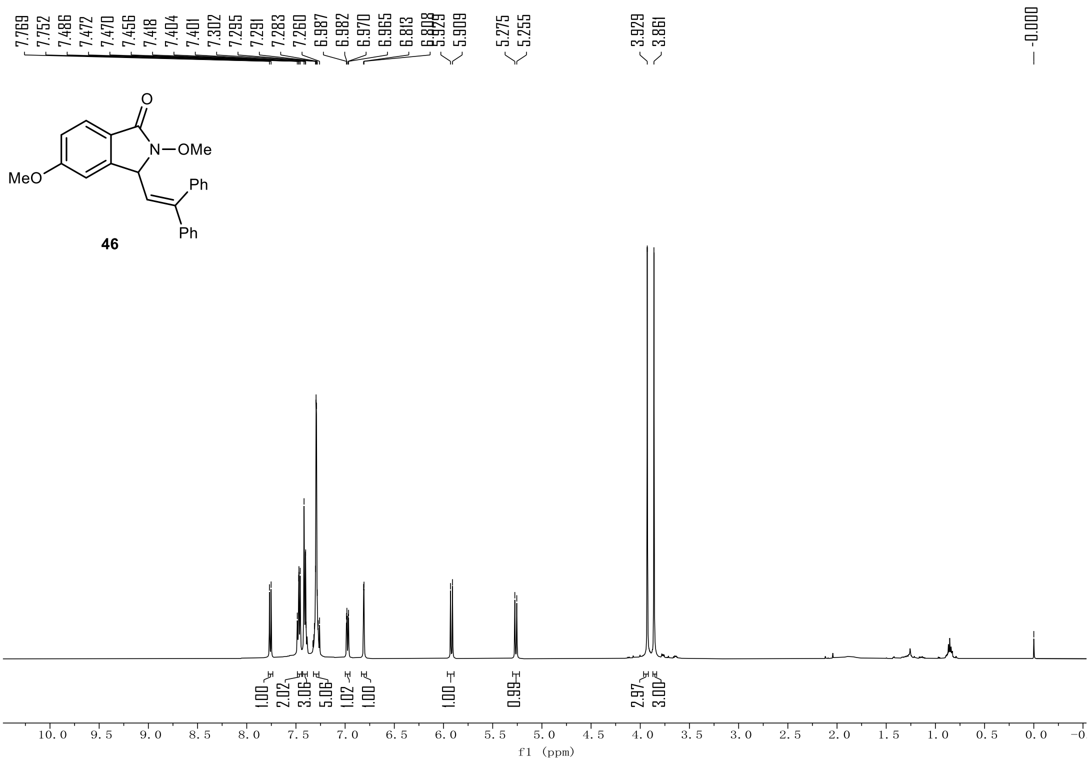
7.769
7.752
7.486
7.472
7.470
7.456
7.418
7.404
7.401
7.302
7.295
7.291
7.283
7.260
6.987
6.982
6.970
6.965
6.813
5.929
5.909
5.275
5.255
3.929
3.861
-0.000
O
N−OMe
MeO
Ph
Ph
46
1.00
2.02
3.06
5.06
1.02
1.00
1.00
0.99
2.97
3.00
f1 (ppm)

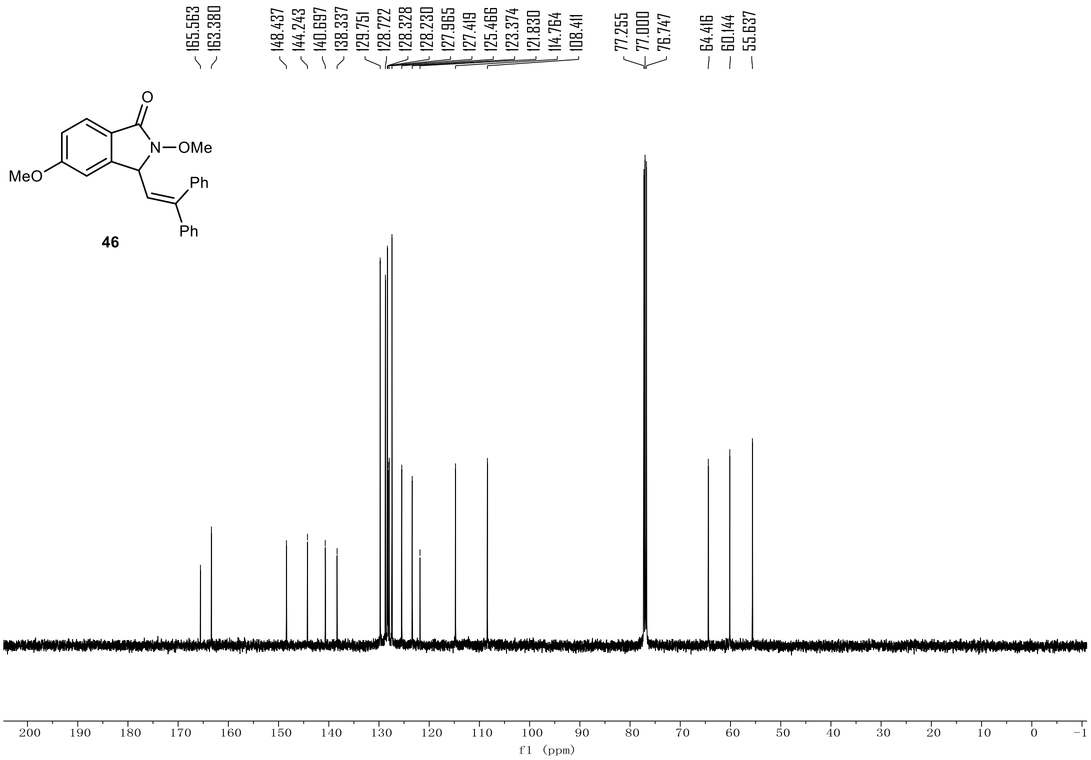
165.563
163.380
148.437
144.243
140.697
138.337
129.751
128.722
128.328
128.230
127.965
127.419
125.466
123.374
121.830
114.764
108.411
77.255
77.000
76.747
64.416
60.144
55.637
f1 (ppm)
MeO
N−OMe
O
Ph
Ph

46

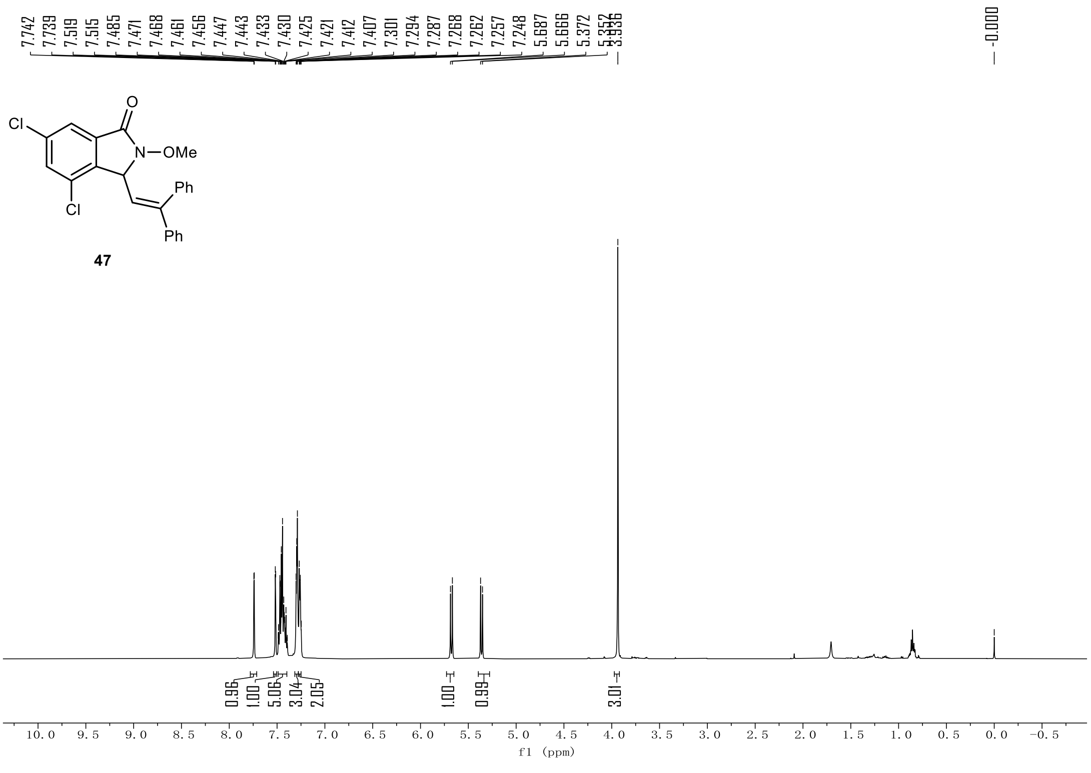
7.742
7.739
7.519
7.515
7.485
7.471
7.468
7.461
7.456
7.447
7.443
7.433
7.430
7.425
7.421
7.412
7.407
7.301
7.294
7.287
7.268
7.262
7.257
7.248
5.687
5.666
5.372
3.936
-0.000
0.96
1.00
5.06
3.04
2.05
1.00
0.99
3.01
f1 (ppm)
Cl
Cl
O
N−OMe
Ph
Ph

47

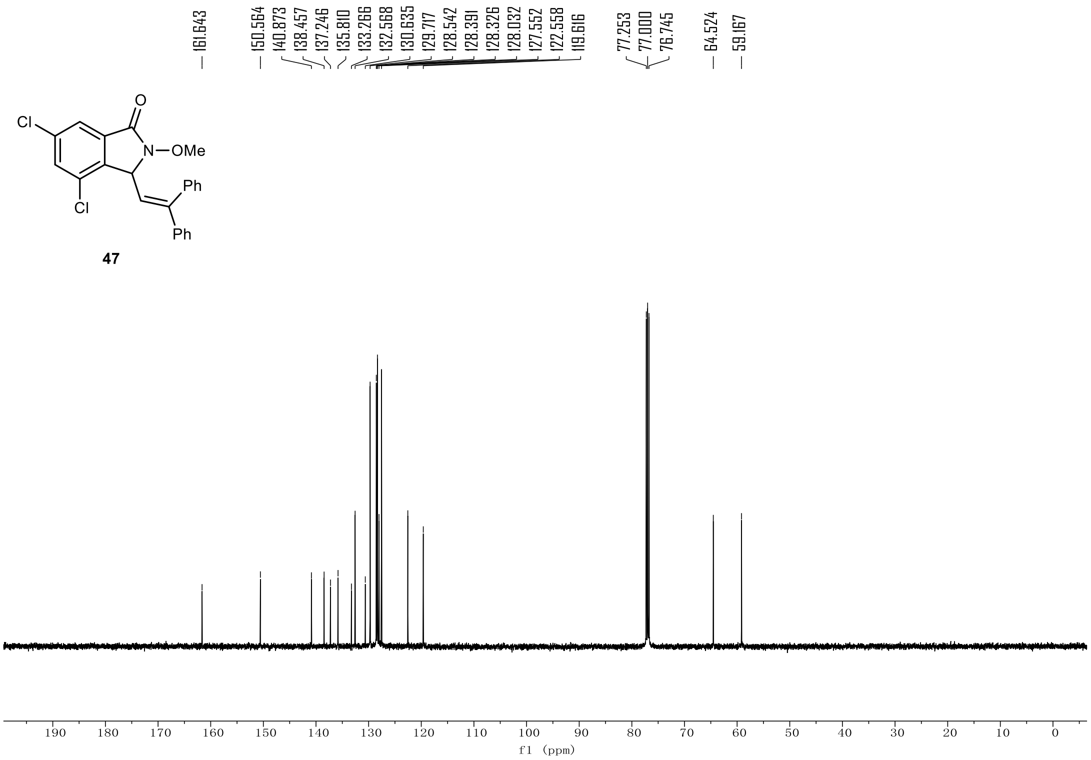
161.643
150.564
140.873
138.457
137.246
135.810
133.266
132.568
130.635
129.717
128.542
128.391
128.326
128.032
127.552
122.558
119.616
77.253
77.000
76.745
64.524
59.167
Cl
O
N−OMe
Ph
Cl
Ph
190 180 170 160 150 140 130 120 110 100 90 80 70 60 50 40 30 20 10 0
f1 (ppm)

47

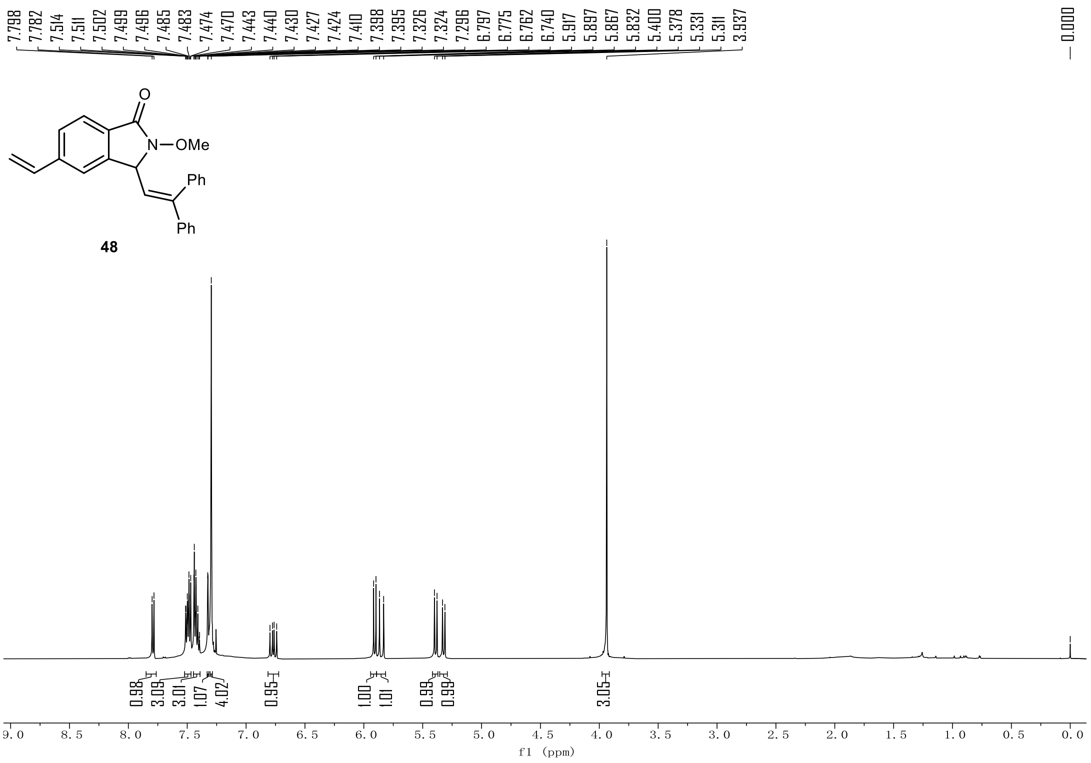
7.798
7.782
7.514
7.511
7.502
7.499
7.496
7.485
7.483
7.474
7.470
7.443
7.440
7.430
7.427
7.424
7.410
7.398
7.395
7.326
7.324
7.296
6.797
6.775
6.762
6.740
5.917
5.897
5.867
5.832
5.400
5.378
5.331
5.311
3.937
0.000
O
N−OMe
Ph
Ph
0.98
3.09
3.01
1.07
4.02
0.95
1.00
1.01
0.99
0.99
3.05
9.0 8.5 8.0 7.5 7.0 6.5 6.0 5.5 5.0 4.5 4.0 3.5 3.0 2.5 2.0 1.5 1.0 0.5 0.0
f1 (ppm)

48

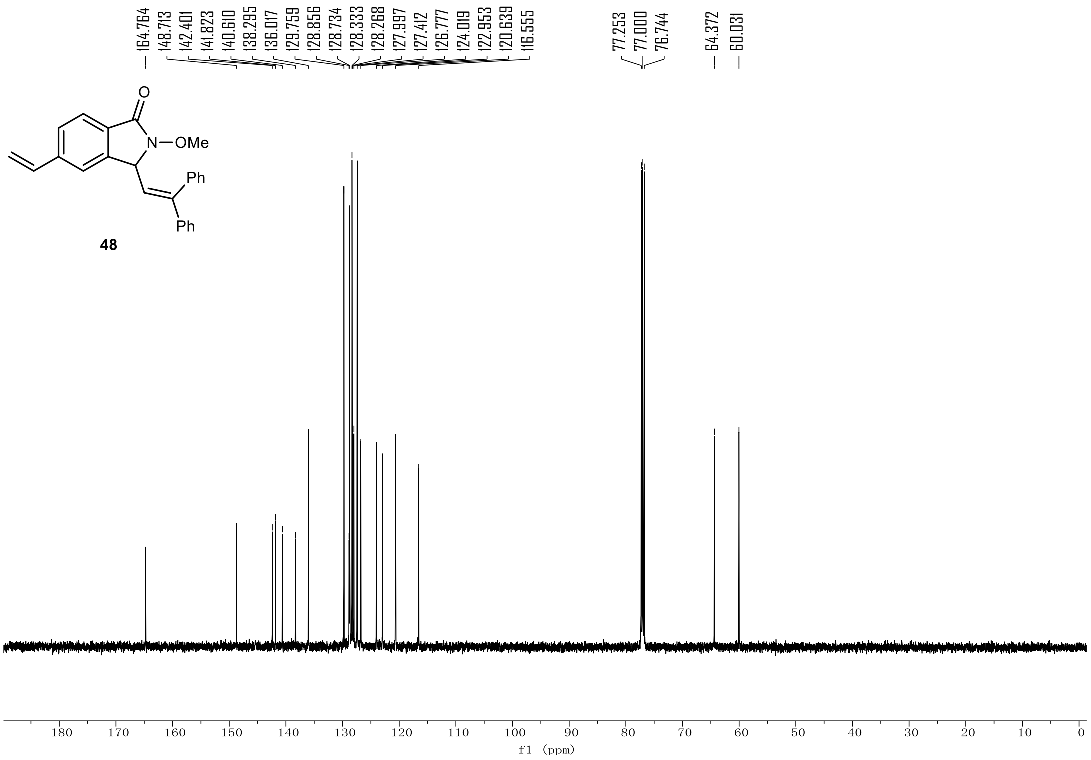
164.764
148.713
142.401
141.823
140.610
138.295
136.017
129.759
128.856
128.734
128.333
128.268
127.997
127.412
126.777
124.019
122.953
120.639
116.555
77.253
77.000
76.744
64.372
60.031
O
N
OMe
Ph
Ph
48
f1 (ppm)

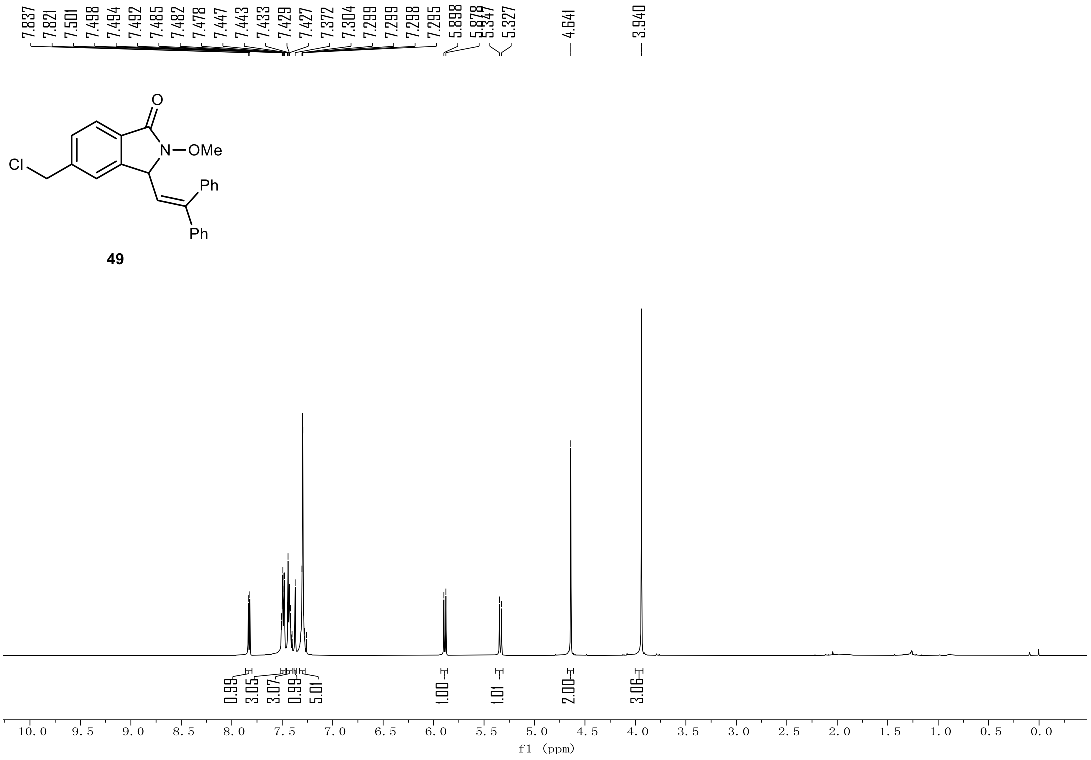
7.837
7.821
7.501
7.498
7.494
7.492
7.485
7.482
7.478
7.447
7.443
7.433
7.429
7.427
7.372
7.304
7.299
7.299
7.298
7.295
5.898
5.878
5.347
5.327
4.641
3.940
O
N
OMe
Cl
Ph
Ph
49
0.99
3.05
3.07
0.99
5.01
1.00
1.01
2.00
3.06
f1 (ppm)

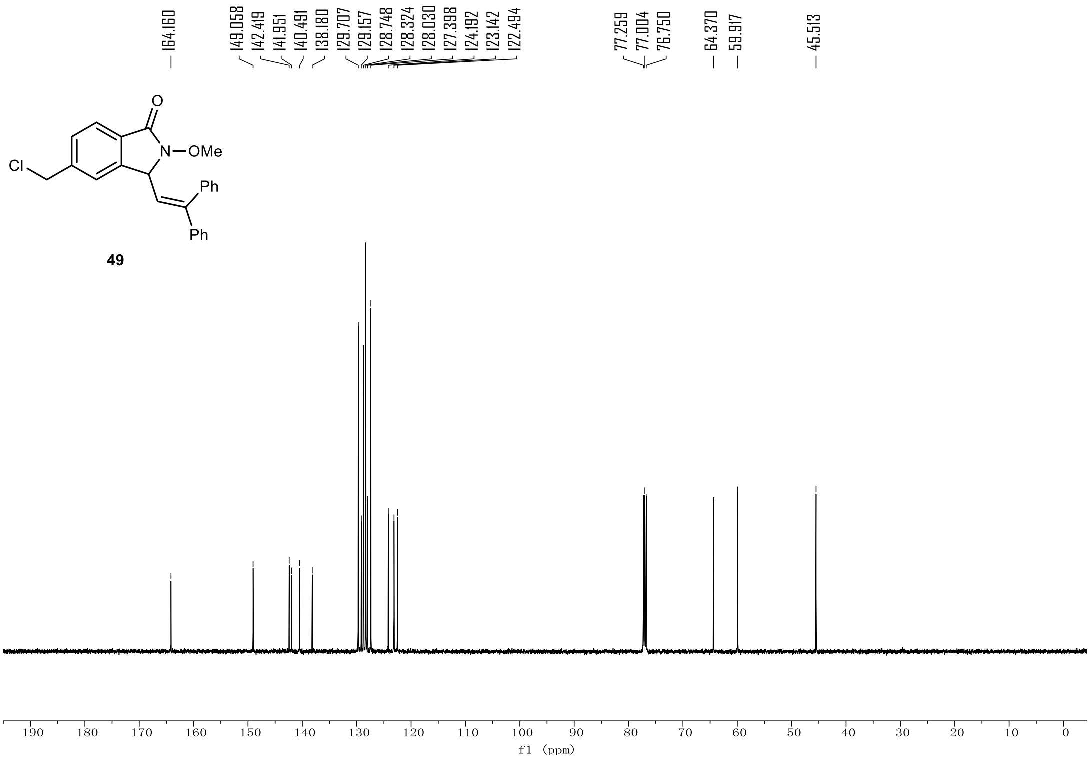

164.160
149.058
142.419
141.951
140.491
138.180
129.707
129.157
128.748
128.324
128.030
127.398
124.192
123.142
122.494
77.259
77.004
76.750
64.370
59.917
45.513
Cl
O
N−OMe
Ph
Ph
f1 (ppm)

49

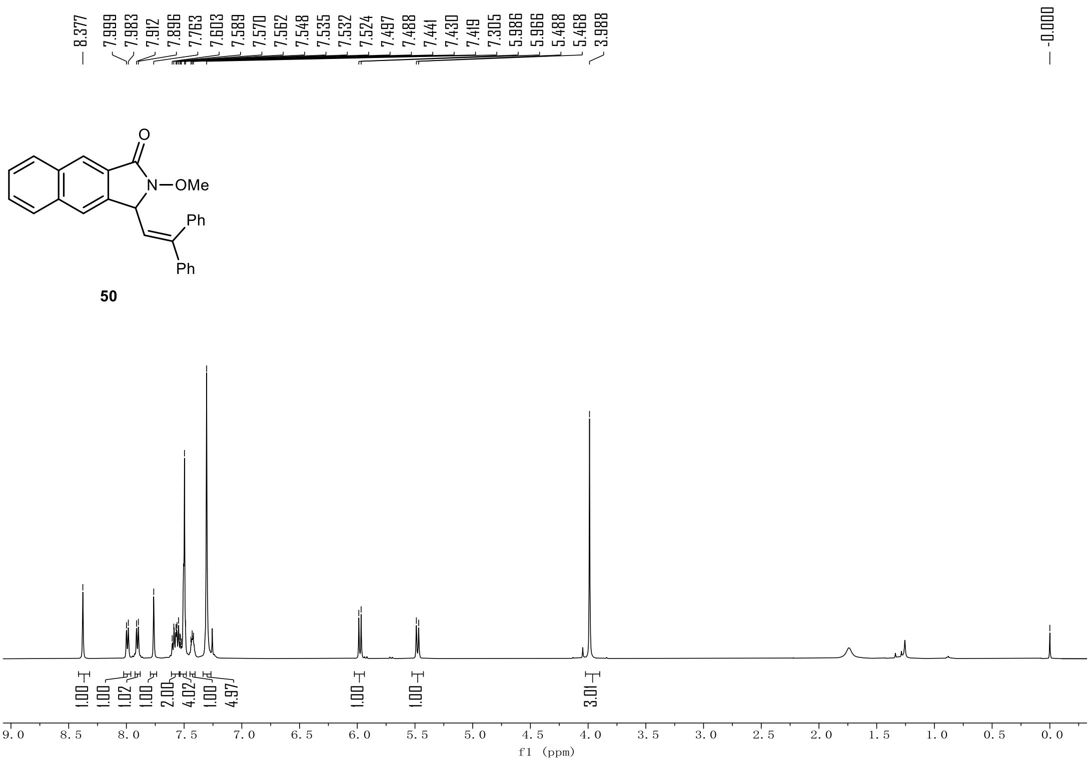

8.377
7.999
7.983
7.912
7.896
7.763
7.603
7.589
7.570
7.562
7.548
7.535
7.532
7.524
7.497
7.488
7.441
7.430
7.419
7.305
5.986
5.966
5.488
5.468
3.988
-0.000
O
N−OMe
Ph
Ph
1.00
1.00
1.02
1.00
2.00
4.02
1.00
4.97
1.00
1.00
3.01
f1 (ppm)

50

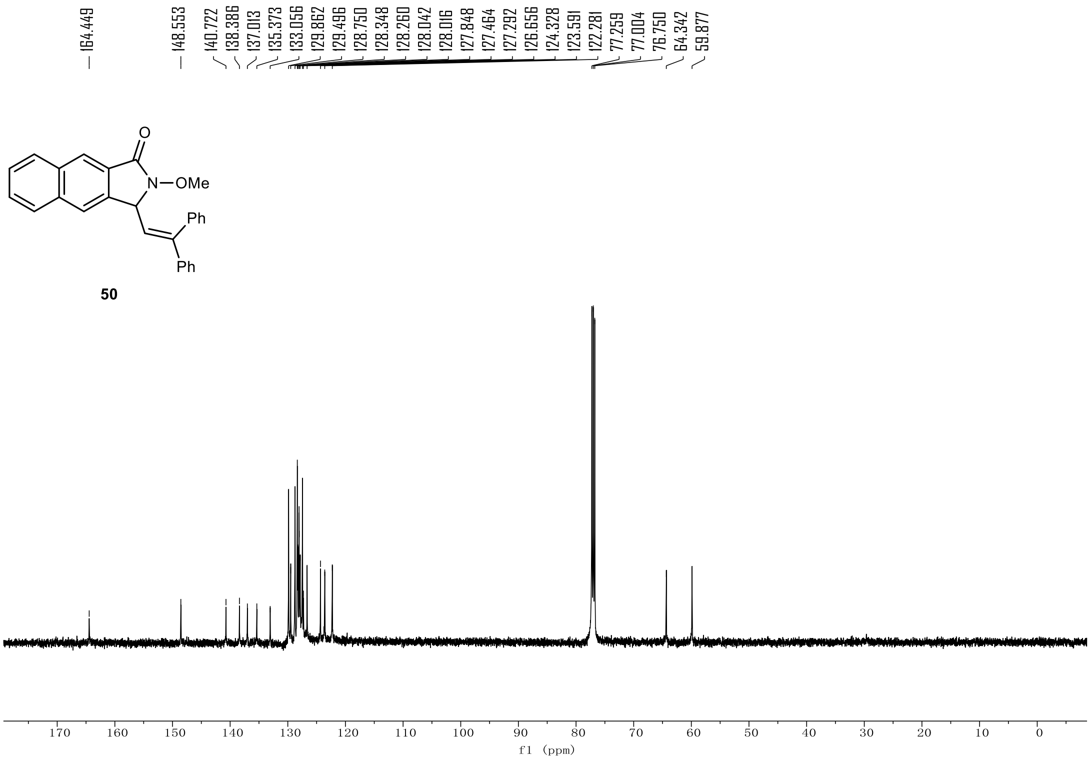
164.449
148.553
140.722
138.386
137.013
135.373
133.056
129.862
129.496
128.750
128.348
128.260
128.042
128.016
127.848
127.464
127.292
126.656
124.328
123.591
122.281
77.259
77.004
76.750
64.342
59.877
O
N–OMe
Ph
Ph
170 160 150 140 130 120 110 100 90 80 70 60 50 40 30 20 10 0
f1 (ppm)

50

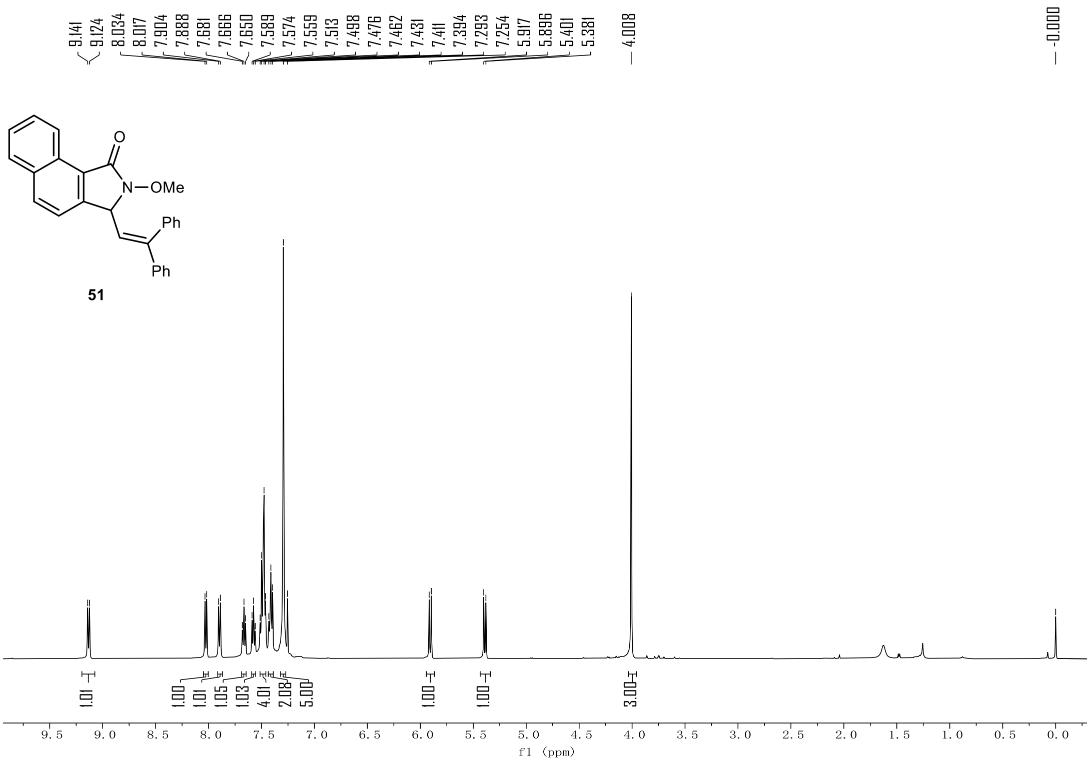
9.141
9.124
8.034
8.017
7.904
7.888
7.681
7.666
7.650
7.589
7.574
7.559
7.513
7.498
7.476
7.462
7.431
7.411
7.394
7.293
7.254
5.917
5.896
5.401
5.381
4.008
0.000
O
N–OMe
Ph
Ph
1.01
1.00
1.01
1.05
1.03
4.01
2.08
5.00
1.00
1.00
3.00
9.5 9.0 8.5 8.0 7.5 7.0 6.5 6.0 5.5 5.0 4.5 4.0 3.5 3.0 2.5 2.0 1.5 1.0 0.5 0.0
f1 (ppm)

51

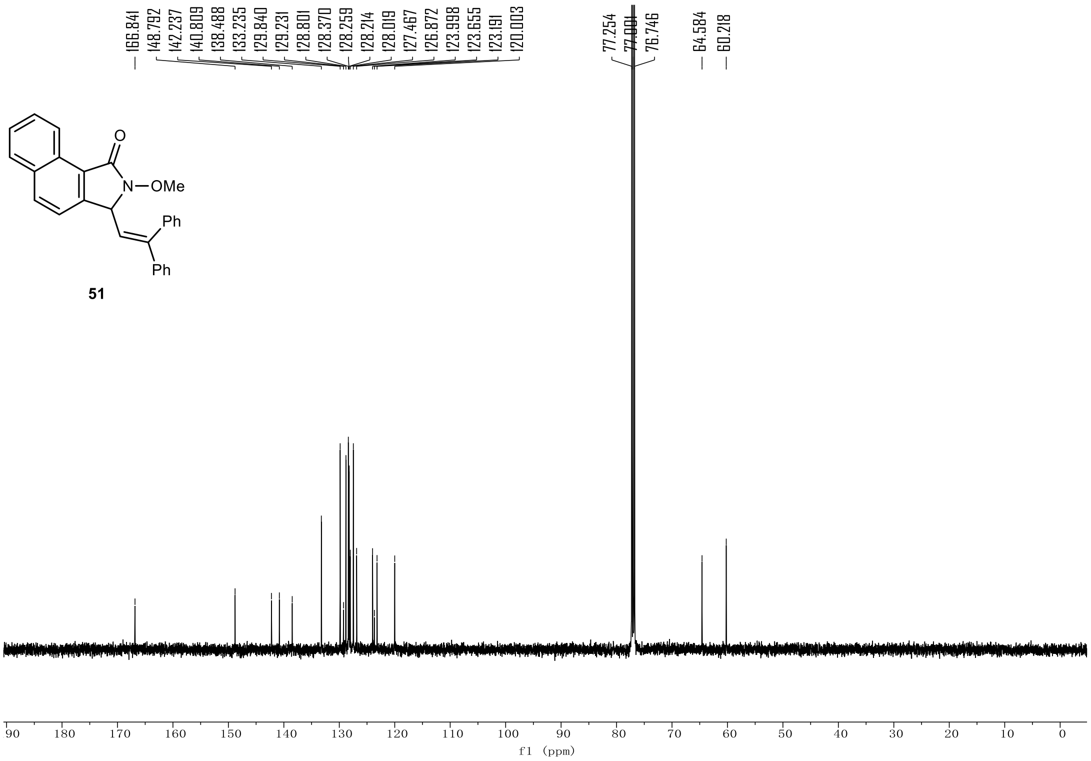
166.841
148.792
142.237
140.809
138.488
133.235
129.840
129.231
128.801
128.370
128.259
128.214
128.019
127.467
126.872
123.998
123.655
123.191
120.003
77.254
77.001
76.746
64.584
60.218
O
N—OMe
Ph
Ph
51
f1 (ppm)

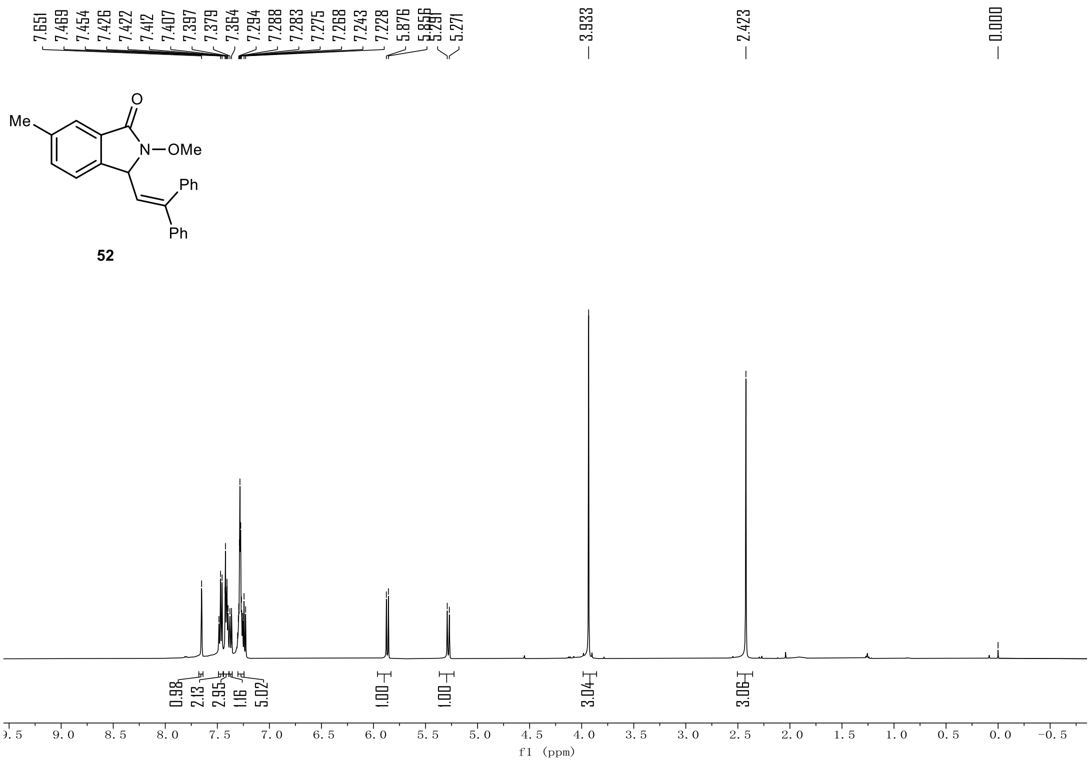
7.651
7.469
7.454
7.426
7.422
7.412
7.407
7.397
7.379
7.364
7.294
7.288
7.283
7.275
7.268
7.243
7.228
5.876
5.856
5.291
5.271
3.933
2.423
0.000
Me
O
N—OMe
Ph
Ph
52
0.98
2.13
2.95
1.16
5.02
1.00
1.00
3.04
3.06
f1 (ppm)

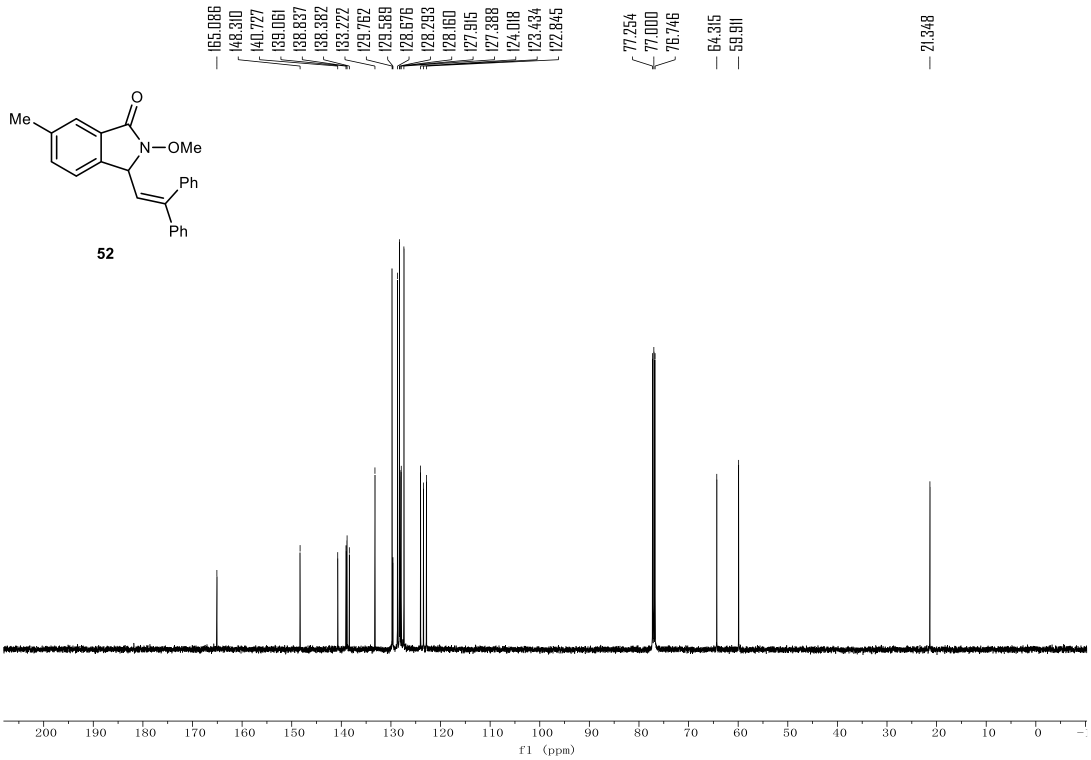
165.086
148.310
140.727
139.061
138.837
138.382
133.222
129.762
129.589
128.676
128.293
128.160
127.915
127.388
124.018
123.434
122.845
77.254
77.000
76.746
64.315
59.911
21.348
Me
O
N−OMe
Ph
Ph
52
200 190 180 170 160 150 140 130 120 110 100 90 80 70 60 50 40 30 20 10 0
f1 (ppm)

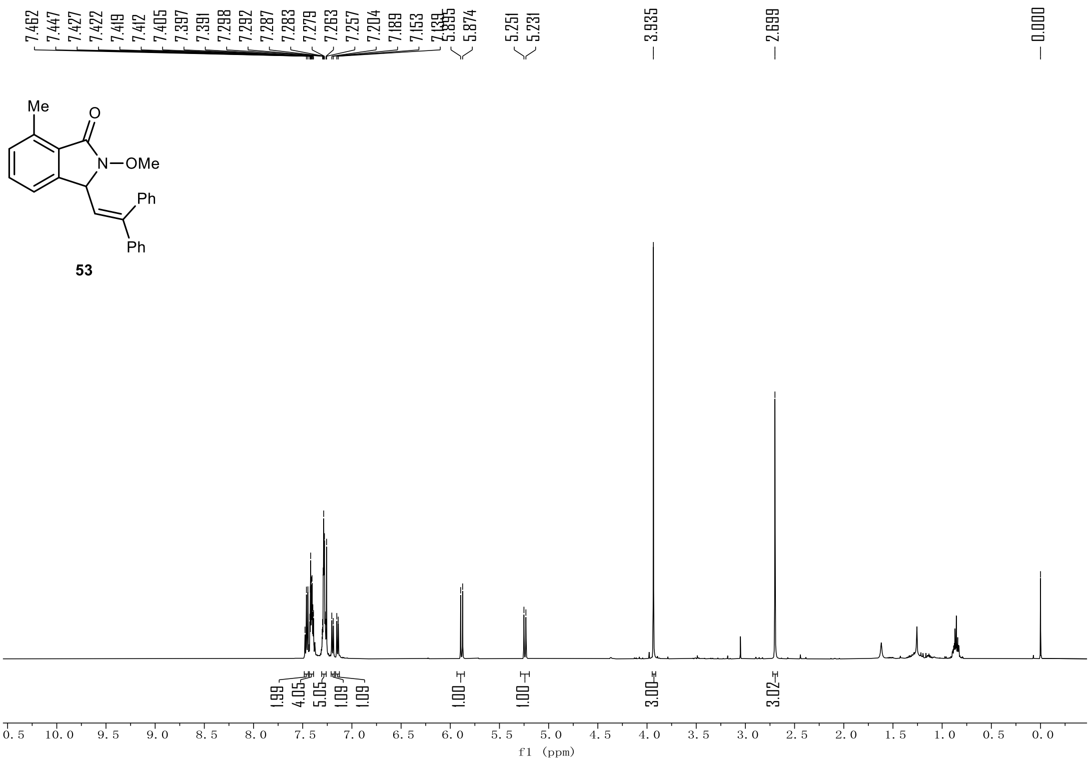
7.462
7.447
7.427
7.422
7.419
7.412
7.405
7.397
7.391
7.298
7.292
7.287
7.283
7.279
7.263
7.257
7.204
7.189
7.153
5.874
5.251
5.231
3.935
2.699
0.000
Me
O
N−OMe
Ph
Ph
53
1.99
4.05
5.05
1.09
1.09
1.00
1.00
3.00
3.02
10.0 9.5 9.0 8.5 8.0 7.5 7.0 6.5 6.0 5.5 5.0 4.5 4.0 3.5 3.0 2.5 2.0 1.5 1.0 0.5 0.0
f1 (ppm)

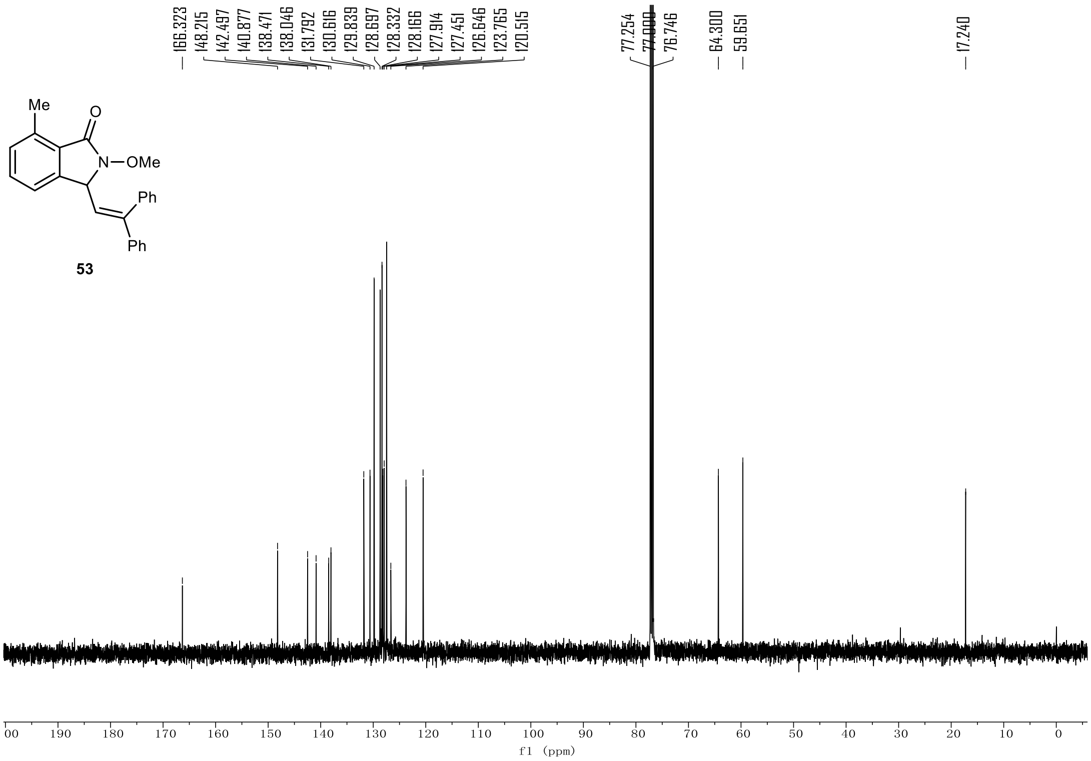
166.323
148.215
142.497
140.877
138.471
138.046
131.792
130.616
129.839
128.697
128.332
128.166
127.914
127.451
126.646
123.765
120.515
77.254
77.000
76.746
64.300
59.651
17.240
Me
O
N−OMe
Ph
Ph
f1 (ppm)

53

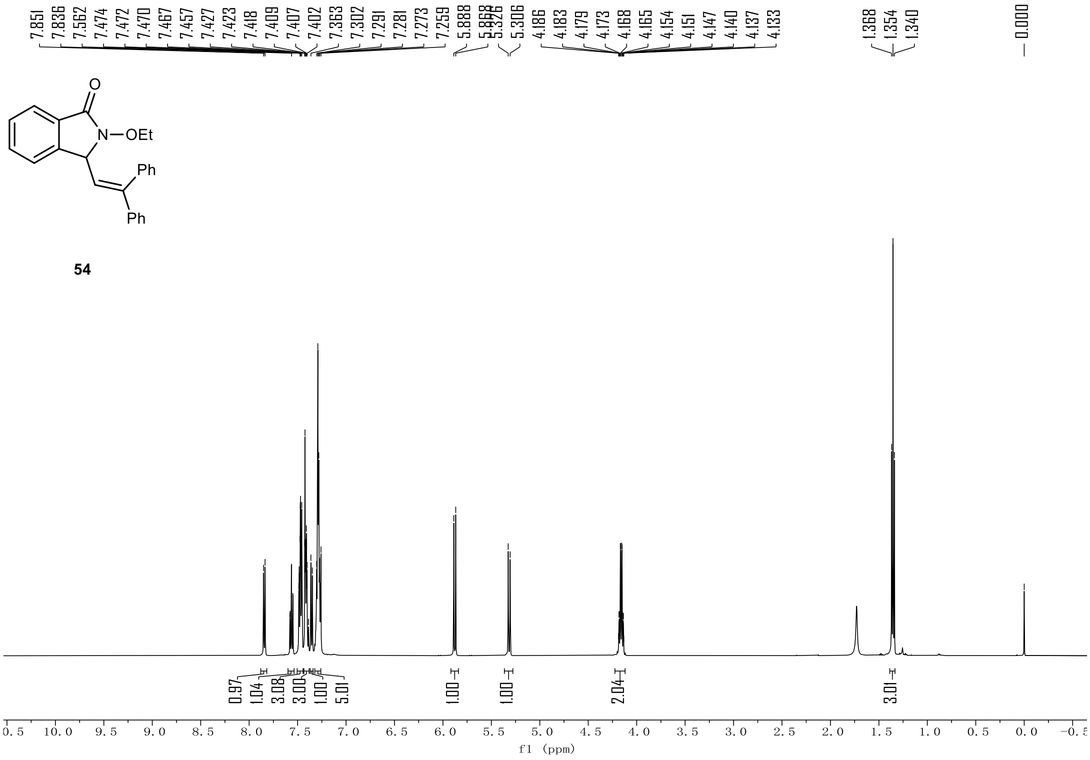
7.851
7.836
7.562
7.474
7.472
7.470
7.467
7.457
7.427
7.423
7.418
7.409
7.407
7.402
7.363
7.302
7.291
7.281
7.273
7.259
5.888
5.868
5.326
5.306
4.186
4.183
4.179
4.173
4.168
4.165
4.154
4.151
4.147
4.140
4.137
4.133
1.368
1.354
1.340
0.000
O
N−OEt
Ph
Ph
0.97
1.04
3.08
3.00
1.00
5.01
1.00
1.00
2.04
3.01
f1 (ppm)

54

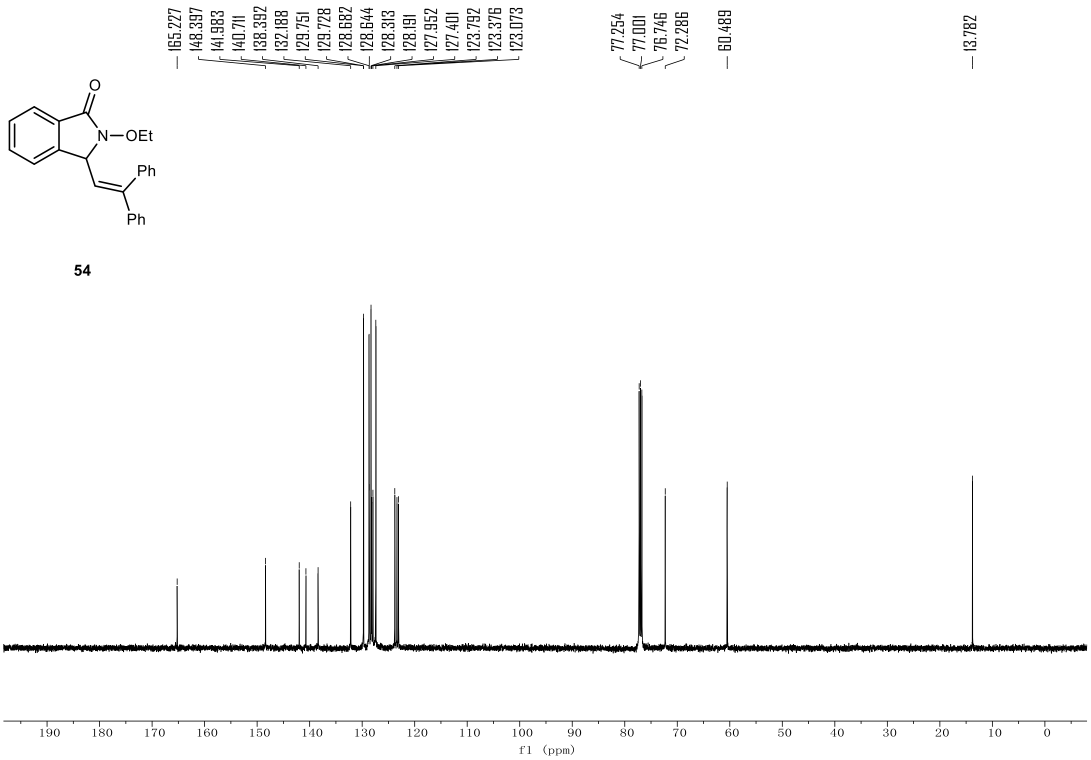
165.227
148.397
141.983
140.711
138.392
132.188
129.751
129.728
128.682
128.644
128.313
128.191
127.952
127.401
123.792
123.376
123.073
77.254
77.001
76.746
72.286
60.489
13.782
N−OEt
Ph
Ph
54
f1 (ppm)

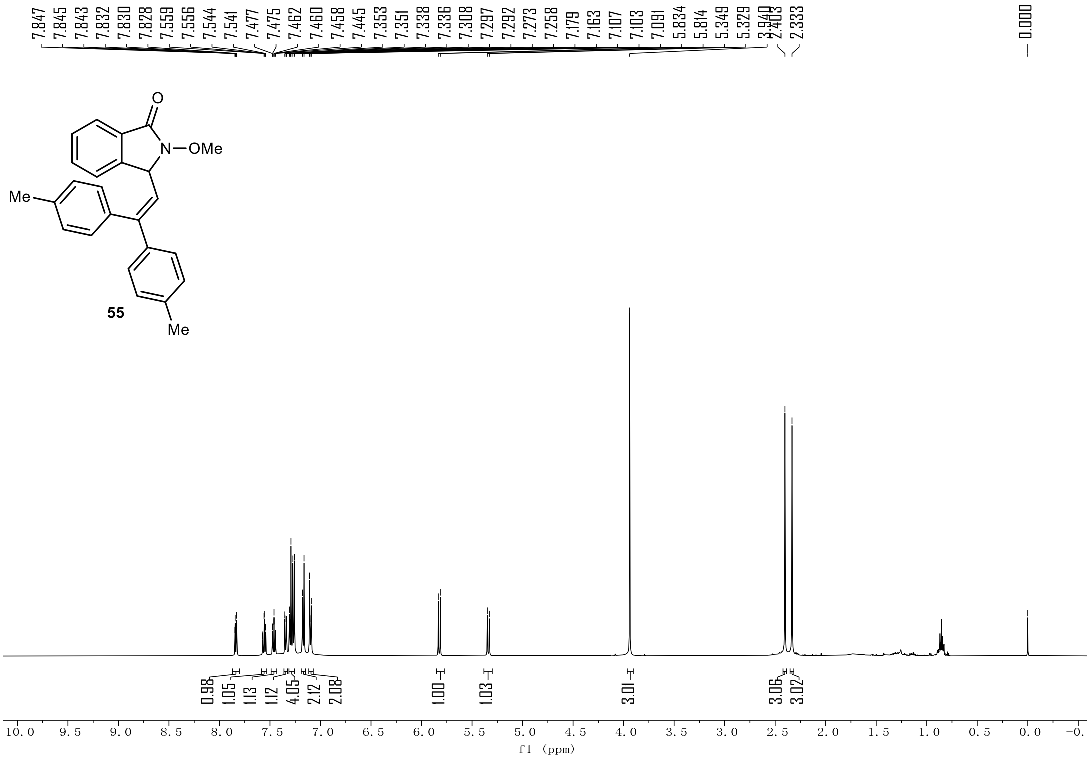
7.847
7.845
7.843
7.832
7.830
7.828
7.559
7.556
7.544
7.541
7.477
7.475
7.462
7.460
7.458
7.445
7.353
7.351
7.338
7.336
7.308
7.297
7.292
7.273
7.258
7.179
7.163
7.107
7.103
7.091
5.834
5.814
5.349
5.329
3.940
2.403
2.333
0.000
N−OMe
Me
Me
55
f1 (ppm)

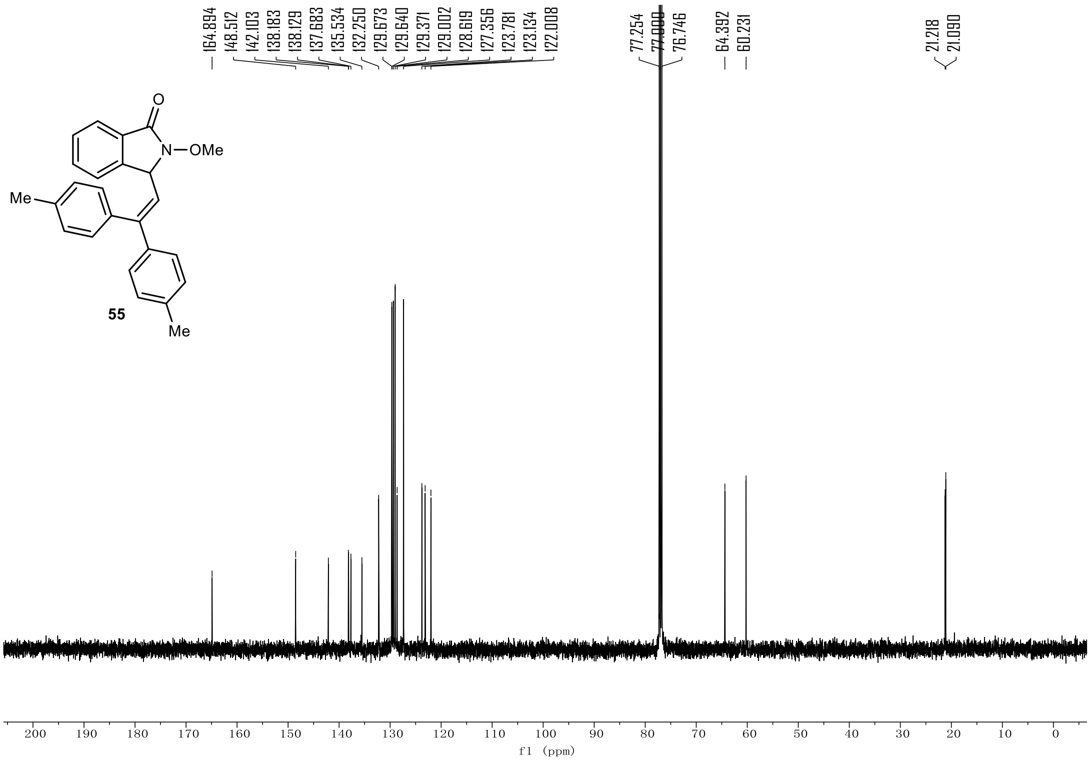
164.894
148.512
142.103
138.183
138.129
137.683
135.534
132.250
129.673
129.640
129.371
129.002
128.619
127.356
123.781
123.134
122.008
77.254
77.000
76.746
64.392
60.231
21.218
21.090
O
N
OMe
Me
Me
f1 (ppm)

55

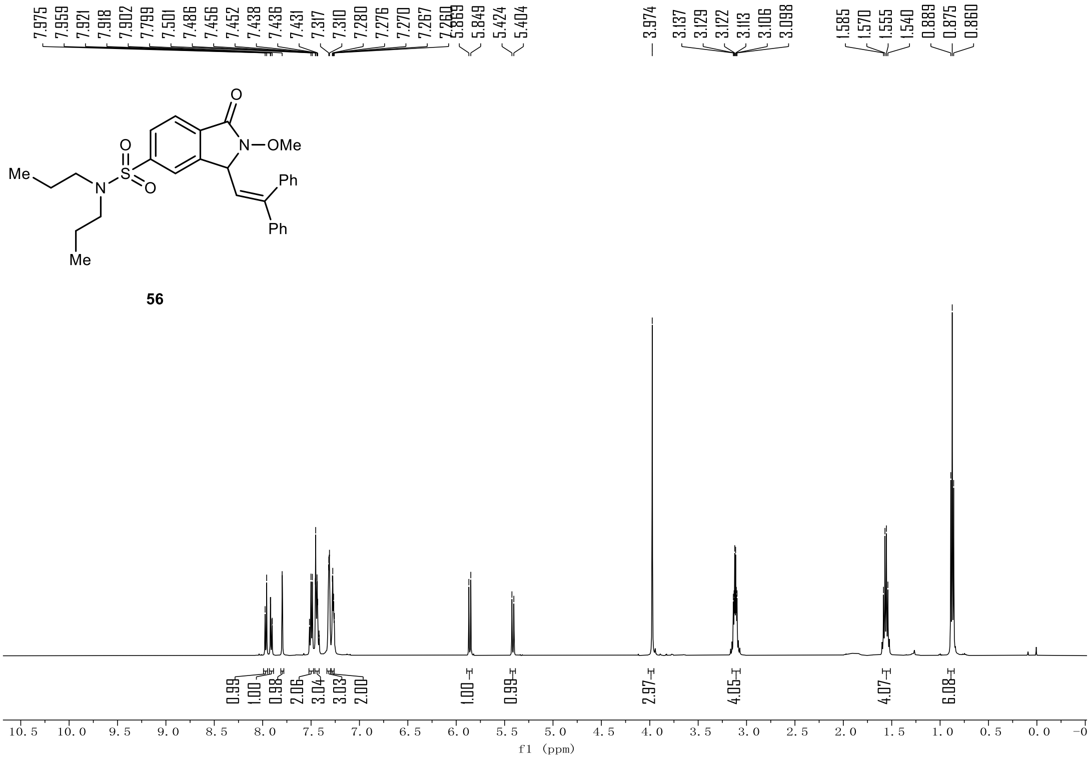
7.975
7.959
7.921
7.918
7.902
7.799
7.501
7.486
7.456
7.452
7.438
7.436
7.431
7.317
7.310
7.280
7.276
7.270
7.267
7.260
5.869
5.849
5.424
5.404
3.974
3.137
3.129
3.122
3.113
3.106
3.098
1.585
1.570
1.555
1.540
0.889
0.875
0.860
O
N
OMe
S
O
O
N
Me
Me
Ph
Ph
0.99
1.00
0.98
2.06
3.04
3.03
2.00
1.00
0.99
2.97
4.05
4.07
6.08
f1 (ppm)

56

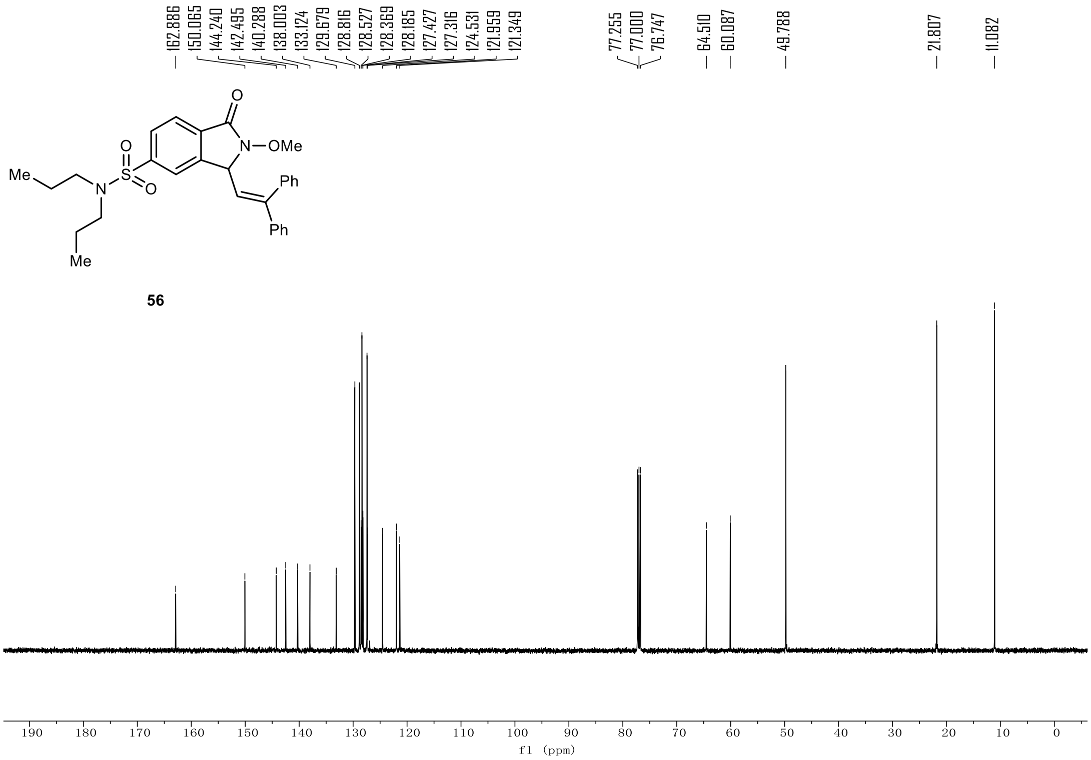
162.886
150.065
144.240
142.495
140.288
138.003
133.124
129.679
128.816
128.527
128.369
128.185
127.427
127.316
124.531
121.959
121.349
77.255
77.000
76.747
64.510
60.087
49.788
21.807
11.082
O
N
OMe
O
S
O
Me
N
Ph
Ph
Me
f1 (ppm)

56

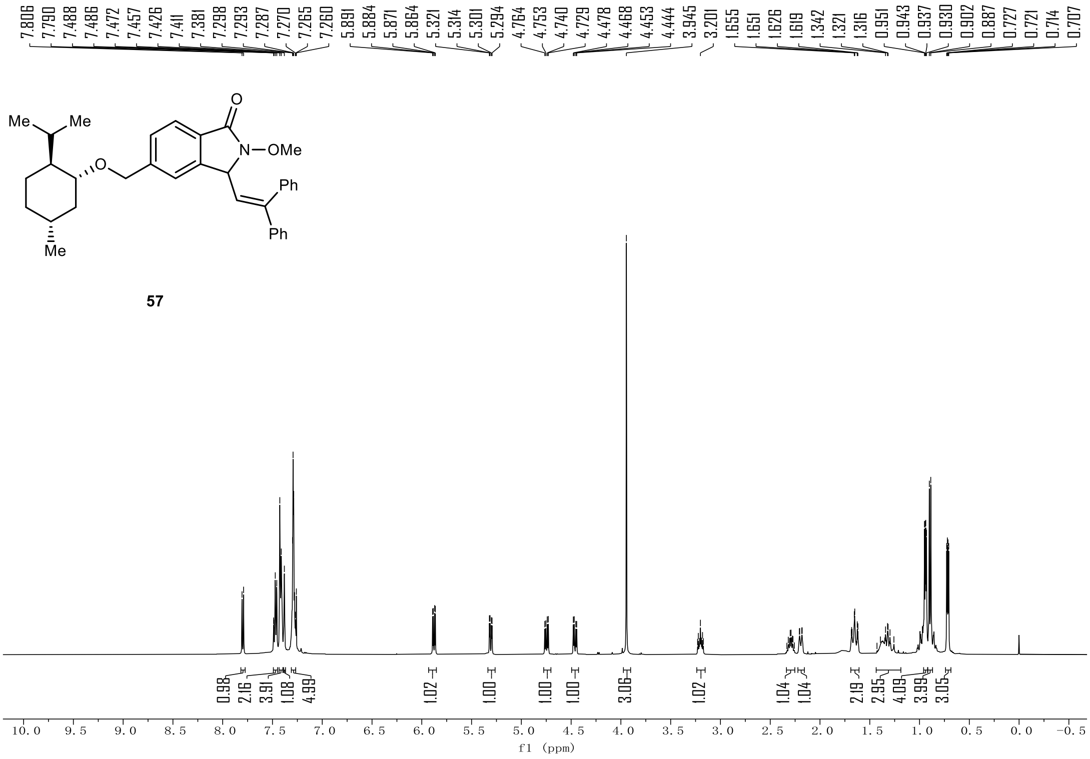
7.806
7.790
7.488
7.486
7.472
7.457
7.426
7.411
7.381
7.298
7.293
7.287
7.270
7.265
7.260
5.891
5.884
5.871
5.864
5.321
5.314
5.301
5.294
4.764
4.753
4.740
4.729
4.478
4.468
4.453
4.444
3.945
3.201
1.655
1.651
1.626
1.619
1.342
1.321
1.316
0.951
0.943
0.937
0.930
0.902
0.887
0.727
0.721
0.714
0.707
Me
Me
O
O
N
OMe
Ph
Ph
Me
0.98
2.16
3.91
1.08
4.99
1.02
1.00
1.00
1.00
3.06
1.02
1.04
1.04
2.19
2.95
4.09
3.99
3.05
f1 (ppm)

57

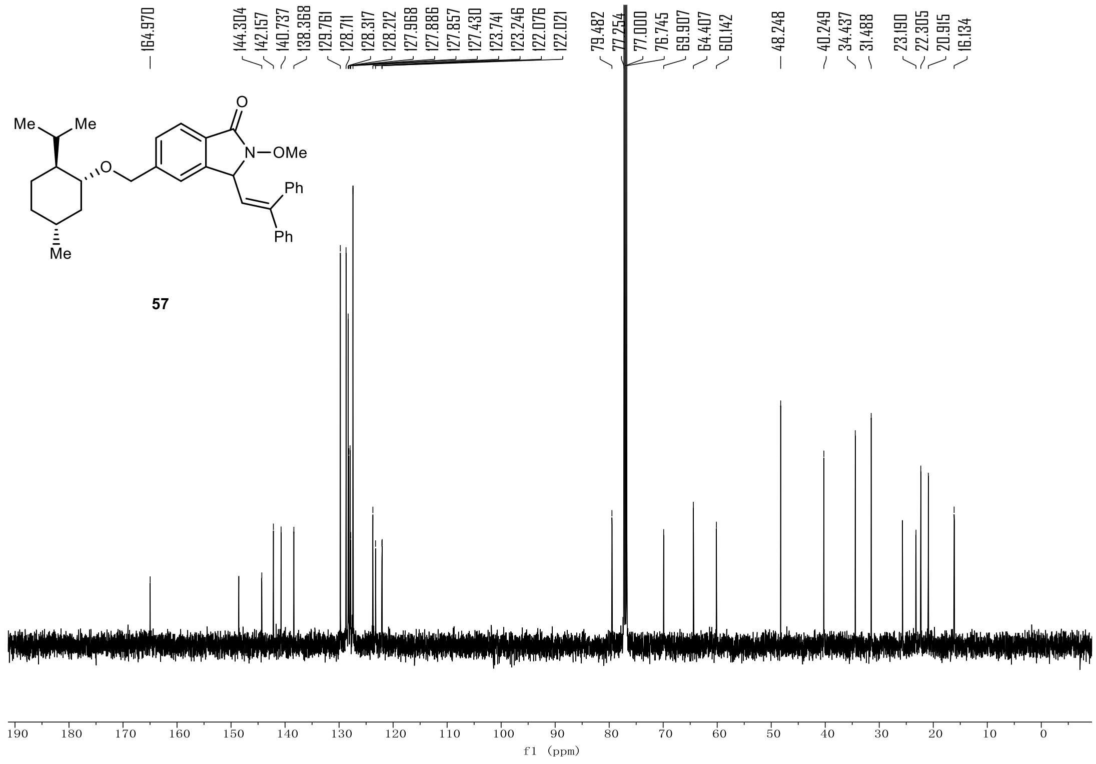

57
f1 (ppm)

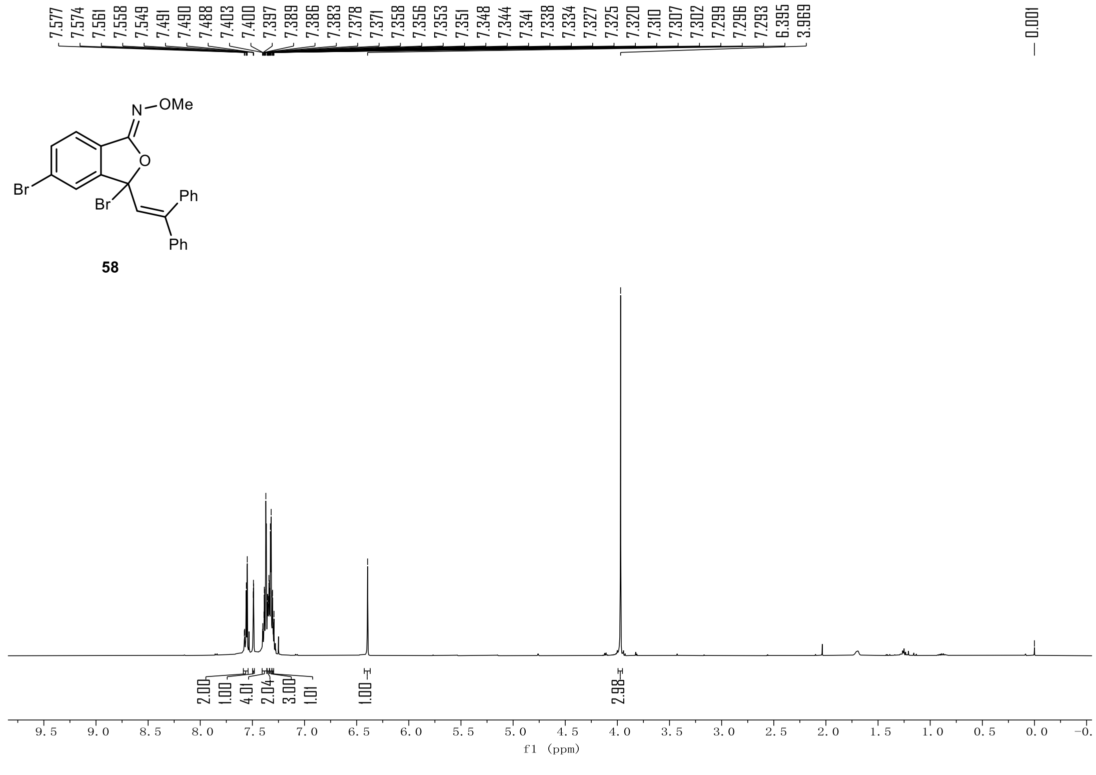

58
f1 (ppm)

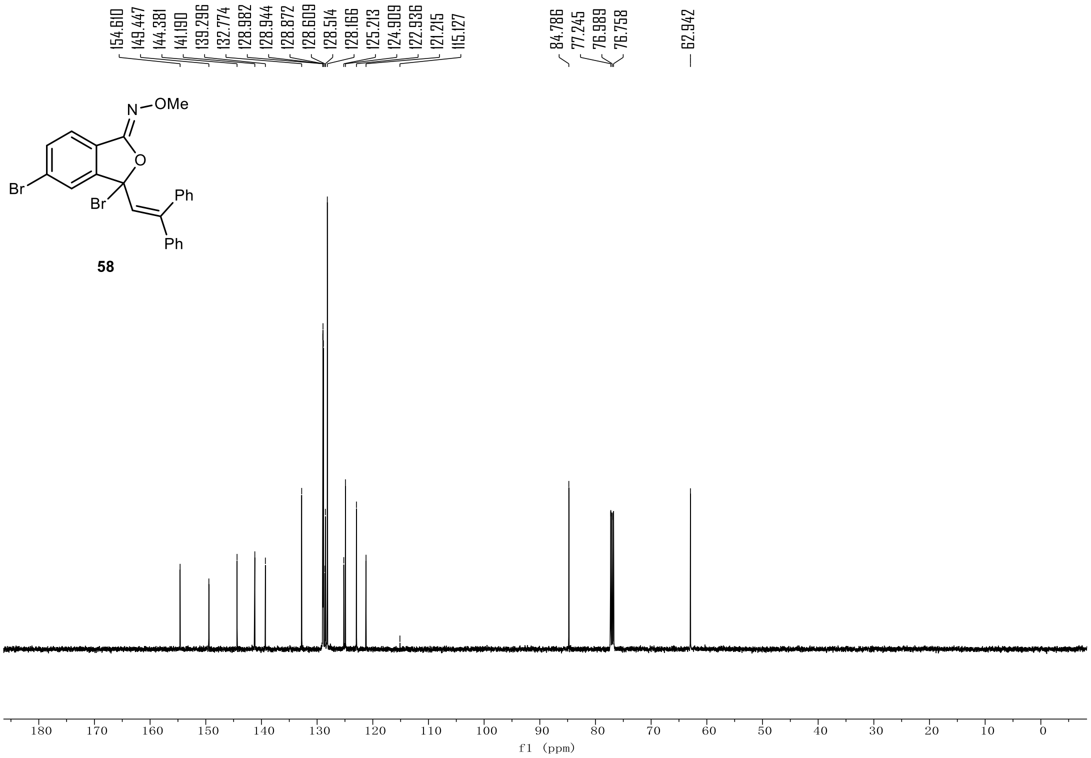
154.610
149.447
144.381
141.190
139.296
132.774
128.982
128.944
128.872
128.609
128.514
128.166
125.213
124.909
122.936
121.215
115.127
84.786
77.245
76.989
76.758
62.942
N
OMe
O
Br
Br
Ph
Ph
58
f1 (ppm)

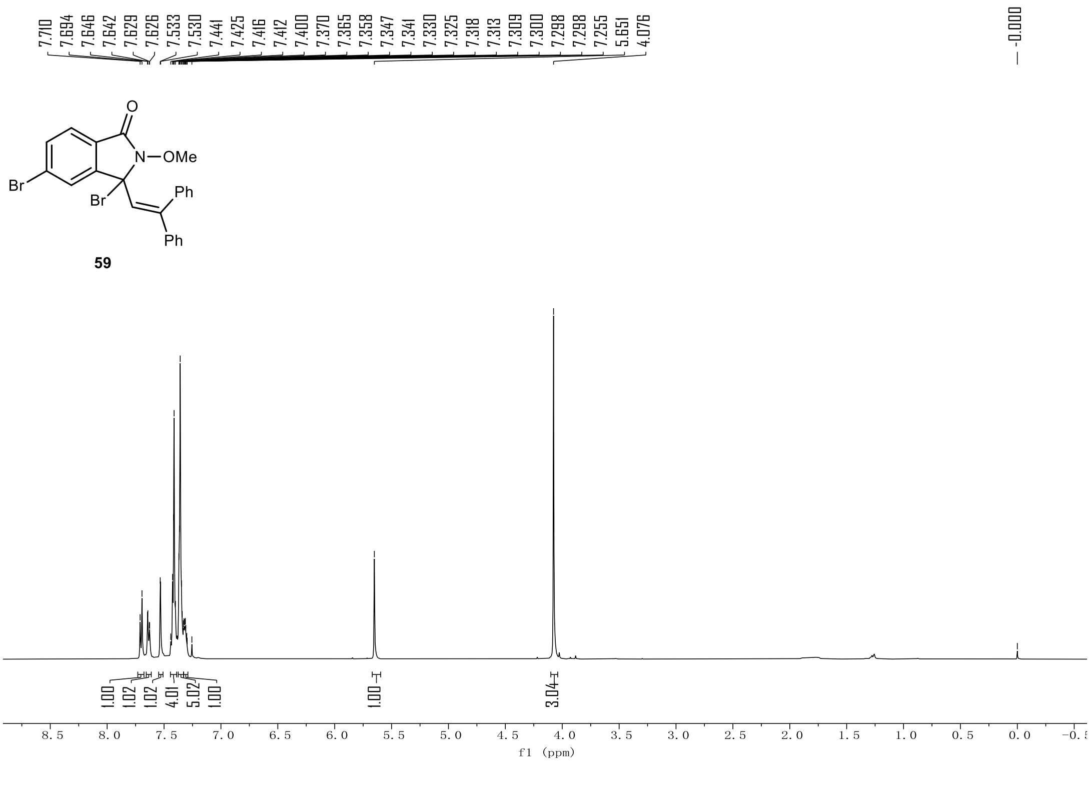
7.710
7.694
7.646
7.642
7.629
7.626
7.533
7.530
7.441
7.425
7.416
7.412
7.400
7.370
7.365
7.358
7.347
7.341
7.330
7.325
7.318
7.313
7.309
7.300
7.298
7.298
7.255
5.651
4.076
-0.000
O
N
OMe
Br
Br
Ph
Ph
59
1.00
1.02
1.02
4.01
5.02
1.00
1.00
3.04
f1 (ppm)

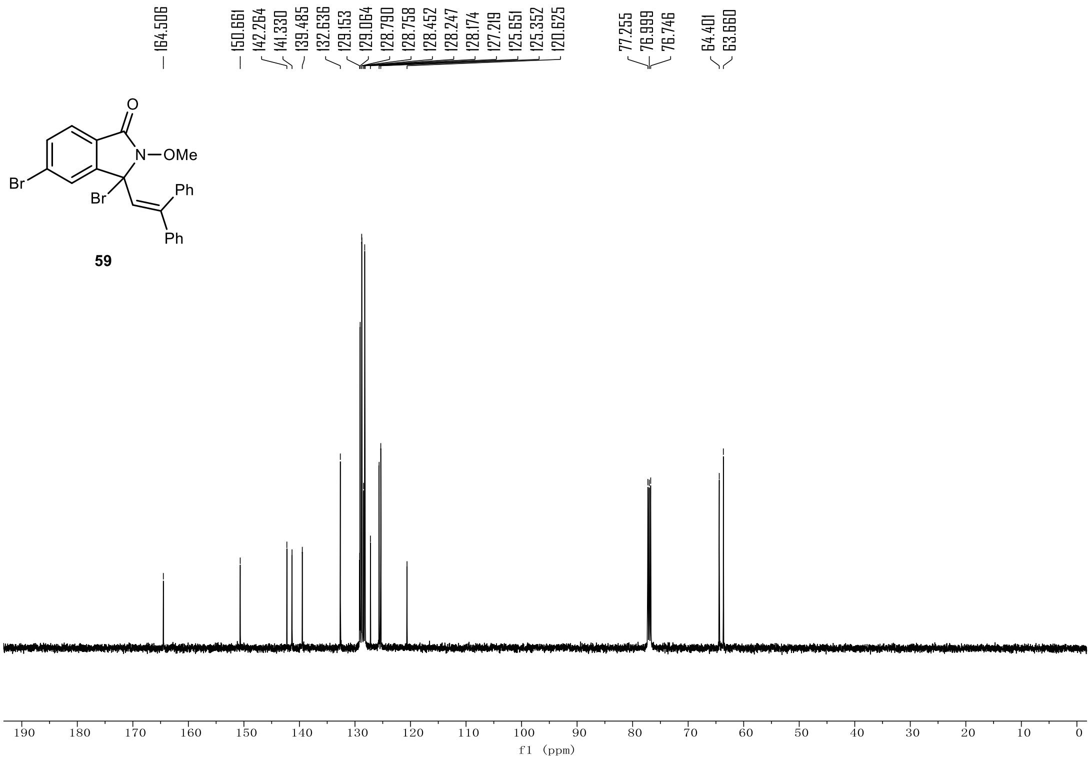

164.506
150.661
142.264
141.330
139.485
132.636
129.153
129.064
128.790
128.758
128.452
128.247
128.174
127.219
125.651
125.352
120.625
77.255
76.999
76.746
64.401
63.660
O
N−OMe
Br
Br
Ph
Ph
59
190 180 170 160 150 140 130 120 110 100 90 80 70 60 50 40 30 20 10 0
f1 (ppm)

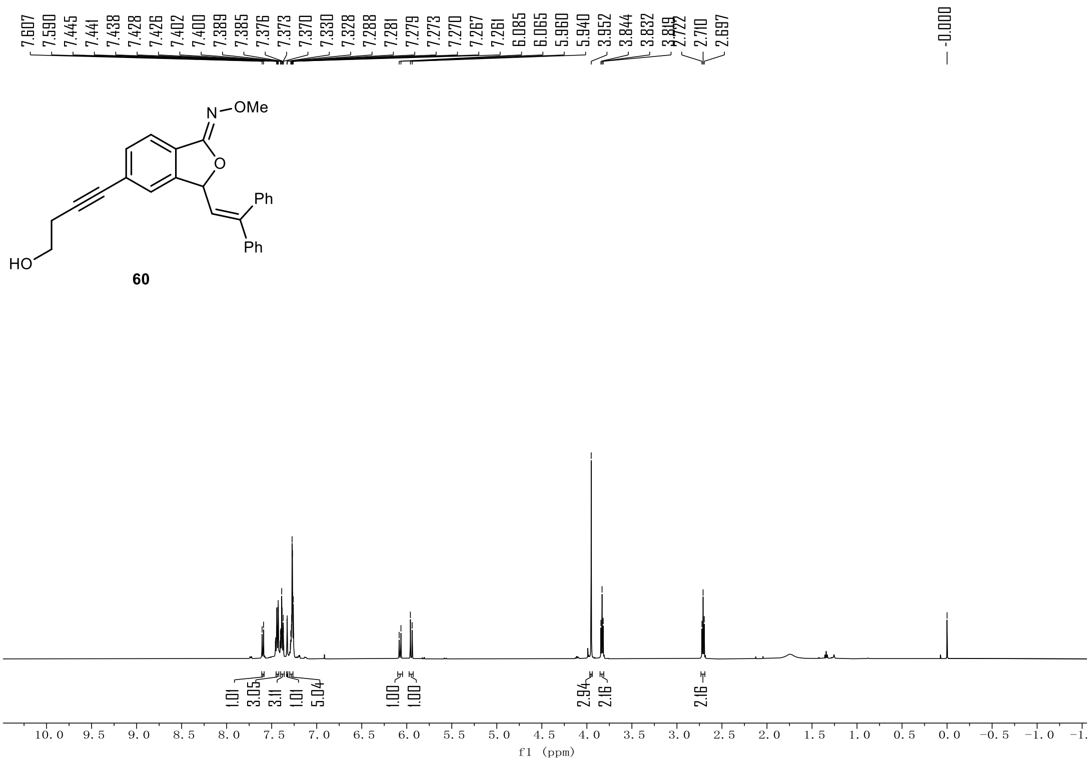

7.607
7.590
7.445
7.441
7.438
7.428
7.426
7.402
7.400
7.389
7.385
7.376
7.373
7.370
7.330
7.328
7.288
7.281
7.279
7.273
7.270
7.267
7.261
6.085
6.065
5.960
5.940
3.952
3.844
3.832
2.722
2.710
2.697
-0.000
N−OMe
O
Ph
Ph
HO
60
1.01
3.05
3.11
1.01
5.04
1.00
1.00
2.94
2.16
2.16
10.0 9.5 9.0 8.5 8.0 7.5 7.0 6.5 6.0 5.5 5.0 4.5 4.0 3.5 3.0 2.5 2.0 1.5 1.0 0.5 0.0 −0.5 −1.0
f1 (ppm)

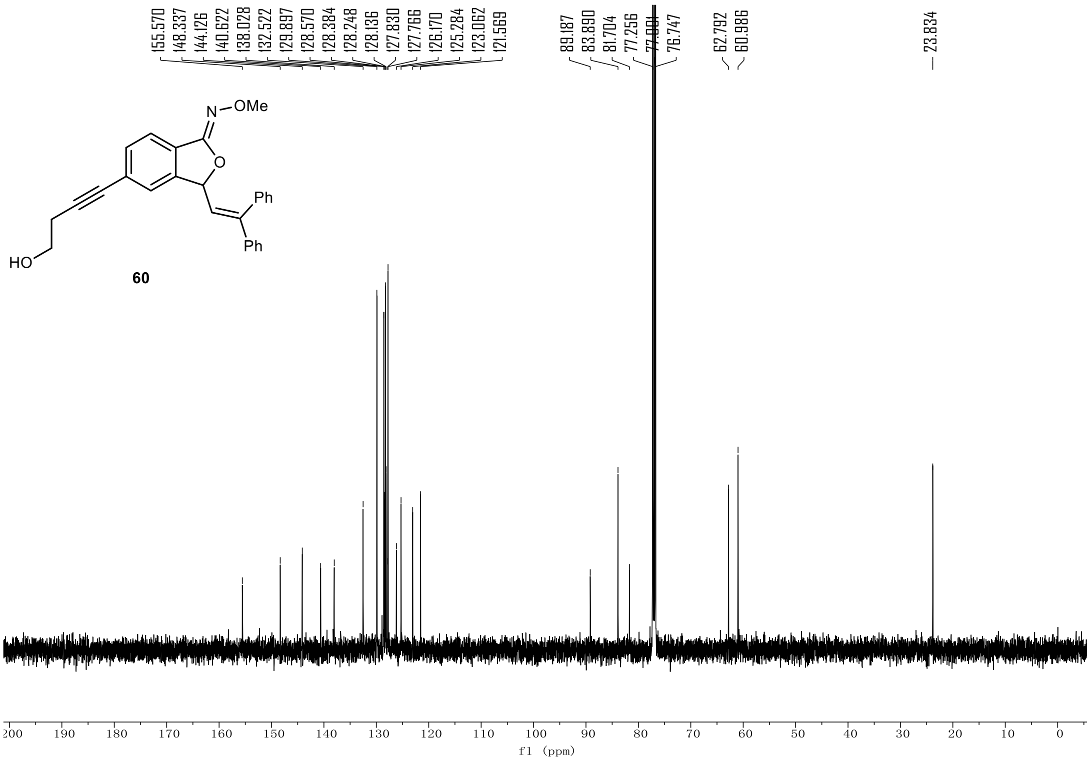
155.570
148.337
144.126
140.622
138.028
132.522
129.897
128.570
128.384
128.248
128.136
127.830
127.766
126.170
125.284
123.062
121.569
89.187
83.890
81.704
77.256
76.747
62.792
60.986
23.834
N–OMe
O
Ph
Ph
HO
f1 (ppm)

60

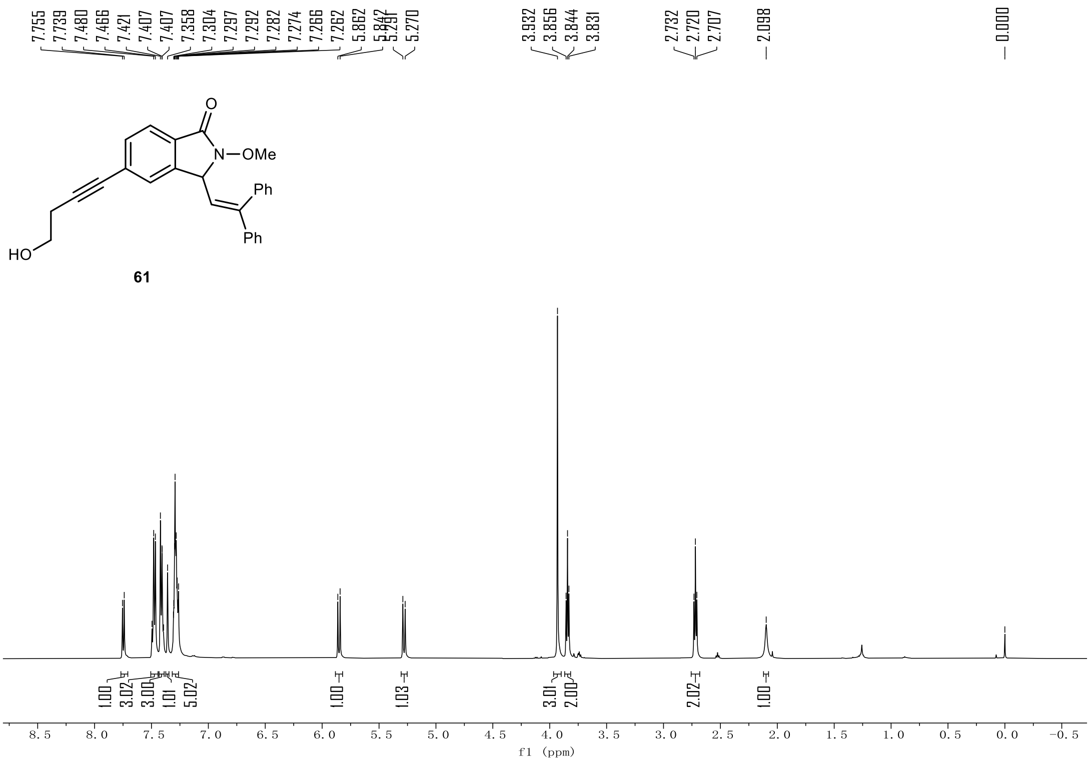
7.755
7.739
7.480
7.466
7.421
7.407
7.407
7.358
7.304
7.297
7.292
7.282
7.274
7.266
7.262
5.862
5.270
3.932
3.856
3.844
3.831
2.732
2.720
2.707
2.098
0.000
O
N–OMe
Ph
Ph
HO
1.00
3.02
3.00
1.01
5.02
1.00
1.03
3.01
2.00
2.02
1.00
f1 (ppm)

61

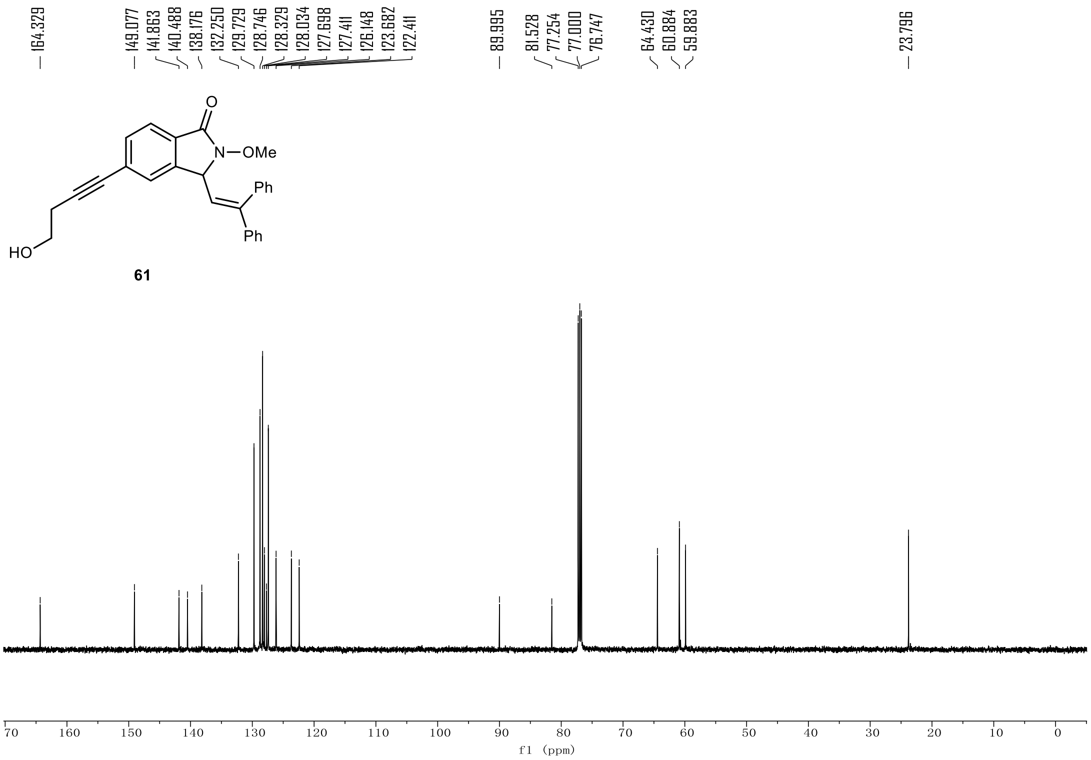
164.329
149.077
141.863
140.488
138.176
132.250
129.729
128.746
128.329
128.034
127.698
127.411
126.148
123.682
122.411
89.995
81.528
77.254
77.000
76.747
64.430
60.884
59.883
23.796
O
N—OMe
Ph
Ph
HO
61
70 160 150 140 130 120 110 100 90 80 70 60 50 40 30 20 10 0
f1 (ppm)

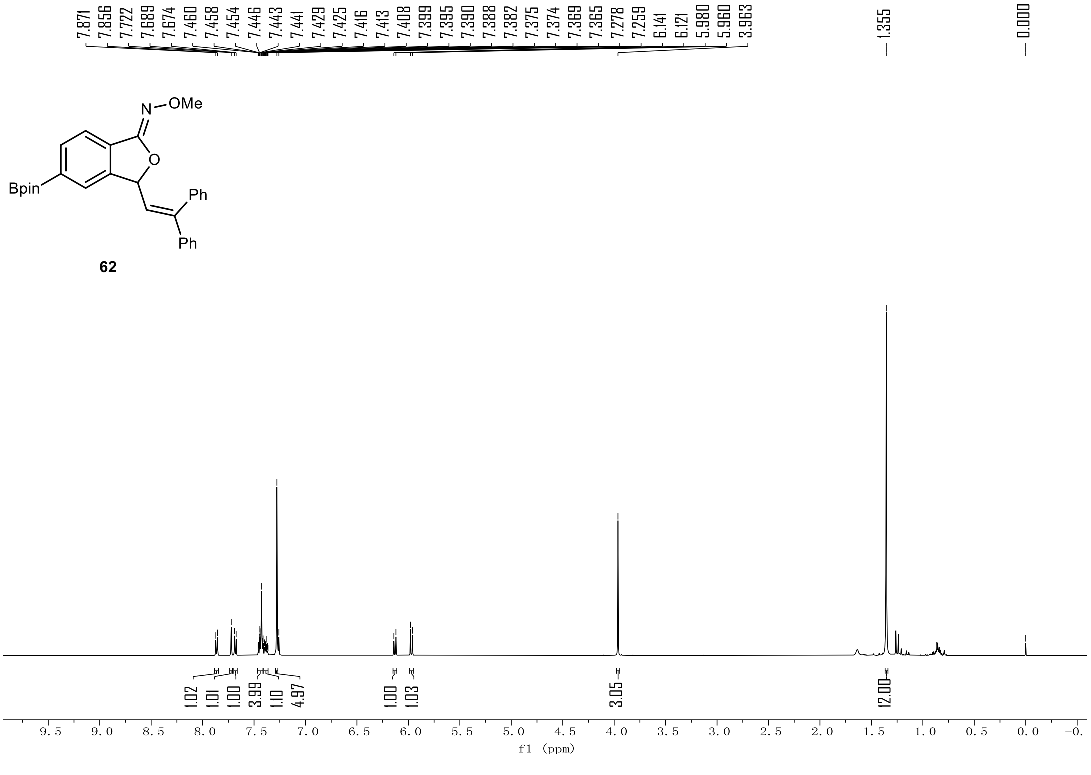
7.871
7.856
7.722
7.689
7.674
7.460
7.458
7.454
7.446
7.443
7.441
7.429
7.425
7.416
7.413
7.408
7.399
7.395
7.390
7.388
7.382
7.375
7.374
7.369
7.365
7.278
7.259
6.141
6.121
5.980
5.960
3.963
1.355
0.000
N—OMe
O
Bpin
Ph
Ph
62
1.02
1.01
1.00
3.99
1.10
4.97
1.00
1.03
3.05
12.00
9.5 9.0 8.5 8.0 7.5 7.0 6.5 6.0 5.5 5.0 4.5 4.0 3.5 3.0 2.5 2.0 1.5 1.0 0.5 0.0 -0.
f1 (ppm)

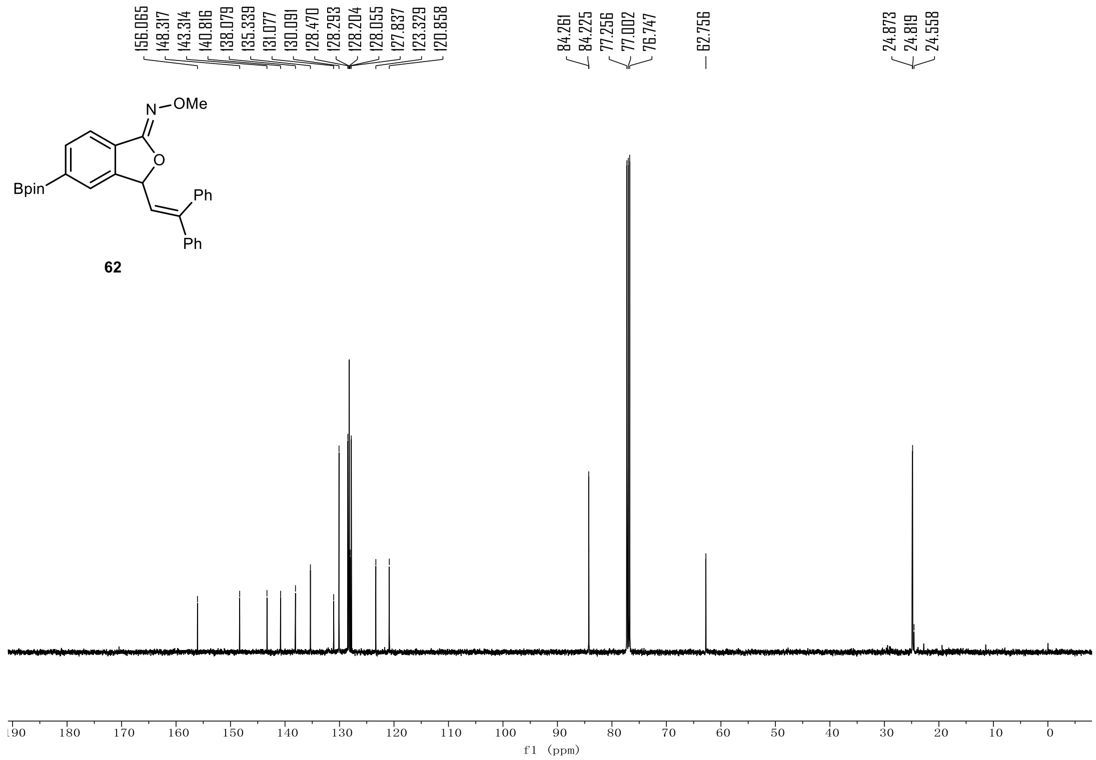
156.065
148.317
143.314
140.816
138.079
135.339
131.077
130.091
128.470
128.293
128.204
128.055
127.837
123.329
120.858
84.261
84.225
77.256
77.002
76.747
62.756
24.873
24.819
24.558
N–OMe
O
Bpin
Ph
Ph
62
f1 (ppm)

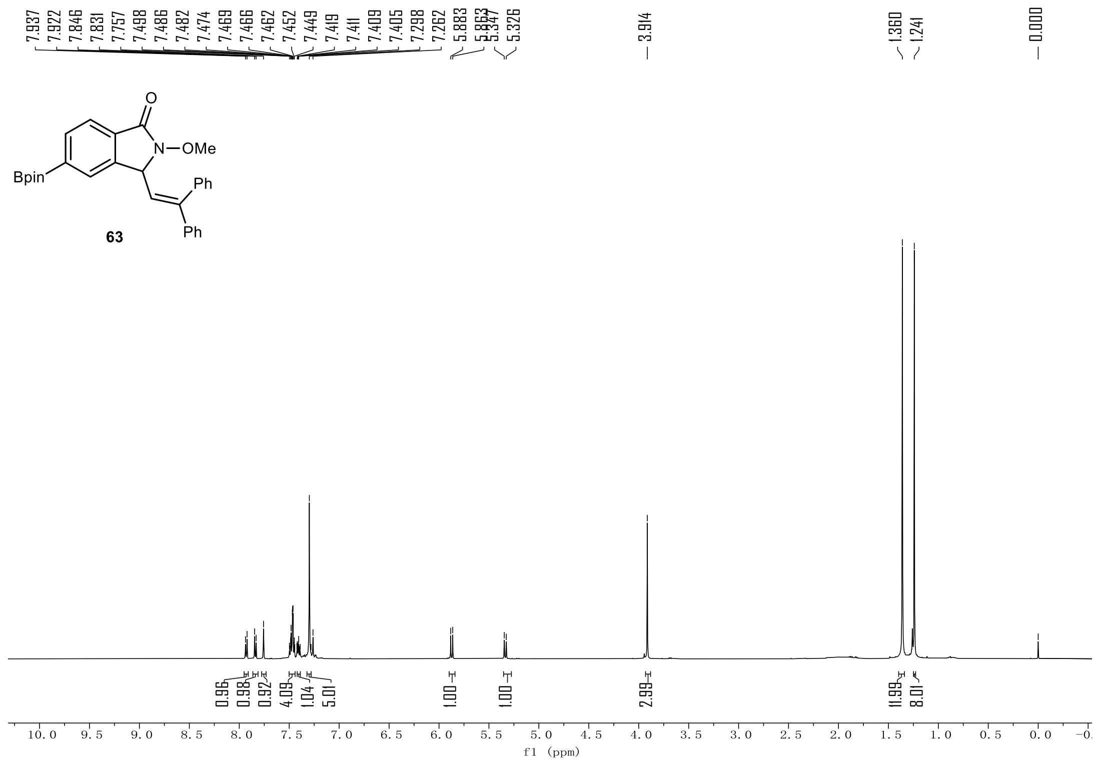
7.937
7.922
7.846
7.831
7.757
7.498
7.486
7.482
7.474
7.469
7.466
7.462
7.452
7.449
7.419
7.411
7.409
7.405
7.298
7.262
5.883
5.863
5.347
5.326
3.914
1.360
1.241
0.000
O
N–OMe
Bpin
Ph
Ph
63
0.95
0.98
0.92
4.09
1.04
5.01
1.00
1.00
2.99
11.99
8.01
f1 (ppm)

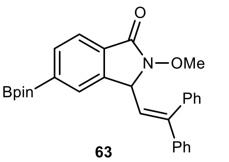
O
N−OMe
Bpin
Ph
Ph

63

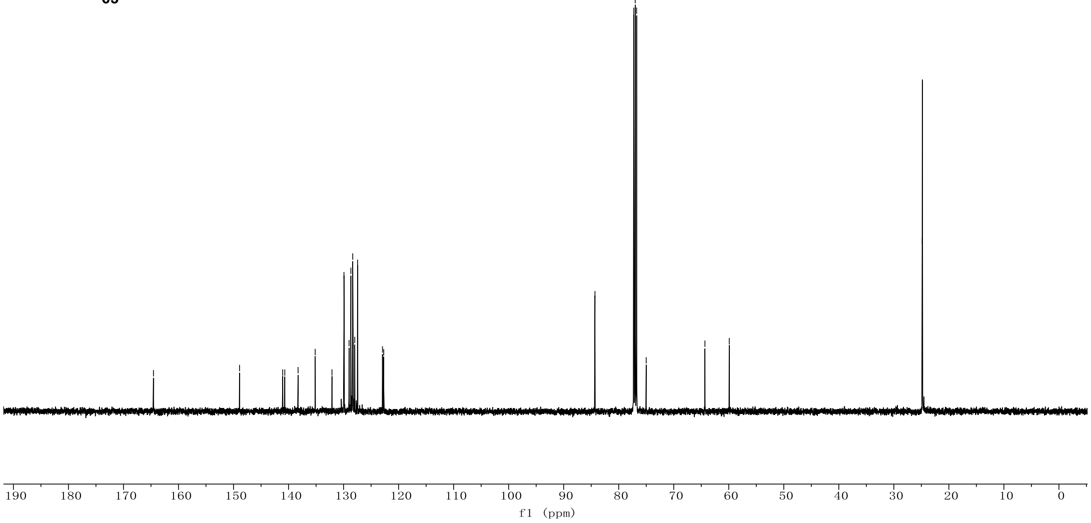
164.550
148.904
141.063
140.679
138.266
135.152
132.107
129.927
129.004
128.669
128.333
128.254
127.964
127.452
122.927
122.700
84.313
77.254
77.000
76.747
75.006
64.326
59.909
24.844
24.819
f1 (ppm)

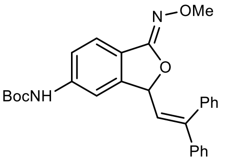
N−OMe
O
BocNH
Ph
Ph

68

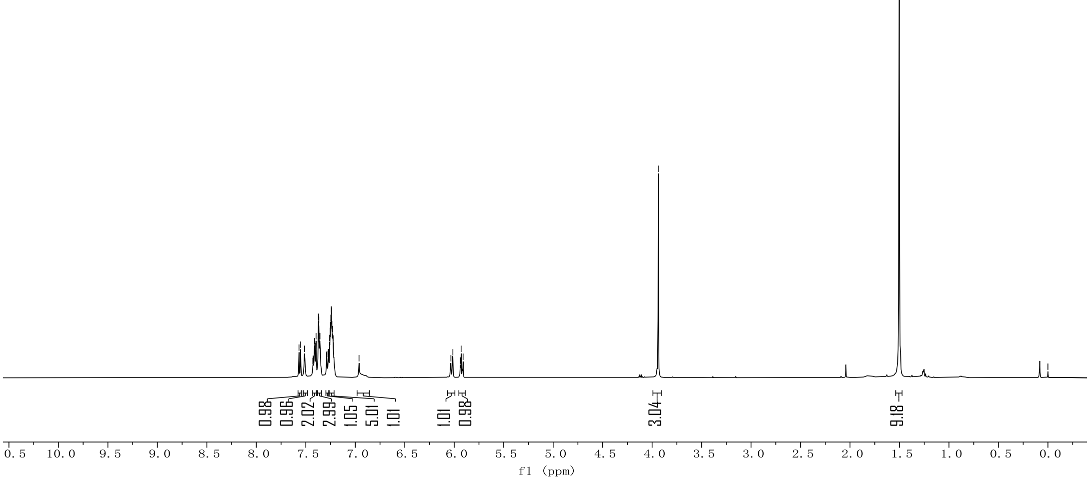
7.569
7.552
7.512
7.429
7.425
7.421
7.412
7.408
7.400
7.396
7.376
7.372
7.368
7.358
7.357
7.290
7.286
7.272
7.269
7.259
7.255
7.250
7.247
7.243
7.240
7.236
7.233
7.228
7.224
7.221
6.962
6.033
6.019
6.014
5.938
5.930
5.910
3.938
1.503
0.000
0.98
0.96
2.02
2.99
1.05
5.01
1.01
1.01
0.98
3.04
9.18
f1 (ppm)

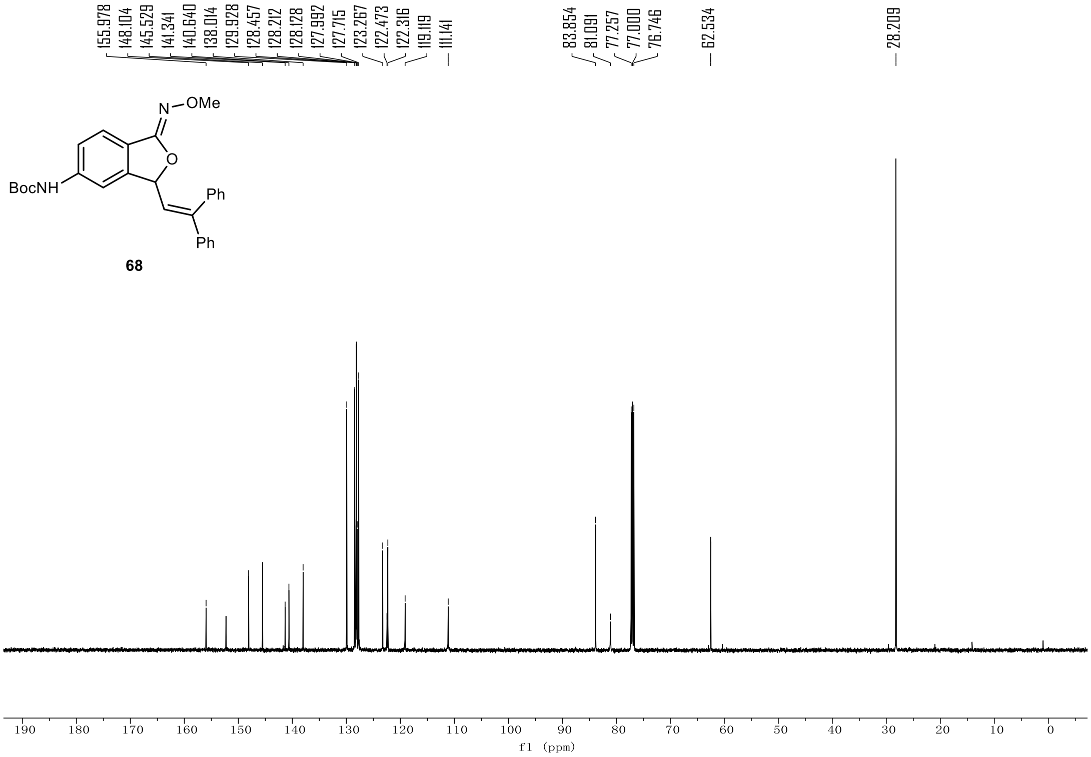
155.978
148.104
145.529
141.341
140.640
138.014
129.928
128.457
128.212
128.128
127.992
127.715
123.267
122.473
122.316
119.119
111.141
83.854
81.091
77.257
77.000
76.746
62.534
28.209
N
OMe
O
BocNH
Ph
Ph
68
f1 (ppm)

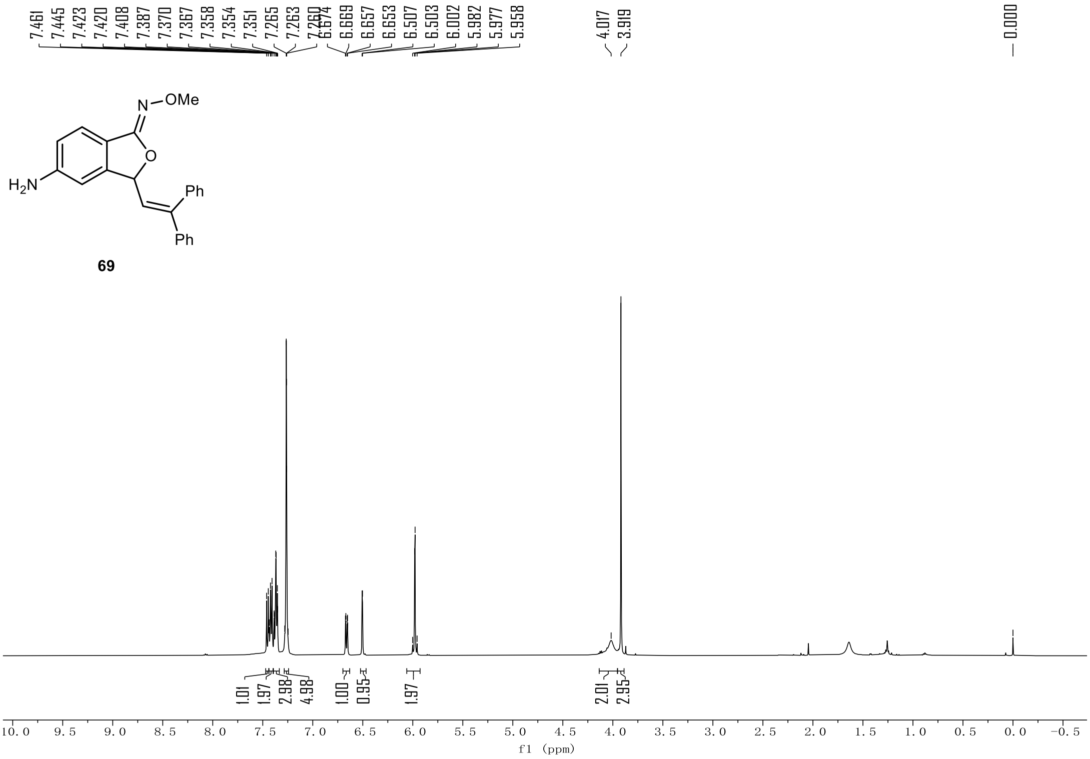
7.461
7.445
7.423
7.420
7.408
7.387
7.370
7.367
7.358
7.354
7.351
7.265
7.263
7.260
6.674
6.669
6.657
6.653
6.507
6.503
6.002
5.982
5.977
5.958
4.017
3.919
0.000
N
OMe
O
H2N
Ph
Ph
69
1.01
1.97
2.98
4.98
1.00
0.95
1.97
2.01
2.95
f1 (ppm)

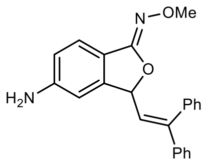
N
OMe
O
H2N
Ph
Ph

69

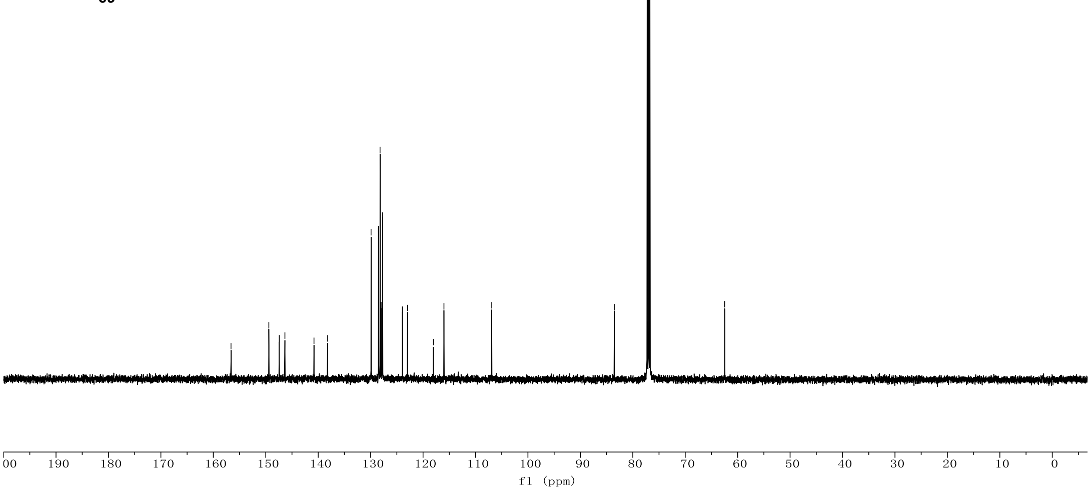
156.630
149.413
147.423
146.347
140.803
138.202
129.919
128.494
128.203
127.996
127.715
123.940
122.954
118.023
116.016
106.902
83.519
77.254
77.000
76.746
62.480
00 190 180 170 160 150 140 130 120 110 100 90 80 70 60 50 40 30 20 10 0
f1 (ppm)

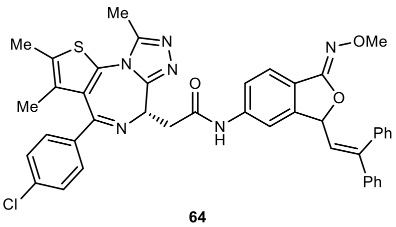
Me
Me
S
N
N
Me
Me
N
O
N
H
N
OMe
O
Ph
Ph
Cl

64

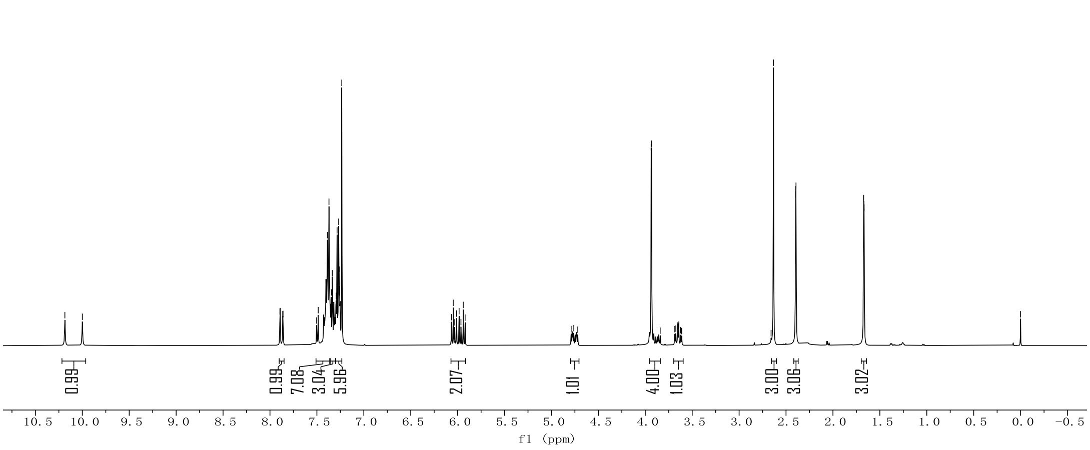
10.186
10.000
7.894
7.891
7.864
7.861
7.503
7.486
7.429
7.387
7.371
7.347
7.336
7.294
7.285
7.268
7.235
6.067
6.047
6.031
6.011
5.984
5.965
5.940
5.920
4.790
4.762
4.719
3.938
3.933
3.841
3.685
3.674
3.656
3.653
3.645
3.642
3.623
3.612
2.659
2.633
2.398
2.394
1.673
1.668
0.000
0.99
0.99
7.08
3.04
5.96
2.07
1.01
4.00
1.03
3.00
3.06
3.02
10.5 10.0 9.5 9.0 8.5 8.0 7.5 7.0 6.5 6.0 5.5 5.0 4.5 4.0 3.5 3.0 2.5 2.0 1.5 1.0 0.5 0.0 −0.5
f1 (ppm)

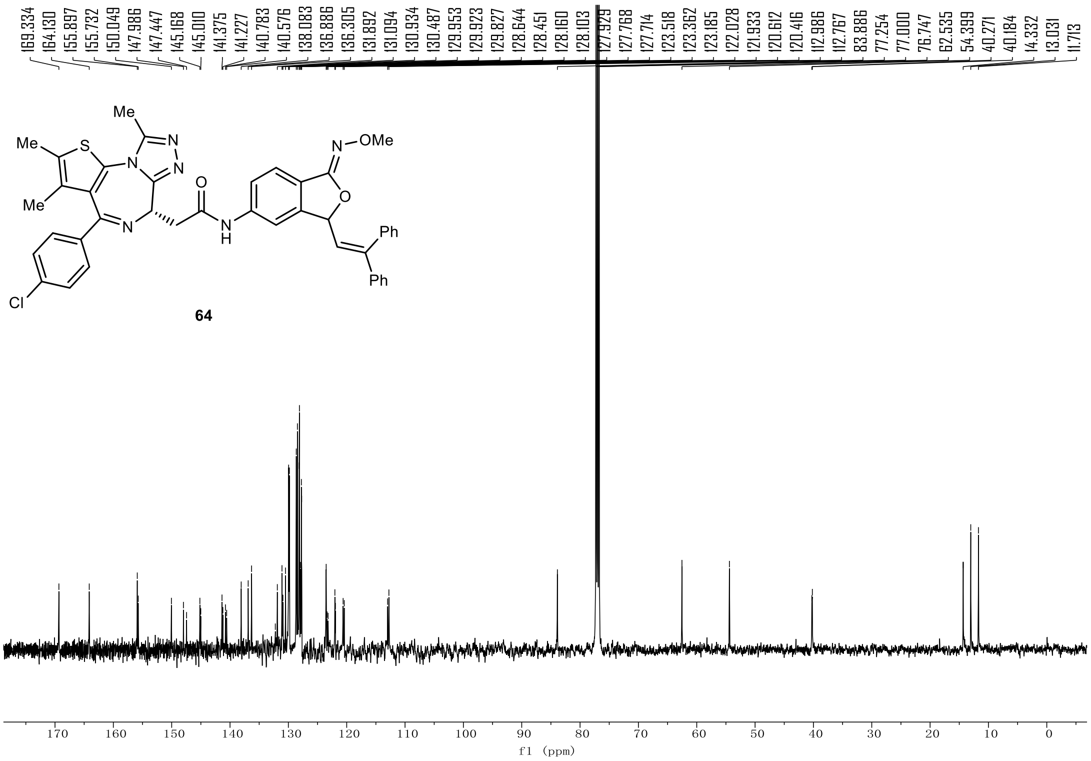
169.334
164.130
155.897
155.732
150.049
147.986
147.447
145.168
145.010
141.375
141.227
140.783
140.576
138.083
136.886
136.305
131.892
131.094
130.934
130.487
129.953
129.923
129.827
128.644
128.451
128.160
128.103
127.929
127.768
127.714
123.518
123.362
123.185
122.028
121.933
120.612
120.416
112.986
112.767
83.886
77.254
77.000
76.747
62.535
54.399
40.271
40.184
14.332
13.031
11.713
Me
S
N
N
N
O
N
OMe
O
H
Ph
Ph
Cl
64
f1 (ppm)

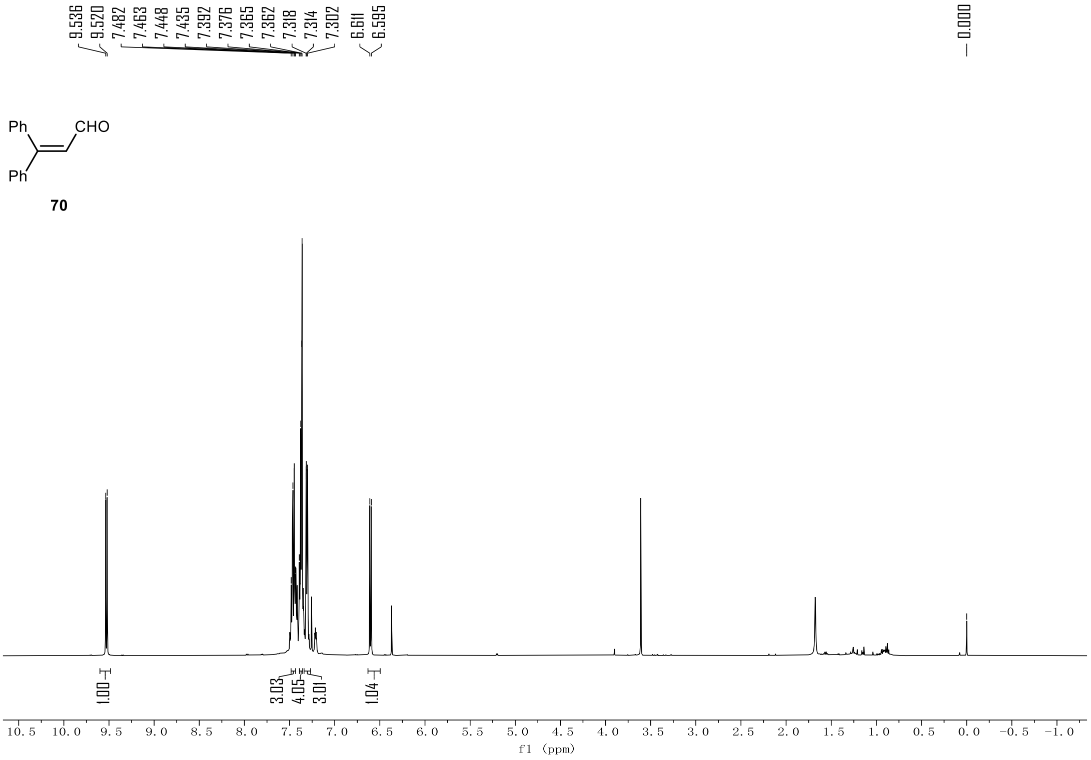
9.536
9.520
7.482
7.463
7.448
7.435
7.392
7.376
7.365
7.362
7.318
7.314
7.302
6.611
6.595
0.000
Ph
CHO
Ph
70
1.00
3.03
4.05
3.01
1.04
f1 (ppm)

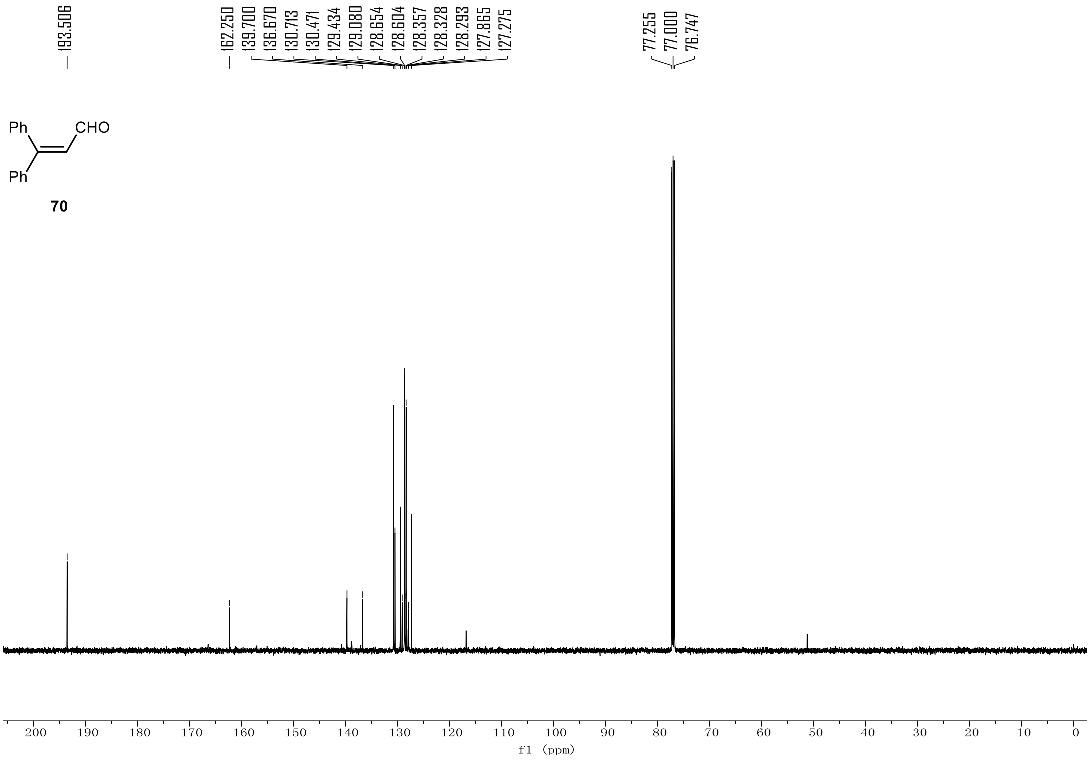
193.506
162.250
139.700
136.670
130.713
130.471
129.434
129.080
128.654
128.604
128.357
128.328
128.293
127.865
127.275
77.255
77.000
76.747
Ph
CHO
Ph
70
f1 (ppm)

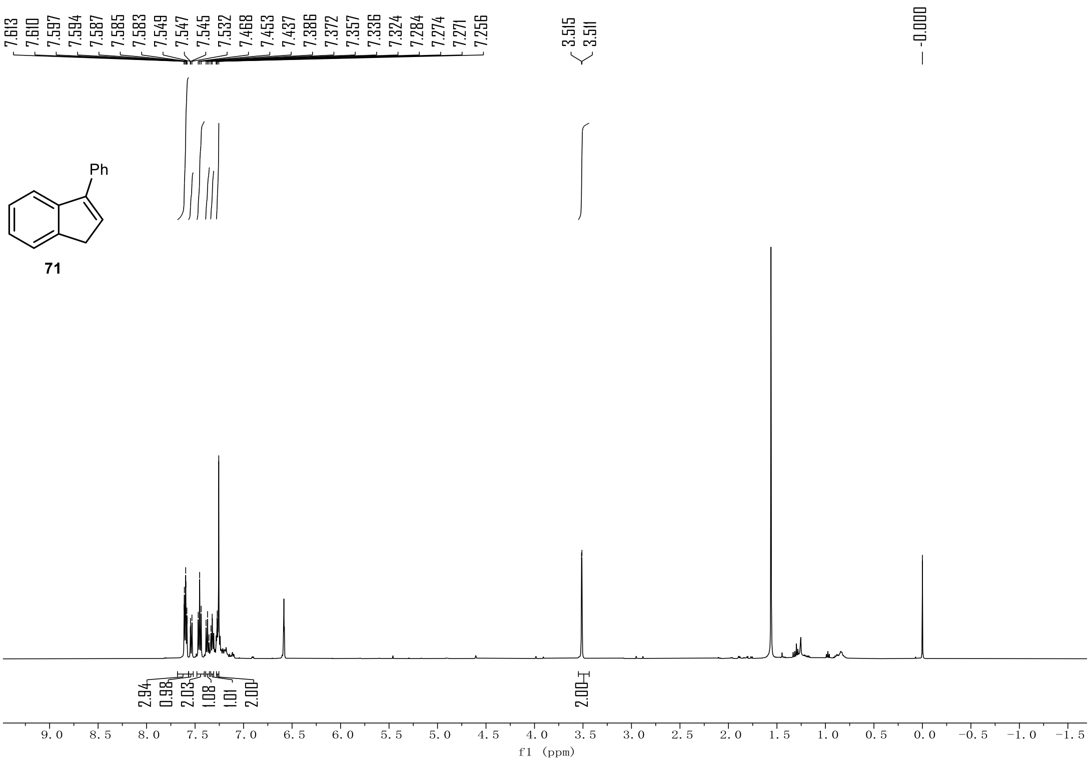
7.613
7.610
7.597
7.594
7.587
7.585
7.583
7.549
7.547
7.545
7.532
7.468
7.453
7.437
7.386
7.372
7.357
7.336
7.324
7.284
7.274
7.271
7.256
3.515
3.511
-0.000
Ph
71
2.94
0.98
2.03
1.08
1.01
2.00
2.00
f1 (ppm)

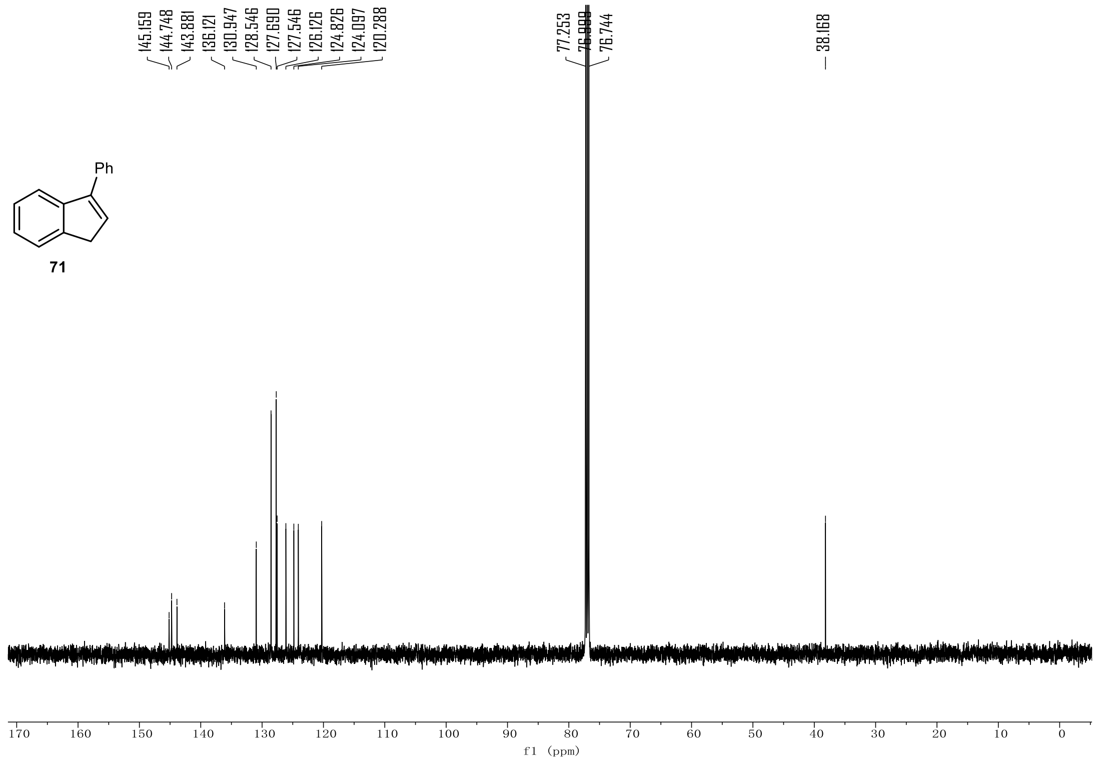

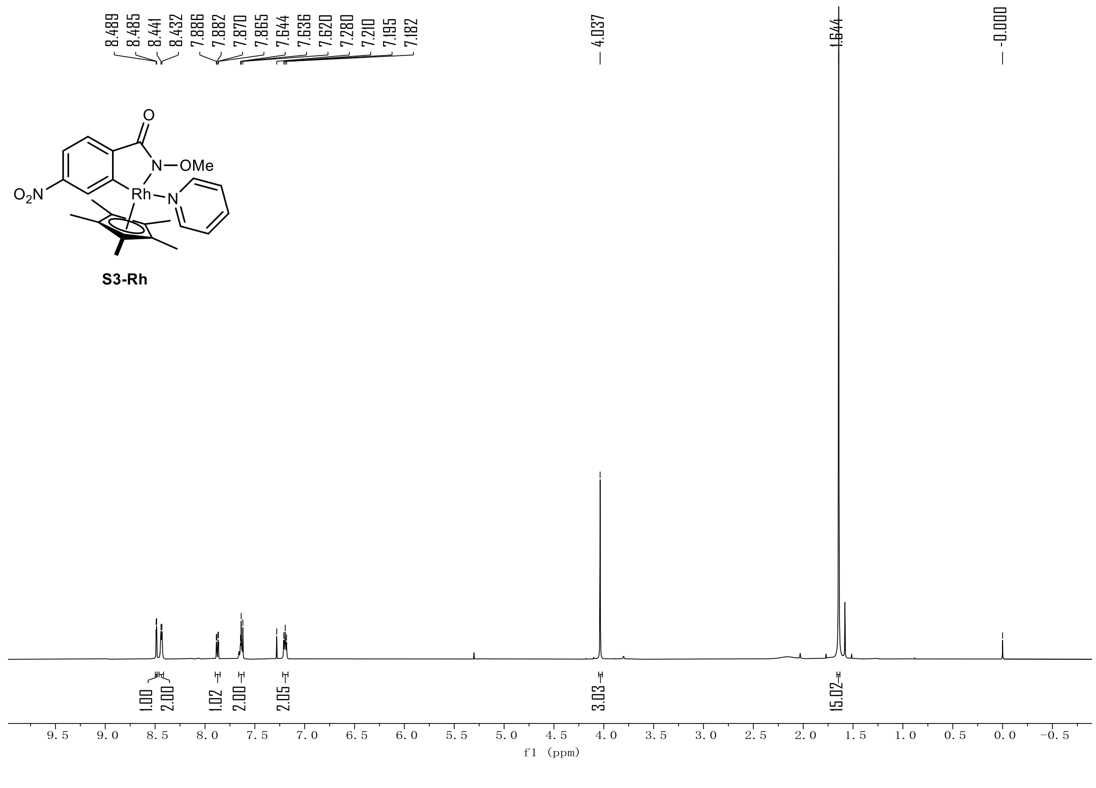

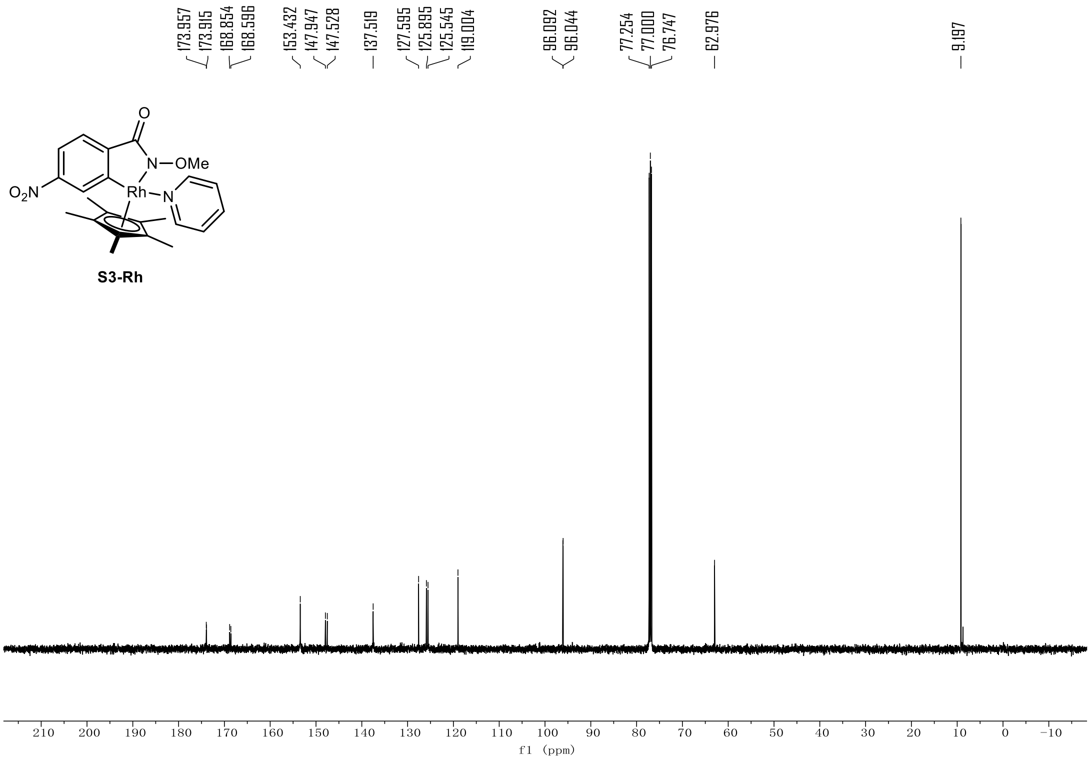
173.957
173.915
168.854
168.596
153.432
147.947
147.528
137.519
127.595
125.895
125.545
119.004
96.092
96.044
77.254
77.000
76.747
62.976
9.197
S3-Rh
f1 (ppm)

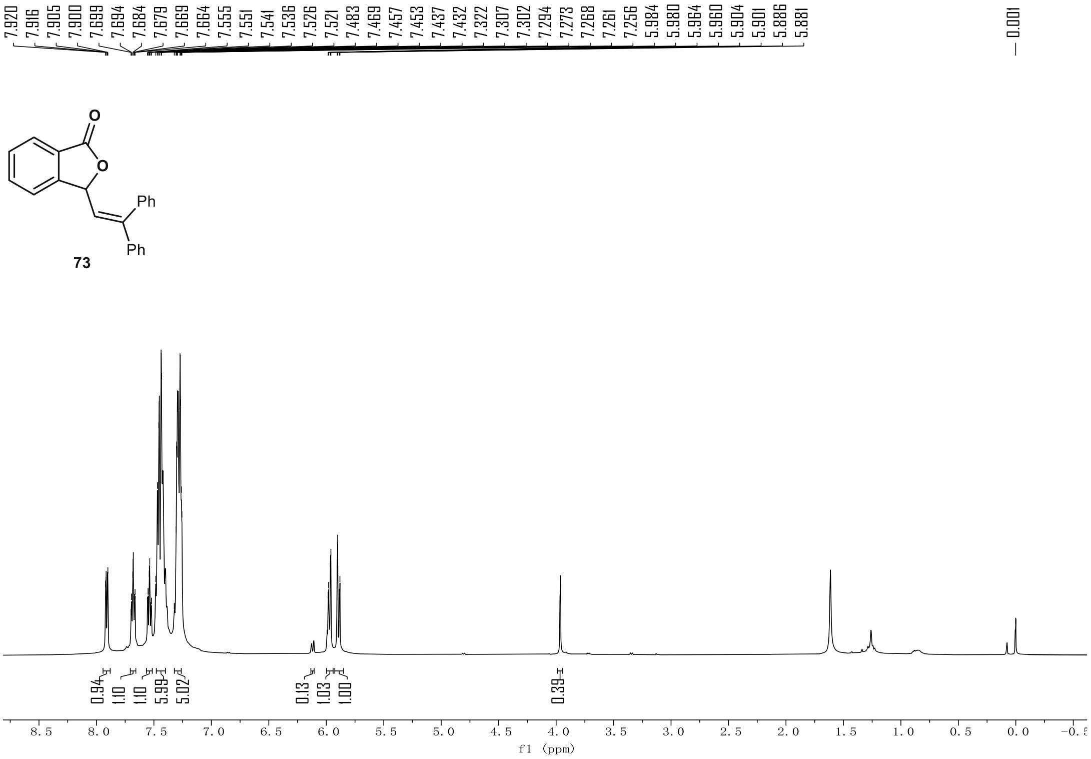
7.920
7.916
7.905
7.900
7.699
7.694
7.684
7.679
7.669
7.664
7.555
7.551
7.541
7.536
7.526
7.521
7.483
7.469
7.457
7.453
7.437
7.432
7.322
7.307
7.302
7.294
7.273
7.268
7.261
7.256
5.984
5.980
5.964
5.960
5.904
5.901
5.886
5.881
0.001
Ph
Ph
73
0.94
1.10
1.10
5.99
5.02
0.13
1.03
1.00
0.39
f1 (ppm)

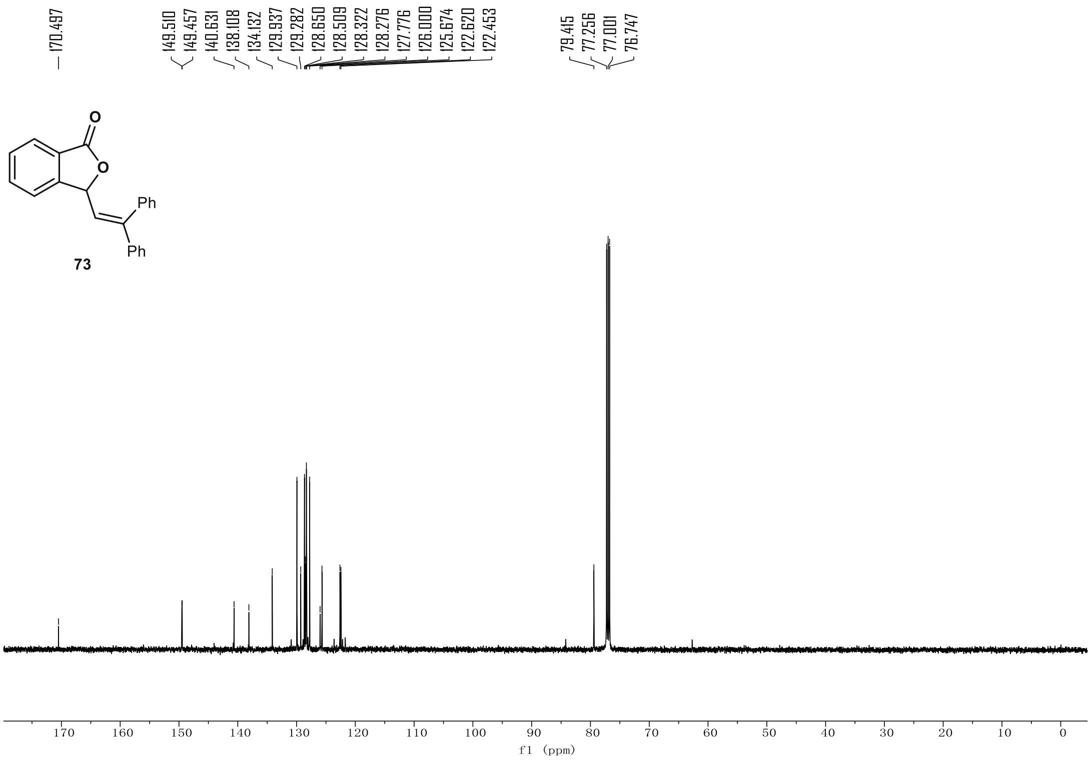

170.497
149.510
149.457
140.631
138.108
134.132
129.937
129.282
128.650
128.509
128.322
128.276
127.776
126.000
125.674
122.620
122.453
79.415
77.256
77.001
76.747
O
O
Ph
Ph
73
170
160
150
140
130
120
110
100
90
80
70
60
50
40
30
20
10
0
f1 (ppm)
