# Supplementary material for: Ring Transformation of Cyclopropenes to Benzo‐Fused Five‐Membered Oxa‐ and Aza‐Heterocycles via a Formal [4+1] Cyclization
Source: Adv Sci (Weinh). 2024 Aug 29;11(40):2407931. doi: 10.1002/advs.202407931 (PMC11516165; doi:10.1002/advs.202407931)

checkCIF/PLATON report

Structure factors have been supplied for datablock(s) 1_a_sq

THIS REPORT IS FOR GUIDANCE ONLY. IF USED AS PART OF A REVIEW PROCEDURE FOR PUBLICATION, IT SHOULD NOT REPLACE THE EXPERTISE OF AN EXPERIENCED CRYSTALLOGRAPHIC REFEREE.

No syntax errors found.   CIF dictionary   Interpreting this report

Datablock: 1_a_sq

```
Bond precision:   C-C = 0.0084 A          Wavelength=1.54178

Cell:             a=11.00000        b=13.97200       c=15.42300
                  alpha=73.4700     beta=72.0400     gamma=84.3400
Temperature:      300 K

                 Calculated                  Reported
Volume           2161.541                    2162
Space group      P -1                        P -1
Hall group       -P 1                        -P 1
Moiety formula   C23 H18 Br N O2 [+ solvent] ?
Sum formula      C23 H18 Br N O2 [+ solvent] C23 H18 Br N O2
Mr               420.28                      420.29
Dx,g cm-3        1.291                       1.291
Z                4                           4
Mu (mm-1)        2.713                       2.713
F000             856.0                       856.0
F000'            855.37
h,k,lmax         13,16,18                    0,0,0
Nref             7611                        7504
Tmin,Tmax        0.758,0.805                 0.864,0.864
Tmin'            0.688

Correction method= # Reported T Limits: Tmin=0.864 Tmax=0.864
AbsCorr = MULTI-SCAN

Data completeness= 0.986           Theta(max)= 66.453

R(reflections)= 0.0898( 4846)                     wR2(reflections)=
                                                  0.2935( 7504)
S = 1.065                  Npar= 488
```

The following ALERTS were generated. Each ALERT has the format
test-name_ALERT_alert-type_alert-level.
Click on the hyperlinks for more details of the test.

Alert level C

```
PLAT084_ALERT_3_C High wR2 Value (i.e. > 0.25) ...................       0.29 Report
PLAT141_ALERT_4_C s.u. on a - Axis Small or Missing .............    0.00000 Ang.
PLAT142_ALERT_4_C s.u. on b - Axis Small or Missing .............    0.00000 Ang.
PLAT143_ALERT_4_C s.u. on c - Axis Small or Missing .............    0.00000 Ang.
PLAT144_ALERT_4_C s.u. on alpha    Small or Missing .............     0.0000 Degree
PLAT145_ALERT_4_C s.u. on beta     Small or Missing .............     0.0000 Degree
PLAT146_ALERT_4_C s.u. on gamma    Small or Missing .............     0.0000 Degree
PLAT151_ALERT_1_C No s.u. (esd) Given on Volume .................     Please Do !
PLAT341_ALERT_3_C Low Bond Precision on  C-C Bonds ..............    0.00844 Ang.
PLAT906_ALERT_3_C Large K Value in the Analysis of Variance ......      5.373 Check
PLAT911_ALERT_3_C Missing FCF Refl Between Thmin & STh/L=    0.595        107 Report
               -1  1  0,   1  1  0,   2  2  0,  -1  3  0,   1  3  0,   2  3  0,
               -2  4  0,   2  4  0,  -3  5  0,   0  5  0,   3  5  0,  -3  6  0,
               -2  6  0,   2  6  0,   3  6  0,  -1  7  0,   1  7  0,  -2 12  0,
                2 12  0,  -4 15  0,   0 15  0,  -2 -5  1,   1 -5  1,   3 -4  1,
                4 -4  1, -12 -2  1,  12 -2  1, -11  0  1, -12  2  1,   1  2  1,
               12  3  1,   1  5  1,   1  7  1,   5 15  1,   2 16  1, -11  0  2,
               -2  0  2,  -2  1  2, -11  2  2,  -1  2  2,   5-12  3,   1 -4  3,
               -2 -3  3,  -2  5  3,   1  5  3,   6 15  3,  -2-14  4,  -1 -4  4,
               -3 -2  4,   1  1  4,   2  1  4,  13  1  4,  13  2  4,  -3  4  4,
               -1  6  4,  -2 16  4,   1-14  5,  13  1  5,  13  2  5,   3  6  5,
               -1-12  6,  -1 15  6,  13  2  7,  -7 -7  8,   3  2  8,   9  9  9,
               -7 -1 10,  -3 14 10,   6 -9 11,  -4 -7 11,  -4 -5 12,  -5 10 12,
               -4 11 12,   4 15 12,  -2 -6 13,   3 -4 13,   7 11 13,   3 -5 14,
                0 13 14,   5 -5 15,  10  1 15,  10  7 15,  -3  8 15,  -1 11 15,
                3 13 15,   1 -3 16,  -1 -1 16,   8 -1 16,  -2  1 16,  -1  4 16,
                2 -1 17,   0  1 17,   8  2 17,   1  9 17,   5 10 17,   3  2 18,
```

Alert level G

```
PLAT605_ALERT_4_G Largest Solvent Accessible VOID in the Structure        294 A**3
PLAT869_ALERT_4_G ALERTS Related to the Use of SQUEEZE  Suppressed          ! Info
PLAT881_ALERT_1_G No Datum for _diffrn_reflns_av_R_equivalents ...     Please Do !
PLAT883_ALERT_1_G No Info/Value for _atom_sites_solution_primary .     Please Do !
PLAT913_ALERT_3_G Missing # of Very Strong Reflections in FCF ....          2 Note
                1  1  4,   2  1  4,
PLAT941_ALERT_3_G Average HKL Measurement Multiplicity ..........        1.0 Low
PLAT961_ALERT_5_G Dataset Contains no Negative Intensities .......     Please Check
PLAT965_ALERT_2_G The SHELXL WEIGHT Optimisation has not Converged     Please Check
PLAT967_ALERT_5_G Note: Two-Theta Cutoff Value in Embedded .res ..      133.0 Degree
PLAT969_ALERT_5_G The 'Henn et al.' R-Factor-gap value ...........       3.54 Note
             Predicted wR2: Based on SigI**2  8.30 or SHELX Weight 28.51
PLAT978_ALERT_2_G Number C-C Bonds with Positive Residual Density.          0 Info
PLAT992_ALERT_5_G Repd & Actual _reflns_number_gt Values Differ by          2 Check
```

0 **ALERT level A** = Most likely a serious problem - resolve or explain
0 **ALERT level B** = A potentially serious problem, consider carefully
11 **ALERT level C** = Check. Ensure it is not caused by an omission or oversight
12 **ALERT level G** = General information/check it is not something unexpected

```
3 ALERT type 1 CIF construction/syntax error, inconsistent or missing data
2 ALERT type 2 Indicator that the structure model may be wrong or deficient
6 ALERT type 3 Indicator that the structure quality may be low
8 ALERT type 4 Improvement, methodology, query or suggestion
4 ALERT type 5 Informative message, check
```

It is advisable to attempt to resolve as many as possible of the alerts in all categories. Often the minor alerts point to easily fixed oversights, errors and omissions in your CIF or refinement strategy, so attention to these fine details can be worthwhile. In order to resolve some of the more serious problems it may be necessary to carry out additional measurements or structure refinements. However, the purpose of your study may justify the reported deviations and the more serious of these should normally be commented upon in the discussion or experimental section of a paper or in the "special_details" fields of the CIF. checkCIF was carefully designed to identify outliers and unusual parameters, but every test has its limitations and alerts that are not important in a particular case may appear. Conversely, the absence of alerts does not guarantee there are no aspects of the results needing attention. It is up to the individual to critically assess their own results and, if necessary, seek expert advice.

Publication of your CIF in IUCr journals

A basic structural check has been run on your CIF. These basic checks will be run on all CIFs submitted for publication in IUCr journals (*Acta Crystallographica*, *Journal of Applied Crystallography*, *Journal of Synchrotron Radiation*); however, if you intend to submit to *Acta Crystallographica Section C* or *E* or *IUCrData*, you should make sure that full publication checks are run on the final version of your CIF prior to submission.

Publication of your CIF in other journals

Please refer to the *Notes for Authors* of the relevant journal for any special instructions relating to CIF submission.

PLATON version of 06/01/2024; check.def file version of 05/01/2024

Datablock 1_a_sq - ellipsoid plot

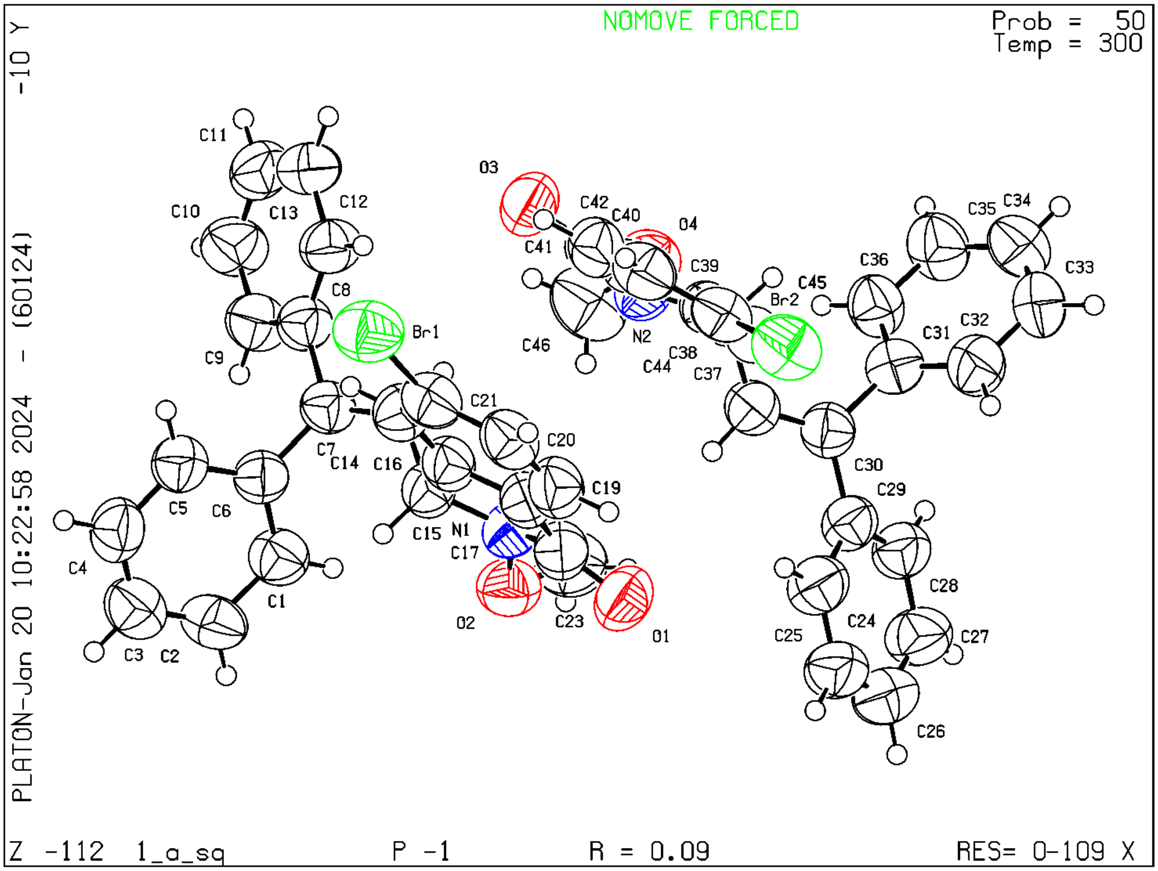

Supplement: Supplementary file 2 — Supporting Information [file ADVS-11-2407931-s002.zip › 43-checkcif.pdf]
