# Supplementary material for: Ring Transformation of Cyclopropenes to Benzo‐Fused Five‐Membered Oxa‐ and Aza‐Heterocycles via a Formal [4+1] Cyclization
Source: Adv Sci (Weinh). 2024 Aug 29;11(40):2407931. doi: 10.1002/advs.202407931 (PMC11516165; doi:10.1002/advs.202407931)

checkCIF/PLATON report

Structure factors have been supplied for datablock(s) 1_a

THIS REPORT IS FOR GUIDANCE ONLY. IF USED AS PART OF A REVIEW PROCEDURE FOR PUBLICATION, IT SHOULD NOT REPLACE THE EXPERTISE OF AN EXPERIENCED CRYSTALLOGRAPHIC REFEREE.

No syntax errors found. CIF dictionary Interpreting this report

Datablock: 1_a

Bond precision: C-C = 0.0055 A Wavelength=1.54178

Cell:	a=11.6213(7)	b=16.4338(11)	c=11.4359(8)
	alpha=90	beta=115.688(3)	gamma=90
Temperature:	300 K		

	Calculated	Reported
Volume	1968.2(2)	1968.2(2)
Space group	P 21/c	P 21/c
Hall group	-P 2ybc	-P 2ybc
Moiety formula	C23 H18 Br N O2	?
Sum formula	C23 H18 Br N O2	C23 H18 Br N O2
Mr	420.28	420.29
Dx,g cm-3	1.418	1.418
Z	4	4
Mu (mm-1)	2.979	2.979
F000	856.0	856.0
F000’	855.37	
h,k,lmax	14,20,14	14,20,14
Nref	3881	3829
Tmin,Tmax	0.545,0.585	0.864,0.864
Tmin’	0.495	

Correction method= # Reported T Limits: Tmin=0.864 Tmax=0.864
AbsCorr = MULTI-SCAN

Data completeness= 0.987 Theta(max)= 72.271

R(reflections)= 0.0897(3292) wR2(reflections)= 0.2464(3829)

S = 1.054 Npar= 244

The following ALERTS were generated. Each ALERT has the format
test-name_ALERT_alert-type_alert-level.
Click on the hyperlinks for more details of the test.

Alert level C

```
PLAT767_ALERT_4_C INS Embedded LIST 6 Instruction Should be LIST 4     Please Check
PLAT906_ALERT_3_C Large K Value in the Analysis of Variance ......      3.056 Check
PLAT911_ALERT_3_C Missing FCF Refl Between Thmin & STh/L=    0.600         41 Report
                0  8  0,   0  8  1,  -7  0  2,  -6  0  2,  -5  0  2,  -4  0  2,
               -3  0  2,  -2  0  2,  -6  1  2,  -4  6  2,   1  7  2,  -3  1  3,
               -6  3  3,  -5  3  3,  -5  5  3,  -5  0  4,  -3  0  4,  -2  0  4,
               -6  1  4,  -5  1  4,  -4  1  4,  -3  1  4,  -5  4  4,  -5  8  4,
               -5  1  5,  -4  1  5,  -5  2  5,  -5  3  5,  -4  3  5,  -5  0  6,
               -4  0  6,  -3  0  6,  -4  1  6,  -3  1  6, -13  0  8,  -3  0 12,
               -3  1 12,  -4  1 13,  -3  1 13,  -4  2 13,  -3  2 13,
PLAT972_ALERT_2_C Check Calcd Resid. Dens.  0.82Ang From Br1            -2.17 eA-3
PLAT976_ALERT_2_C Check Calcd Resid. Dens.  0.97Ang From O2      .      -0.50 eA-3
PLAT976_ALERT_2_C Check Calcd Resid. Dens.  1.06Ang From N1      .      -0.45 eA-3
```

Alert level G

```
PLAT883_ALERT_1_G No Info/Value for _atom_sites_solution_primary .     Please Do !
PLAT912_ALERT_4_G Missing # of FCF Reflections Above STh/L=  0.600         11 Note
PLAT933_ALERT_2_G Number of HKL-OMIT Records in Embedded .res File          1 Note
               -5  8  4,
PLAT969_ALERT_5_G The 'Henn et al.' R-Factor-gap value ..........       3.86 Note
              Predicted wR2: Based on SigI**2  6.38 or SHELX Weight 24.16
PLAT978_ALERT_2_G Number C-C Bonds with Positive Residual Density.          2 Info
```

0 **ALERT level A** = Most likely a serious problem - resolve or explain
0 **ALERT level B** = A potentially serious problem, consider carefully
6 **ALERT level C** = Check. Ensure it is not caused by an omission or oversight
5 **ALERT level G** = General information/check it is not something unexpected

1 ALERT type 1 CIF construction/syntax error, inconsistent or missing data
5 ALERT type 2 Indicator that the structure model may be wrong or deficient
2 ALERT type 3 Indicator that the structure quality may be low
2 ALERT type 4 Improvement, methodology, query or suggestion
1 ALERT type 5 Informative message, check

It is advisable to attempt to resolve as many as possible of the alerts in all categories. Often the minor alerts point to easily fixed oversights, errors and omissions in your CIF or refinement strategy, so attention to these fine details can be worthwhile. In order to resolve some of the more serious problems it may be necessary to carry out additional measurements or structure refinements. However, the purpose of your study may justify the reported deviations and the more serious of these should normally be commented upon in the discussion or experimental section of a paper or in the "special_details" fields of the CIF. checkCIF was carefully designed to identify outliers and unusual parameters, but every test has its limitations and alerts that are not important in a particular case may appear. Conversely, the absence of alerts does not guarantee there are no aspects of the results needing attention. It is up to the individual to critically assess their own results and, if necessary, seek expert advice.

Publication of your CIF in IUCr journals

A basic structural check has been run on your CIF. These basic checks will be run on all CIFs submitted for publication in IUCr journals (*Acta Crystallographica*, *Journal of Applied Crystallography*, *Journal of Synchrotron Radiation*); however, if you intend to submit to *Acta Crystallographica Section C* or *E* or *IUCrData*, you should make sure that full publication checks are run on the final version of your CIF prior to submission.

Publication of your CIF in other journals

Please refer to the *Notes for Authors* of the relevant journal for any special instructions relating to CIF submission.

PLATON version of 06/01/2024; check.def file version of 05/01/2024

Datablock 1_a - ellipsoid plot

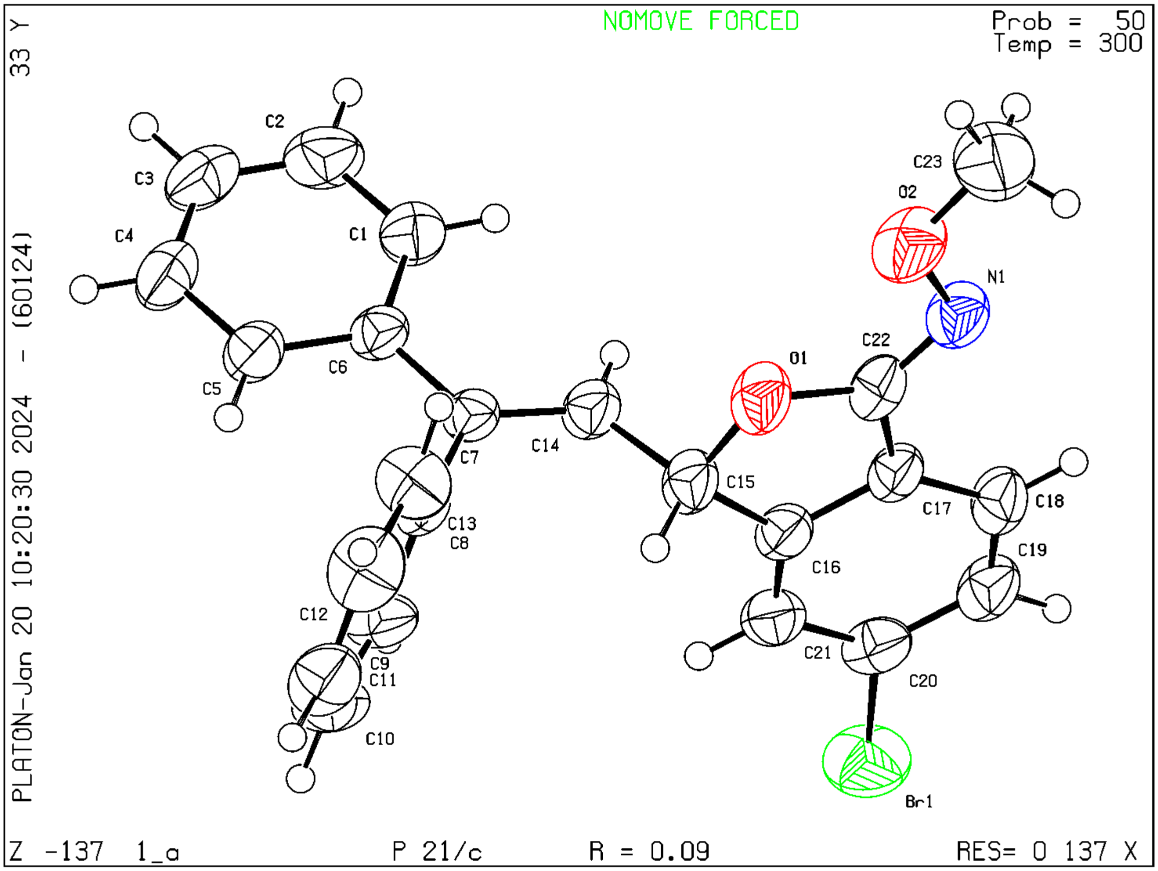

Supplement: Supplementary file 2 — Supporting Information [file ADVS-11-2407931-s002.zip › 5-checkcif.pdf]
